# Computer-Aided Design and Biological Evaluation of Diazaspirocyclic D_4_R Antagonists

**DOI:** 10.1021/acschemneuro.4c00086

**Published:** 2024-06-07

**Authors:** Caleb A. H. Jones, Benjamin P. Brown, Daniel C. Schultz, Julie Engers, Valerie M. Kramlinger, Jens Meiler, Craig W. Lindsley

**Affiliations:** † Warren Center for Neuroscience Drug Discovery, Vanderbilt University School of Medicine, Nashville, Tennessee 37232, United States; ‡ Department of Pharmacology, Vanderbilt University School of Medicine, Nashville, Tennessee 37232, United States; § Department of Chemistry, Vanderbilt University, Nashville, Tennessee 37232, United States; ∥ Center for Structural Biology, Vanderbilt University, Nashville, Tennessee 37232, United States; ⊥ Center for Applied AI in Protein Dynamics, Vanderbilt University, Nashville, Tennessee 37232, United States; # Institute for Drug Discovery, Leipzig University Medical School, Leipzig SAC 04103, Germany

**Keywords:** dopamine receptors, D_4_R antagonism, Parkinson’s disease

## Abstract

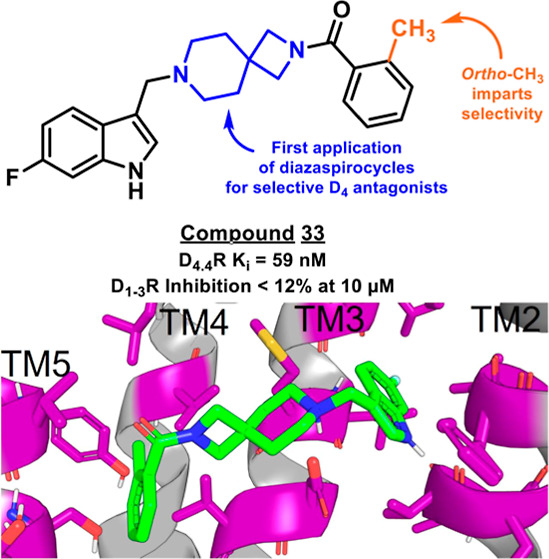

Parkinson’s disease (PD) is a neurodegenerative disorder characterized by the progressive loss of dopaminergic neurons in the substantia nigra, resulting in motor dysfunction. Current treatments are primarily centered around enhancing dopamine signaling or providing dopamine replacement therapy and face limitations such as reduced efficacy over time and adverse side effects. To address these challenges, we identified selective dopamine receptor subtype 4 (D_4_R) antagonists not previously reported as potential adjuvants for PD management. In this study, a library screening and artificial neural network quantitative structure–activity relationship (QSAR) modeling with experimentally driven library design resulted in a class of spirocyclic compounds to identify candidate D_4_R antagonists. However, developing selective D_4_R antagonists suitable for clinical translation remains a challenge.

## Introduction

Parkinson’s disease (PD) is a debilitating neurodegenerative disorder characterized by progressive motor dysfunction resulting from the degeneration of dopaminergic neurons in the substantia nigra.^1,2^ The resulting dopamine deficiency leads to the classic motor symptoms of PD, including bradykinesia, resting tremors, and rigidity.^1^ While current treatments, such as enhancing dopamine signaling and providing dopamine replacement therapy, have been effective in alleviating motor symptoms in the early stages of PD, the need for innovative therapeutic approaches is underscored by the challenges of maintaining their long-term efficacy and minimizing the risk of side effects, including medication-induced dyskinesias.^2,3^ One promising avenue of exploration lies in the design and development of selective dopamine receptor subtype 4 (D_4_R) antagonists as potential adjuvants for PD management.^4−6^

Dopamine receptors are divided into two families based on structural similarities, function, and pharmacological properties: the D_1_-like receptor family, which includes primarily the D_1_R and D_5_R subtypes, and the D_2_-like receptor family, which includes D_2_R, D_3_R, and D_4_R.^7−9^ Functionally, these two families have opposing mechanisms, with D_1_-like receptors stimulating adenyl cyclase through G_αs_ signaling and D_2_-like receptors inhibiting adenyl cyclase through G_αi/o_ signaling.^7^ Further receptor subtype heterogeneity can be found at the level of genetic polymorphisms. D_4_R itself comprises 10 different genotypes, with D_4.2_, D_4.4_, and D_4.7_ being the most prevalent of these.^10−12^ The pharmacological management of PD currently focuses primarily on enhancing dopamine signaling through D_2_R, such as by providing dopamine precursor therapy with levodopa or through direct agonism with pramipexole or ropinirole.^13−25^

**Figure 1:**
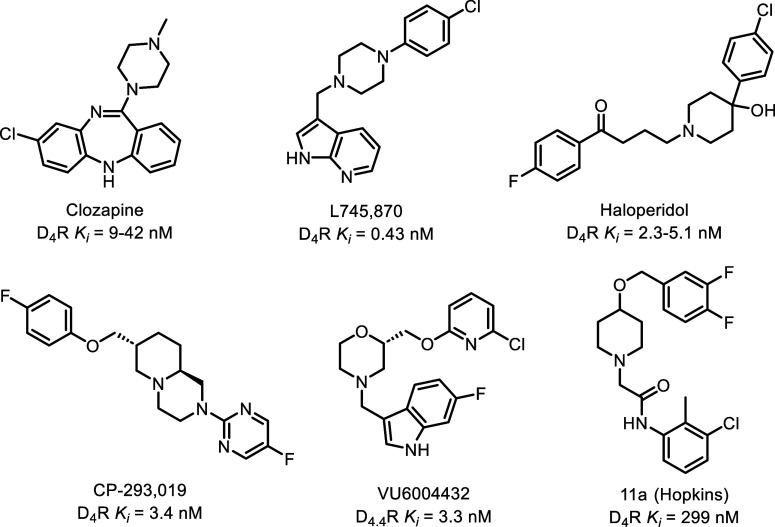
Selected historical compounds demonstrating antagonism at D_4_R.^9,30,49−59^

D_4_R has garnered increasing attention in recent years due to its distinctive expression pattern within the central nervous system and its potential role in modulating dopamine signaling.^5,6,26^ Unlike other dopamine receptor subtypes, D_4_R is primarily located in the frontal cortex and limbic system, areas that are associated with cognitive and emotional processes, and consequently has been implicated largely in neuropsychiatric conditions (though D_4_R is also expressed in the periphery).^27−40^ Early D_4_R antagonists were considered as potential therapeutic avenues for diseases such as addiction and attention-deficit/hyperactivity disorder (ADHD).^41−44^ Additionally, due to the expression of D_4_R within the basal ganglia, which is associated with the development of dyskinesias in PD patients, research has also unveiled the involvement of D_4_R in motor control, making it a compelling target in the context of PD for the treatment of levodopa-induced dyskinesia (LID).^3,4,33,45−48^ Consequentially, interest in the development of selective D_4_R antagonists has increased in recent decades, selected examples of which can be seen in Figure 1. The approved antipsychotics clozapine and haloperidol have also been included for reference due to their historical significance, though these are not selective for D_4_R.^9,30,49−59^

The central challenge in designing D_4_R antagonists as an adjuvant therapy for PD lies in obtaining selectivity for D_4_R over the other dopamine receptor subtypes, action at which could produce undesired side effects. For instance, antagonism or partial agonism of D_2_R has been demonstrated to worsen Parkinsonism, while action at D_1_R in conjunction with levodopa administration is associated with increased LID severity.^60−64^ Therefore, the pursuit of D_4_R antagonists for PD therapy demands meticulous attention to the selectivity and efficacy of the designed compounds. Recent advances in synthetic chemistry, structural biology, and pharmacology have enabled the design and characterization of diverse selective D_4_R antagonists, as exemplified in several key studies.^59,65−69^ Building off of these rich structure–activity relationship data, we disclose herein the development of a novel class of potent, selective D_4_R antagonists suitable for further preclinical optimization.

## Results

### Ligand-Based Ultralarge Library Screening to Identify Candidate D_4_R Antagonists

To identify new D_4_R antagonists, we first performed ligand-based ultralarge library screening using multitask classification artificial neural network (ANN) quantitative structure–activity relationship (QSAR) models (see Computational Methods and Materials in the Supporting Information). We trained four unique QSAR models on publicly available confirmatory screening data (molecules had reported IC_50_ and/or *K*_i_/*K*_d_ values) from PubChem, one each for D_2_R, D_3_R, D_4_R, and D_5_R. Each model was trained to predict the likelihood that a molecule is active at or below the following thresholds: 1, 10, 100, 1000, and 10,000 nM. Two primary metrics guided our analysis: (1) the probability that a molecule is active against D_4_R at or below 10 nM and (2) the predicted selectivity for D_4_R, where selectivity is given by the equation below.
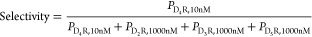
where *P*_D_4_R,10nM_ is the QSAR-predicted probability of a molecule to be active at or below 10 nM, *P*_D_4_R,1000nM_ is the same metric for D_2_R at or below 1000 nM, etc. Our formulation of selectivity specifically evaluates the likelihood of a molecule being selective for D_4_R at 2 orders of magnitude (active at 10 nM D_4_R vs 1000 nM D_2_, D_3_, and D_5_).

**Figure 2:**
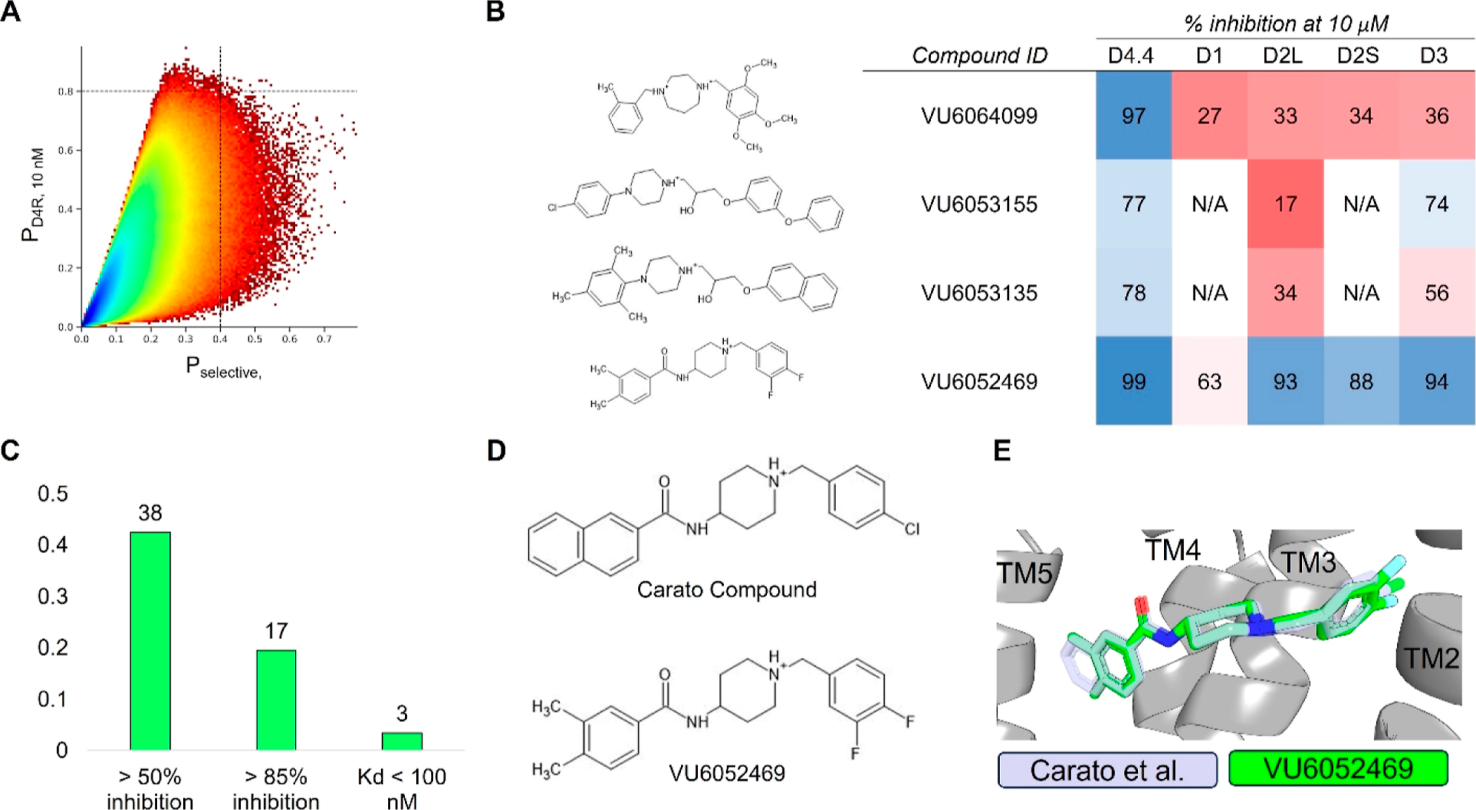
Virtual high-throughput screening for D_4_R antagonists. (A) Predicted D_4_R activity vs selectivity from the ligand-based multitask ANN QSAR model ultralarge library virtual high-throughput screening. Dashed lines indicate QSAR-predicted active classification probabilities at or greater than 80% (horizontal) and 40% (vertical) for D_4_R 10 nM activity and overall selectivity, respectively. Plot color is contoured by the density of molecules, with higher-density regions appearing blue and lower-density regions appearing red. (B) Sample molecules identified during the virtual high-throughput screening. (C) D_4_R hit-rate for experimentally validated molecules. (D) 2D structures of Carato et al.: compound **22**(71) and *VU6052469*. (E) Overlay of docked poses of Carato et al.: compound **22**(71) and *VU6052469* within D_4_R.

We applied our QSAR models to screen over 1 billion molecules sourced from LifeChemicals and the Enamine REAL database (Figure 2A). Compounds with 10 nM D_4_R activity prediction scores at or above 0.8 were moved forward for further analysis. Preference was given to compounds also exhibiting a selectivity score exceeding 0.4. We performed property-based flexible alignment^70^ of a subset of 500 molecules to the crystallographically bound pose of the D_4_R-selective antagonist L-745,870,^68^ followed by visual inspection. Ultimately, we chose 89 molecules to acquire from Enamine and LifeChemicals for experimental screening at Eurofins Discovery.

Our screening efforts yielded notable outcomes, with 38 of the selected molecules displaying inhibitory activity exceeding 50% at 10 μM and 17 (see Supporting Information for structures) showing greater than 85% inhibition at 10 μM for D_4_R (Figure 2B,C). Our success for identifying selective molecules was much lower. This is not unexpected as the selectivity metric is built from multiple independent predictions (eq S1), and thus, error from each prediction accumulates in the final score. Frequently, molecules predicted to be D_4_R selective were only selective against a single off-target subtype. Nonetheless, a subset of D_4_R-active compounds exhibited varying degrees of selectivity relative to at least one other dopamine receptor subtype (Figure 2B).

### Identification of a Spirocyclic Core for D_4_R Antagonists

From our initial screen, we identified compound *VU6052469*, which is structurally similar to a previously published D_4_R antagonist by Carato et al. bearing a piperidine core with a naphthamide substituent that exhibits high potency and selectivity for D_4_R over D_2_R;^71^ however, *VU6052469* itself is nonselective (Figure 2B,D). We docked *VU6052469* and the Carato compound into D_4_R (PDB ID: 6IQL)^68^ to investigate the potential binding mode of our hit (Figure 2E). One challenge with designing D_4_R antagonists is the topological pseudosymmetry of D_4_R-active compounds, which in the case of *VU6052469* and the Carato compound entails two distal aryl rings linked to a piperidine core (Figure 2D). In principle, this symmetry could enable the molecules to bind such that the halogen-substituted phenyl ring interacts with either transmembrane helices 2 (TM2) and TM3 (Figure S103A) or alternatively with TM4/5/6 (Figure S103B). In either binding pose, for example, *VU6052469* hydrogen bonds with the conserved D3.32 side chain, and V3.33 can stack with its aromatic rings (Figure S103). The pocket formed by TM2/3 is hydrophobic and has previously been implicated in ligand selectivity.^68,69^ Indeed, the TM2/3 interface differs between D_4_R and D_2_R in that D_2_R contains aromatic ring side chains, while in D_4_R, there are aliphatic chains (Figure S104). In contrast, the amino acid composition of TM4/5/6 is a mixture of polar and hydrophobic residues. Notably, a cluster of serine residues engaged in internal backbone hydrogen bonds in TM5/6 renders this portion of the pocket more sterically accessible.

We reasoned that the latter pose is less likely as it induces a greater loss of planarity of the amide linker within the docked pose, which is supported by density functional theory (DFT) conformational stability calculations and molecular orbital analysis performed at the wB97X-D/6-311G(d,p) level of theory^72^ (Figure S103C,D). We estimate that the first pose of *VU6052469* (Figure S103A) is 11.3 kcal/mol more energetically favorable, and it follows that the Carato compound adopts a similar binding conformation (Figure 2E). Despite being nonselective, our docked poses suggest that *VU6052469* could readily be made selective through extending the amide bond via a methylene linker and truncating the arene without altering the orientation of the ligand within the binding pocket. To that end, we replaced the secondary amide with an azetidine amide to give a 2,7-diazaspiro[3.5]nonane core, resulting in compound **4**, which displayed selectivity for D_4_R with only a partial loss of on-target activity (Table 1).

**Table 1:**
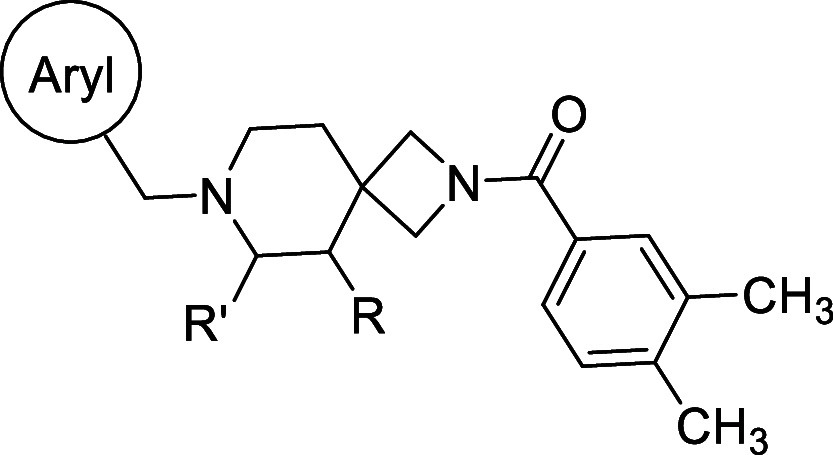

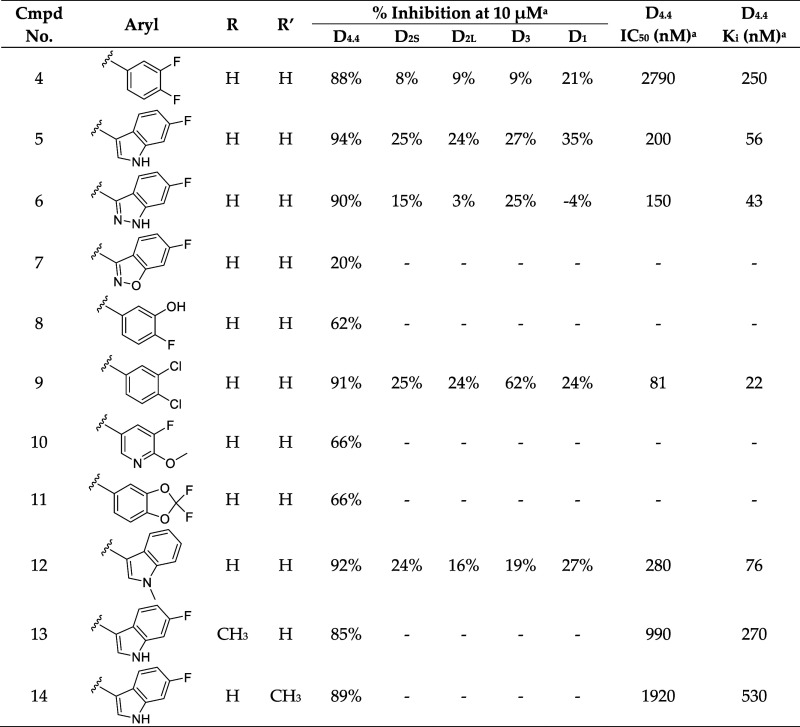
Southern Region SAR

a Values were obtained from Eurofins Discovery. See Supporting Information for more details.

**Figure 3:**
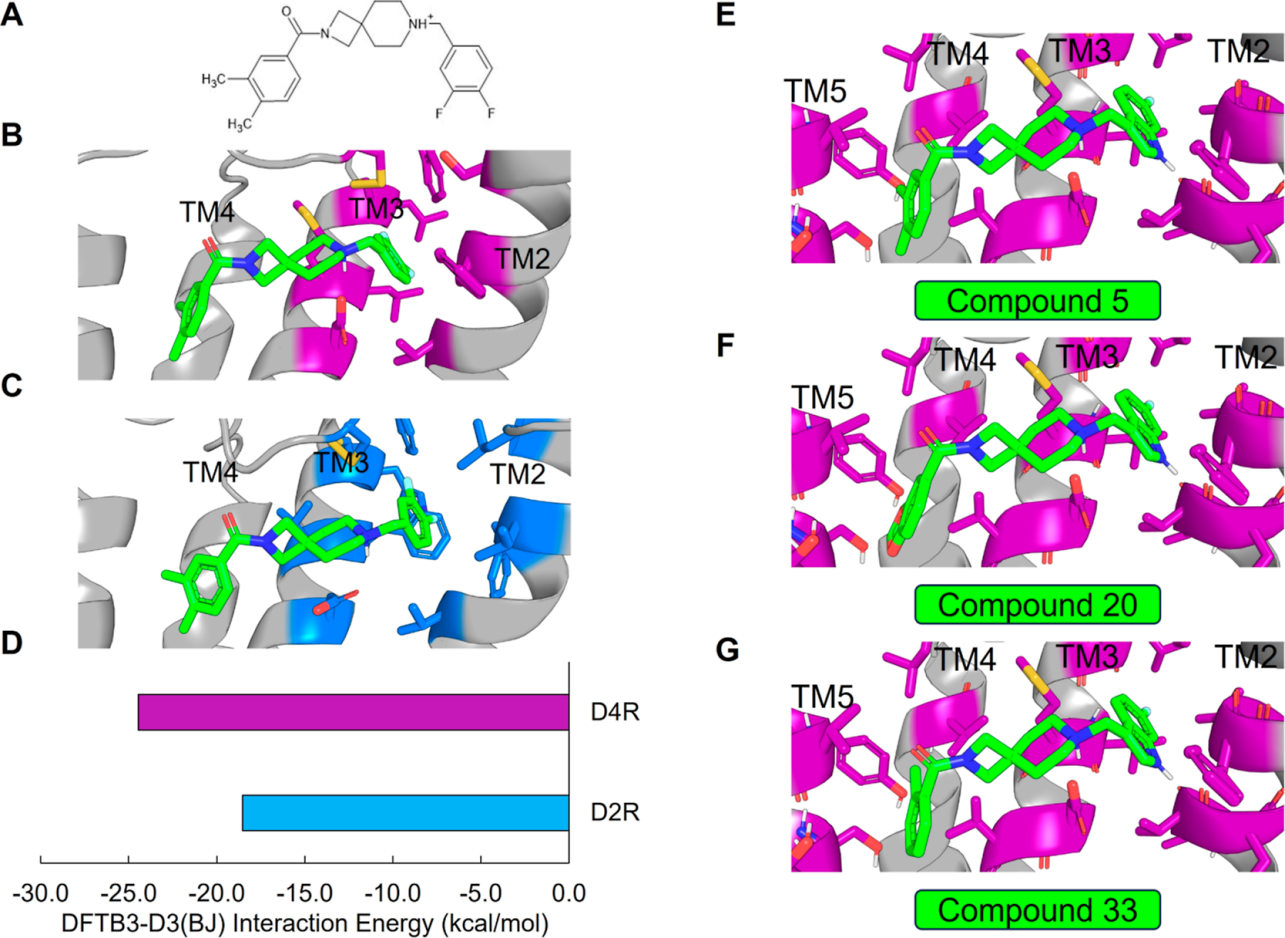
SAR analysis of D_4_R selective antagonists. (A) Chemical structure of the spirocyclic compound **4**. (B) Docked pose of compound **4** (green) in D_4_R. (C) Docked pose of compound **4** (green) in D_2_R. (D) DFTB3-D3(BJ) interaction energy (kcal/mol) between compound **4** and the central aspartate and TM2/TM3 hydrophobic pocket of D_4_R (purple) and D_2_R (blue). Docked poses of compounds (E) **5**, (F) **20**, and (G) **33**.

To better understand the mechanism of selectivity imparted by the spirocyclic core, we docked **4** into D_4_R and D_2_R (see Supporting Information) (Figure 3A–C). We verified the binding mode by running molecular dynamics (MD) simulations and analyzing ligand root-mean-square-deviation (rmsd) over time (Figure S105). Our docked poses suggest that the difluorophenyl of **4** differentially engages the TM2/3 hydrophobic pocket in D_4_R versus D_2_R. Compared to its complex with D_4_R, in the D_2_R complex **4** is shifted deeper into the TM2/3 pocket such that the hydrogen bond geometry between the orthosteric pocket aspartate D3.32 and the protonated piperidine is suboptimal (Figure S106). We confirmed that the D_2_R electrostatic interactions are less favorable than D_4_R by performing geometry optimization and subsequent interface energy calculations of the complexes using the semiempirical quantum mechanics (QM) tight-binding density functional theory (DFTB) method with dispersion corrections, DFTB3-D3(BJ) (Figure 3D) (see Supporting Information).^73,74^ The interaction energies of **4** with respect to the conserved central aspartate D3.32 and TM2/3 hydrophobic pocket in D_4_R and D_2_R are estimated to be −24.46 and −18.53 kcal/mol, respectively (Figure 3D).

### Optimization of Spirocyclic D_4_R Antagonist Potency and Selectivity

**Scheme 1:**
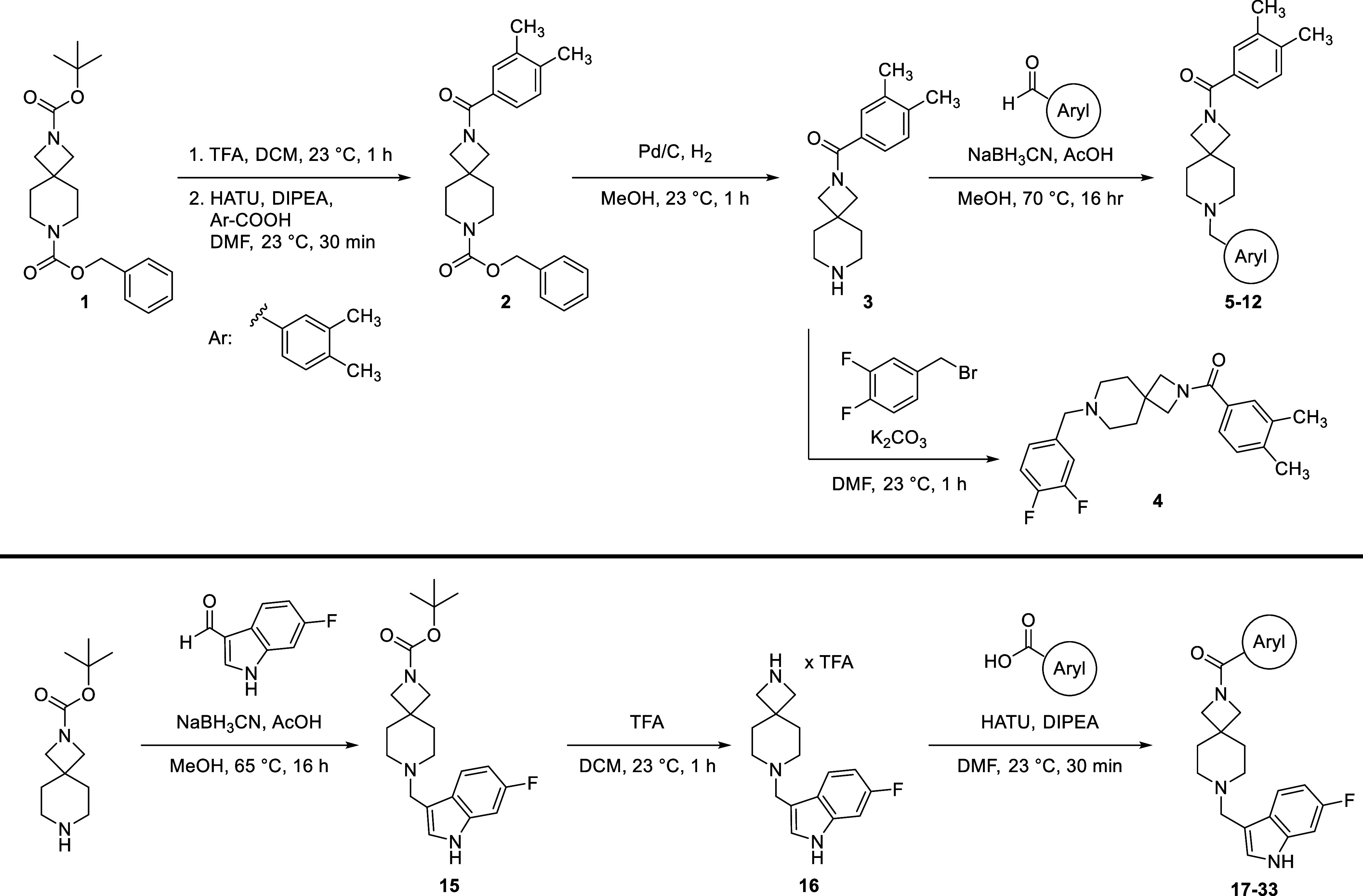
Synthesis of Diazaspiro[3.5]nonane D_4_R Antagonists

We sought to improve upon the potency and selectivity of **4** by screening analogues with differing polar aromatic or heteroaromatic groups on the southern end of the compound, installing methyl groups at the 2 or 3 position of the piperidine and probing the effect of the substitution pattern and substituent type on the northern phenyl ring on activity (Tables 1 and 2). The general synthetic scheme for this class of compounds is shown in Scheme 1, and detailed experimental procedures are provided in the Supporting Information for all intermediates and final compounds as well as compound **1**. Briefly, compound **1** underwent TFA-mediated *boc*-deprotection followed by HATU amide coupling to afford intermediate **2**. Subsequent benzyloxycarbonyl removal via hydrogen over palladium reduction gave key intermediate **3**, which was subjected to either reductive amination with assorted aryl aldehydes to afford compounds **5**–**12** or an S_N_2 reaction with 3,4-difluorobenzyl bromide to provide compound **4**. To obtain azetidine amides **17**–**33**, commercially available *tert*-butyl 2,7-diazaspiro[3.5]nonane-2-carboxylate was subjected to reductive amination with 6-fluoro-1*H*-indole-3-carbaldehyde to give intermediate **15**. *Boc*-deprotection with TFA afforded **16**, which then underwent HATU amide coupling with assorted aryl carboxylic acids to give compounds **17**–**34**.

**Table 2:**
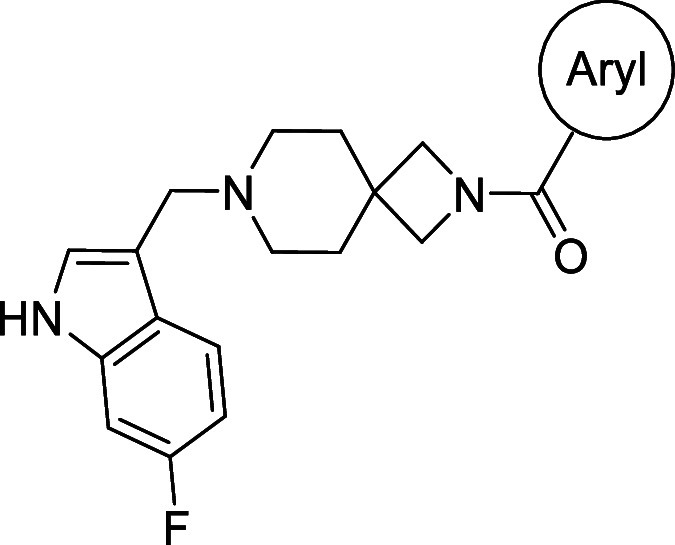

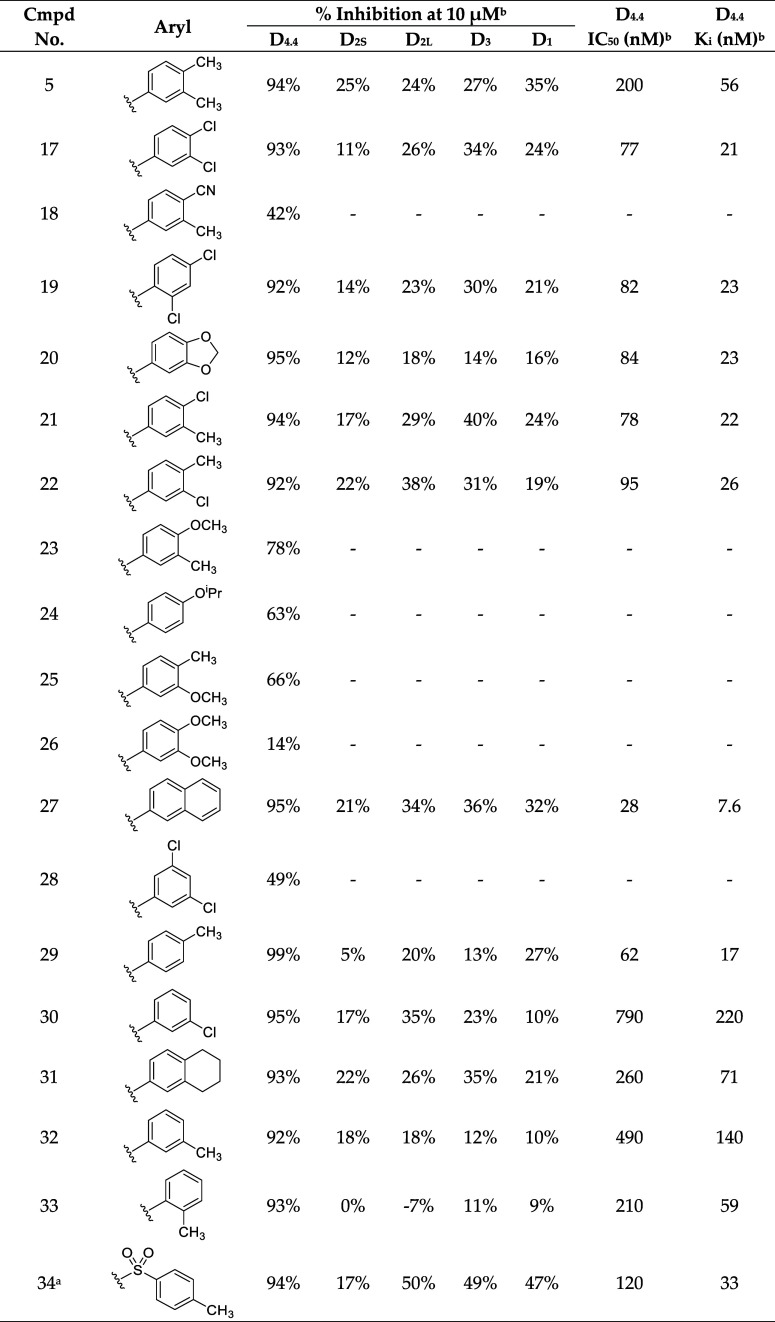
Northern Ring SAR

a Structure for this compound is a sulfonamide bound to the azetidine nitrogen of the spirocycle.

b Values were obtained from Eurofins Discovery. See Supporting Information for more details.

**Table 3:** In Vivo and In Vitro Results of Selected Compounds

compound	*f*_u,plasma_^a^		CL_H_^a^ (mL/min/kg)		CL_p_^a^ (mL/min/kg)	*t*_1/2_^a^ (h)	V_ss_^a^ (L/kg)	AUC^a^ (h·ng/mL)
	human	rat	human	rat				
4	0.01	0.03	16.9	59.1	116	1.05	5.52	28.7
33	0.19	0.26	16.0	39.0	123	4.02	36.9	27.0
32	0.06	0.14	14.7	46.2				
29	0.05	0.15	17.3	49.2				
20	0.10	0.23	13.7	36.2	126	4.55	44.4	26.4

a *f*_u_ = Fraction unbound; equilibrium dialysis assay; CL_H_ = hepatic clearance; CL_p_ = plasma clearance; *t*_1/2_ = terminal phase plasma half-life; V_*ss*_ = volume of distribution at steady-state; AUC = area under the curve.

Overall, this focused collection of spirocyclic antagonists provided a number of valuable SAR insights. With respect to the southern region, replacing the difluorophenyl moiety with the analogous dichlorophenyl substituent (**9**) resulted in significantly increased activity; however, a significant decrease in selectivity between the DR subtypes was also observed. Incorporation of other substituted arenes, such as fluorophenol (**8**), benzodioxole (**11**), and fluoropyridine (**10**), resulted in a steep decrease in inhibition (Table 1). By installing a 6-fluoroindole heterocycle (**5**) as we used previously in our morpholine core D_4_R antagonist (VU6004432, Figure 1),^58^ we observed drastically improved activity over **4**, though the overall selectivity was mildly decreased. Exchanging the indole for an indazole **6** resulted in an improvement in the selectivity against all subtypes, with a mild improvement in activity at D_4.4_R. This is in stark contrast to the incorporation of benzisoxazole (**7**), which essentially abolishes activity. Modifications to the spirocyclic core were not favorable as the addition of methyl groups to the 2 or 3 position of the piperidine ring (compounds **14** and **13**, respectively) significantly reduced the potency and affinity of the compound compared to that observed with **5** (Table 1), while expansion of the azetidine to a pyrrolidine led to a substantial decrease in inhibitory activity (compound **42**; see Supporting Information).

**Figure 4:**
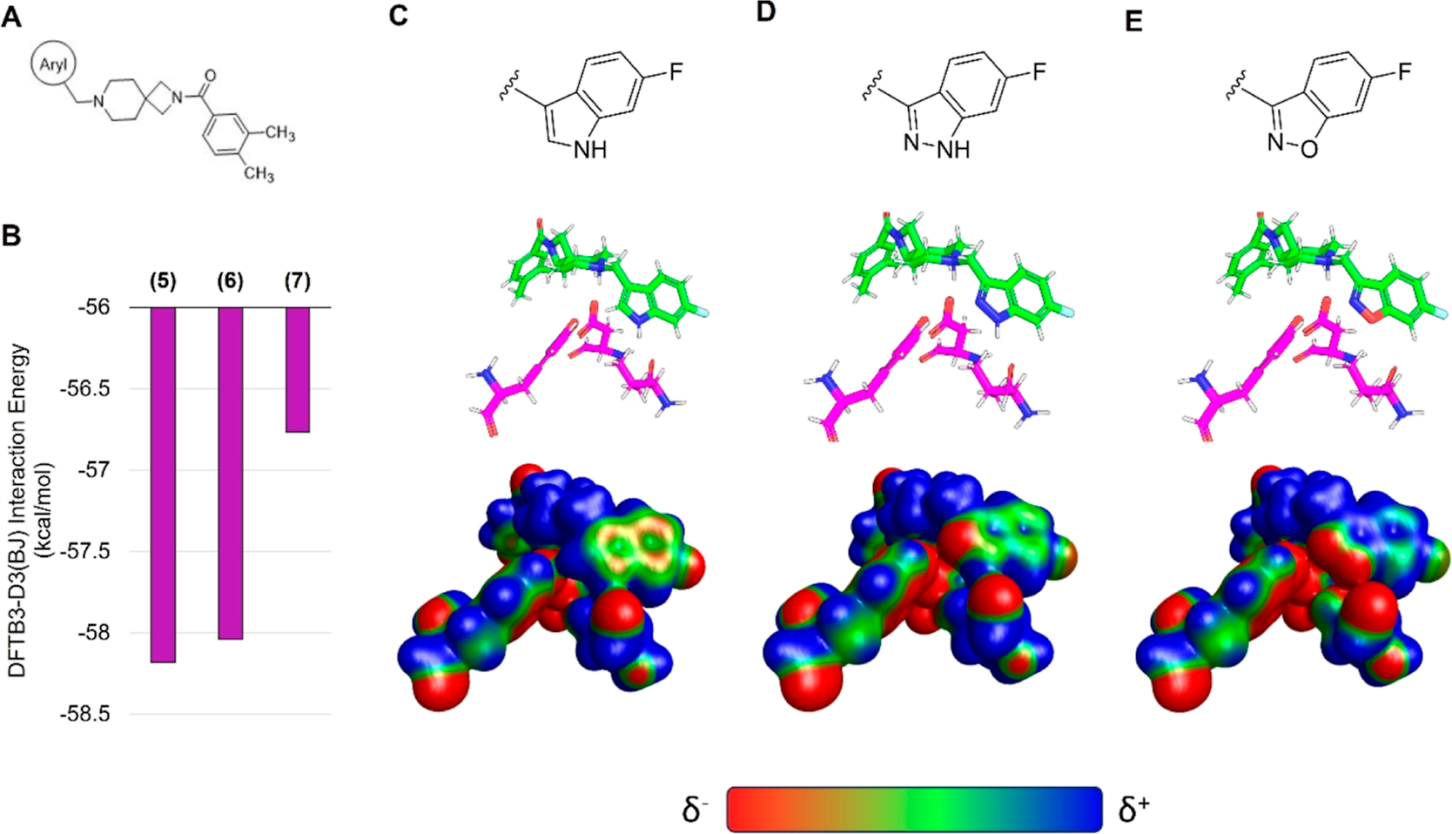
Surface electrostatics analysis of D_4_R selective antagonists in complex with D_4_R. (A) Schematic southern aryl substitution on compound **4**. (B) Interaction energies for the model systems containing compounds **5**, **6**, or **7**. Surface electrostatic potential analysis of (C) compound **5**, (D) compound **6**, and (E) compound **7**. Electrostatic potentials are calculated for model systems (C–D) at the wB97X-D/6-31G(d) level of theory with solvation model density (SMD) aqueous implicit solvent following geometry optimization of the receptor pocket and ligand in complex utilizing DFTB3-D3(BJ) with SMD solvent water.

To better understand the differences in activity between **5**, **6**, and **7**, we first docked **5** to D_4_R. Once again, pseudosymmetry within **5** rendered two flipped binding modes plausible. The first binding mode (Figure 3E) follows from the predicted poses of *VU6052469* and **4**. Interestingly, however, an alternative binding mode in which the indole ring of **5** adopts a pose mimicking the experimentally determined bound pose of L745,870^68^ is also possible. To determine which pose is more likely, we performed MD simulations starting from each docked pose. We observed that the pose consistent with **4** (Figure 3E) is more likely to remain near the docked binding pose (Figure S107A,B) and adopt favorable hydrogen bond geometry with D3.32 (Figure S107C,D). Furthermore, the interaction energy rankings for this binding mode (Figure 3E) are consistent with the experimental results and demonstrate the activity cliff in **7** (Table 1 and Figure 4A,B). In contrast, the binding mode mimicking the L745,870 pose yields interaction energy estimates inconsistent with experiment (data not shown). Visualization of the surface electrostatic potentials of D_4_R complexed with **5**, **6**, or **7** at the DFT wB97X-D/6-31G(d) level of theory^72^ suggests that this activity cliff is due to loss of complementary electrostatic interactions and an abundance of anionic charge near TM2 (Figure 4C–E).

While indazole antagonist **6** provided the best potency and selectivity profile thus far, we proceeded with the combination of the 6-fluoroindole southern ring and the unmodified 2,7-diazaspiro[3.5]nonane core for exploration of the northern region SAR as **5** performed similarly and was more cost-effective for library synthesis. Therefore, we employed **5** as a starting point for pursuing a focused library of aryl amides on the northern end of the scaffold for further improvement of DR subtype selectivity (Table 2). Overall, alkyl and chloro substituents were well-tolerated, with the sole exception of the 3,5-dichlorophenyl analogue (**28**), which demonstrated drastically reduced inhibitory activity (49%). The 2,4-dichlorophenyl regioisomer (**19**) retained activity, however, indicating that D_4.4_R inhibition is sensitive to subtle changes in substitution pattern in this region. In contrast to alkyl and chloro groups, incorporation of alkoxy groups generally led to a significant reduction in activity against D_4.4_R (**23**–**26**), with the sole exception being **20** (Figure 3F), which bears a benzodioxole heterocycle (D_4.4_R IC_50_ = 84 nM; *K*_i_ = 23 nM). The potency of benzodioxole-bearing compound **20** suggests that the lack of activity observed in compounds **23**–**26** is a result of unfavorable steric interactions facilitated by their freely rotating alkyl groups rather than ring electronics. In addition to the benzodioxole example (**20**), increasing the size of the aryl amide from a monocycle to a fused bicycle in other instances was also well tolerated (**27**, **31**), with naphthalene **27** exhibiting particularly potent activity (D_4.4_R IC_50_ = 28 nM; *K*_i_ = 7.6 nM). With respect to selectivity, a strong sensitivity to regioisomerism was observed, which was most clearly demonstrated in compounds **29**, **32**, and **33**, which bear *para*-, *meta*-, and *ortho*-toluamides, respectively. Of these, compound **29** demonstrates the highest D_4.4_R activity (D_4.4_R IC_50_ = 62 nM; *K*_i_ = 17 nM), and it exhibits a moderately improved selectivity profile over **5**. Both *meta* and *ortho* isomers (compounds **32** and **33**, respectively) display reduced activity compared to *para* isomer **29**. Compound **33** (Figure 3G), however, exhibited the best selectivity profile of all compounds disclosed herein, with a notable 0% activity against D_2S_. It was also observed that replacement of the *para*-toluamide of **29** with a tosylamide (**34**) mildly reduced the D_4.4_R activity but notably increased the inhibitory activity at all other tested DR subtypes, possibly due to the reduced planarity of the sulfonamide.

### In Vitro and In Vivo DMPK Analysis of Selected Compounds

A subset of compounds that demonstrated high potency and excellent selectivity were selected for pharmacokinetic characterization (Table 3). In vitro stability experiments in rat and human microsomes returned high clearance (>70% *Q*_h_) across all compounds, except for **20**, which exhibited moderate hepatic clearance (CL_*H*_ of 13.7 and 36.2 in human and rat microsomes, respectively). The free fraction in plasma ranged from 1 to 19% in rat and 3–26% in human. Notably, both compounds **33** and **20** exhibited increased free fractions compared to the original hit (**4**). Three compounds (**4**, **20**, and **33**) were selected to assess in vivo pharmacokinetics (compounds **29** and **32** were excluded as they exhibited worse free fraction in plasma compared to **20** and **33**). Upon intravenous dosing in rats, all three compounds demonstrated superhepatic clearance (>100% *Q*_h_). This result is consistent with these compounds experiencing high hepatic metabolic clearance and may also indicate contribution to clearance through a different route, such as extrahepatic metabolism or active direct excretion. Despite high clearance, compounds **4**, **20**, and **33** exhibited moderate to high distribution into tissues (volume of distributions of 5.52, 44.4, and 36.9 L/kg, respectively), explaining the reasonable half-lives for these compounds (1.05, 4.55, and 4.02 h, respectively).

**Figure 5:**
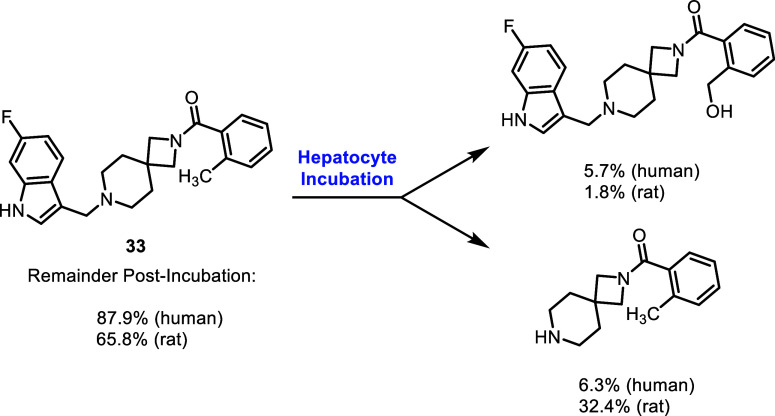
Metabolite analysis of compound **33** in human and rat hepatocytes. Parent compound incubated in human or rat hepatocytes for 4 h. Percentages (determined via LC/MS) indicate the relative percentage of compounds present postincubation. See Supporting Information for details.

Compound **33** was subjected to metabolite profiling in human and rat hepatocytes to provide insight into potential clearance mechanisms and metabolic liabilities (Figure 5). After incubation for 4 h, **33** exhibited low turnover in human hepatocytes and moderate turnover in rat hepatocytes, with 87.9 and 65.8% of parent compound (**33**) remaining postincubation, respectively. In both species, only two major metabolites were observed: mono-oxidation of the benzylic methyl group and piperidine *N*-dealkylation. The latter means of metabolism was elevated in rats (32.4%) compared to that in humans (6.3%).

## Discussion

The application of spirocycles to drug discovery efforts has increased in recent years as a means to increase compound three-dimensionality, modulate DMPK properties, incorporate additional sp^3^ centers, and generate novel intellectual property.^75−78^ One of the central findings of the present study was the discovery of 2,7-diazaspiro[3.5]nonane as an applicable core motif for selective D_4_R antagonists. While we initially identified the highly potent antagonist *VU6052469*, which exhibited a high degree of structural similarity to a previously reported selective D_4_R antagonist,^71^ it notably lacked selectivity (Figure 2B,D,E). We postulated that this lack of selectivity arose from the difference in length between these two compounds, with the naphthalene and 4-chlorobenzyl moieties of the Carato compound potentially leading to poorer steric interactions within the TM2/3 pocket of D_2_R than the dimethylphenyl and 3,4-difluorobenzyl moieties of *VU6052469* (Figure 2E). By replacing the core piperidine of *VU6052469* with 2,7-diazaspiro[3.5]nonane, the dimethylphenyl ring is extended further into the TM4/5/6 pocket, affording potent and selective activity against D_4_R (Table 1). While there have been reported examples of substituted diazaspirocycles bearing D_4_R activity, this activity was not the desired mode of action (i.e., the intent was to target σ receptors) nor did the more potent compounds exhibit DR subtype selectivity.^79^ Therefore, to the best of our knowledge, this is the first report of the use of diazaspirocycles in pursuit of selective D_4_R antagonists.

Interestingly, our investigation revealed an activity cliff when comparing the indole/indazole- vs benzisoxazole-substituted compounds (compounds **5/6** and **7**, respectively). Activity cliffs are subtle structural changes leading to significant alterations in inhibitory activity. In this case, the subtle difference in ligand interaction energies with the receptor went undetected by the docking score function. It was only after performing geometry optimization and interaction energy calculations with the more computationally demanding semiempirical QM method DFTB3-D3(BJ) that we understood the case of the reduction in binding affinity, which was a result of an accumulation of anionic charge near TM2 with no available hydrogen bond donors. This example emphasizes the continued importance of developing force fields and/or deep learning algorithms for binding affinity prediction that can be used during rapid screening protocols.

A key challenge in the rational design of selective D_4_R antagonists is the topological pseudosymmetry displayed by most antagonists. This challenge is 2-fold: (1) highly similar antagonists may be oriented in conformations 180° opposed to one another, and (2) the internal pseudosymmetry of many D_4_R antagonists renders it difficult to ascertain their appropriate binding modes. Despite extensive computational validation, it is possible that our putative binding modes are inaccurate, which may lead to false structure–activity relationships. Further experimental structural evidence, such as crystal structures of these spirocyclic antagonists bound to D_4_R, will be valuable in the design of future D_4_R antagonists with similar potencies and selectivity.

Modifications to the northern aryl amide of this scaffold demonstrated the sensitivity of D_4_R potency and selectivity to ring substituent choice and regioisomerism. Overall, compound **33**, which bears an *ortho*-toluamide northern substituent, displayed the best selectivity profile of the tested compounds while retaining potent D_4.4_R antagonism and affinity (IC_50_ = 210 nM; *K*_i_ = 59 nM). Though our study has yielded promising D_4_R antagonists such as this, an ongoing challenge in the design of this class of compounds is the optimization of pharmacokinetic properties. While this class of compounds exhibited excellent aqueous solubility (see Supporting Information), both in vitro and in vivo pharmacokinetic analysis of selected compounds demonstrated a key limitation of the present class: high metabolic clearance. The findings of these assays underscore the need for continued efforts to improve the pharmacokinetic profiles of potential D_4_R antagonist drug candidates, most likely via design changes to remove metabolic hotspots within this chemical series.

Altogether, our study has unveiled a spirocyclic core for D_4_R selective antagonists, providing a foundation for further drug development efforts in the context of PD. Our insight into DR subtype selectivity and activity cliffs offers valuable guidance for future research in this area. The improvement of spirocyclic D_4_R antagonist DMPK properties, however, remains requisite for the development of a suitable preclinical lead within this class as a potential adjuvant therapy for PD.

## Supporting Information Available

Experimental procedure for the synthesis of compounds, ^1^H and ^13^C{^1^H} NMR spectra for all compounds, 2D NMR (NOESY, HMBC, HSQC, and COSY) for compounds that required further characterization, DMPK experimental methods, computational methods, and metabolite profiling (PDF)
