# Supplementary material for: Computer-Aided Design and Biological Evaluation of Diazaspirocyclic D4R Antagonists
Source: ACS Chem Neurosci. 2024 Jun 7;15(12):2396–407. doi: 10.1021/acschemneuro.4c00086 (PMC11191600; doi:10.1021/acschemneuro.4c00086)
Supplement: Supplementary file 1 — cn4c00086_si_001.pdf [file cn4c00086_si_001.pdf]

Supporting Information

Computer-Aided Design and Biological Evaluation of Diazaspirocyclic D_4R Antagonists

Caleb A. H. Jones[1,2,3*,#], Benjamin P. Brown[3,4,5*], Daniel C. Schultz[1,2,3*], Julie Engers[1,2,3], Valerie M. Kramlinger[1,2,3], Jens Meiler[3,4,5,6,#], Craig W. Lindsley[1,2,3,#]

[1]Warren Center for Neuroscience Drug Discovery, Vanderbilt University School of Medicine, Nashville, TN, 37232, USA

[2]Department of Pharmacology, Vanderbilt University School of Medicine, Nashville, TN, 37232, USA.

[3]Department of Chemistry, Vanderbilt University, Nashville, TN, USA. Nashville, TN 37232, USA.

[4]Center for Structural Biology, Vanderbilt University, Nashville, TN, USA. Nashville, TN 37232, USA.

[5]Center for Applied AI in Protein Dynamics, Vanderbilt University, Nashville, TN, USA. Nashville,
TN 37232, USA.

[6]Institute for Drug Discovery, Leipzig University Medical School, Leipzig, SAC 04103, Germany.

*Equally contributing

# Correspondence:
Craig W. Lindsley, PhD
Warren Center for Neuroscience Drug Discovery
Vanderbilt University School of Medicine
Departments of Chemistry and Pharmacology
Vanderbilt University
Nashville, TN 37232-8725 USA
E-mail: craig.lindsley@vanderbilt.edu

Jens Meiler, PhD
Department of Chemistry
Vanderbilt University
Nashville, TN 37232-8725 USA
E-mail: jens.meiler@vanderbilt.edu

Caleb A. H. Jones, PhD
Warren Center for Neuroscience Drug Discovery
Vanderbilt University School of Medicine

Departments of Chemistry and Pharmacology
Vanderbilt University
Nashville, TN 37232-8725 USA
E-mail: caleb.a.jones@vanderbilt.edu

Keywords: Dopamine receptors, D_4R antagonism, Parkinson's disease

Contents

I. General Information S4
i. General Considerations. S4
ii. Mass Spectrometry. S4
II. Experimental Procedures S5
III. NMR Spectra S32
IV. DMPK Methods S134
i. Human Dopamine GPCR Binding Antagonist Assay S134
ii. Materials S135
iii. Microsomal Stability S136
iv. Plasma Protein Binding S136
v. Animal Care and Housing S137
vi. *In vivo* DMPK experimental S137
vii. Liquid Chromatography-Mass Spectrometry Analysis S137
viii. Kinetic Solubility S138
ix. References S139
V. Computational Methods S140
i. Virtual Screening S140
ii. Property-based Small Molecule Flexible Alignment S141
iii. Protein-ligand Docking and Interaction Energy Analysis S141
iv. Molecular Dynamics Simulations S142
v. Surface Electrostatic Potential and Molecular Orbital Analysis S143
vi. References S144
vii. Supplemental Figures S146
viii. Structures of Validated HTS Hits S151
VI. Metabolite Profiling S153

I. General Information

i. General Considerations.

Syntheses and manipulations were conducted in air unless otherwise specified. Reaction solvents were purchased from Sigma-Aldrich in anhydrous form with a sure seal. All reagents and building blocks for which procedures are not given below were procured from commercial vendors and used without further purification. ^{1}H, $^{13}C\{^{1}H\}$ and 2D NMR spectra were recorded on a 400 MHz Bruker AV-400 spectrometer at ambient temperature unless otherwise noted. ^{1}H and $^{13}C\{^{1}H\}$ chemical shifts are referenced to residual solvent signals in the following solvents: $CDCl_3$ (^{1}H NMR: 7.26 ppm; $^{13}C\{^{1}H\}$ NMR: 77.16 ppm), DMSO-d_6 (^{1}H NMR: 2.50 ppm, $^{13}C\{^{1}H\}$ NMR: 39.52 ppm), and MeOD-d_4 (^{1}H NMR: 3.31 ppm, $^{13}C\{^{1}H\}$ NMR: 49.00 ppm). Chemical shifts are reported in ppm and multiplicities are abbreviated as follows: br = broad, s = singlet, d = doublet, t = triplet, q = quartet, quint = quintet, dd = doublet of doublets, dt = doublet of triplets, td = triplet of doublets, ddd = doublet of doublet of doublets, tdd = triplet of doublet of doublets, m = multiplet. Automated flash column chromatography (normal phase) was conducted using a Teledyne ISCO CombiFlash system with certified ACS grade solvents. Reverse phase HPLC was performed on a Gilson preparative reverse-phase HPLC system comprised of a 333 aqueous pump with solvent-selection valve, 334 organic pump, GX-271 or GX-281 liquid hander, two column switching valves, and a 155 UV detector. UV wavelength for fraction collection was user-defined, with absorbance at 254 nm always monitored. Column: Phenomenex Axia-packed Gemini C18, 30 x 50 mm, 5 µm. Mobile phase: CH_3CN in H_2O (0.05% v/v NH_4OH). Gradient conditions: 0.75 min equilibration, followed by user-defined gradient (starting organic percentage, ending organic percentage, duration), hold at 95% CH_3CN in H_2O (0.05% v/v NH_4OH) for 1 min, 50 mL/min, 23 °C.

ii. Mass Spectrometry.

High-resolution mass spectra were obtained on an Agilent 6540 UHD Q-TOF with Dual AJS source. MS parameters were as follows: fragmentor: 150; capillary voltage: 4000 V; nebulizer pressure: 60 psi; drying gas flow: 13 L/min; drying gas temperature: 275 °C. Samples were introduced via an Agilent 1290 UHPLC comprised of a G4220A binary pump, G4226A ALS, G1316C TCC, and G4212A DAD with ULD flow cell. UV absorption was observed at 215 nm and 254 nm with a 4 nm bandwidth. Column: Waters Acquity BEH C18, 1.0 x 50 mm, 1.7 um. Gradient conditions: 5% to 95% CH_3CN in H_2O (0.1% Formic Acid) over 1.25 min, hold at 95% CH_3CN for 0.25 min, 0.3 mL/min, 40 °C.

II. Experimental Procedures

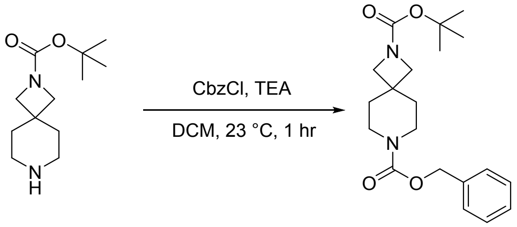

7-benzyl 2-(*tert*-butyl) 2,7-diazaspiro[3.5]nonane-2,7-dicarboxylate (1):

In a vial, *tert*-butyl 2,7-diazaspiro[3.5]nonane-2-carboxylate (200 mg, 0.88 mmol) was dissolved in dichloromethane (4 mL), followed by the addition of triethylamine (370 µL, 2.65 mmol) and the dropwise addition of benzyl chloroformate (140 µL, 0.97 mmol). The reaction was then stirred at room temperature for one hour at which point LCMS indicated full conversion. The reaction mixture was then quenched with saturated sodium bicarbonate and extracted with dichloromethane (x3). Organics were passed through a phase separator and made concentrated. Crude product was purified using a Teledyne ISCO Combi-Flash system (liquid loading, 40G column, 0-70% EtOAc/Hex) to give **(1)** (255 mg, 0.71 mmol, 80% yield) as a clear oil.

^{1}H NMR (400 MHz, $CDCl_3$): δ 7.31-7.37 (m, 5H), 5.12 (s, 2H), 3.64 (s, 4H), 3.43 (m, 4H), 1.71 (m, 4H), 1.44 (s, 9H).

^{13}C{^{1}H} NMR (101 MHz, $CDCl_3$): δ 156.5, 155.4, 136.8, 128.6, 128.2, 128.0, 79.7, 67.3, 58.8, 41.1, 35.2, 33.6, 28.5.

HRMS (ESI) calculated for formula $C_{20}H_{29}N_2O_4$ ($[M+H]^+$) 361.2122, found 361.2127.

Note: ^{1}H spectrum aligns with published data.[1] No ^{13}C{^{1}H} spectra reported.

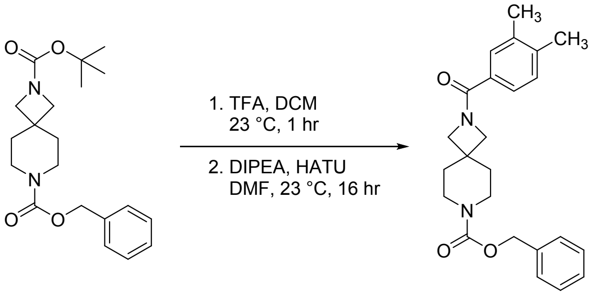

Benzyl 2-(3,4-dimethylbenzoyl)-2,7-diazaspiro[3.5]nonane-7-carboxylate (2):

In a vial, **(1)** (254.7 mg, 0.71 mmol) was dissolved in dichloromethane (2 mL) and cooled to 0 °C, followed by the addition of trifluoroacetic acid (1 mL, 13.06 mmol). The solution was then warmed to room temperature and stirred for one hour. At this point, LCMS indicated full conversion, and the reaction was concentrated, quenched with saturated sodium bicarbonate, and extracted with dichloromethane (x3). Organics were passed through a phase separator, concentrated, and dissolved in *N*,*N*-dimethylformamide (1 mL). In a separate vial, 3,4-dimethylbenzoic acid (159.2 mg, 1.06 mmol) and HATU (403.0 mg, 1.06 mmol) were dissolved

in *N,N*-dimethylformamide (1 mL) before the addition of *N,N*-diisopropylethylamine (0.74 mL, 4.24 mmol). The solution was then stirred for 10 minutes before the addition of the intermediate produced above and stirring at room temperature for 30 minutes. At this point, LCMS indicated some starting material remained, and additional *N,N*-diisopropylethylamine (0.74 mL, 4.24 mmol) was added before stirring overnight. The following morning, the mixture was diluted in water and extracted with ethyl acetate. The organic layer was then washed with brine, dried over sodium sulfate, filtered, and made concentrated. Crude product was purified using a Teledyne ISCO Combi-Flash system (liquid loading, 40G column, 0-10% MeOH/DCM; elution ~ 5%) to give **(2)** (266.8 mg,0.68 mmol, 96% yield) as a colorless oil.

^{1}H NMR (400 MHz, $CDCl_3$): δ 7.44 – 7.44 (m, 1H), 7.38 – 7.29 (m, 5H), 7.15 (d, *J* = 7.7 Hz, 1H), 5.12 (s, 2H), 3.99 – 3.92 (m, 4H), 3.45 (q, *J* = 5.1 Hz, 4H), 2.29 (s, 6H), 1.76 (br. s, 4H).

^{13}C{^{1}H} NMR (101 MHz, $CDCl_3$): δ 155.4, 137.1, 136.8, 129.7, 129.6, 129.6 129.4, 128.7, 128.2, 128.1, 125.5, 125.5, 67.4, 41.1, 35.2, 34.4, 34.4, 20.0, 19.9.

HRMS (ESI) calculated for formula $C_{24}H_{29}N_2O_3$ ([M+H]$^+$) 393.2173, found 393.2175.

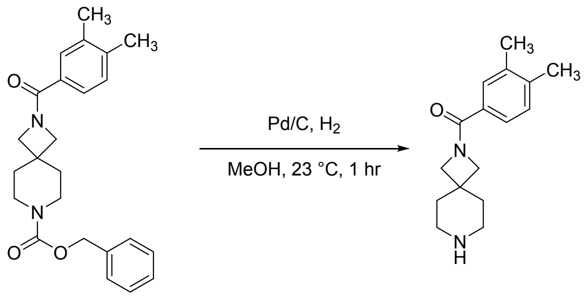

(3,4-dimethylphenyl)(2,7-diazaspiro[3.5]nonan-2-yl)methanone (3):

To a solution of **(2)** (266.8 mg, 0.68 mmol) in methanol (5 mL) was added palladium on activated carbon (72.3 mg, 0.07 mmol, 10 % wt). The vessel was then sealed, and the ambient atmosphere was displaced with nitrogen and then hydrogen. The reaction was then stirred at room temperature for one hour at which point LCMS indicated full conversion. The hydrogen atmosphere was then displaced with nitrogen before opening the vessel and filtering the reaction through a pad of Celite and concentrating under vacuum. **(3)** (169.1 mg, 0.65 mmol, 96% yield) was obtained as beige oil and was used in the next step without further purification.

^{1}H NMR (400 MHz, MeOD-*d*$_4$): δ 7.44 – 7.43 (m, 1H), 7.37 (dd, *J* = 7.8, 1.9 Hz, 1H), 7.23 – 7.21 (m, 1H), 4.06 (s, 2H), 3.86 (s, 2H), 3.36 (s, 1H), 2.82 – 2.74 (m, 4H), 2.31 (s, 6H), 1.77 (t, *J* = 5.6 Hz, 4H).

^{13}C{^{1}H} NMR (101 MHz, MeOD-*d*$_4$): δ 172.6, 141.7, 138.1, 131.6, 130.7, 129.9, 126.5, 64.8, 60.1, 43.7, 36.4, 35.3, 19.8, 19.7.

HRMS (ESI) calculated for formula $C_{16}H_{23}N_2O$ ([M+H]$^+$) 259.1805, found 259.1811.

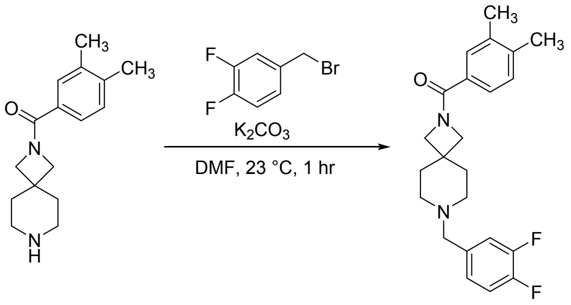

[7-[(3,4-difluorophenyl)methyl]-2,7-diazaspiro[3.5]nonan-2-yl]-(3,4-dimethylphenyl)methanone (4):

To a suspension of **(3)** (21.9 mg, 0.08 mmol) and potassium carbonate (23.8 mg, 0.17 mmol) in *N*,*N*-dimethylformamide (1 mL) was added 3,4-difluorobenzyl bromide (11.9 µL, 0.09 mmol). The reaction mixture was then stirred at room temperature for one hour. At this point, the reaction mixture was then filtered through a PTFE filter and purified using the Gilson (Basic, 30x 100 mm column, 5-95% ACN/ 0.05% aqueous NH_4OH, 10 min run). Fractions containing the desired product were concentrated and purified once more via Teledyne ISCO Combi-Flash system (liquid loading, 4G column, 0-10% MeOH/DCM) to give **(4)** (14.3 mg, 0.037 mmol, 44% yield) as a white solid.

^{1}H NMR (400 MHz, DMSO-d_6): δ 7.41 – 7.30 (m, 4H), 7.20 – 7.14 (m, 2H), 3.97 (br. s, 2H), 3.70 (br. s, 2H), 3.41 (br. s, 2H), 2.33-2.25 (m, 10H), 1.71 (br. s, 4H).

^{13}C{^{1}H} NMR (101 MHz, DMSO-d_6): δ 169.6, 151.0 (d, J_{C-F} = 244.6 Hz), 149.3 (d, J_{C-F} = 244.6 Hz), 139.9, 137.0 (br. s) 136.8, 131.2, 129.8, 129.2, 125.8, 117.7 (br. s), 63.1, 61.2, 58.6, 50.2, 35.3, 33.9, 19.8, 19.8.

Note: Broadening is observed in the ^{13}C{^{1}H} spectrum for the peaks correlating to the difluorobenzyl due to splitting from the fluorine nuclei, specifically at 117.7 ppm and 137.0 ppm. Some broadening is also observed at 125.8 ppm, which 2D NMR indicates is due to a buried carbon peak.

HRMS (ESI) calculated for formula $C_{23}H_{27}F_2N_2O$ ($[M+H]^+$) 385.2086, found 385.2092.

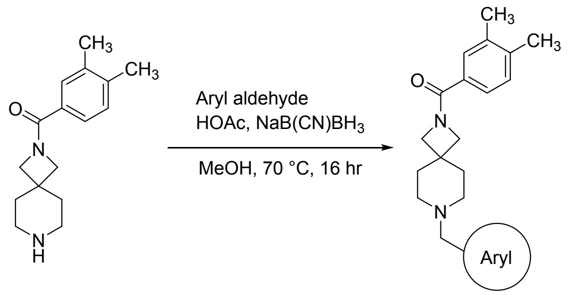

General Procedure A:

To a vial containing **(3)** (15.4 mg, 0.06 mmol, 1.0 equiv.) and an aryl aldehyde 0.07 mmol, 1.2 equiv.) in methanol (0.5 mL) was added acetic acid (0.17 mmol, 2.5 equiv.) and sodium cyanoborohydride (0.12 mmol, 2 equiv.). The vial was then sealed, heated to 70 ˚C, and stirred

overnight. The following morning, the reaction was made concentrated before quenching with saturated sodium bicarbonate and extracting with dichloromethane (x3). Organics were passed through a phase separator and made concentrated. Crude product was then purified either using reverse phase HPLC (Gilson) or normal phase silica gel chromatography (ISCO).

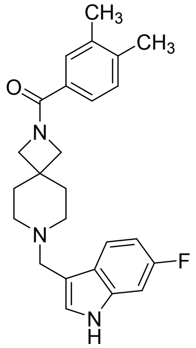

(3,4-dimethylphenyl)(7-((6-fluoro-1*H*-indol-3-yl)methyl)-2,7-diazaspiro[3.5]nonan-2-yl)methanone (5):

The compound was synthesized by following **General Procedure A.** Crude product was dissolved in DMSO (1 mL) and purified using the Gilson (Basic, 30x 100 mm column, 15-75% ACN/ 0.05% aqueous NH_4OH, 10 min run). Fraction containing the desired product were concentrated and purified once more via Teledyne ISCO Combi-Flash system (liquid loading, 4G column, 0-10% MeOH/DCM) to give **(5)** (5.5 mg, 0.01 mmol, 23% yield) as a white solid.

^{1}H NMR (400 MHz, $CDCl_3$): δ 10.96 (s, 1H), 7.60 (dd, *J* = 8.7, 5.6 Hz, 1H), 7.40 (d, *J* = 1.5 Hz, 1H), 7.33 (dd, *J* = 7.8, 1.7 Hz, 1H), 7.19-7.17 (m, 2H), 7.10 (dd, *J* = 10.2, 2.3 Hz, 1H), 6.85-6.79 (m, 1H), 3.93 (s, 2H), 3.68 (s, 2H), 3.55 (s, 2H) , 2.40-2.24 (m, 10H), 1.67 (s, 4H). ,

^{13}C NMR{^{1}H} (101 MHz, DMSO-d_6): δ 169.1, 158.8 (d, $J_{C\text{-}F}$ = 233.7 Hz), 139.4, 136.3, 136.1 (d, $J_{C\text{-}F}$ = 12.7 Hz), 130.8, 129.3, 128.8, 125.3, 125.0, 124.4, 120.1 (d, $J_{C\text{-}F}$ = 10.1 Hz), 111.4, 106.8 (d, $J_{C\text{-}F}$ = 24.4 Hz), 97.2 (d, $J_{C\text{-}F}$ = 25.4 Hz), 62.7, 58.3, 53.3, 49.8, 35.1, 33.7, 19.4, 19.3.

HRMS (ESI) calculated for formula $C_{25}H_{29}FN_3O$ ([M+H]$^+$) 406.2289, found 406.2289.

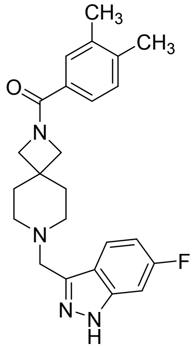

(3,4-dimethylphenyl)(7-((6-fluoro-1*H*-indazol-3-yl)methyl)-2,7-diazaspiro[3.5]nonan-2-yl)methanone (6):

The compound was synthesized by following **General Procedure A.** Crude product was dissolved in DMSO (1 mL) and purified using the Gilson (Basic, 30x 100 mm column, 15-75% ACN/ 0.05% aqueous NH_4OH, 10 min run). Fractions containing the desired product were concentrated to give **(6)** (12.5 mg, 0.03 mmol, 52% yield) as a white solid.

^{1}H NMR (400 MHz, DMSO-d_6): δ 7.88 (dd, J = 8.8, 5.4 Hz, 1H), 7.41 – 7.40 (m, 1H), 7.34 (dd, J = 7.8, 2.0 Hz, 1H), 7.25 – 7.17 (m, 1H), 7.18 (d, J = 7.8 Hz, 1H), 6.95 (ddd, J = 9.5, 8.8, 2.2 Hz, 1H), 3.95 (s, 2H), 3.76 (s, 2H), 3.69 (s, 2H), 2.24-2.40 (m, 10H), 1.69 (t, J = 5.6 Hz, 4H). Note: Indazole *NH* does not appear in this solvent.

^{13}C{^{1}H} NMR (101 MHz, DMSO-d_6): δ 169.1, 161.3 (d, $J_{C\text{-}F}$ = 241.0 Hz), 142.7, 141.0 (d, $J_{C\text{-}F}$ = 13.0 Hz), 139.4, 136.3, 130.8, 129.3, 128.8, 125.3, 122.5 (d, $J_{C\text{-}F}$ = 11.1 Hz), 119.2, 109.3 (d, $J_{C\text{-}F}$ = 26.0 Hz), 95.4 (d, $J_{C\text{-}F}$ = 25.9 Hz), 62.7, 58.2, 54.6, 50.0, 35.0, 33.4, 19.3, 19.3.

HRMS (ESI) calculated for formula $C_{24}H_{28}FN_4O$ ($[M+H]^+$) 407.2240, found 407.2242.

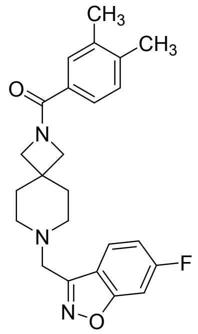

(3,4-dimethylphenyl)(7-((6-fluoro-1,2-benzoxazol-3-yl)methyl)-2,7-diazaspiro[3.5]nonan-2-yl)methanone (7):

The compound was synthesized by following **General Procedure A.** Crude product was dissolved in DMSO (1 mL) and purified using the Gilson (Basic, 30x 100 mm column, 15-75% ACN/ 0.05% aqueous NH4OH, 10 min run). Fractions containing the desired product were concentrated to give **(7)** (8.7 mg, 0.02 mmol, 36% yield) as a clear glass.

^{1}H NMR (400 MHz, DMSO-d_6) δ: 8.03 (dd, J = 8.7, 5.4 Hz, 1H), 7.70 (dd, J = 9.1, 2.1 Hz, 1H), 7.41 (d, J = 1.9 Hz, 1H), 7.36 – 7.27 (m, 2H), 7.19 (d, J = 7.8 Hz, 1H), 3.96 (s, 2H), 3.88 (s, 2H), 3.70 (s, 2H), 2.30 (s, 2H), 2.43-2.25 (m, 7H), 1.72 (t, J = 5.4 Hz, 4H).

^{13}C{^{1}H} NMR (101 MHz, DMSO-d_6) δ 169.1, 163.7 (d, $J_{C\text{-}F}$ = 247.8 Hz), 163.0 (d, $J_{C\text{-}F}$ = 14.3 Hz), 156.2, 139.5, 136.3, 130.8, 129.3, 128.8, 125.3, 124.4 (d, $J_{C\text{-}F}$ = 11.3 Hz), 118.1, 112.7 (d, $J_{C\text{-}F}$ = 25.3 Hz), 97.2 (d, $J_{C\text{-}F}$ = 27.3 Hz), 62.6, 58.2, 51.9, 50.0, 34.9, 33.2, 19.3, 19.3.

HRMS (ESI) calculated for formula $C_{24}H_{27}FN_3O_2$ ($[M+H]^+$) 408.2081, found 408.2082.

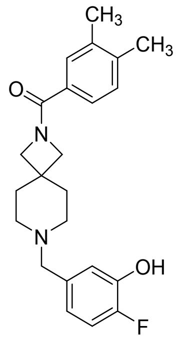

(3,4-dimethylphenyl)(7-((4-fluoro-3-hydroxyphenyl)methyl)-2,7-diazaspiro[3.5]nonan-2-yl)methanone (8):
The compound was synthesized by following **General Procedure A.** Crude product was dissolved in DMSO (1 mL) and purified using the Gilson (Basic, 30x 100 mm column, 15-75% ACN/ 0.05% aqueous NH_4OH, 10 min run). Fractions containing the desired product were concentrated to give **(8)** (10 mg, 0.03 mmol, 44% yield) as a clear glass.
^{1}H NMR (400 MHz, DMSO-d_6): δ 9.71 (br. s, 1H), 7.41 (d, *J* = 1.9 Hz, 1H), 7.34 (dd, *J* = 7.8, 1.9 Hz, 1H), 7.19 (d, *J* = 7.8 Hz, 1H), 7.02 (dd, *J* = 11.3, 8.2 Hz, 1H), 6.88 (dd, *J* = 8.8, 2.1 Hz, 1H), 6.65 (ddd, *J* = 8.2, 4.4, 2.1 Hz, 1H), 3.95 (s, 2H), 3.69 (s, 2H), 3.29 (s, 2H), 2.29-2.24 (m, 10H), 1.69 – 1.67 (m, 4H).
^{13}C{^{1}H} NMR (101 MHz, DMSO-d_6): δ 169.1, 150.0 (d, $J_{C\text{-}F}$ = 239.4 Hz), 144.5 (d, $J_{C\text{-}F}$ = 12.2 Hz), 139.5, 136.3, 135.1 (d, $J_{C\text{-}F}$ = 3.2 Hz), 130.8, 129.3, 128.8, 125.3, 119.3 (d, $J_{C\text{-}F}$ = 6.4 Hz), 117.8 (d, $J_{C\text{-}F}$ = 3.0 Hz), 115.5 (d, $J_{C\text{-}F}$ = 18.0 Hz), 62.7, 61.5, 58.2, 49.8, 35.0, 33.5, 19.4, 19.3.
HRMS (ESI) calculated for formula $C_{23}H_{28}FN_2O_2$ ([M+H]$^+$) 417.1495, found 383.2129.

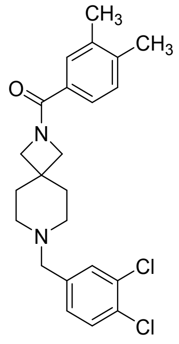

(7-(3,4-dichlorobenzyl)-2,7-diazaspiro[3.5]nonan-2-yl)(3,4-dimethylphenyl)methanone (9):
The compound was synthesized by following **General Procedure A.** Crude product was dissolved in DMSO (1 mL) and purified using the Gilson (Basic, 30x 100 mm column, 15-75% ACN/ 0.05% aqueous NH4OH, 10 min run). Fractions containing the desired product were concentrated to give **(9)** (13.4 mg, 0.03mmol, 54% yield) as a yellow glass.
^{1}H NMR (400 MHz, DMSO-d_6): δ 7.57 (d, *J* = 8.2 Hz, 1H), 7.53 (d, *J* = 1.9 Hz, 1H), 7.41 (d, *J* = 1.8 Hz, 1H), 7.34 (dd, *J* = 7.7, 1.9 Hz, 1H), 7.28 (dd, *J* = 8.2, 2.0 Hz, 1H), 7.18 (d, *J* = 7.8 Hz, 1H), 3.96 (s, 2H), 3.70 (s, 2H), 3.45 (s, 2H), 2.33-2.24 (m, 10H), 1.70 (t, *J* = 5.4 Hz, 4H).

$^{13}C\{^1H\}$ **NMR** (101 MHz, DMSO-d_6): δ 169.1, 139.5, 136.3, 130.8, 130.8, 130.4, 130.4, 129.4, 129.3, 129.0, 128.8, 125.3, 62.6, 60.4, 58.2, 49.7, 34.8, 33.4, 19.3, 19.3.
HRMS (ESI) calculated for formula $C_{23}H_{27}Cl_2N_2O$ ([M+H]$^+$) 417.1495, found 417.1494.

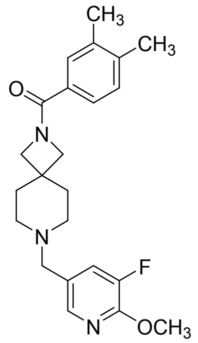

(3,4-dimethylphenyl)(7-((5-fluoro-6-methoxypyridin-3-yl)methyl)-2,7-diazaspiro[3.5]nonan-2-yl)methanone (10):
The compound was synthesized by following **General Procedure A.** Crude product was dissolved in DMSO (1 mL) and purified using the Gilson (Basic, 30x 100 mm column, 15-75% ACN/ 0.05% aqueous NH_4OH, 10 min run). Fractions containing the desired product were concentrated and purified once more via a Teledyne ISCO Combi-Flash system (liquid loading, 4G column, 0-10% MeOH/DCM) to give **(10)** (10.2 mg, 0.03 mmol, 43% yield) as a clear glass.
^1H **NMR** (400 MHz, DMSO-d_6): δ 7.86 (d, J = 1.8 Hz, 1H), 7.55 (dd, J = 11.3, 1.8 Hz, 1H), 7.41 (d, J = 1.9 Hz, 1H), 7.34 (dd, J = 7.8, 1.9 Hz, 1H), 7.18 (d, J = 7.8 Hz, 1H), 3.96 (s, 2H), 3.92 (s, 3H), 3.69 (s, 2H), 3.42 (s, 2H), 2.33-2.24 (m, 10H), 1.69 (t, J = 5.5 Hz, 4H).
$^{13}C\{^1H\}$ **NMR** (101 MHz, DMSO-d_6): δ 169.1, 151.7 (d, J_{C-F} = 11.3 Hz), 146.5 (d, J_{C-F} = 257.0 Hz), 141.2, 139.5, 136.3, 130.8, 129.3, 128.8, 125.3, 124.2 (d, J_{C-F} = 14.4 Hz), 62.6, 58.2, 57.9, 53.5, 49.5, 34.7, 33.4, 19.3, 19.3.
HRMS (ESI) calculated for formula $C_{23}H_{29}FN_3O_2$ ([M+H]$^+$) 398.2238, found 398.2238.

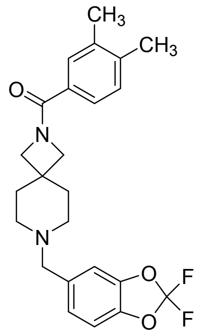

(7-((2,2-difluorobenzo[*d*][1,3]dioxol-5-yl)methyl)-2,7-diazaspiro[3.5]nonan-2-yl)(3,4-dimethylphenyl)methanone (11):

The compound was synthesized by following **General Procedure A.** Crude product was dissolved in DMSO (1 mL) and purified using the Gilson (Basic, 30x 100 mm column, 15-75% ACN/ 0.05% aqueous NH_4OH, 10 min run). Fractions containing the desired product were concentrated to give **(11)** (11.4 mg, 0.03 mmol, 45% yield) as a clear glass.

^{1}H NMR (400 MHz, DMSO-d_6): δ 7.41 (d, J = 1.8 Hz, 1H), 7.39 – 7.30 (m, 3H), 7.18 (d, J = 7.8 Hz, 1H), 7.11 (dd, J = 8.2, 1.6 Hz, 1H), 3.96 (s, 2H), 3.70 (s, 2H), 3.44 (s, 2H), 2.33-2.24 (m, 10H), 1.69 (t, J = 5.4 Hz, 4H).

^{13}C{^{1}H} NMR (101 MHz, DMSO-d_6): δ 169.1, 142.8, 141.6, 139.5, 136.3, 135.7, 130.8, 129.9 (t, $J_{C\text{-}F}$ = 252.2 Hz), 129.3, 128.8, 125.3, 124.5, 110.2, 109.6, 62.7, 61.3, 58.2, 49.7, 34.9, 33.5, 19.3, 19.3.

HRMS (ESI) calculated for formula $C_{24}H_{27}F_2N_2O$ ([M+H]$^+$) 429.1984, found 429.1982.

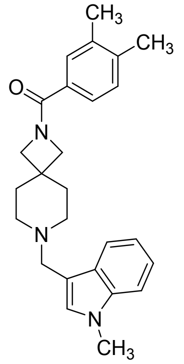

(3,4-dimethylphenyl)(7-[(1-methylindol-3-yl)methyl)-2,7-diazaspiro[3.5]nonan-2-yl)methanone (12):

The compound was synthesized by following **General Procedure A.** Crude product was dissolved in DMSO (1 mL) and purified using the Gilson (Basic, 30x 100 mm column, 15-75% ACN/ 0.05% aqueous NH_4OH, 10 min run). Fraction containing the desired product were concentrated to give **(12)** (9.1 mg, 0.02 mmol, 38% yield) as a clear glass.

^{1}H NMR (400 MHz, DMSO-d_6): δ 7.62 (dt, J = 7.9, 1.0 Hz, 1H), 7.40 – 7.32 (m, 3H), 7.18 (t, J = 3.9 Hz, 2H), 7.12 (ddd, J = 8.2, 7.0, 1.2 Hz, 1H), 7.00 (ddd, J = 7.9, 7.0, 1.0 Hz, 1H), 3.93 (s, 2H), 3.74 (s, 3H), 3.68 (s, 2H), 3.55 (s, 2H), 2.40-2.24 (m, 10H), 1.67 (t, J = 5.5 Hz, 4H).

^{13}C{^{1}H} NMR (101 MHz, DMSO-d_6): δ 169.1, 139.4, 136.7, 136.3, 130.8, 129.3, 128.79, 128.76, 127.9, 125.3, 121.0, 119.2, 118.5, 110.3, 109.5, 62.7, 58.3, 53.1, 49.8, 35.1, 33.6, 32.3, 19.34, 19.28.

HRMS (ESI) calculated for formula $C_{26}H_{32}N_3O$ ([M+H]$^+$) 402.2541, found 402.2540.

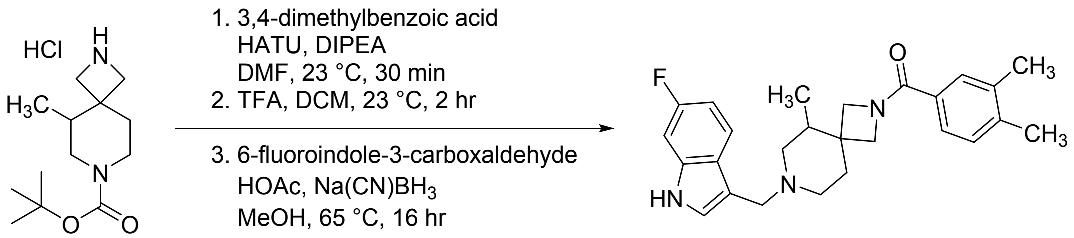

(3,4-dimethylphenyl)(7-((6-fluoro-1*H*-indol-3-yl)methyl)-5-methyl-2,7-diazaspiro[3.5]nonan-2-yl)methanone (13): To a test tube was added 3,4-dimethylbenzoic acid (27.5 mg, 0.18 mmol) and HATU (65.8 mg, 0.17 mmol), which were dissolved in anhydrous *N,N*-dimethylformamide (0.25 mL), followed by the addition of *N,N*-diisopropylethylamine (75.5 μL, 0.43 mmol). The solution was stirred at room temperature for 10 minutes. At this time, a solution of *tert*-butyl 5-methyl-2,7-diazaspiro[3.5]nonane-7-carboxylate hydrochloride (40.0 mg, 0.14 mmol) and *N,N*-diisopropylethylamine (75.5 μL, 0.43 mmol) in anhydrous *N,N*-dimethylformamide (0.25 mL) was added, and the solution was stirred at room temperature for 4.6 hours. The reaction was quenched with AQ $NaHCO_3$ and extracted 3x with DCM. The organic layers were combined and concentrated. This crude residue was dissolved in anhydrous DCM (1 mL) and cooled to 0 °C before the addition of trifluoroacetic acid (750 μL, 9.79 mmol). The reaction was allowed to slowly warm to room temperature. After 4.9 hours, the reaction was quenched with 2N NaOH and extracted with three portions of 3:1 $CHCl_3$:IPA. The organic layers were passed through a phase separator and concentrated. To a vial containing crude amine from the previous step was added 6-fluoro-1*H*-indole-3-carbaldehyde (25.7 mg, 0.16 mmol), anhydrous methanol (1 mL), and acetic acid (10.0 μL, 0.17 mmol). The solution was then stirred at room temperature for 12 minutes before the addition of sodium cyanoborohydride (18.6 mg, 0.30 mmol). The reaction was heated to 65 °C and stirred overnight. After 15.25 hours, the reaction was cooled to room temperature, quenched with AQ K_2CO_3, extracted 3x with DCM, and concentrated. The crude material was purified via RP-HPLC (Basic Gilson, 5-95% ACN in H_2O w/ 0.5 mL/L NH_4OH, medium column), then automated flash column chromatography (ISCO, 12 g column, 0-15% MeOH in DCM, solid loading) to afford **(13)** (17.3 mg, 41.2 μmol, 29% yield over 3 steps) as a clear glass.

^{1}H NMR (400 MHz, MeOD-d_4): δ 7.58 (appt. ddd, *J* = 8.7, 6.7, 5.6 Hz, 1H), 7.40 (m, 1H), 7.34 (appt. ddd, *J* = 7.4, 5.0, 1.7 Hz, 1H), 7.22 – 7.16 (m, 2H), 7.04 (appt. dt, *J* = 9.9, 2.4 Hz, 1H), 6.81 (m, 1H), 4.11 (d, *J* = 9.2 Hz, 1H), 4.02 – 3.86 (m, 2H), 3.76 – 3.61 (m, 3H), 2.95 – 2.51 (br. m, 2H), 2.29 (s, 3H), 2.29 (s, 3H), 2.34 – 2.09 (br. m, 1H, partially buried), 2.02 – 1.68 (m, 4H), 0.99 (appt. dd, *J* = 6.4, 5.5 Hz, 3H). Note: Several peaks exhibit higher multiplicity than they should, presumably due to slowly interconverting conformations. These peaks are annotated with "appt." for "apparent" multiplicities.

^{13}C{^{1}H} NMR (101 MHz, MeOD-d_4): δ 172.4, 161.1 (d, $J_{C\text{-}F}$ = 235.3 Hz), 141.7, 138.2, 137.9 (d, $J_{C\text{-}F}$ = 12.6 Hz), 131.6, 131.5, 130.7, 129.9, 126.6, 126.4 (d, $J_{C\text{-}F}$ = 2.7 Hz), 126.0, 120.7 (d, $J_{C\text{-}F}$ = 10.1 Hz), 111.1, 108.5 (d, $J_{C\text{-}F}$ = 24.7 Hz), 98.2 (d, $J_{C\text{-}F}$ = 25.9 Hz), 62.5, 57.7, 57.7, 53.9, 51.1, 51.0, 38.6, 38.6, 36.7, 19.8, 19.7, 13.2.

Note: Based on 2-D NMR experiments, the broad peak at 57.7 ppm corresponds to two distinct carbons. Additionally, due to the fluxional, asymmetric nature of the spirocycle, the peaks at 51.1 and 51.0 ppm correspond to only one carbon, as do the peaks at 38.6 and 38.6 ppm. The peaks at 131.6 and 131.5 ppm also correspond to a single quaternary carbon on the dimethylphenyl ring, which likely appear as distinct peaks due to the slow interconversion between spirocycle conformers and amide rotamers.

HRMS (ESI) calculated for formula $C_{26}H_{31}FN_3O$ ($[M+H]^+$) 420.2446, found 420.2444.

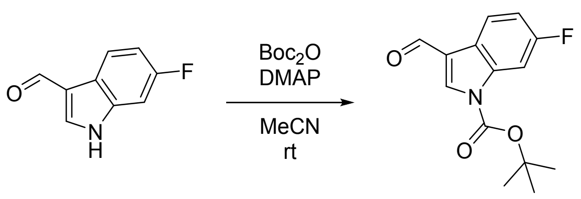

***Tert*-butyl 6-fluoro-3-formyl-1*H*-indole-1-carboxylate (35):** To a vial was added 6-fluoro-1*H*-indole-3-carbaldehyde (202.5 mg, 1.24 mmol) and 4-dimethylaminopyridine (15.5 mg, 0.127 mmol) , which were dissolved in anhydrous MeCN (1.7 mL). To the solution was added di-*tert*-butyl decarbonate (310 uL, 1.35 mmol). The reaction was stirred at room temperature for 50 minutes, then quenched with AQ $NaHCO_3$ and extracted with 3 portions of DCM. The organic layers were combined and concentrated. The crude material was purified via automated flash column chromatography (0-25% EtOAc in hexanes) to afford **(35)** (283.5 mg, 1.08 mmol, 87% yield) as a light-yellow solid.

^{1}H NMR (400 MHz, DMSO-d_6): δ 10.07 (s, 1H), 8.69 (s, 1H), 8.15 (dd, *J* = 8.7, 5.7 Hz, 1H), 7.84 (dd, *J* = 10.2, 2.4 Hz, 1H), 7.29 (ddd, *J* = 9.5, 8.8, 2.5 Hz, 1H), 1.66 (s, 9H).

^{13}C{^{1}H} NMR (101 MHz, DMSO-d_6): δ 187.1, 160.6 (d, J_{C-F} = 240.1 Hz), 148.1, 138.8 (d, J_{C-F} = 3.1 Hz), 135.6 (d, J_{C-F} = 13.0 Hz), 122.7 (d, J_{C-F} = 10.0 Hz), 122.2, 120.4, 112.7 (d, J_{C-F} = 23.8 Hz), 102.2 (d, J_{C-F} = 28.6 Hz), 86.1, 27.5.

HRMS (ESI) calculated for formula $C_{10}H_7FNO_3$ ([M-C(CH$_3$)$_3$+H]$^+$) 208.0404, found 208.0406.

Note: ^{1}H spectrum aligns with literature.[2] No ^{13}C{^{1}H} data reported.

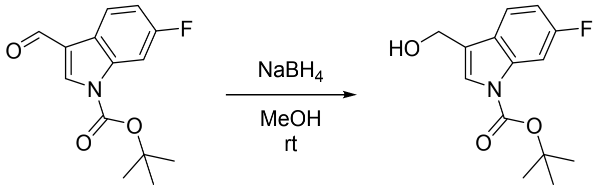

***Tert*-butyl 6-fluoro-3-(hydroxymethyl)-1*H*-indole-1-carboxylate (36):** To a vial containing **35** (282.9 mg, 1.07 mmol) was added anhydrous methanol (4.2 mL), followed by sodium borohydride (80.7 mg, 2.13 mmol). The mixture was stirred at room temperature for 21 minutes, then quenched with brine and extracted 3x with DCM. The organic layers were combined and concentrated to afford **(36)** (273 mg,1.029 mmol, 96% yield) as a clear residue. Product was pure and required no further purification.

^{1}H NMR (400 MHz, MeOD-d_4): δ 7.82 (dd, *J* = 10.4, 1.8 Hz, 1H), 7.62 (dd, *J* = 8.7, 5.4 Hz, 1H), 7.56 (m, 1H), 7.02 (ddd, *J* = 9.4, 8.7, 2.4 Hz, 1H), 4.73 (d, *J* = 1.0 Hz, 2H), 1.67 (s, 9H). Note: Hydroxyl *OH* peak is not observed in this solvent.

^{13}C{^{1}H} NMR (101 MHz, MeOD-d_4): δ 162.4 (d, J_{C-F} = 238.8 Hz), 150.8, 137.4 (d, J_{C-F} = 12.9 Hz), 127.19, 124.8 (d, J_{C-F} = 3.8 Hz), 122.2, 121.5 (d, J_{C-F} = 10.0 Hz), 111.6 (d, J_{C-F} = 24.4 Hz), 103.1 (d, J_{C-F} = 28.8 Hz), 85.3, 56.9, 28.3.

HRMS (ESI) calculated for formula $C_{10}H_7FNO_3$ ([M-OC(CH$_3$)$_3$]$^+$) 192.0455, found 192.0456.

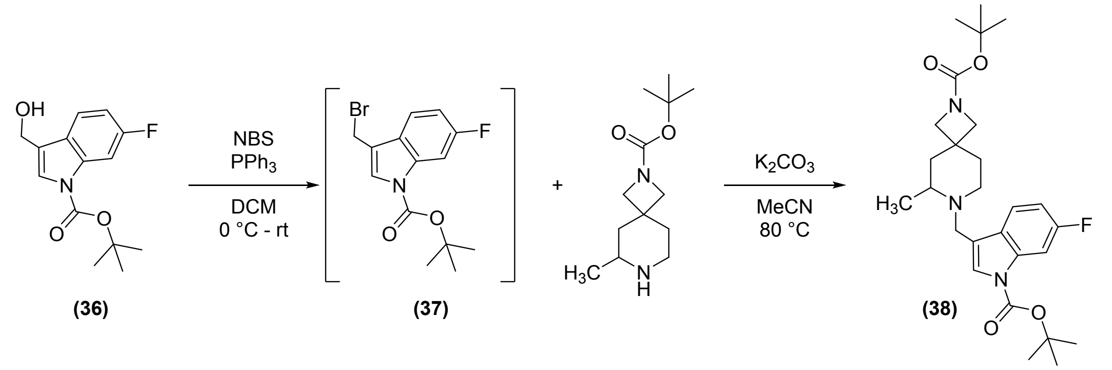

$\textit{Tert}$-butyl 7-((1-(*tert*-butoxycarbonyl)-6-fluoro-1*H*-indol-3-yl)methyl)-6-methyl-2,7-diazaspiro[3.5]nonane-2-carboxylate (38): To a vial containing **(36)** (270.7 mg, 1.02 mmol) was added anhydrous DCM (5 mL). The solution was cooled to 0 °C, then triphenylphosphine (325.1 mg, 1.24 mmol) was added. The solution was stirred at 0 °C for 30 minutes, then *N*-bromosuccinimide (239.8 mg, 1.35 mmol) was added. The solution was allowed to warm to room temperature, after which it was stirred for a further 70 minutes. The reaction was then diluted with DCM, washed with AQ $NaHCO_3$, and concentrated to afford **(37)** as a red oil. To a separate vial was then added *tert*-butyl 6-methyl-2,7-diazaspiro[3.5]nonane-2-carboxylate (204.1 mg, 0.85 mmol) and K_2CO_3 (432 mL, 3.08 mmol), followed by anhydrous MeCN (3.5mL). To this mixture was then added a solution of fresh **(37)** in anhydrous MeCN (3.5mL). The mixture was heated to 80 °C for 55 minutes, then cooled to room temperature and quenched with 2 mL AQ $NaHCO_3$. The organic layer was separated, and the AQ layer was extracted 3x with DCM. All organic layers were combined and concentrated. The crude material was dry loaded onto celite and purified via automated flash column chromatography (Teledyne ISCO, 0-15% MeOH in DCM) to afford **(38)** (229.7 mg, 0.417 mmol, 55% yield) as a red-gold oil.

^{1}H NMR (400MHz, MeOD-d_4): δ 7.81 (dd, *J* = 10.3, 1.5 Hz, 1H), 7.67 (dd, *J* = 8.7, 5.4 Hz, 1H), 7.53 (s, 1H), 7.00 (m, 1H), 4.16 (d, *J* = 13.8 Hz, 1H), 3.69 – 3.48 (m, 3H), 3.26 (d, *J* = 13.7 Hz, 1H), 2.82 (dt, *J* = 12.0, 3.5 Hz, 1H), 2.32 – 2.20 (m, 1H), 1.93 (td, *J* = 12.3, 2.4 Hz, 1H), 1.86 (dt, *J* = 13.3, 2.5 Hz, 1H), 1.69 (m, 1H, partially buried), 1.67 (s, 9H), 1.63 – 1.48 (m, 2H), 1.42 (s, 9H), 1.29 (d, *J* = 6.1 Hz, 3H), 1.28 (m, 1H, partially buried).

^{13}C{^{1}H} NMR (101MHz, MeOD-d_4): δ 162.3 (d, J_{C-F} = 238.9 Hz), 158.3, 150.7, 137.1 (d, J_{C-F} = 13.0 Hz), 128.4 (d, J_{C-F} = 1.5 Hz), 126.3 (d, J_{C-F} = 3.8 Hz), 121.9 (d, J_{C-F} = 9.9 Hz), 118.3, 111.6 (d, J_{C-F} = 24.4 Hz), 103.1 (d, J_{C-F} = 28.7 Hz), 85.4, 80.9, 61.3, 60.2, 59.2, 55.3, 50.1, 45.0, 36.1, 34.9, 28.6, 28.3, 20.6.

HRMS (ESI) calculated for formula $C_{27}H_{39}FN_3O_4$ ($[M+H]^+$) 488.2919, found 488.2926.

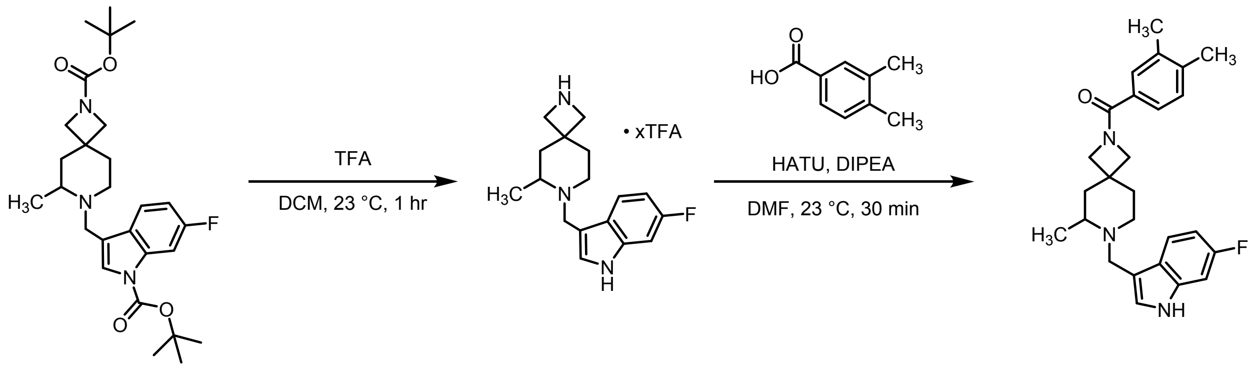

(3,4-dimethylphenyl)(7-((6-fluoro-1*H*-indol-3-yl)methyl)-6-methyl-2,7-diazaspiro[3.5]nonan-2-yl)methanone (14): In a vial, **(38)** (105 mg, 0.27 mmol) in dichloromethane (1 mL) was cooled to 0 °C before the addition of trifluoroacetic acid (0.5 mL, 6.53 mmol) . The reaction was then warmed to room temperature and stirred for 1 hour. At this time the reaction was made concentrated to afford the TFA salt of **(39)** (0.185 g, 0.29 mmol, > 98% yield) as a clear syrup, which was taken directly to the next step without purification. Then, HATU (14.0 mg, 0.04 mmol) and 3,4-dimethylbenzoic acid (5.6 mg, 0.04 mmol) were added to a vial and dissolved in DMF (0.5 mL), followed by addition of *N,N*-diisopropylethylamine (25.7 μL, 0.15 mmol). The solution was stirred at room temperature for 10 minutes. At this time, **(39)** (15.5 mg, 0.02 mmol) in DMF (0.5 mL) was added, and the solution was stirred for an additional 30 minutes at which point LCMS indicated full conversion. Crude product was purified using the Gilson (Basic, 30x 100 mm column, 15-75% ACN/ 0.05% aqueous NH_4OH, 10 min run). Fractions containing the desired product were purified once more via Teledyne Isco combi-flash system (liquid loading, 4g column, 0-10% MeOH/DCM) to give **(14)** (4.4 mg, 0.011 mmol, 43% yield) as a clear glass.

^{1}H NMR (400 MHz, MeOH-d_4): δ 7.60 (appt. td, *J* = 8.7, 5.3 Hz, 1H), 7.40 (m, 1H), 7.33 (appt. ddd, *J* = 7.7, 3.7, 1.6 Hz, 1H), 7.26 (appt. d, *J* = 7.8 Hz, 1H), 7.19 (d, *J* = 7.9 Hz, 1H), 7.06 (appt. ddd, *J* = 9.8, 4.1, 2.3 Hz, 1H), 6.84 (appt. tdd, *J* = 9.2, 5.5, 2.3 Hz, 1H), 4.31 (d, *J* = 13.4 Hz, 1H), 4.10 – 3.94 (m, 2H), 3.89 – 3.75 (m, 2H), 3.64 (m, 1H), 3.03 (appt. d, *J* = 11.6 Hz, 1H), 2.62 – 2.39 (m, 1H), 2.29 (s, 3H), 2.29 (s, 3H), 2.19 (m, 1H), 1.99 (appt. d, *J* = 13.4 Hz, 1H), 1.87 (m, 1H), 1.77 – 1.53 (m, 2H), 1.40 (appt. dd, *J* = 5.7, 3.4 Hz, 3H). Note: Several peaks exhibit higher multiplicity than they should, presumably due to slowly interconverting conformations. These peaks are annotated with "appt." for "apparent" multiplicities. The peaks at 3.03 and 1.99 ppm are also denoted as such, but in this case they exhibit lesser multiplicity.

^{13}C{^{1}H} NMR (101 MHz, MeOH-d_4): δ 172.6, 172.6, 161.2 (d, *J* = 235.6 Hz), 141.7, 138.1, 137.8 (d, *J* = 12.9 Hz), 131.6, 130.6, 129.9, 127.1, 126.5, 126.5, 126.1, 120.6 (d, *J* = 10.1 Hz), 109.8, 108.7 (d, *J* = 24.8 Hz), 98.3 (d, *J* = 25.9 Hz), 64.8, 63.9, 60.2, 59.3, 55.1, 54.9, 49.5, 48.5, 44.2, 35.3, 20.2, 19.8, 19.7. Note: Based on 2-D NMR experiments, the following peak pairs correspond to a single carbon: 172.58 and 172.56 ppm (carbonyl); 126.51 and 126.46 ppm (-*CH ortho* to amide); 64.8 and 63.9 ppm (azetidine -CH_2); 60.2 and 59.3 ppm (azetidine -CH_2); and 55.1 and 54.9 ppm (piperidine -*CH*). These peaks appear split due to slow interconversion between conformers, as well as slow conversion between amide rotamers. The peaks at 49.5 ppm (piperidine C6) and 48.5 ppm

(benzylic -CH_2) were assigned via HSQC as they were buried under the MeOD-d_4 signal. Finally, the spirocycle quaternary carbon was not directly observed as a peak or via 2-D experiments. Based on the chemical shift of the analogous carbon in compound **(37)**, this peak could be buried under the peak at 35.3 ppm.
HRMS (ESI) calculated for formula $C_{26}H_{31}FN_3O$ ([M+H]$^+$) 420.2446, found 420.2442.

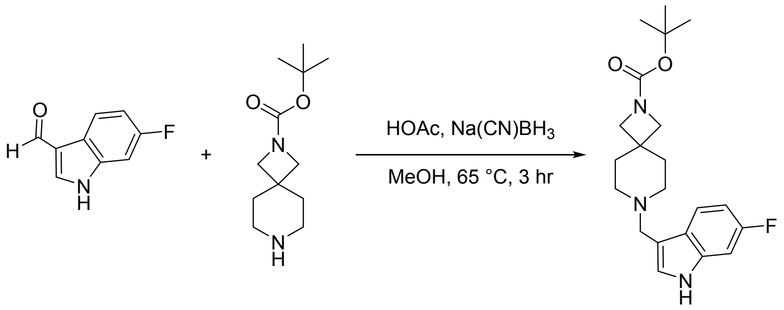

***Tert*-butyl 7-((6-fluoro-1*H*-indol-3-yl)methyl)-2,7-diazaspiro[3.5]nonane-2-carboxylate (15):**
In a vial *tert*-butyl 2,7-diazaspiro[3.5]nonane-2-carboxylate (166.5 mg, 0.74 mmol), and 6-fluoroindole-3-carboxaldehyde (100 mg, 0.61 mmol) were dissolved in methanol (3 mL), followed by the addition of acetic acid (38.6 µL, 0.67 mmol) and sodium cyanoborohydride (77.0 mg, 1.23 mmol). The vial was sealed, and the reaction was heated to 65 ˚C and stirred for three hours. The reaction mixture was then cooled to room temperature and made concentrated before quenching with saturated sodium bicarbonate and extracting with ethyl acetate (x3). Organics were passed through a phase separator, made concentrated, and purified using Teledyne ISCO Combi-Flash system (solid loading, 40G column, 0-10% MeOH/DCM) to give **(15**) (198.1 mg, 0.53 mmol, 87% yield) as a white solid.
^{1}H NMR (400 MHz, MeOD-d_4): δ 7.57 (dd, J = 8.7, 5.3 Hz, 1H), 7.18 (s, 1H), 7.04 (dd, J = 9.9, 2.3 Hz, 1H), 6.81 (ddd, J = 9.7, 8.8, 2.3 Hz, 1H), 3.67 (s, 2H), 3.62-3.52 (m, 4H), 2.66-2.22 (br. m, 4H), 1.75 (t, J = 5.5 Hz, 4H), 1.42 (s, 9H).
^{13}C{^{1}H} NMR (101 MHz, MeOD-d_4): δ 161.1 (d, $J_{C\text{-}F}$ = 235.1 Hz), 158.3, 137.8 (d, $J_{C\text{-}F}$ = 12.5 Hz), 126.6 (d, $J_{C\text{-}F}$ = 3.4 Hz), 126.1, 120.6 (d, $J_{C\text{-}F}$ = 10.1 Hz), 111.4, 108.4 (d, $J_{C\text{-}F}$ = 24.7 Hz), 98.1 (d, $J_{C\text{-}F}$ = 25.9 Hz), 80.9, 60.6, 59.4, 53.9, 51.1, 36.0, 34.3, 28.6.
HRMS (ESI) calculated for formula $C_{21}H_{29}FN_3O_2$ ([M+H]$^+$) 374.2238, found 374.2245.

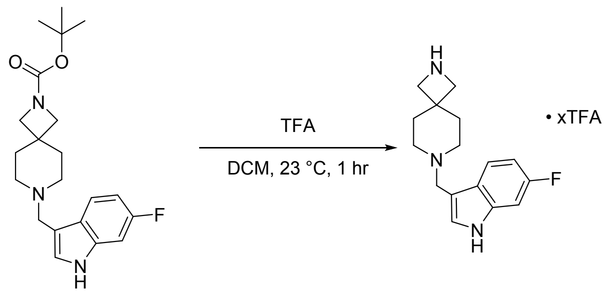

7-((6-fluoro-1*H*-indol-3-yl)methyl)-2,7-diazaspiro[3.5]nonane trifluoroacetic acid (16):

In a vial, **(15**) (0.458 g, 1.23 mmol) in dichloromethane (3 mL) was cooled to 0 °C before the addition of trifluoroacetic acid (1.0 mL, 13.06 mmol). The reaction was then warmed to room temperature and stirred for 1 hour. At this time the reaction was made concentrated and used as the TFA salt without any further purification. **(16**) (781 mg, 1.27 mmol, > 98% yield) was obtained as a clear syrup.

^{1}H NMR (400 MHz, DMSO-d_6) δ: 9.63 (br. s, 1H), 8.95 (br. s, 2H), 7.75 (dd, J = 8.7, 5.4 Hz, 1H), 7.53 (d, J = 2.6 Hz, 1H), 7.23 (dd, J = 10.0, 2.3 Hz, 1H), 7.01 – 6.95 (m, 1H), 4.39 (d, J = 4.6 Hz, 2H), 3.83 (t, J = 6.1 Hz, 2H), 3.72 (t, J = 6.1 Hz, 2H), 3.36 (d, J = 12.1 Hz, 2H), 2.97 (q, J = 11.0 Hz, 2H), 2.17 (d, J = 14.10 Hz, 2H), 1.84 – 1.76 (m, 2H).

^{13}C{^{1}H} NMR (101 MHz, DMSO-d_6) δ: 159.1 (d, $J_{C\text{-}F}$ = 235.3 Hz), 158.5 (q, $J_{C\text{-}F}$ = 35.1 Hz), 135.9 (d, $J_{C\text{-}F}$ = 12.8 Hz), 129.4 (d, $J_{C\text{-}F}$ = 3.2 Hz), 124.3, 119.8 (d, $J_{C\text{-}F}$ = 10.2 Hz), 116.1 (q, $J_{C\text{-}F}$ = 293.4 Hz), 108.2 (d, $J_{C\text{-}F}$ = 24.5 Hz), 102.9, 97.9 (d, $J_{C\text{-}F}$ = 25.6 Hz), 54.1, 53.6, 50.2, 47.5, 34.6, 30.8.

HRMS (ESI) calculated for formula $C_{16}H_{21}FN_3$ ([M+H]$^+$) 274.1714, found 274.1718.

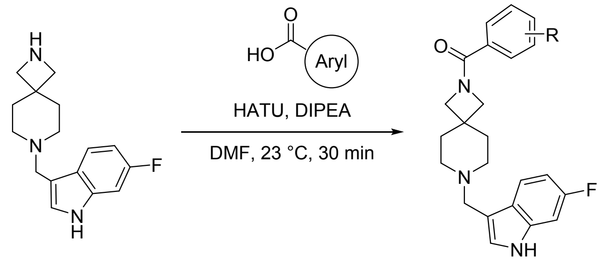

(33) **(12-28)**

General Procedure B:

In a vial, HATU (1.6 equiv.) and a benzoic acid (1.6 equiv.) were dissolved in DMF (0.5mL), followed by the addition of *N,N*-diisopropylethylamine (3.8 equiv) and stirring at room temperature for 10 minutes. At this time**, (33**) (1 equiv.) in DMF (0.5 mL) was added, and the solution was stirred for an additional 30 minutes. Crude product was then purified either using Reverse phase HPLC (Gilson) or normal phase silica gel chromatography (ISCO).

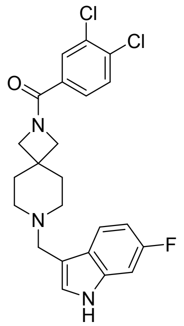

(3,4-dichlorophenyl)(7-((6-fluoro-1H-indol-3-yl)methyl)-2,7-diazaspiro[3.5]nonan-2-yl)methanone (17):

The compound was synthesized by following **General Procedure B**. Crude product was purified using the Gilson (Basic, 30x 100 mm column, 15-75% ACN/ 0.05% aqueous NH_4OH, 10 min run). The fractions containing the desired product were concentrated to give **(17)** (7.1 mg, 0.02 mmol, 50% yield) as a clear glass.

^{1}H NMR (400 MHz, MeOD-d_4): δ 7.81 (d, J = 1.9 Hz, 1H), 7.61 (d, J = 8.4 Hz, 1H), 7.59 – 7.53 (m, 2H), 7.19 (s, 1H), 7.04 (dd, J = 9.9, 2.3 Hz, 1H), 6.80 (ddd, J = 9.7, 8.8, 2.3 Hz, 1H), 4.04 (s, 2H), 3.83 (s, 2H), 3.68 (s, 2H), 2.70 – 2.21 (br. m, 4H), 1.82 (t, J = 5.3 Hz, 4H). Note: Indole *NH* not observed.

^{13}C{^{1}H} NMR (101 MHz, MeOD-d_4): δ 169.5, 161.1 (d, J_{C-F} = 235.0 Hz), 137.8 (d, J_{C-F} = 12.5 Hz), 136.4, 134.5, 133.8, 131.8, 131.1, 128.6, 126.5 (d, J_{C-F} = 3.4 Hz), 126.1, 120.6 (d, J_{C-F} = 10.2 Hz), 111.4, 108.4 (d, J_{C-F} = 24.8 Hz), 98.1 (d, J_{C-F} = 25.9 Hz), 64.2, 59.9, 53.9, 51.0, 35.9, 35.1.

HRMS (ESI) calculated for formula $C_{23}H_{23}Cl_2FN_3O$ ([M+H]$^+$) 446.1197, found 446.1194.

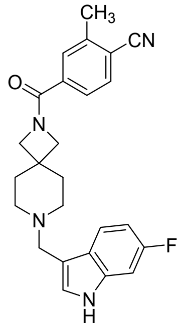

4-(7-((6-fluoro-1*H*-indol-3-yl)methyl)-2,7-diazaspiro[3.5]nonane-2-carbonyl)-2-methylbenzonitrile (18):

The compound was synthesized by following **General Procedure B**. Crude product was purified using the Gilson (Basic, 30x 100 mm column, 15-75% ACN/ 0.05% aqueous NH_4OH, 10 min run). Fractions containing the desired product were concentrated to give **(18**) (9.4 mg, 0.02 mmol, 69% yield) as a clear glass.

^{1}H NMR (400 MHz, MeOD-d_4): δ 7.73 (d, J = 8.0 Hz, 1H), 7.65 (m, 1H), 7.60 – 7.54 (m, 2H), 7.18 (s, 1H), 7.04 (dd, J = 9.9, 2.3 Hz, 1H), 6.80 (ddd, J = 9.7, 8.8, 2.3 Hz, 1H), 4.00 (s, 2H), 3.84 (s, 2H), 3.69 (s, 2H), 2.67 - 2.28 (br. m, 4H), 2.56 (s, 3H), 1.82 (t, J = 5.3 Hz, 4H). Note: Indole *NH* not observed.

^{13}C{^{1}H} NMR (101 MHz, MeOD-d_4): δ 170.5, 161.1 (d, J_{C-F} = 235.2 Hz), 143.7, 138.4, 137.8 (d, J_{C-F} = 12.5 Hz), 133.8, 130.5, 126.8, 126.6 (d, J_{C-F} = 3.5 Hz), 126.1, 120.6 (d, J_{C-F} = 10.1 Hz), 118.2, 116.1, 111.4, 108.4 (d, J_{C-F} = 24.8 Hz), 98.1 (d, J_{C-F} = 25.8 Hz), 64.0, 59.8, 53.9, 51.0, 35.9, 35.1, 20.4.

HRMS (ESI) calculated for formula $C_{25}H_{26}FN_4O$ ([M+H]$^+$) 417.2085, found 417.2084.

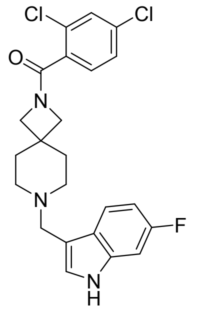

(2,4-dichlorophenyl)(7-((6-fluoro-1*H*-indol-3-yl)methyl)-2,7-diazaspiro[3.5]nonan-2-yl)methanone (19):

The compound was synthesized by following **General Procedure B**. Crude product was purified using the Gilson (Basic, 30x 100 mm column, 15-75% ACN/ 0.05% aqueous NH_4OH, 10 min run). Fractions containing the desired product were concentrated to give **(19)** (6.2 mg, 0.01 mmol, 43% yield) as a clear glass.

^{1}H NMR (400 MHz, MeOD-d_4): δ 7.60 – 7.55 (m, 2H), 7.43 (dd, J = 8.2, 1.9 Hz, 1H), 7.38 (d, J = 8.2 Hz, 1H), 7.18 (s, 1H), 7.03 (dd, J = 9.9, 2.3 Hz, 1H), 6.80 (ddd, J = 9.7, 8.8, 2.3 Hz, 1H), 3.84 (s, 2H), 3.67 (s, 2H), 3.64 (s, 2H), 2.64 – 2.28 (br. m, 4H), 1.88 – 1.74 (m, 4H). Note: Indole *NH* not observed.

^{13}C{^{1}H} NMR (101 MHz, MeOD-d_4): δ 169.3, 161.1 (d, $J_{C\text{-}F}$ = 235.3 Hz), 137.8 (d, $J_{C\text{-}F}$ = 12.5 Hz), 137.5, 133.8, 132.6, 130.8, 130.7, 129.0, 126.5 (d, $J_{C\text{-}F}$ = 3.4 Hz), 126.1, 120.6 (d, $J_{C\text{-}F}$ = 10.1 Hz), 111.5, 108.4 (d, $J_{C\text{-}F}$ = 24.8 Hz), 98.1 (d, $J_{C\text{-}F}$ = 25.9 Hz), 61.8, 59.2, 53.9, 51.0, 35.8, 35.1.

HRMS (ESI) calculated for formula $C_{23}H_{23}Cl_2FN_3O$ ($[M+H]^+$) 446.1195, found 446.1197.

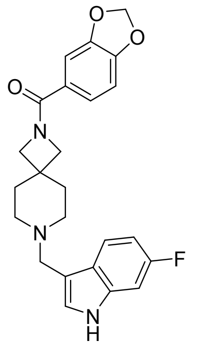

Benzo[*d*][1,3]dioxol-5-yl(7-((6-fluoro-1*H*-indol-3-yl)methyl)-2,7-diazaspiro[3.5]nonan-2-yl)methanone (20):

The compound was synthesized by following **General Procedure B**. Crude product was purified using the Gilson (Basic, 30x 100 mm column, 15-75% ACN/ 0.05% aqueous NH_4OH, 10 min run). Fractions containing the desired product were concentrated to give **(20)** (9.7 mg, 0.02 mmol, 71% yield) as a white solid.

^{1}H NMR (400 MHz, MeOD-d_4): δ 7.60 (dd, J = 8.7, 5.3 Hz, 1H), 7.25 (s, 1H), 7.20 (dd, J = 8.1, 1.7 Hz, 1H), 7.13 (d, J = 1.6 Hz, 1H), 7.06 (dd, J = 9.9, 2.3 Hz, 1H), 6.87 (d, J = 8.1 Hz, 1H), 6.84 (ddd, J

= 9.6, 8.8, 2.3 Hz, 1H), 6.02 (s, 2H), 4.07 (s, 2H), 3.83 (s, 4H), 2.91 – 2.32 (m, 4H), 1.86 (t, *J* = 5.1 Hz, 4H). Note: Indole *NH* not observed.

^{13}C{^{1}H} NMR (101 MHz, MeOD-d_4): δ 171.6, 161.2 (d, $J_{C\text{-}F}$ = 235.4 Hz), 151.7, 149.3, 137.8 (d, $J_{C\text{-}F}$ = 12.6 Hz), 127.8, 127.2, 126.0, 124.1, 120.6 (d, $J_{C\text{-}F}$ = 10.2 Hz), 109.1, 109.0, 108.7 (d, $J_{C\text{-}F}$ = 24.9 Hz), 103.2, 98.2 (d, $J_{C\text{-}F}$ = 26.0 Hz), 64.4, 59.7, 53.7, 50.9, 35.4, 34.8.

HRMS (ESI) calculated for formula $C_{24}H_{25}FN_3O_3$ ([M+H]$^+$) 422.1874, found 422.1880.

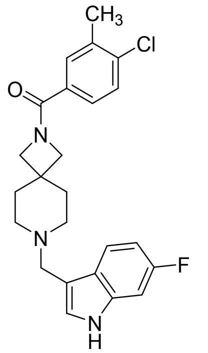

(3-chloro-4-methylphenyl)(7-((6-fluoro-1*H*-indol-3-yl)methyl)-2,7-diazaspiro[3.5]nonan-2-yl)methanone (21):

The compound was synthesized by following **General Procedure B**. Crude product was purified using the Gilson (Basic, 30x 100 mm column, 15-75% ACN/ 0.05% aqueous NH_4OH, 10 min run). Fractions containing the desired product were concentrated to give **(21)** (8.4 mg, 0.02 mmol, 61% yield) as a clear glass.

^{1}H NMR (400 MHz, MeOD-d_4) δ: 7.60 – 7.54 (m, 2H), 7.45 – 7.39 (m, 2H), 7.18 (s, 1H), 7.04 (dd, *J* = 9.9, 2.3 Hz, 1H), 6.80 (ddd, *J* = 9.7, 8.8, 2.4 Hz, 1H), 4.01 (s, 2H), 3.81 (s, 2H), 3.67 (s, 2H), 2.69 – 2.24 (br. m, 4H), 2.39 (m, 3H), 1.80 (t, *J* = 5.4 Hz, 4H). Note: Indole *NH* not observed.

^{13}C{^{1}H} NMR (101 MHz, MeOD-d_4) δ: 171.2, 161.1 (d, $J_{C\text{-}F}$ = 235.2 Hz), 138.4, 137.8 (d, $J_{C\text{-}F}$ = 12.5 Hz), 137.7, 132.9, 131.5, 130.2, 127.9, 126.5 (d, $J_{C\text{-}F}$ = 3.3 Hz), 126.1, 120.6 (d, $J_{C\text{-}F}$ = 10.2 Hz), 111.4, 108.4 (d, $J_{C\text{-}F}$ = 24.8 Hz), 98.1 (d, $J_{C\text{-}F}$ = 25.8 Hz), 64.3, 59.8, 53.9, 51.1, 35.9, 35.1, 20.0.

HRMS (ESI) calculated for formula $C_{24}H_{26}ClFN_3O$ ([M+H]$^+$) 426.1740, found 426.1743.

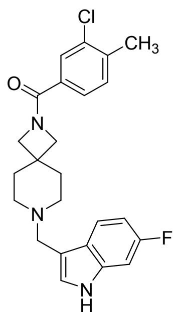

(4-chloro-3-methylphenyl)(7-((6-fluoro-1*H*-indol-3-yl)methyl)-2,7-diazaspiro[3.5]nonan-2-yl)methanone (22):

The compound was synthesized by following **General Procedure B**. Crude product was purified using the Gilson (Basic, 30x 100 mm column, 15-75% ACN/ 0.05% aqueous NH_4OH, 10 min run). Fractions containing the desired product were concentrated to give **(22)** (8.8 mg, 0.02 mmol, 64% yield) as a clear glass.

^{1}H NMR (400 MHz, MeOD-d_4): δ 7.64 (d, J = 1.7 Hz, 1H), 7.58 (dd, J = 8.7, 5.4 Hz, 1H), 7.47 (dd, J = 7.9, 1.7 Hz, 1H), 7.37 (d, J = 8.0 Hz 1H), 7.19 (s, 1H), 7.04 (dd, J = 9.9, 2.3 Hz, 1H), 6.81 (ddd, J = 9.7, 8.8, 2.3 Hz, 1H), 4.04 (s, 2H), 3.83 (s, 2H), 3.69 (s, 2H), 2.66 - 2.31 (br. m, 4H), 2.41 (s, 3H), 1.82 (t, J = 5.4 Hz, 4H). Note: Indole *NH* not observed.

^{13}C{^{1}H} NMR (101 MHz, MeOD-d_4): δ 170.6, 161.1 (d, J_{C-F} = 235.2 Hz), 140.8, 137.8 (d, J_{C-F} = 12.4 Hz), 135.6, 133.5, 132.2, 129.5, 127.4, 126.6 (d, J_{C-F} = 3.3 Hz), 126.1, 120.7 (d, J_{C-F} = 10.2 Hz), 111.4, 108.4 (d, J_{C-F} = 24.8 Hz), 98.1 (d, J_{C-F} = 25.9 Hz), 64.3, 59.8, 53.9, 51.1, 35.9, 35.1, 20.1.

HRMS (ESI) calculated for formula $C_{24}H_{26}ClFN_3O$ ($[M+H]^+$) 426.1743, found 426.1739.

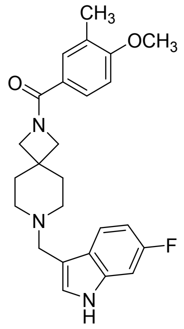

(7-((6-fluoro-1*H*-indol-3-yl)methyl)-2,7-diazaspiro[3.5]nonan-2-yl)(4-methoxy-3-methylphenyl)methanone (23):

The compound was synthesized by following **General Procedure B**. Crude product was purified using the Gilson (Basic, 30x 100 mm column, 15-75% ACN/ 0.05% aqueous NH_4OH, 10 min run). Fractions containing the desired product were concentrated and were purified once more using Teledyne ISCO Combi-Flash system (liquid loading, 4G column, 0-10% MeOH/DCM) to give **(23)** (7.1 mg,0.02 mmol, 52% yield) as a clear glass.

^{1}H NMR (400 MHz, MeOD-d_4): δ 7.58 (dd, J = 8.8, 5.4 Hz, 1H), 7.49 (dd, J = 8.5, 2.3 Hz, 1H), 7.46 - 7.43 (m, 1H), 7.18 (s, 1H), 7.04 (dd, J = 9.9, 2.3 Hz, 1H), 6.93 (d, J = 8.5 Hz, 1H), 6.81 (ddd, J = 9.7, 8.8, 2.3 Hz, 1H), 4.04 (s, 2H), 3.86 (s, 3H), 3.81 (s, 2H), 3.68 (s, 2H), 2.66 - 2.27 (br. m, 4H), 2.20 (s, 3H), 1.80 (t, J = 5.4 Hz, 4H). Note: Indole -*NH* not observed.

^{13}C{^{1}H} NMR (101 MHz, MeOD-d_4): δ 172.2, 161.7, 161.1 (d, J_{C-F} = 235.1 Hz), 137.8 (d, J_{C-F} = 12.5 Hz), 131.3, 128.7, 127.8, 126.6 (d, J_{C-F} = 3.5 Hz), 126.1, 125.6, 120.7 (d, J_{C-F} = 10.2 Hz), 111.4, 110.5, 108.4 (d, J_{C-F} = 24.8 Hz), 98.1 (d, J_{C-F} = 25.9 Hz), 64.6, 59.8, 56.0, 53.9, 51.1, 36.0, 35.0, 16.3.

HRMS (ESI) calculated for formula $C_{25}H_{29}FN_3O_2$ ($[M+H]^+$) 422.2237, found 422.2238.

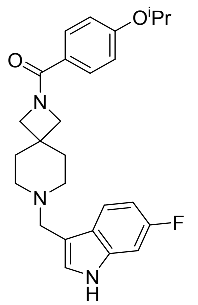

(7-((6-fluoro-1*H*-indol-3-yl)methyl)-2,7-diazaspiro[3.5]nonan-2-yl)(4-isopropoxyphenyl)methanone (24):

The compound was synthesized by following **General Procedure B**. Crude product was purified using the Gilson (Basic, 30x 100 mm column, 15-75% ACN/ 0.05% aqueous NH_4OH, 10 min run). Fractions containing the desired product were concentrated and purified again via Teledyne ISCO Combi-Flash system (liquid loading, 4G column, 0-10% MeOH/DCM) to give **(24)** (7.8 mg,0.02 mmol, 55% yield) as a clear glass.

^{1}H NMR (400 MHz, DMSO-d_6): δ 10.98 (s, 1H), 7.62 – 7.56 (m, 3H), 7.20 – 7.20 (m, 1H), 7.10 (dd, *J* = 10.2, 2.4 Hz, 1H), 6.94 – 6.91 (m, 2H), 6.82 (ddd, *J* = 9.7, 8.6, 2.4 Hz, 1H), 4.66 (s, *J* = 6.0 Hz, 1H), 3.97 (s, 2H), 3.68 (s, 2H), 3.57 (s, 2H), 2.42 – 2.30 (m, 4H), 1.68 (d, *J* = 6.2 Hz, 4H), 1.27 (d, *J* = 6.0 Hz, 6H).

^{13}C{^{1}H} NMR (101 MHz, DMSO-d_6) δ 168.5, 159.5, 158.8 (d, $J_{C\text{-}F}$ = 233.8 Hz), 136.2, 136.0, 129.8, 125.0, 124.4, 120.1, 120.0, 114.9, 106.9 (d, $J_{C\text{-}F}$ = 24.3 Hz), 97.2 (d, $J_{C\text{-}F}$ = 25.5 Hz), 69.3, 62.8, 58.3, 53.2, 49.8, 35.0, 33.6, 21.7.

HRMS (ESI) calculated for formula $C_{26}H_{31}FN_3O_2$ ($[M+H]^+$) 436.2393, found 436.2395.

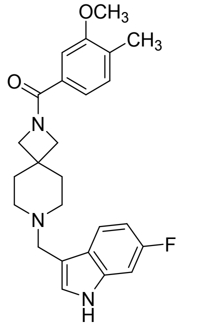

(7-((6-fluoro-1*H*-indol-3-yl)methyl)-2,7-diazaspiro[3.5]nonan-2-yl)(3-methoxy-4-methylphenyl)methanone (25):

The compound was synthesized by following **General Procedure B**. Crude product was purified using the Gilson (Basic, 30x 100 mm column, 15-75% ACN/ 0.05% aqueous NH_4OH, 10 min run). Fractions containing the desired product were concentrated to give **(25)** (8.6 mg, 0.02 mmol, 63% yield) as a clear glass

^{1}H NMR (400 MHz, MeOD-d_4): δ 7.58 (dd, *J* = 8.7, 5.3 Hz, 1H), 7.21 – 7.14 (m, 3H), 7.10 (dd, *J* = 7.6, 1.5 Hz, 1H), 7.04 (dd, *J* = 9.9, 2.3 Hz, 1H), 6.80 (ddd, *J* = 9.7, 8.8, 2.3 Hz, 1H), 4.03 (s, 2H), 3.85 (s,

3H), 3.82 (s, 2H), 3.67 (s, 2H), 2.65 – 2.31 (br. m, 4H), 2.21 (s, 3H), 1.81 (t, *J* = 5.3 Hz, 4H). Note: Indole *NH* not observed.
^{13}C{^{1}H} NMR (101 MHz, MeOD-*d*$_4$): δ 172.3, 161.1 (d, J_{C-F} = 235.2 Hz), 159.2, 137.8 (d, J_{C-F} = 12.5 Hz), 132.8, 131.6, 131.3, 126.5 (d, J_{C-F} = 3.4 Hz), 126.1, 120.9, 120.6 (d, J_{C-F} = 10.1 Hz), 111.4, 110.3, 108.4 (d, J_{C-F} = 24.7 Hz), 98.1 (d, J_{C-F} = 26.0 Hz), 64.5, 59.8, 55.9, 53.9, 51.1, 35.9, 35.1, 16.3.
HRMS (ESI) calculated for formula $C_{25}H_{29}FN_3O_2$ ([M+H]$^+$) 422.2238, found 422.2239.

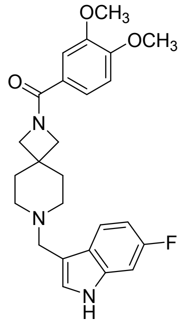

(3,4-dimethoxyphenyl)(7-((6-fluoro-1*H*-indol-3-yl)methyl)-2,7-diazaspiro[3.5]nonan-2-yl)methanone (26):
The compound was synthesized by following **General Procedure B**. Crude product was purified using the Gilson (Basic, 30x 100 mm column, 15-75% ACN/ 0.05% aqueous NH_4OH, 10 min run). Fractions containing the desired product were concentrated to give **(26)** (10.9 mg, 0.02 mmol, 77% yield) as a clear glass.
^{1}H NMR (400 MHz, MeOD-*d*$_4$): δ 7.58 (dd, *J* = 8.7, 5.3 Hz, 1H), 7.27 – 7.22 (m, 2H), 7.19 (s, 1H), 7.04 (dd, *J* = 9.9, 2.3 Hz, 1H), 7.00 – 6.96 (m, 1H), 6.81 (ddd, *J* = 9.7, 8.8, 2.3 Hz, 1H), 4.07 (s, 2H), 3.86 (s, 3H), 3.85 (s, 3H), 3.82 (s, 2H), 3.68 (s, 2H), 2.74 - 2.21 (br. m, 4H), 1.81 (t, *J* = 5.3 Hz, 4H). Note: Indole *NH* not observed.
^{13}C{^{1}H} NMR (101 MHz, MeOD-*d*$_4$): δ 171.8, 161.1 (d, J_{C-F} = 235.1 Hz), 153.2, 150.3, 137.8 (d, J_{C-F} = 12.5 Hz), 126.6 (d, J_{C-F} = 3.4 Hz), 126.4, 126.1, 122.7, 120.7 (d, J_{C-F} = 10.2 Hz), 112.6, 111.9, 111.4, 108.4 (d, J_{C-F} = 24.7 Hz), 98.1 (d, J_{C-F} = 25.9 Hz), 64.7, 59.9, 56.5, 56.4, 53.9, 51.1, 35.9, 35.1.
HRMS (ESI) calculated for formula $C_{25}H_{29}FN_3O_3$ ([M+H]$^+$) 438.2184, found 438.2187.

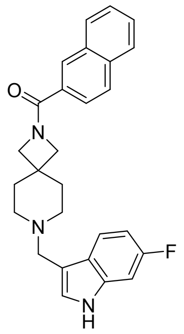

(7-((6-fluoro-1*H*-indol-3-yl)methyl)-2,7-diazaspiro[3.5]nonan-2-yl)(naphthalen-2-yl)methanone (27):

The compound was synthesized by following **General Procedure B**. Crude product was purified using the Gilson (Basic, 30x 100 mm column, 15-75% ACN/ 0.05% aqueous NH_4OH, 10 min run). Fractions containing the desired product were concentrated to give **(27)** (8.4 mg, 0.02 mmol, 60% yield) as a white solid.

^{1}H NMR (400 MHz, MeOD-d_4): δ 8.18 – 8.15 (m, 1H), 7.99 – 7.87 (m, 3H), 7.70 (dd, J = 8.5, 1.7 Hz, 1H), 7.61 – 7.52 (m, 3H), 7.18 (s, 1H), 7.04 (dd, J = 10.0, 2.3 Hz, 1H), 6.80 (ddd, J = 9.7, 8.8, 2.3 Hz, 1H), 4.10 (s, 2H), 3.88 (s, 2H), 3.67 (s, 2H), 2.69 – 2.26 (br. m, 4H), 1.83 (t, J = 5.2 Hz, 4H). Note: Indole *NH* not observed.

^{13}C{^{1}H} NMR (101 MHz, MeOD-d_4): δ 172.3, 161.1 (d, J_{C-F} = 235.2 Hz), 137.8 (d, J_{C-F} = 12.5 Hz), 136.0, 134.0, 131.4, 129.9, 129.4, 129.4, 128.9, 128.8, 127.9, 126.5 (d, J_{C-F} = 3.4 Hz), 126.1, 125.4, 120.7 (d, J_{C-F} = 10.2 Hz), 111.4, 108.4 (d, J_{C-F} = 24.7 Hz), 98.1 (d, J_{C-F} = 25.8 Hz), 64.4, 59.9, 53.9, 51.1, 35.9, 35.1.

HRMS (ESI) calculated for formula $C_{27}H_{27}FN_3O$ ([M+H]$^+$) 428.2131, found 428.2133.

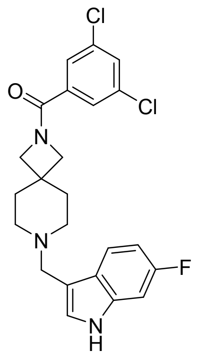

(3,5-dichlorophenyl)(7-[(6-fluoro-1*H*-indol-3-yl)methyl)-2,7-diazaspiro[3.5]nonan-2-yl)methanone (28):

The compound was synthesized by following **General Procedure B**. Crude product was purified using the Gilson (Basic, 30x 100 mm column, 15-75% ACN/ 0.05% aqueous NH_4OH, 10 min run). Fractions containing the desired product were concentrated to give **(28)** (5.2 mg, 0.01 mmol, 36% yield) as a clear glass.

^{1}H NMR (400 MHz, MeOD-d_4): δ 7.60 – 7.55 (m, 4H), 7.18 (s, 1H), 7.04 (dd, J = 10.0, 2.3 Hz, 1H), 6.80 (ddd, J = 9.7, 8.8, 2.3 Hz, 1H), 4.02 (s, 2H), 3.82 (s, 2H), 3.67 (s, 2H), 2.69 – 2.24 (br. m, 4H), 1.81 (t, J = 5.3 Hz, 4H). Note: Indole *NH* not observed.

^{13}C{^{1}H} NMR (101 MHz, MeOD-d_4): δ 169.0, 161.1 (d, J_{C-F} = 235.1 Hz), 137.8 (d, J_{C-F} = 12.5 Hz), 137.5, 136.5, 132.0, 127.5, 126.5 (d, J_{C-F} = 3.4 Hz), 126.1, 120.7 (d, J_{C-F} = 10.2 Hz), 111.4, 108.4 (d, J_{C-F} = 24.8 Hz), 98.1 (d, J_{C-F} = 26.0 Hz), 64.0, 59.9, 53.9, 51.1, 35.8, 35.2.

HRMS (ESI) calculated for formula $C_{23}H_{23}Cl_2FN_3O$ [M+H]$^+$ 446.1197, found 446.1193.

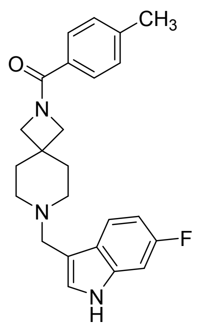

(7-((6-fluoro-1*H*-indol-3-yl)methyl)-2,7-diazaspiro[3.5]nonan-2-yl)(*p*-tolyl)methanone (29):
The compound was synthesized by following **General Procedure B**. Crude product was purified using the Gilson (Basic, 30x 100 mm column, 15-75% ACN/ 0.05% aqueous NH_4OH, 10 min run). Fractions containing the desired product were concentrated to give **(29)** (8.3 mg, 0.02 mmol, 65% yield) as a clear glass.
^{1}H NMR (400 MHz, MeOD-d_4): δ 7.58 (dd, J = 8.7, 5.3 Hz, 1H), 7.55 – 7.50 (m, 2H), 7.29 – 7.23 (m, 2H), 7.19 (s, 1H), 7.04 (dd, J = 9.9, 2.3 Hz, 1H), 6.81 (ddd, J = 9.7, 8.8, 2.3 Hz, 1H), 4.02 (s, 2H), 3.82 (s, 2H), 3.68 (s, 2H), 2.64 – 2.28 (br. m, 4H), 2.37 (s, 3H), 1.81 (t, J = 5.3 Hz, 4H). Note: Indole *NH* not observed.
^{13}C{^{1}H} NMR (101 MHz, MeOD-d_4): δ 172.3, 161.1 (d, $J_{C\text{-}F}$ = 235.2 Hz), 143.1, 137.8 (d, $J_{C\text{-}F}$ = 12.7 Hz), 131.2, 130.2, 129.0, 126.6 (d, $J_{C\text{-}F}$ = 3.4 Hz), 126.1, 120.6 (d, $J_{C\text{-}F}$ = 10.2 Hz), 111.3, 108.4 (d, $J_{C\text{-}F}$ = 24.7 Hz), 98.1 (d, $J_{C\text{-}F}$ = 26.0 Hz), 64.4, 59.7, 53.9, 51.1, 35.9, 35.0, 21.4.
HRMS (ESI) calculated for formula $C_{24}H_{27}FN_3O$ ($[M+H]^+$) 392.2132, found 392.2133.

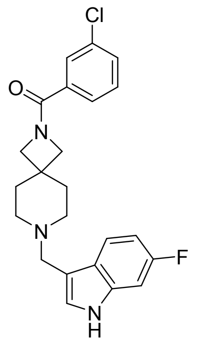

(3-chlorophenyl)(7-[(6-fluoro-1*H*-indol-3-yl)methyl)-2,7-diazaspiro[3.5]nonan-2-yl)methanone (30):
The compound was synthesized by following **General Procedure B**. Crude product was purified using the Gilson (Basic, 30x 100 mm column, 15-75% ACN/ 0.05% aqueous NH_4OH, 10 min run). Fractions containing the desired product were concentrated to give **(30)** (6.7 mg, 0.02 mmol, 50% yield) as a white solid.
^{1}H NMR (400 MHz, MeOD-d_4): δ 7.65 (m, 1H), 7.61 – 7.54 (m, 2H), 7.52 (ddd, J = 8.1, 2.1, 1.2 Hz, 1H), 7.44 (m, 1H), 7.19 (s, 1H), 7.04 (dd, J = 9.9, 2.3 Hz, 1H), 6.81 (ddd, J = 9.7, 8.8, 2.3 Hz, 1H), 4.03

(s, 2H), 3.84 (s, 2H), 3.69 (s, 2H), 2.67 – 2.31 (br. m, 4H), 1.82 (t, *J* = 5.3 Hz, 4H). Note: Indole *NH* not observed.

$^{13}C\{^1H\}$ NMR (101 MHz, MeOD-d_4): δ 170.6, 161.1 (d, J_{C-F} = 235.2 Hz), 137.8 (d, J_{C-F} = 12.5 Hz), 136.2, 135.7, 132.3, 131.3, 128.9, 127.2, 126.5 (d, J_{C-F} = 3.3 Hz), 126.1, 120.7 (d, J_{C-F} = 10.2 Hz), 111.4, 108.4 (d, J_{C-F} = 24.7 Hz), 98.1 (d, J_{C-F} = 26.0 Hz), 64.2, 59.8, 53.9, 51.1, 35.9, 35.1.

HRMS (ESI) calculated for formula $C_{23}H_{24}ClFN_3O$ ($[M+H]^+$) 412.1590, found 412.1586.

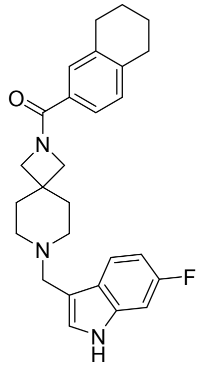

(7-((6-fluoro-1*H*-indol-3-yl)methyl)-2,7-diazaspiro[3.5]nonan-2-yl)(5,6,7,8-tetrahydronaphthalen-2-yl)methanone (31):

The compound was synthesized by following **General Procedure B**. Crude product was purified using the Gilson (Basic, 30x 100 mm column, 15-75% ACN/ 0.05% aqueous NH_4OH, 10 min run). Fractions containing the desired product were concentrated to give **(31)** (8.9 mg, 0.02 mmol, 63% yield) as a clear glass.

^1H NMR (400 MHz, MeOD-d_4): δ 7.58 (dd, *J* = 8.7, 5.3 Hz, 1H), 7.34 – 7.29 (m, 2H), 7.19 (s, 1H), 7.14 – 7.08 (m, 1H), 7.06 – 7.01 (dd, *J* = 10.0, 2.3 Hz, 1H), 6.81 (ddd, *J* = 9.7, 8.8, 2.4 Hz, 1H), 4.01 (s, 2H), 3.81 (s, 2H), 3.68 (s, 2H), 2.83 – 2.73 (m, 4H), 2.68 – 2.24 (br. m, 4H), 1.87 – 1.73 (m, 8H). Note: Indole *NH* not observed.

$^{13}C\{^1H\}$ NMR (101 MHz, MeOD-d_4): δ 172.6, 161.1 (d, J_{C-F} = 235.1 Hz), 142.1, 138.6, 137.8 (d, J_{C-F} = 12.5 Hz), 131.2, 130.2, 129.6, 126.5 (d, J_{C-F} = 3.4 Hz), 126.1, 126.0, 120.6 (d, J_{C-F} = 10.2 Hz), 111.4, 108.4 (d, J_{C-F} = 24.8 Hz), 98.1 (d, J_{C-F} = 26.0 Hz), 64.4, 59.7, 53.9, 51.1, 35.9, 35.0, 30.4, 30.3, 24.2, 24.1.

HRMS (ESI) calculated for formula $C_{27}H_{31}FN_3O$ ($[M+H]^+$) 432.2446, found 432.2446.

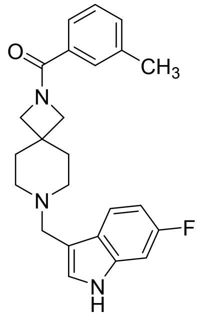

(7-((6-fluoro-1*H*-indol-3-yl)methyl)-2,7-diazaspiro[3.5]nonan-2-yl)(3-methylphenyl)methanone (32):

The compound was synthesized by following **General Procedure B**. Crude product was purified using the Gilson (Basic, 30x 100 mm column, 15-75% ACN/ 0.05% aqueous NH_4OH, 10 min run). Fractions containing the desired product were concentrated to give **(32)** (7.4 mg, 0.02 mmol, 58% yield) as a white solid.

^{1}H NMR (400 MHz, MeOD-d_4): δ 7.59 (dd, J = 8.8, 5.3 Hz, 1H), 7.45 (m, 1H), 7.40 (m, 1H), 7.36 – 7.30 (m, 2H), 7.21 (s, 1H), 7.05 (dd, J = 9.9, 2.3, 1H), 6.82 (ddd, J = 9.7, 8.8, 2.3 Hz, 1H), 4.02 (s, 2H), 3.84 (s, 2H), 3.75 (s, 2H), 2.80 – 2.30 (br. m, 4H), 2.38 (s, 3H), 1.84 (t, J = 5.2 Hz, 4H). Note: Indole -*NH* not observed.

^{13}C{^{1}H} NMR (101 MHz, MeOD-d_4): δ 172.6, 161.2 (d, J_{C-F} = 235.2 Hz), 139.7, 137.8 (d, J_{C-F} = 12.4 Hz), 134.1, 133.0, 129.5, 129.3, 126.8 (d, J_{C-F} = 3.7 Hz), 126.0, 125.9, 120.6 (d, J_{C-F} = 10.2 Hz), 110.9, 108.5 (d, J_{C-F} = 24.8 Hz), 98.2 (d, J_{C-F} = 26.0 Hz), 64.2, 59.7, 53.8, 51.0, 35.7, 34.9, 21.3.

HRMS (ESI) calculated for formula $C_{24}H_{27}FN_3O$ ($[M+H]^+$) 392.2133, found 392.2137.

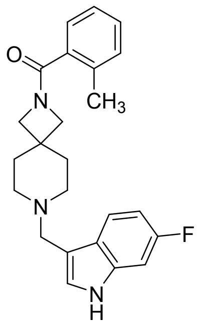

(7-((6-fluoro-1*H*-indol-3-yl)methyl)-2,7-diazaspiro[3.5]nonan-2-yl)(2-methylphenyl)methanone (33):

The compound was synthesized by following **General Procedure A**. Crude product was purified using the Gilson (Basic, 30x 100 mm column, 15-75% ACN/ 0.05% aqueous NH_4OH, 10 min run). Fractions containing the desired product were concentrated to give **(33)** (8.7 mg, 0.02 mmol, 68% yield) as a clear glass.

^{1}H NMR (400 MHz, MeOD-d_4) δ: 7.62 (dd, J = 8.7, 5.2 Hz, 1H), 7.36 – 7.30 (m, 2H), 7.30 – 7.19 (m, 3H), 7.09 (dd, J = 9.8, 2.2 Hz, 1H), 6.87 (m, 1H), 4.02 (s, 2H), 3.88 (s, 2H), 3.66 (s, 2H), 3.00 – 2.59 (m, 4H), 2.34 (s, 3H), 1.99 – 1.83 (m, 4H). Note: Indole *NH* not observed.

^{13}C{^{1}H} NMR (101 MHz, MeOD-d_4) δ: 173.7, 161.3 (d, J_{C-F} = 236.0 Hz), 137.9 (d, J_{C-F} = 12.5 Hz), 136.3, 134.8, 131.9, 131.0, 128.2 (d, J_{C-F} = 3.4 Hz), 127.6, 126.9, 125.7, 120.5 (d, J_{C-F} = 10.1 Hz), 109.1 (d, J_{C-F} = 25.0 Hz), 108.1, 98.5 (d, J_{C-F} = 25.9 Hz), 61.9, 58.7, 53.3, 50.6, 34.5, 34.1, 19.3.

HRMS (ESI) calculated for formula $C_{24}H_{27}FN_3O$ ($[M+H]^+$) 392.2133, found 392.2137.

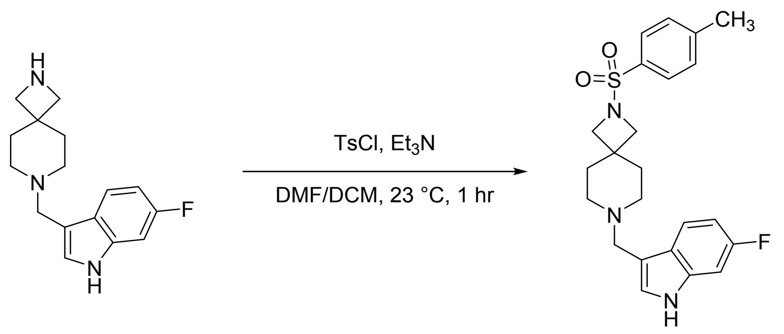

7-((6-fluoro-1*H*-indol-3-yl)methyl)-2-tosyl-2,7-diazaspiro[3.5]nonane (34):
To a test tube was added tosyl chloride (96.4 mg, 0.51 mmol) and DCM (1.5 mL), followed by triethylamine (232 µL, 1.66 mmol). To the mixture was added a solution of **(16)** (91.1 mg, 0.33 mmol) in *N*,*N*-dimethylformamide (540 µL). The solution was stirred at room temperature for 1 hour, then quenched with 0.5 mL aqueous potassium carbonate and diluted with 1 mL of brine. The mixture was extracted with dichloromethane (x3). Organic layers were combined, concentrated, and purified via automated flash column chromatography (ISCO, 0-15% MeOH/DCM, 24g column) to afford **(34)** (13.2 mg, 0.03 mmol, 9% yield) as a clear residue.
^{1}H NMR (400 MHz, MeOD-d_4): δ 7.73 – 7.68 (m, 2H), 7.53 (dd, *J* = 8.8, 5.3 Hz, 1H), 7.48 – 7.42 (m, 2H), 7.15 (s, 1H), 7.02 (dd, *J* = 9.9, 2.3 Hz, 1H), 6.79 (ddd, *J* = 9.7, 8.8, 2.4 Hz, 1H), 3.63 (s, 2H), 3.44 (s, 4H), 2.45 (s, 3H), 2.53 – 2.12 (m, 4H), 1.49 (t, *J* = 5.6 Hz, 4H). Note: Indole *NH* not observed.
^{13}C{^{1}H} NMR (101 MHz, MeOD-d_4): δ 161.1 (d, J_{C-F} = 235.2 Hz), 145.8, 137.8 (d, J_{C-F} = 12.5 Hz), 132.5, 130.9, 129.6, 126.6 (d, J_{C-F} = 3.4 Hz), 126.0, 120.6 (d, J_{C-F} = 10.2 Hz), 111.2, 108.4 (d, J_{C-F} = 24.7 Hz), 98.1 (d, J_{C-F} = 25.9 Hz), 61.4, 53.8, 50.7, 35.7, 33.9, 21.5.
HRMS (ESI) calculated for formula $C_{23}H_{27}FN_3O_2S$ ($[M+H]^+$) 428.1803, found 428.1808.

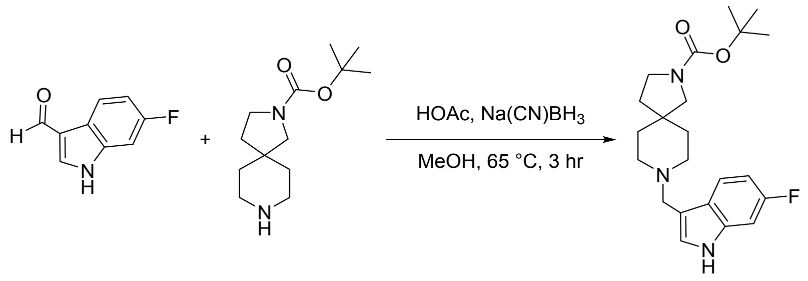

***Tert*-butyl 8-((6-fluoro-1*H*-indol-3-yl)methyl)-2,8-diazaspiro[4.5]decane-2-carboxylate (40):**
In a vial, tert-butyl 2,8-diazaspiro[4.5]decane-2-carboxylate (176.8 mg, 0.74 mmol), and 6-fluoroindole-3-carboxaldehyde (100 mg, 0.61 mmol) were dissolved in methanol (3 mL) followed by the addition of acetic acid (35.1 µL, 0.61 mmol) and sodium cyanoborohydride (77.0 mg, 1.23 mmol). The vial was sealed, and the reaction was heated to 65 ˚C and stirred overnight. The reaction mixture was then cooled to room temperature and made concentrated, before quenching with saturated sodium bicarbonate and extracting with ethyl acetate (x3). Organics were passed through a phase separator, made concentrated, and purified using Teledyne ISCO Combi-flash

system (liquid loading, 40g column, 0-10% MeOH/DCM; elution occurs at 10%) to give **(40)** (113.4 mg, 0.29 mmol, 48% yield) as an off-white solid.
^{1}H NMR (400 MHz, $CDCl_3$): δ 8.67 (d, *J* = 68.3 Hz, 1H), 7.62 (ddd, *J* = 13.7, 8.6, 5.3 Hz, 1H), 7.10 – 7.07 (m, 1H), 7.02 (dd, *J* = 9.7, 2.3 Hz, 1H), 6.88 (td, *J* = 9.2, 2.3 Hz, 1H), 3.71 (s, 2H), 3.35 (dt, *J* = 22.0, 7.1 Hz, 2H), 3.20 (s, 1H), 3.14 (s, 1H), 2.63 – 2.33 (m, 4H), 1.67 (t, *J* = 7.1 Hz, 2H), 1.58 (q, *J* = 5.4, 4.8 Hz, 4H), 1.46 (d, *J* = 2.4 Hz, 9H).
^{13}C{^{1}H} NMR (101 MHz, $CDCl_3$): δ 160.0 (d, $J_{C\text{-}F}$ = 237.4 Hz), 155.0, 136.2 (d, $J_{C\text{-}F}$ = 12.4 Hz), 124.7 (d, $J_{C\text{-}F}$ = 7.8 Hz), 124.3 (d, $J_{C\text{-}F}$ = 16.2 Hz), 120.3, 120.3 (d, $J_{C\text{-}F}$ = 18.5 Hz), 112.6, 112.0, 108.3 (d, $J_{C\text{-}F}$ = 24.4 Hz), 97.5 (d, $J_{C\text{-}F}$ = 25.9 Hz), 79.34, 79.31 53.8, 53.5, 51.0, 50.8, 44.4, 44.1, 40.6, 39.7, 34.8, 28.7.
Note: The higher than expected number of carbon peaks is presumably due to slowly interconverting conformations.
HRMS (ESI) calculated for formula $C_{22}H_{31}FN_3O_2$ ([M+H]$^+$) 388.2395, found 388.2398.

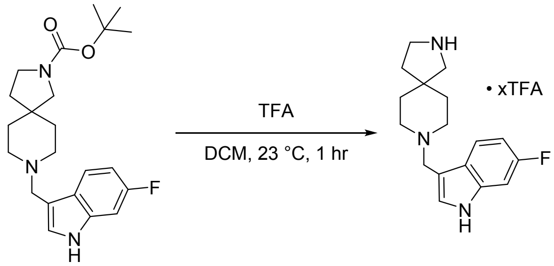

8-((6-fluoro-1*H*-indol-3-yl)methyl)-2,8-diazaspiro[4.5]decane (41):
In a vial, **(40)** (93 mg, 0.24 mmol) in dichloromethane (1mL) was cooled to 0 °C before the addition of trifluoroacetic acid (0.25 mL, 3.26 mmol). The reaction was then warmed to room temperature and stirred for 1 hour. At this time, the reaction was made concentrated and **(41)** was obtained as the trifluoroacetic acid salt (147.8 mg ,0.23 mmol, quant yield) as a red oil.
^{1}H NMR (400 MHz, MeOD-*d*$_4$): δ 11.42 (s, 1H), 7.92 – 7.87 (m, 1H), 7.73 (s, 1H), 7.35 (dd, *J* = 9.7, 2.3 Hz, 1H), 7.17 – 7.11 (m, 1H), 4.71 (s, 2H), 3.74 (d, *J* = 12.3 Hz, 2H), 3.61 (dt, *J* = 22.1, 7.4 Hz, 2H), 3.53 (br. s, 1H), 3.51 (quint, *J* = 1.6 Hz, 1H), 3.46 – 3.22 (m, 2H), 2.28 (t, *J* = 7.5 Hz, 1H), 2.18 – 2.09 (m, 5H). Note: Indole *NH* not observed.
^{13}C{^{1}H} NMR (101 MHz, MeOD-*d*$_4$): δ 162.4 (q, $J_{C\text{-}F}$ = 35.9 Hz), 161.5 (d, $J_{C\text{-}F}$ = 237.0 Hz), 138.0 (d, $J_{C\text{-}F}$ = 12.6 Hz), 130.2, 125.3 (d, $J_{C\text{-}F}$ = 3.5 Hz), 120.3 (d, $J_{C\text{-}F}$ = 10.3 Hz), 110.0 (d, $J_{C\text{-}F}$ = 25.0 Hz), 104.0 (d, $J_{C\text{-}F}$ = 3.7 Hz), 98.9 (d, $J_{C\text{-}F}$ = 26.2 Hz), 56.7, 50.3, 50.1, 45.9, 44.8, 41.0, 38.3.
HRMS (ESI) calculated for formula $C_{17}H_{23}FN_3$ ([M+H]$^+$) 288.1871, found 288.1874.

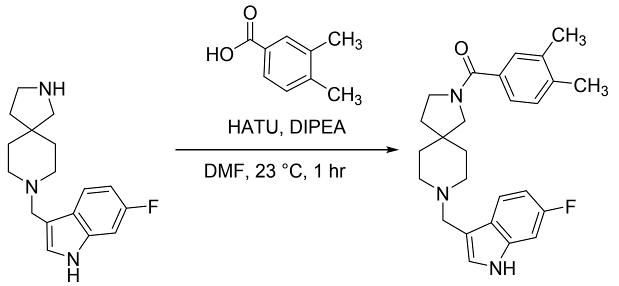

(3,4-dimethylphenyl)(8-[(6-fluoro-1*H*-indol-3-yl)methyl)-2,8-diazaspiro[4.5]decan-2-yl)methanone (42):

To a solution of **(41)** (30.3 mg, 0.05 mmol) and HATU (22.0 mg, 0.06 mmol) in *N,N*-dimethylformamide (0.5 mL) was added *N,N*-diisopropylethylamine (50.3 µL, 0.29 mmol). The reaction mixture was then stirred for 10 minutes before adding to a solution of 3,4-dimethylbenzoic acid (8.7 mg, 0.06 mmol) in *N,N*-dimethylformamide (0.5 mL) and stirring for one hour. Crude product was then purified using the Gilson (Basic, 30x 100 mm column, 5-95% ACN/ 0.05% aqueous NH_4OH, 10 min run). Fractions containing the desired product were concentrated and purified once more via Teledyne ISCO Combi-Flash system (liquid loading, 4G column, 0-10% MeOH/DCM) to give **(42)** (16.5 mg, 0.04 mmol, 82% yield) as a clear glass.

^{1}H NMR (400 MHz, DMSO-d_6): δ 11.03 (br. s, 1H), 7.67 – 7.58 (m, 1H), 7.33 – 7.09 (m, 5H), 6.89 – 6.80 (m, 1H), 3.64 (br. s, 2H), 3.50– 3.41 (m, 3H), 3.29 – 3.22 (m, 3H), 2.56 – 2.37 (m, 2H), 2.23 (s, 6H), 1.70 (dt, J = 21.8, 7.1 Hz, 2H), 1.58 – 1.55 (m, 2H), 1.45 (m, 2H).

^{13}C{^{1}H} NMR (101 MHz, DMSO-d_6): δ 168.7, 168.6, 158.8 (d, $J_{C\text{-}F}$ = 233.7 Hz), 138.1, 138.1, 136.2 (d, $J_{C\text{-}F}$ = 10.6 Hz), 136.0, 134.5, 134.4, 129.1 (d, $J_{C\text{-}F}$ = 9.1 Hz), 128.2, 125.4, 124.5 (d, $J_{C\text{-}F}$ = 9.5 Hz), 124.4 (d, $J_{C\text{-}F}$ = 10.1 Hz), 120.1 (d, $J_{C\text{-}F}$ = 10.1 Hz), 107.0, 97.3 (d, $J_{C\text{-}F}$ = 25.2 Hz), 58.9, 53.5, 49.9, 47.1, 44.1, 33.7, 19.3.

Note: The higher than expected number of carbon peaks is presumably due to slowly interconverting conformations.

HRMS (ESI) calculated for formula $C_{26}H_{31}FN_3O$ ($[M+H]^+$) 420.2446, found 420.2453.

% Inhibition at $D_{4.4}$ was found to be 44% at 10 mM when screened at Eurofins.

References:

(1) Zhang, Chen; Huang, Anbang; Ye, Fei; Huang, Longbin; Huang, Zhenggang; Wang, Jianmin; Wei, Yonggang; Yan, Pangke; Zheng, Wei. Peptide Amide Compound and Preparation Method and Medical Use Thereof. EP3656782A1, May 27, 2020.

(2) Guillon, R.; Logé, C.; Pagniez, F.; Ferchaud-Roucher, V.; Duflos, M.; Picot, C.; Pape, P. L. Synthesis and *in Vitro* Antifungal Evaluation of 2-(2,4-Difluorophenyl)-1-[(1*H*-indol-3-ylmethyl)methylamino]-3-(1*H*-1,2,4-triazol-1-yl)propan-2-ols. *J. Enzyme Inhib. Med. Chem.* **2011**, *26* (2), 261–269. https://doi.org/10.3109/14756366.2010.503607.

III. NMR Spectra

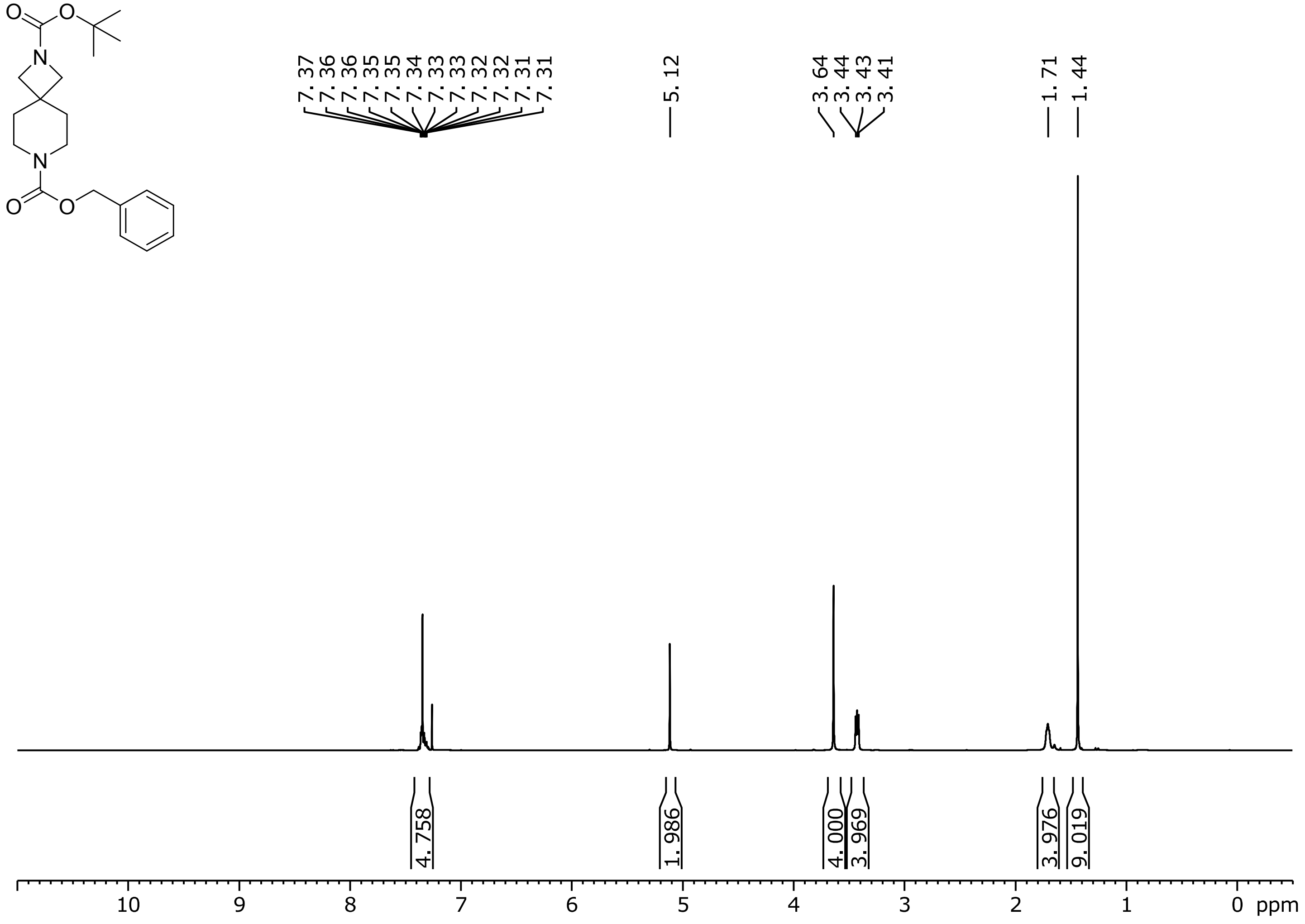

Figure S1: ^{1}H NMR spectra of **1** in $CDCl_3$.

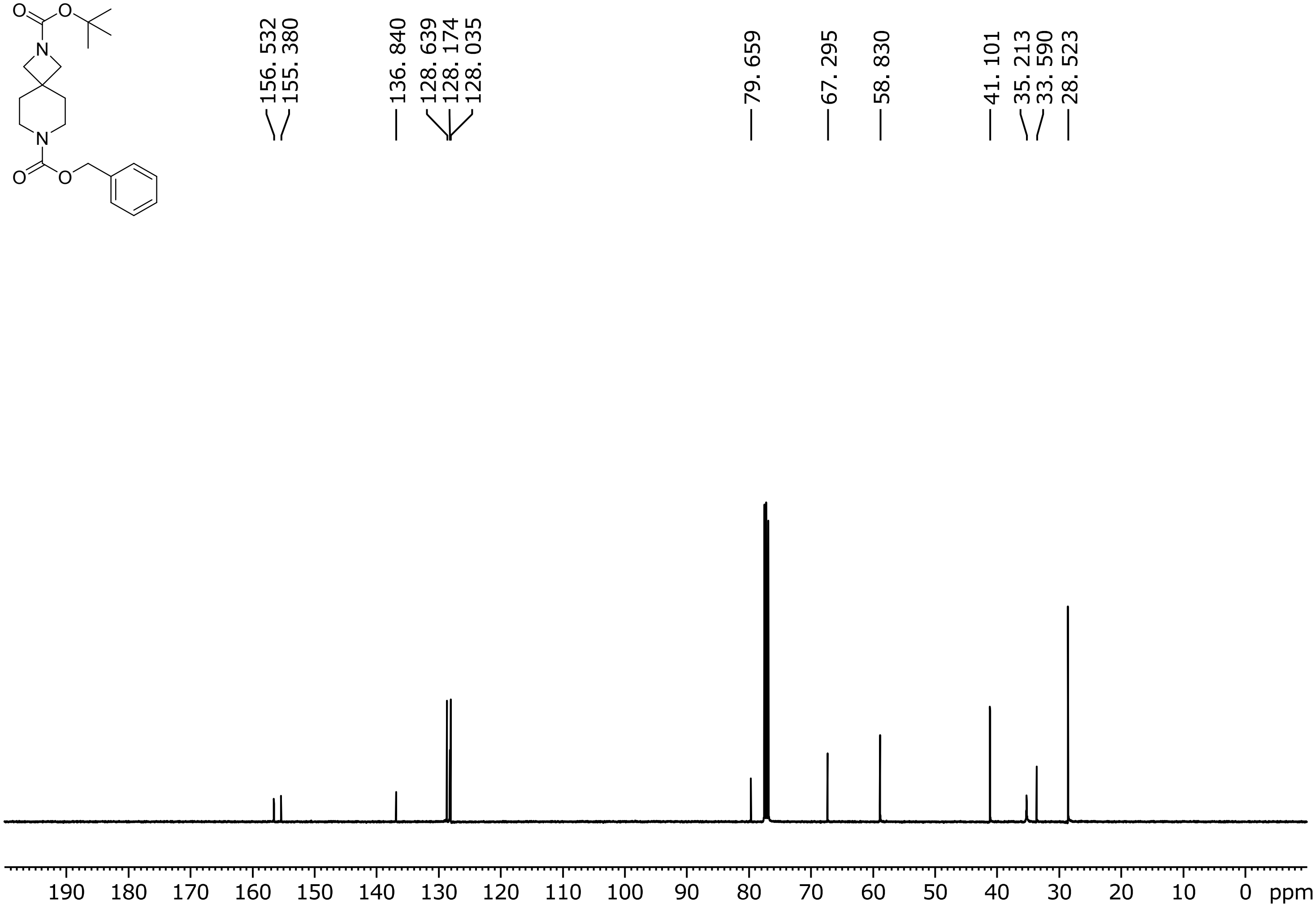

Figure S2: ^{13}C NMR spectra of **1** in $CDCl_3$.

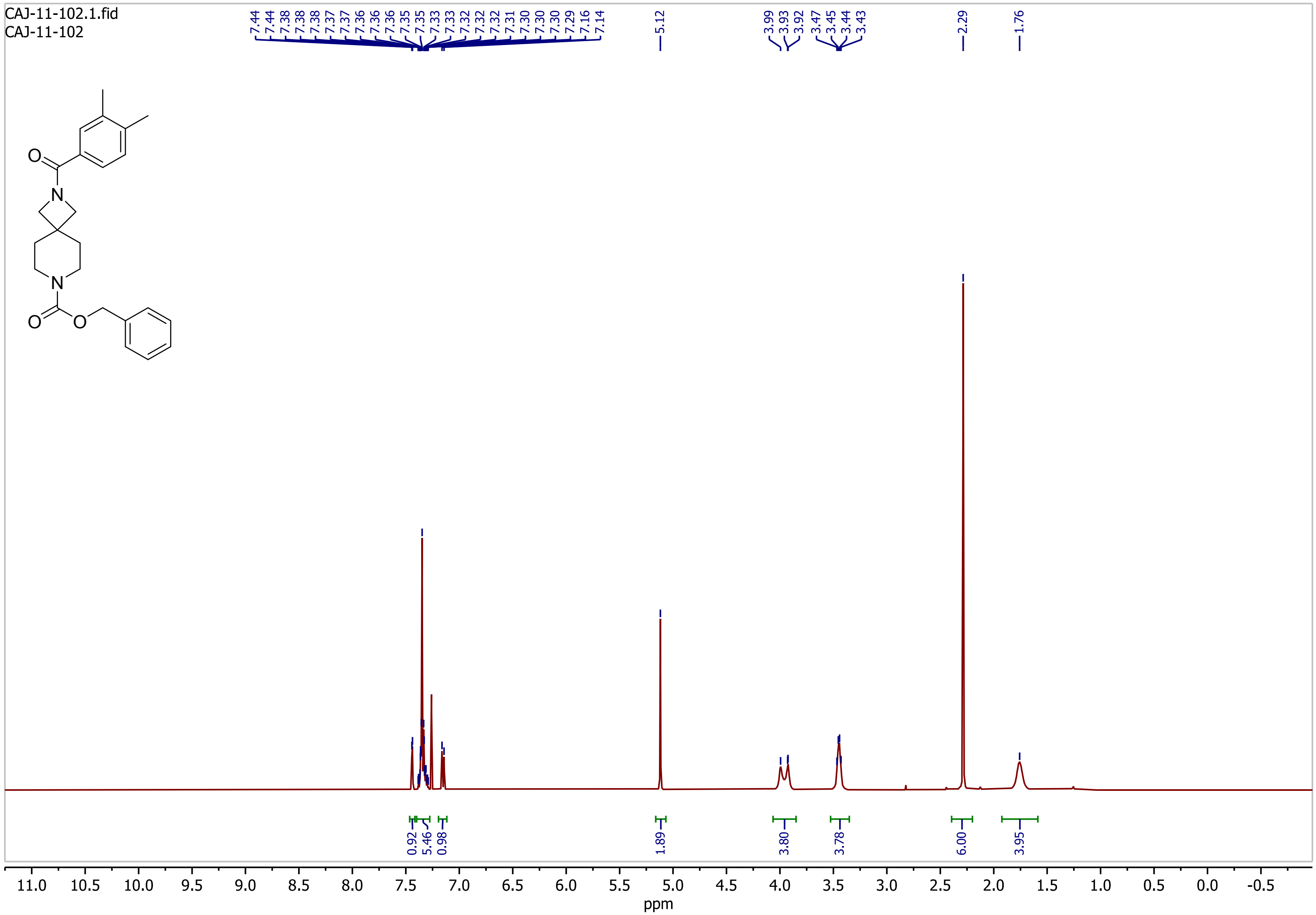

Figure S3: ^{1}H NMR spectra of **2** in $CDCl_3$.

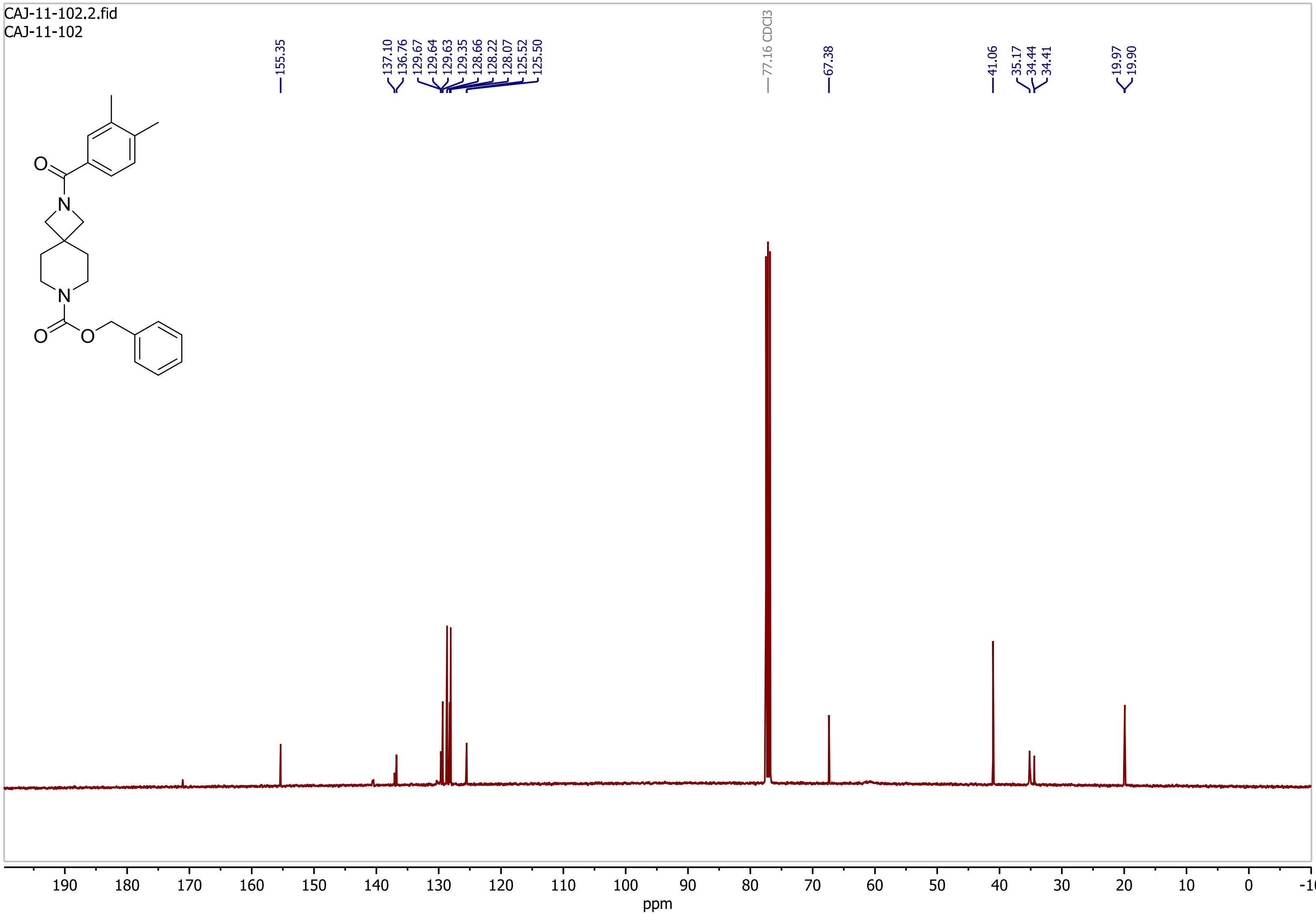

Figure S4: ^{13}C NMR spectra of **2** in $CDCl_3$.

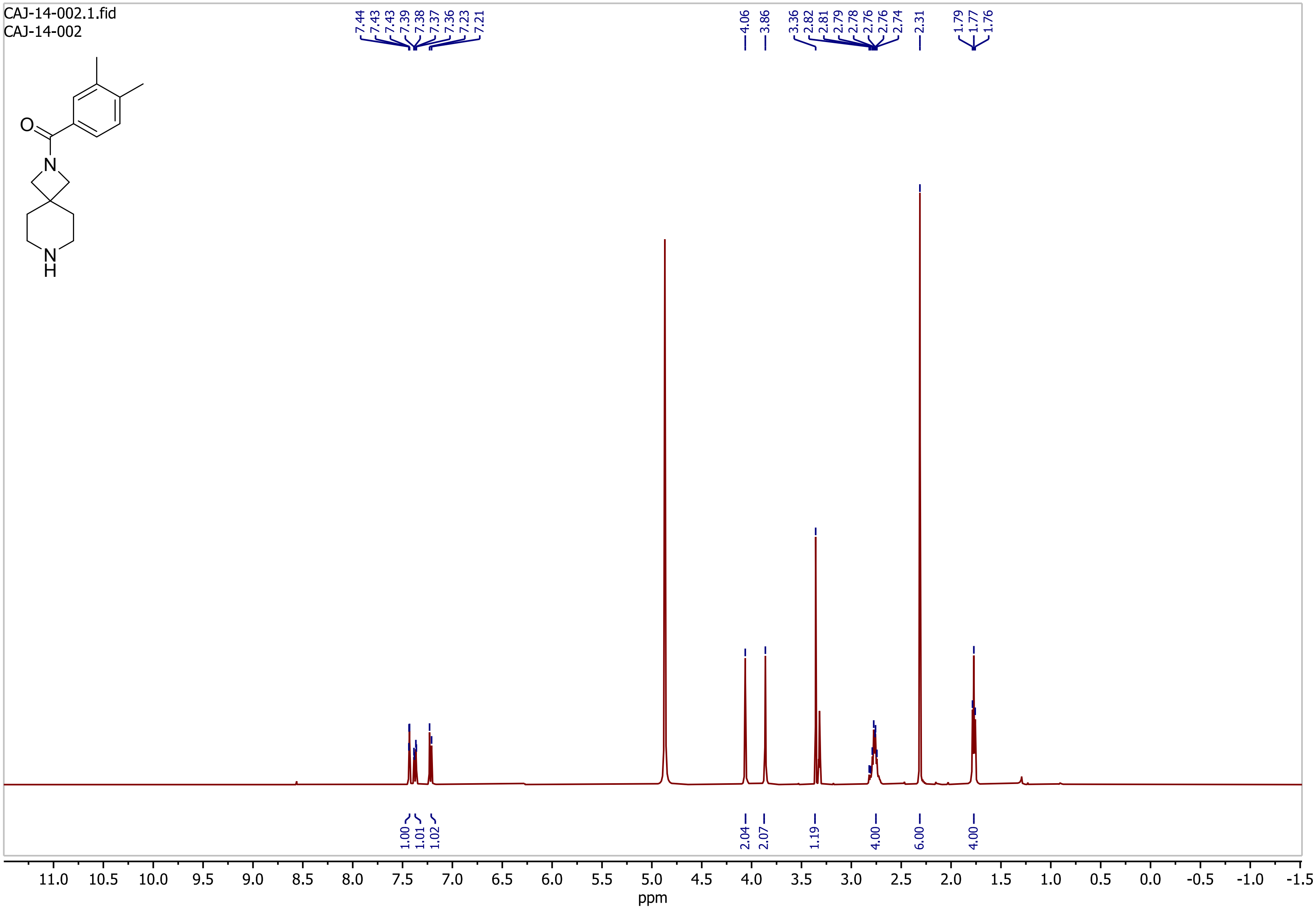

Figure S5: ^{1}H NMR spectra of **3** in MeOD-d_4.

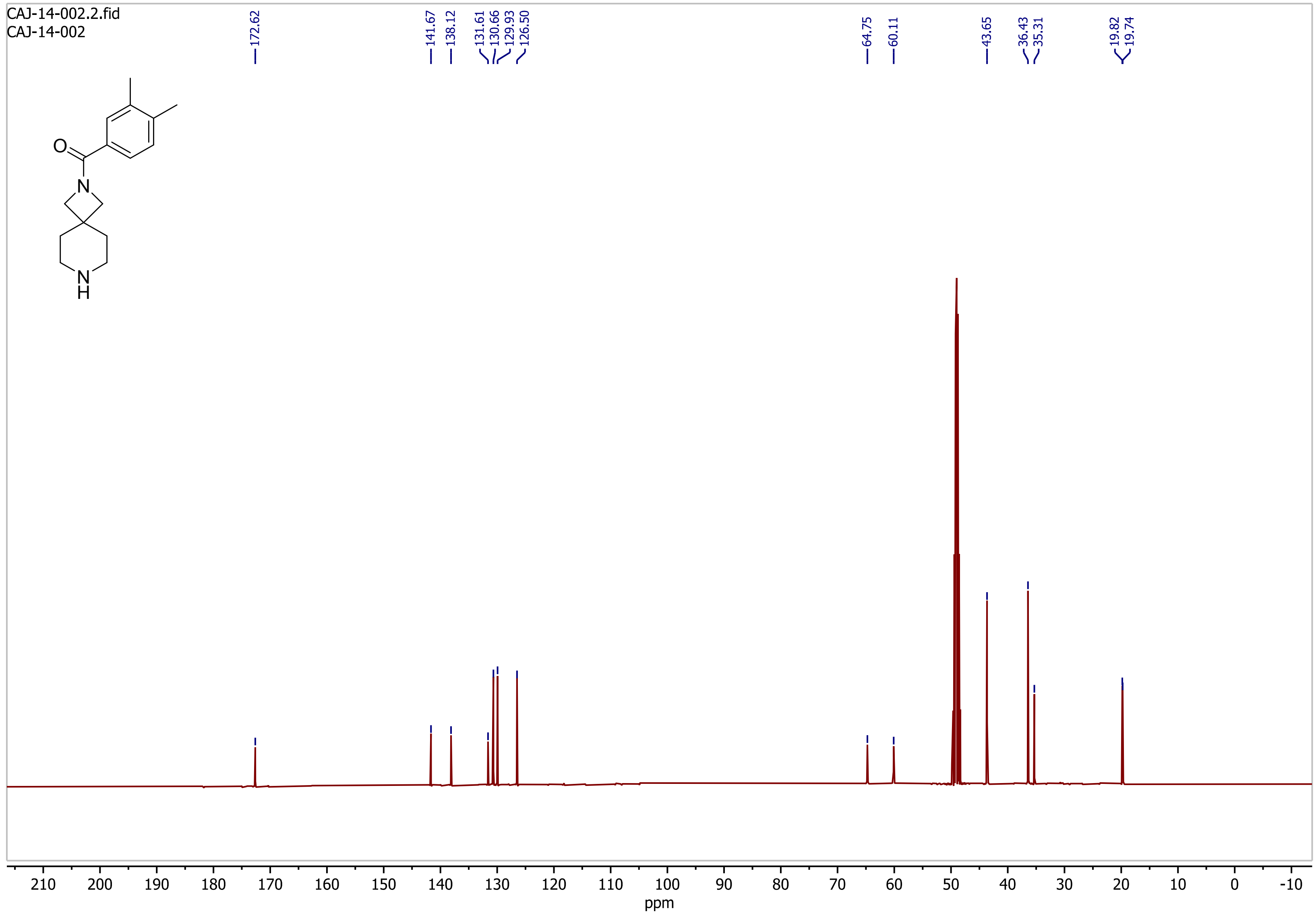

Figure S6: ^{13}C NMR spectra of **3** in MeOD-d_4.

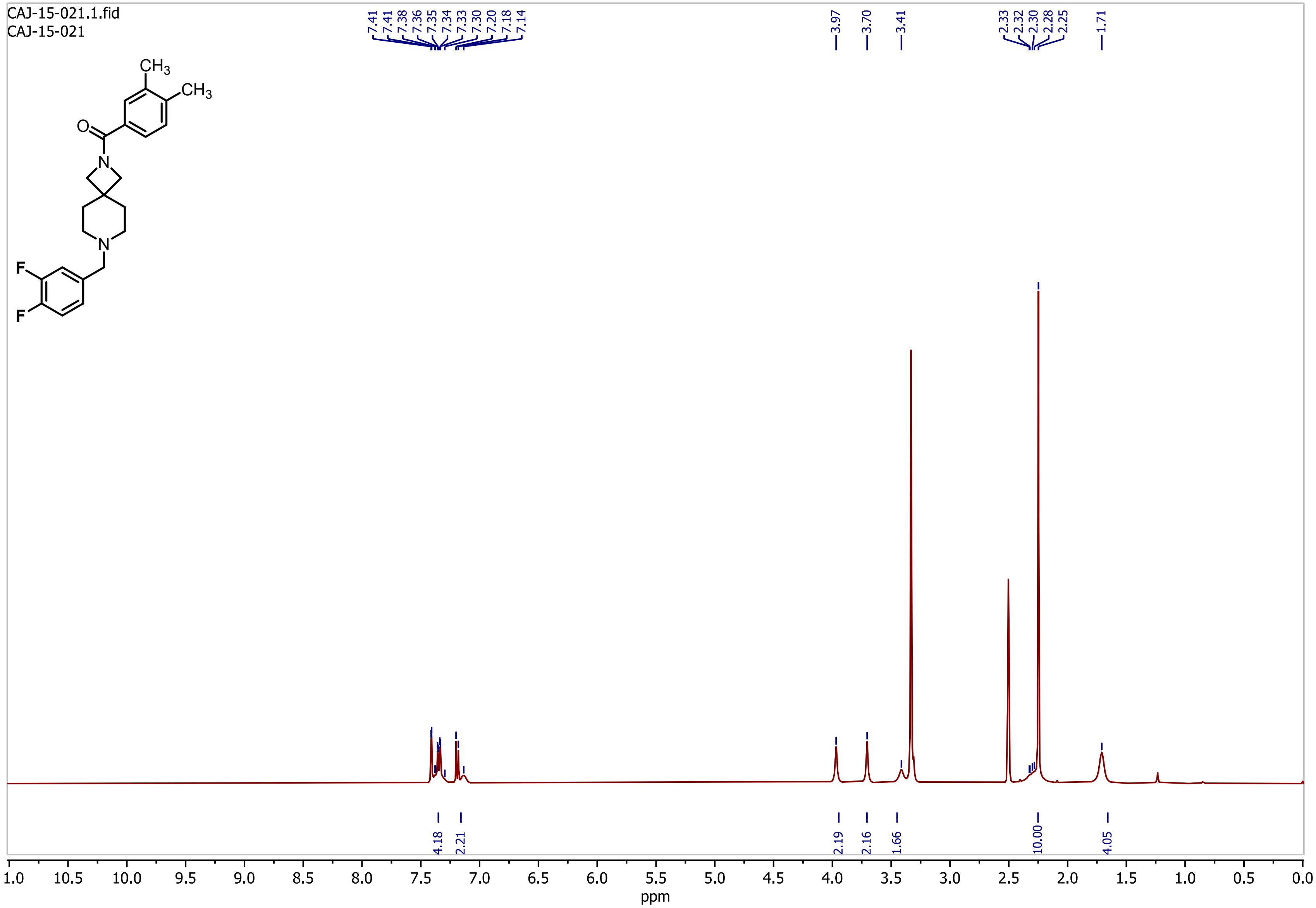

Figure S7: ^{1}H NMR spectrum of **4** in DMSO-d_6.

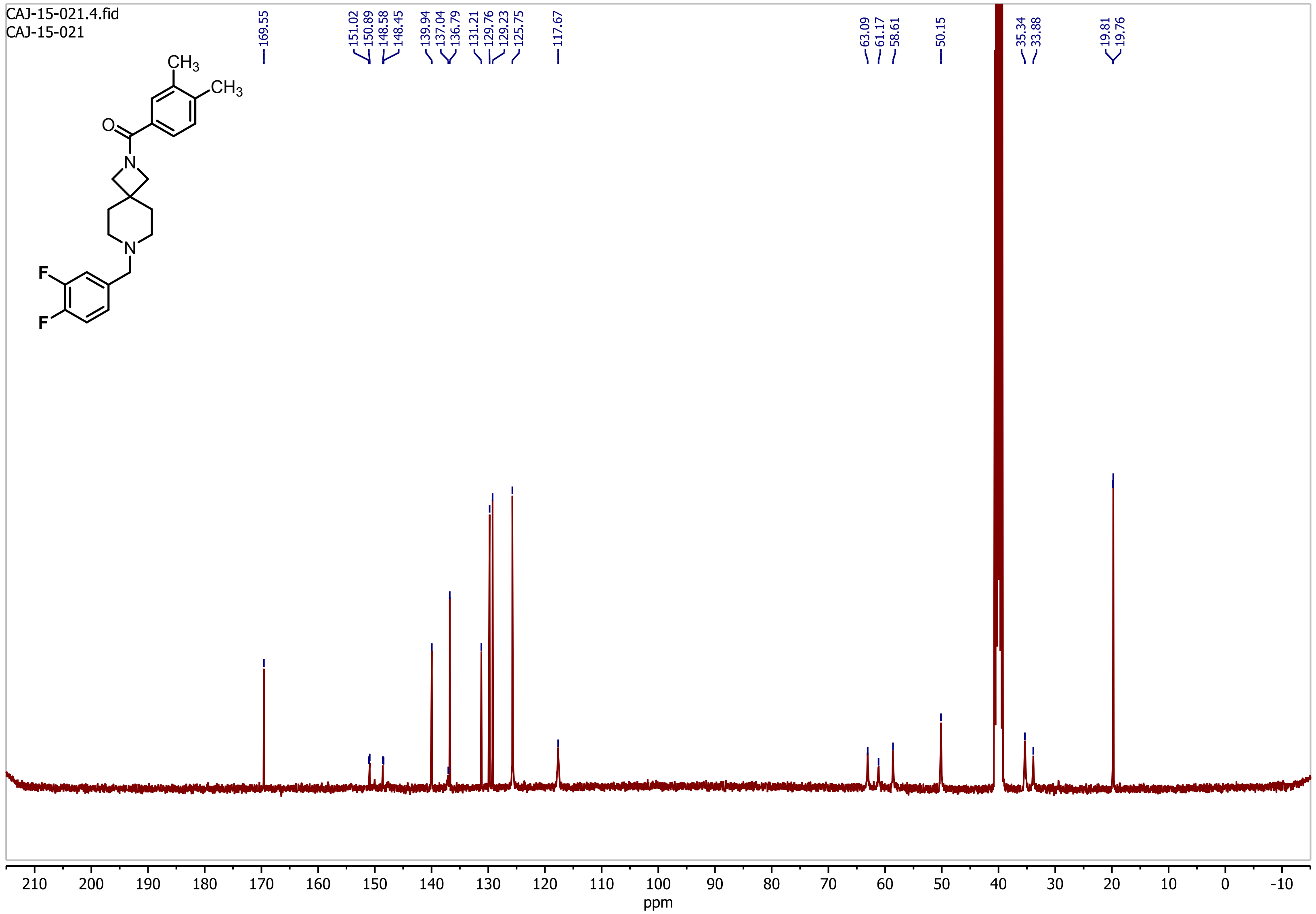

Figure S8: ^{13}C NMR spectrum of **4** in DMSO-d_6.

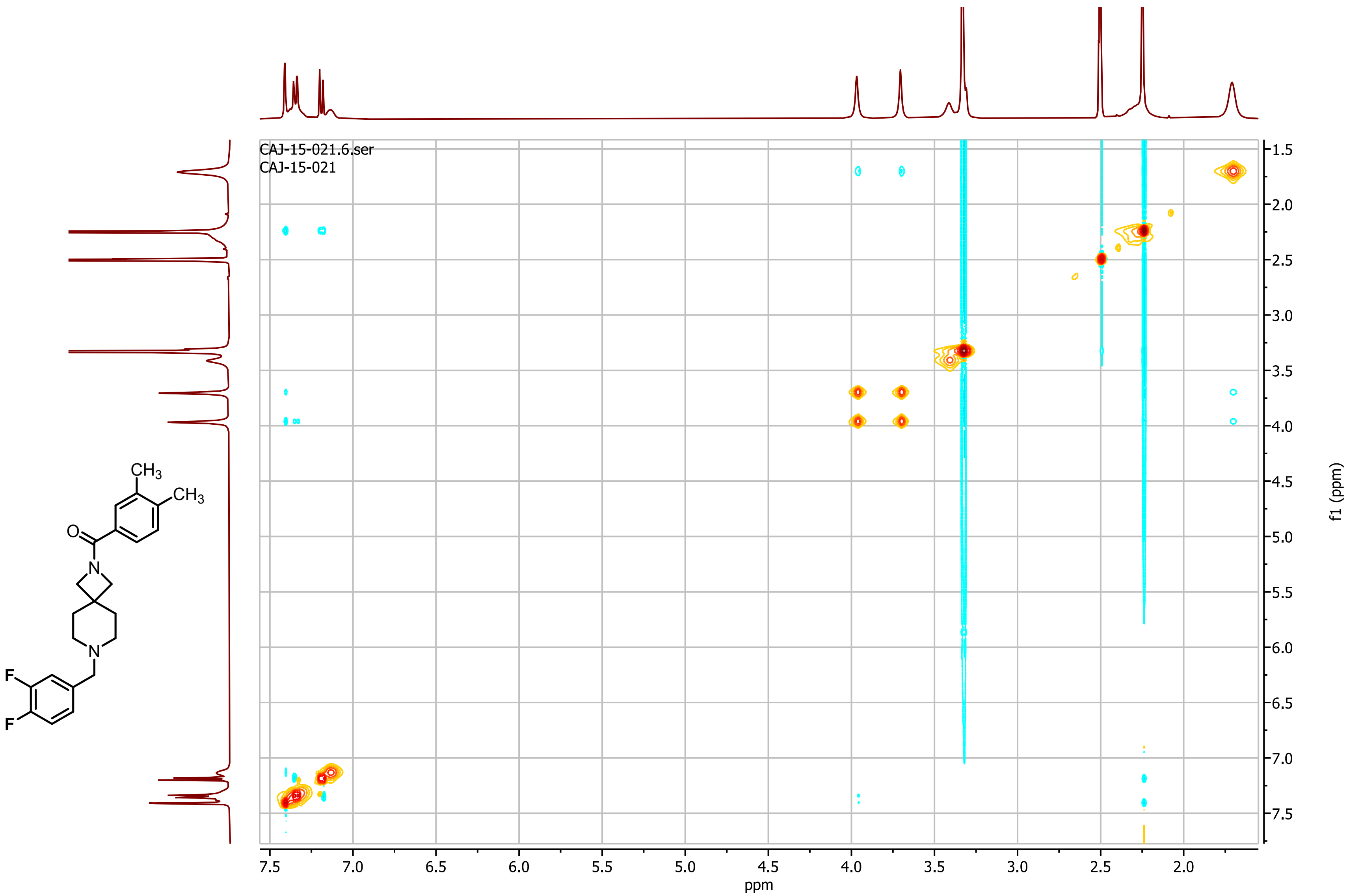

Figure S9: NOESY of **4** in DMSO-d_6.

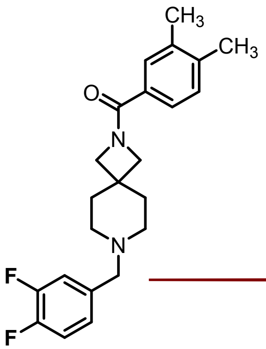

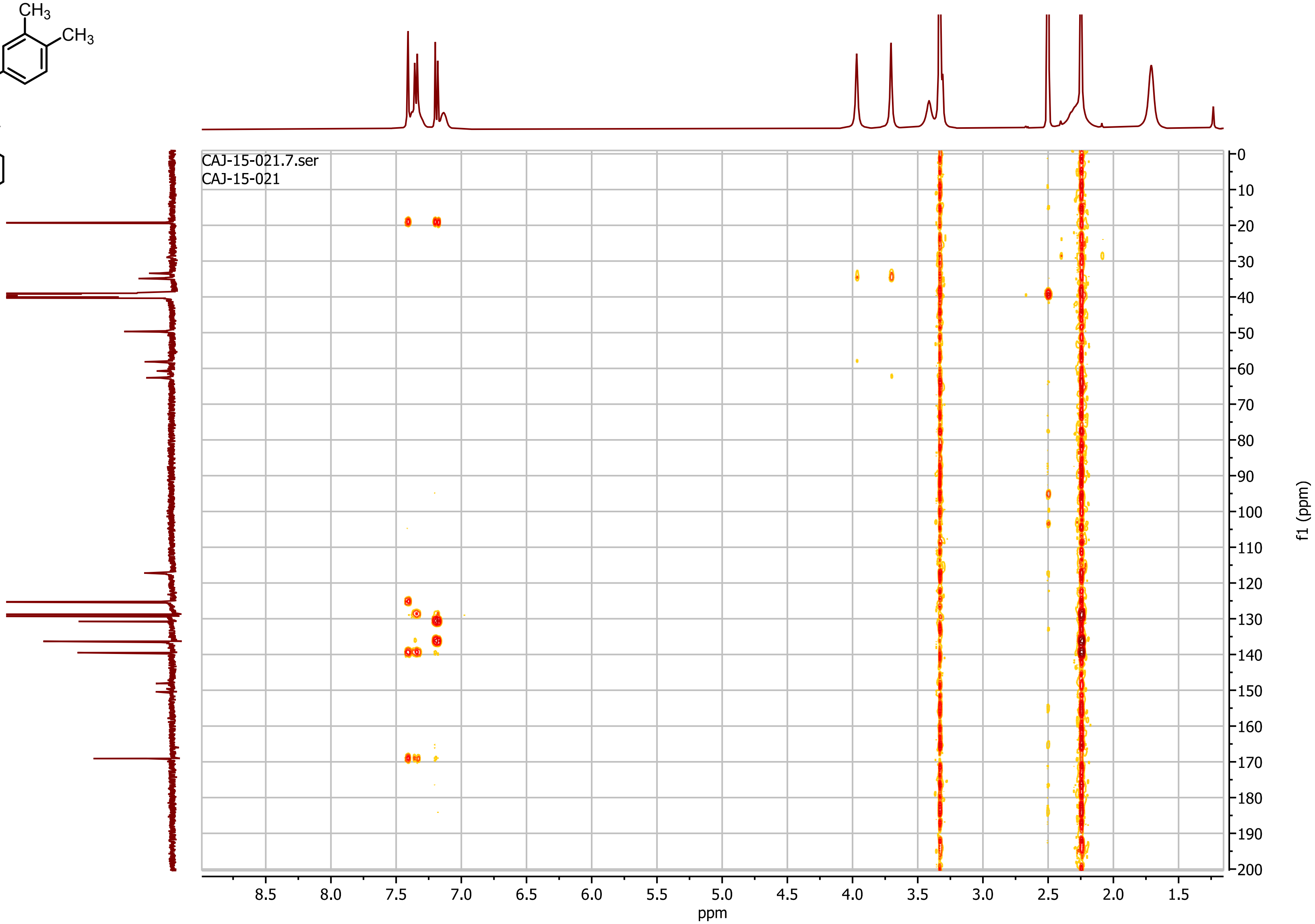

Figure S10: HMBC of **4** in DMSO-d_6.

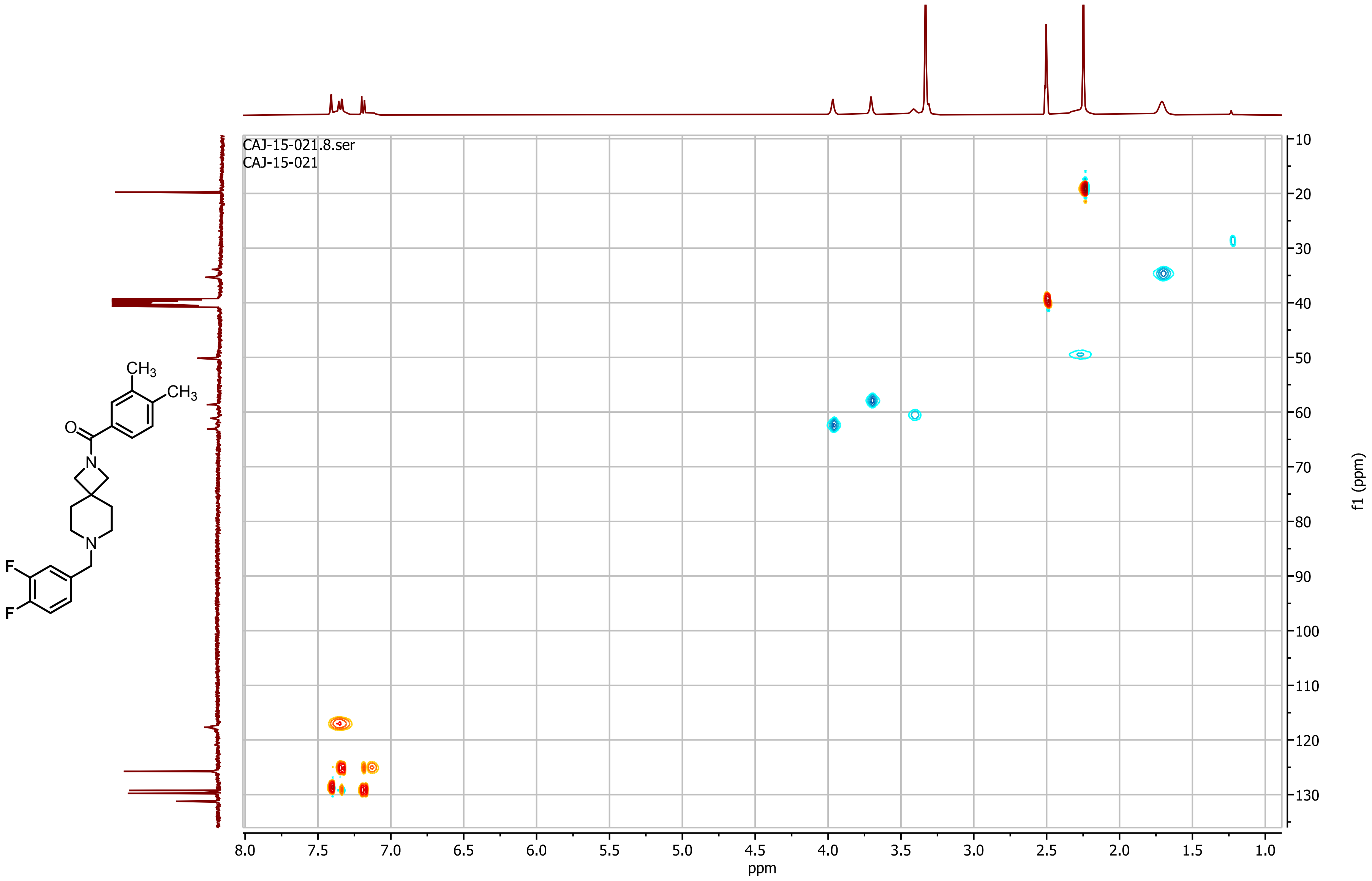

Figure S11: HSQC of **4** in DMSO-d_6.

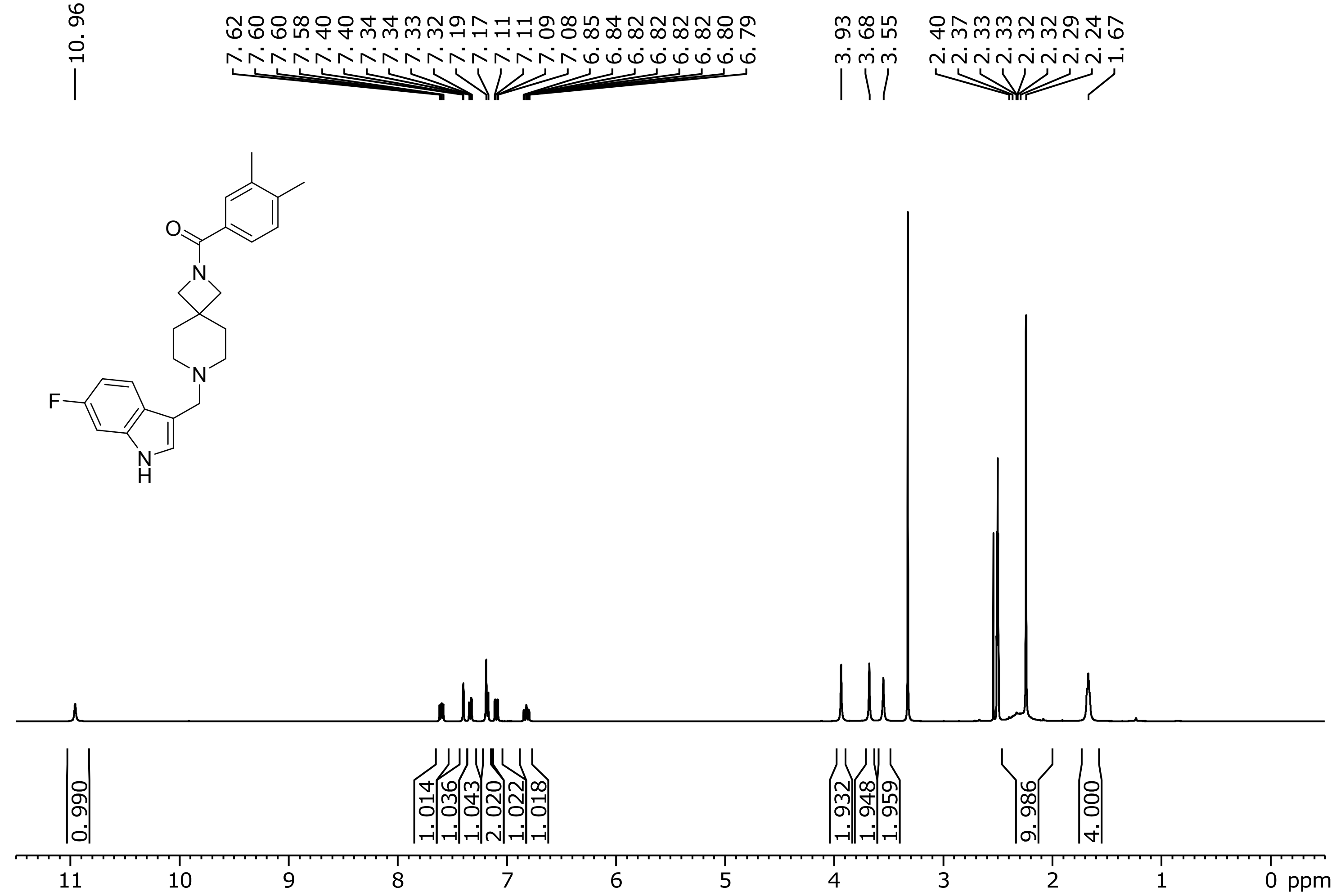

Figure S12: ^{1}H NMR spectra of **5** in DMSO-d_6.

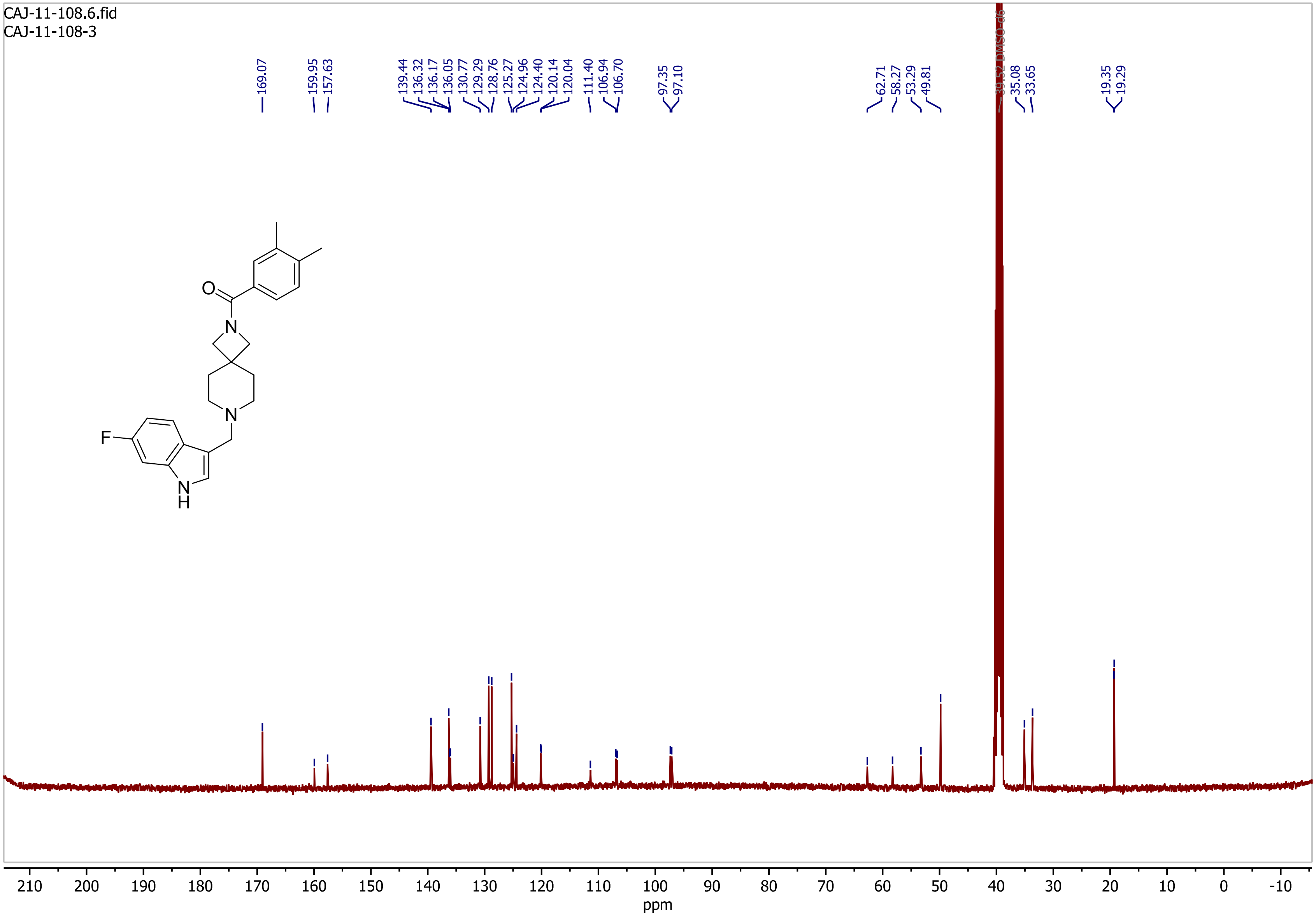

Figure S13: ^{13}C NMR spectra of **5** in DMSO-d_6.

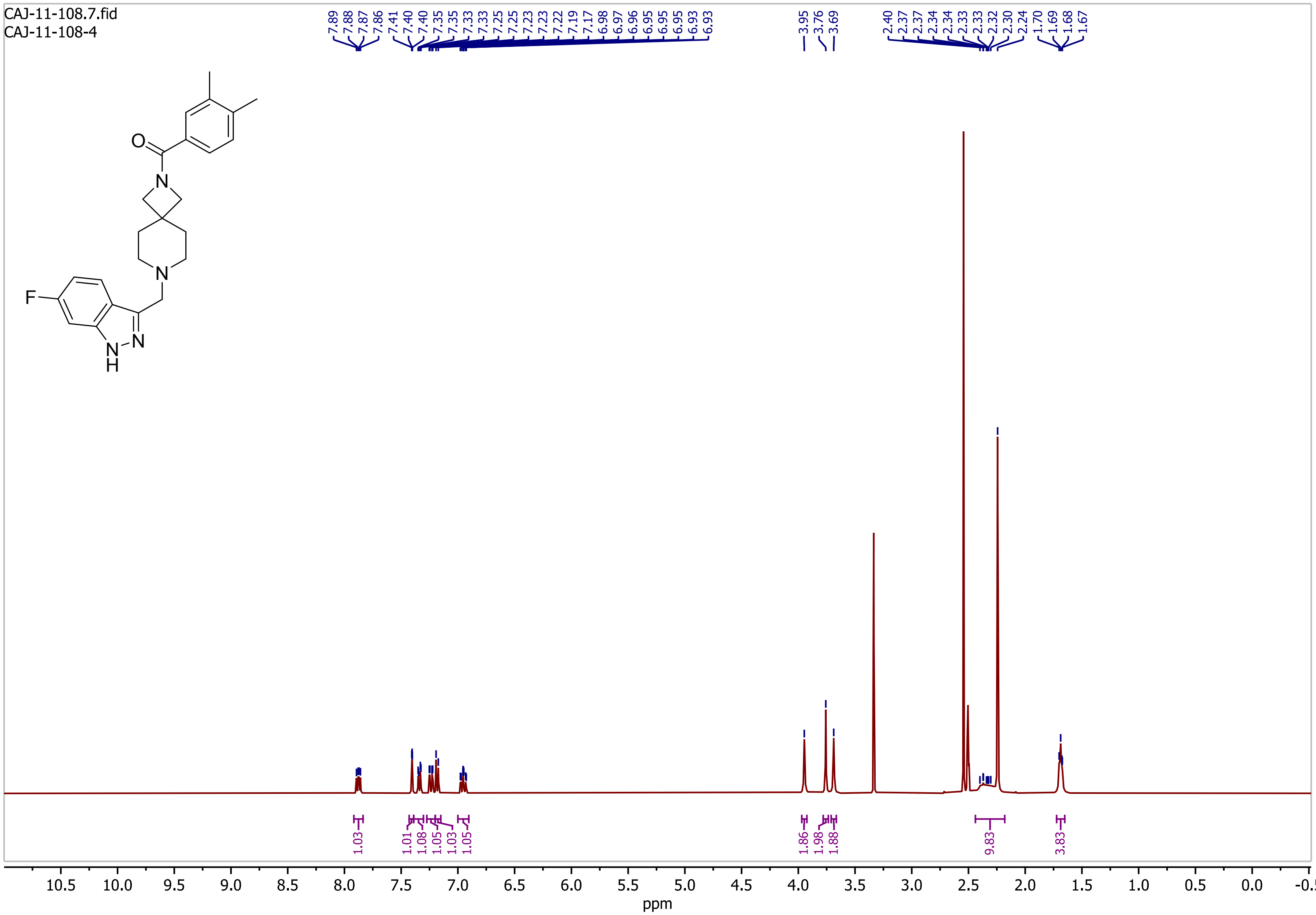

Figure S14: ^{1}H NMR spectra of **6** in DMSO-d_6.

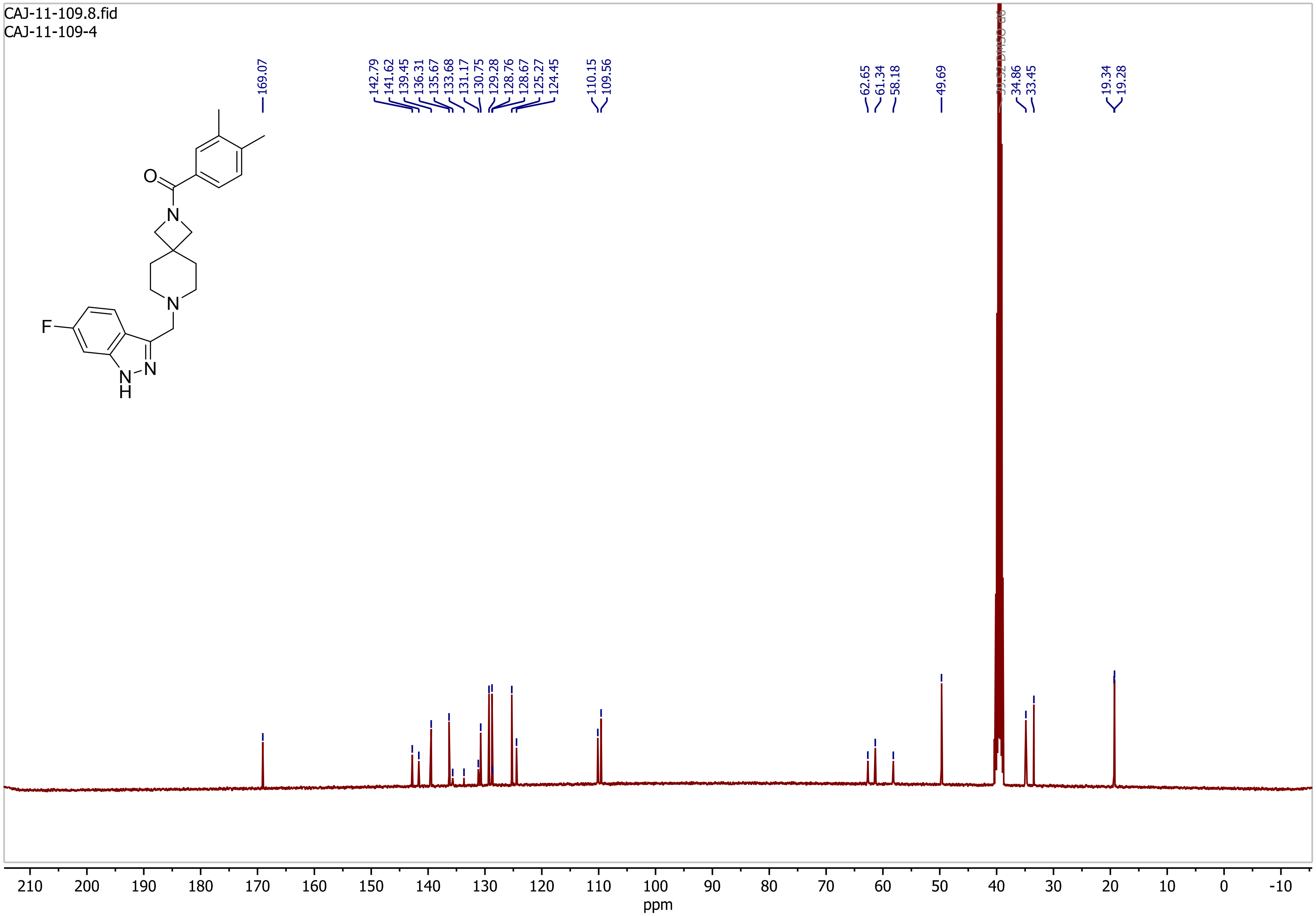

Figure S15: ^{13}C NMR spectra of **6** in DMSO-d_6.

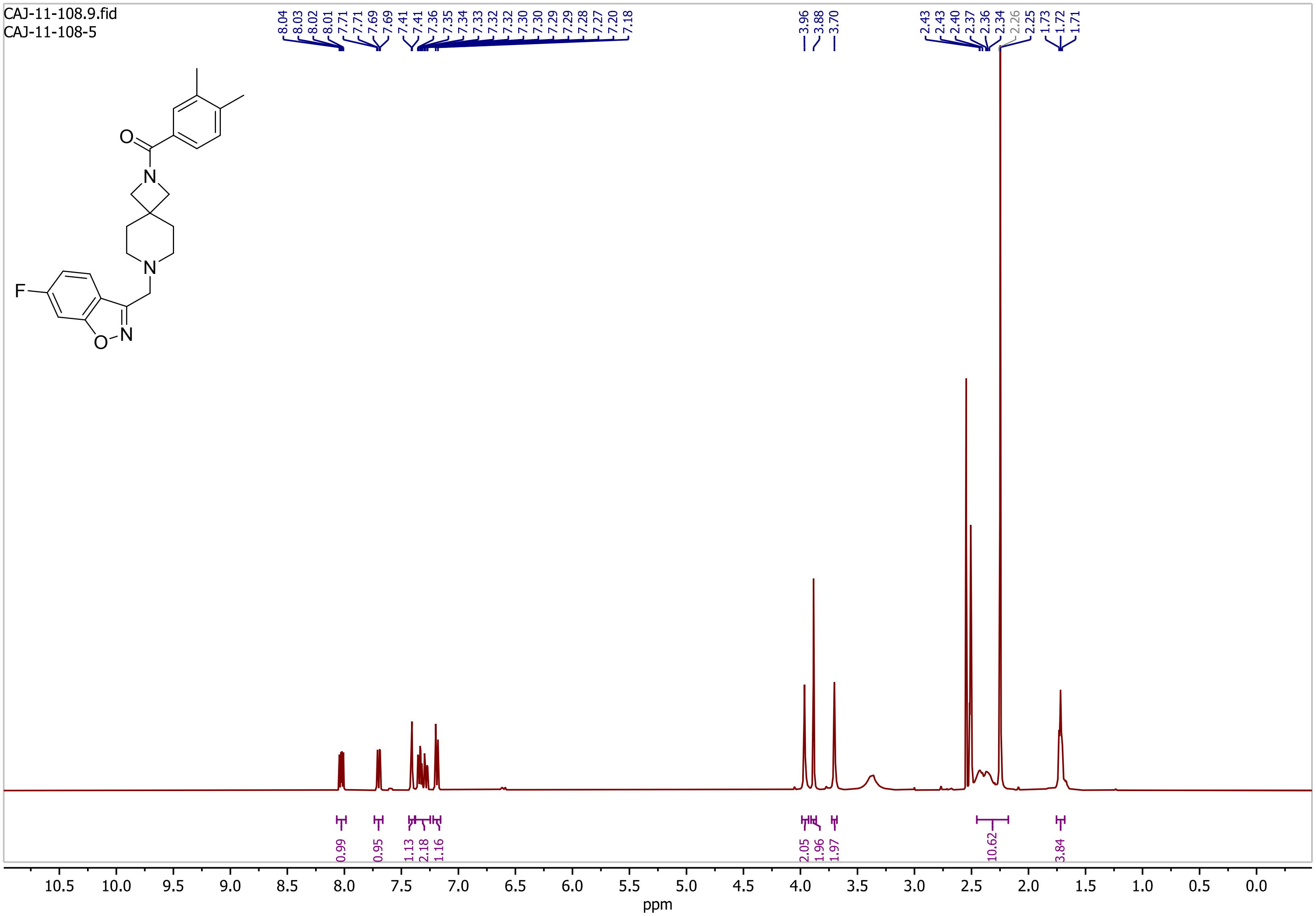

Figure S16: ^{1}H NMR spectra of **7** in DMSO-d_6.

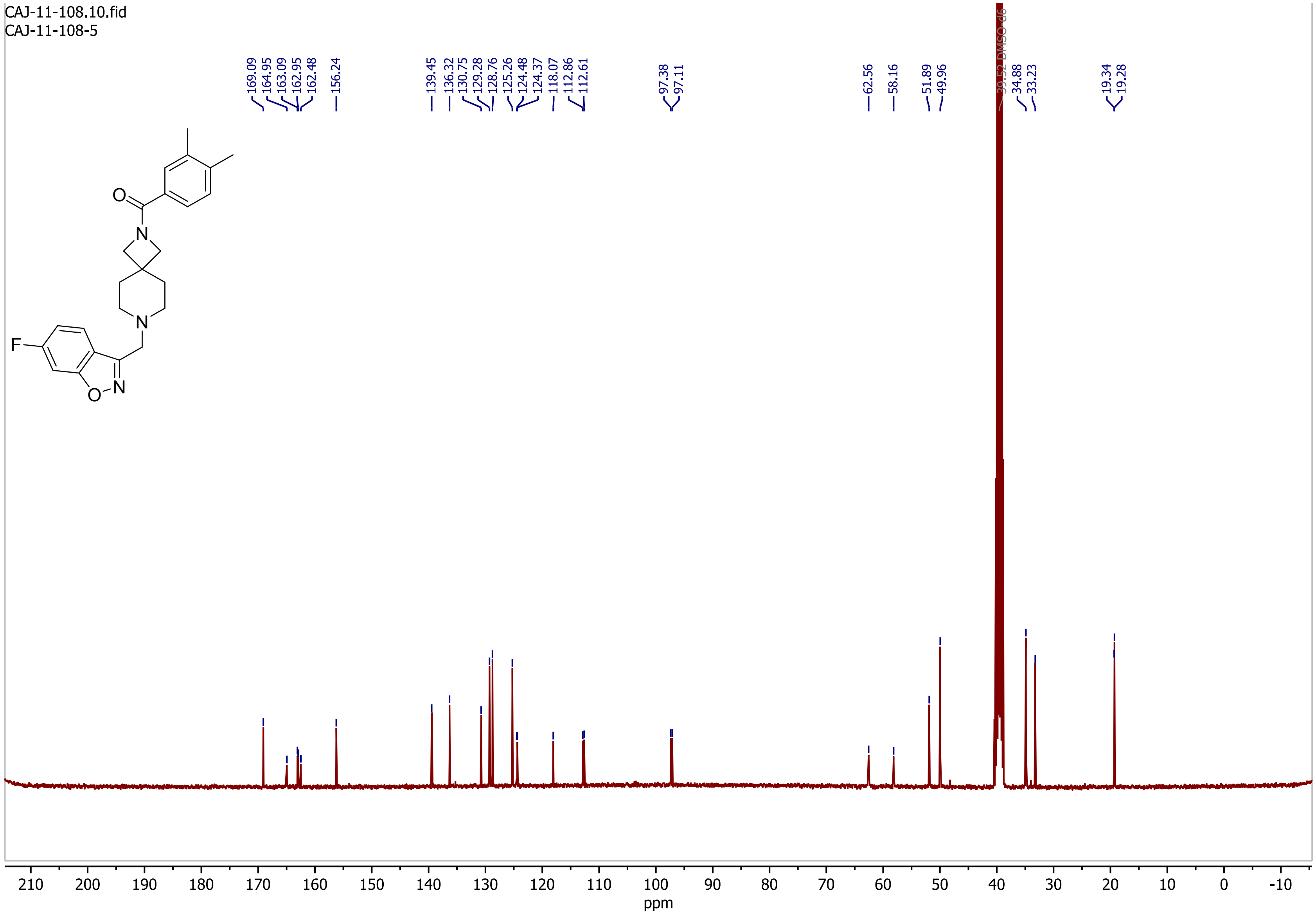

Figure S17: ^{13}C NMR spectra of **7** in DMSO-d_6.

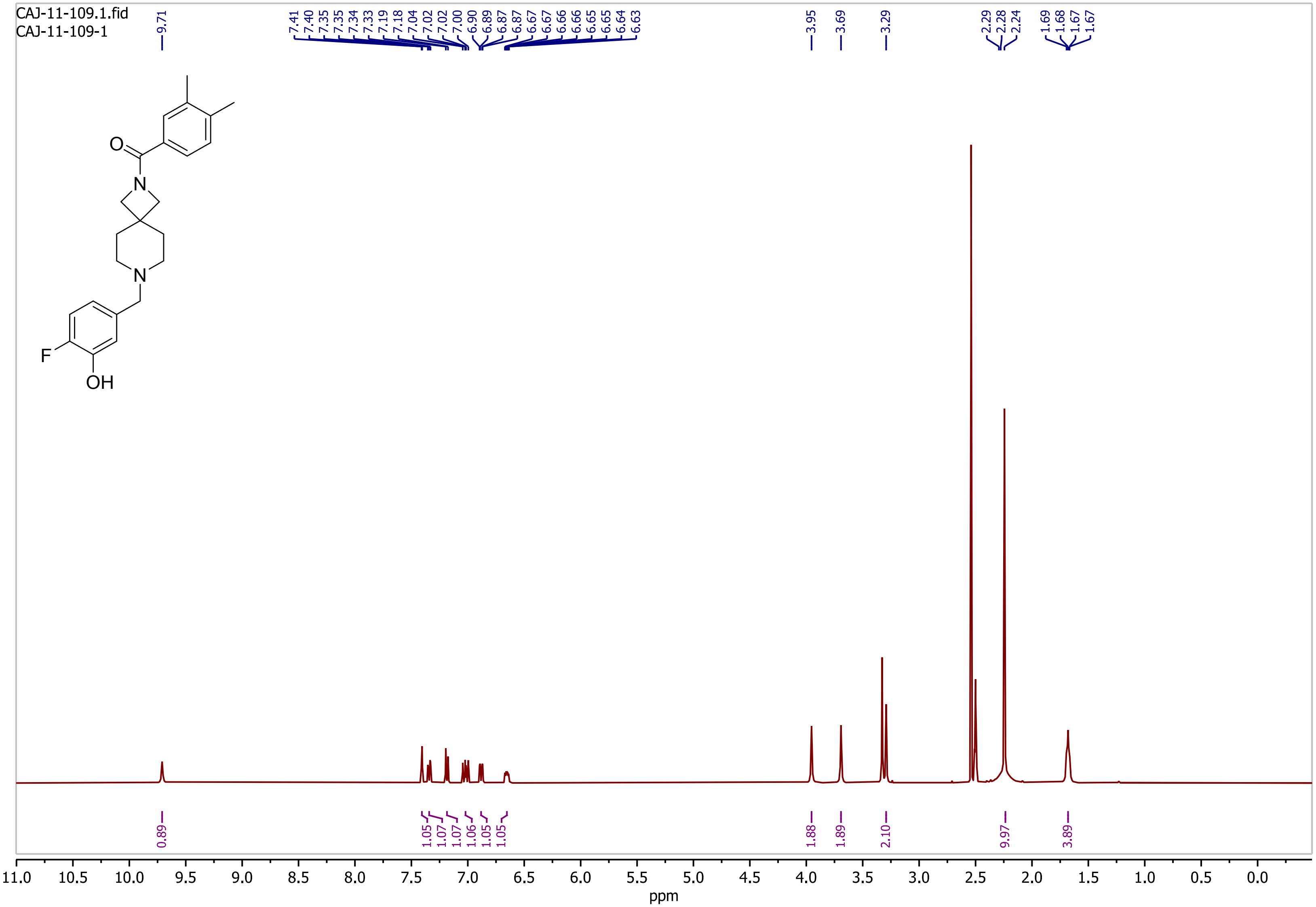

Figure S18: ^{1}H NMR spectra of **8** in DMSO-d_6.

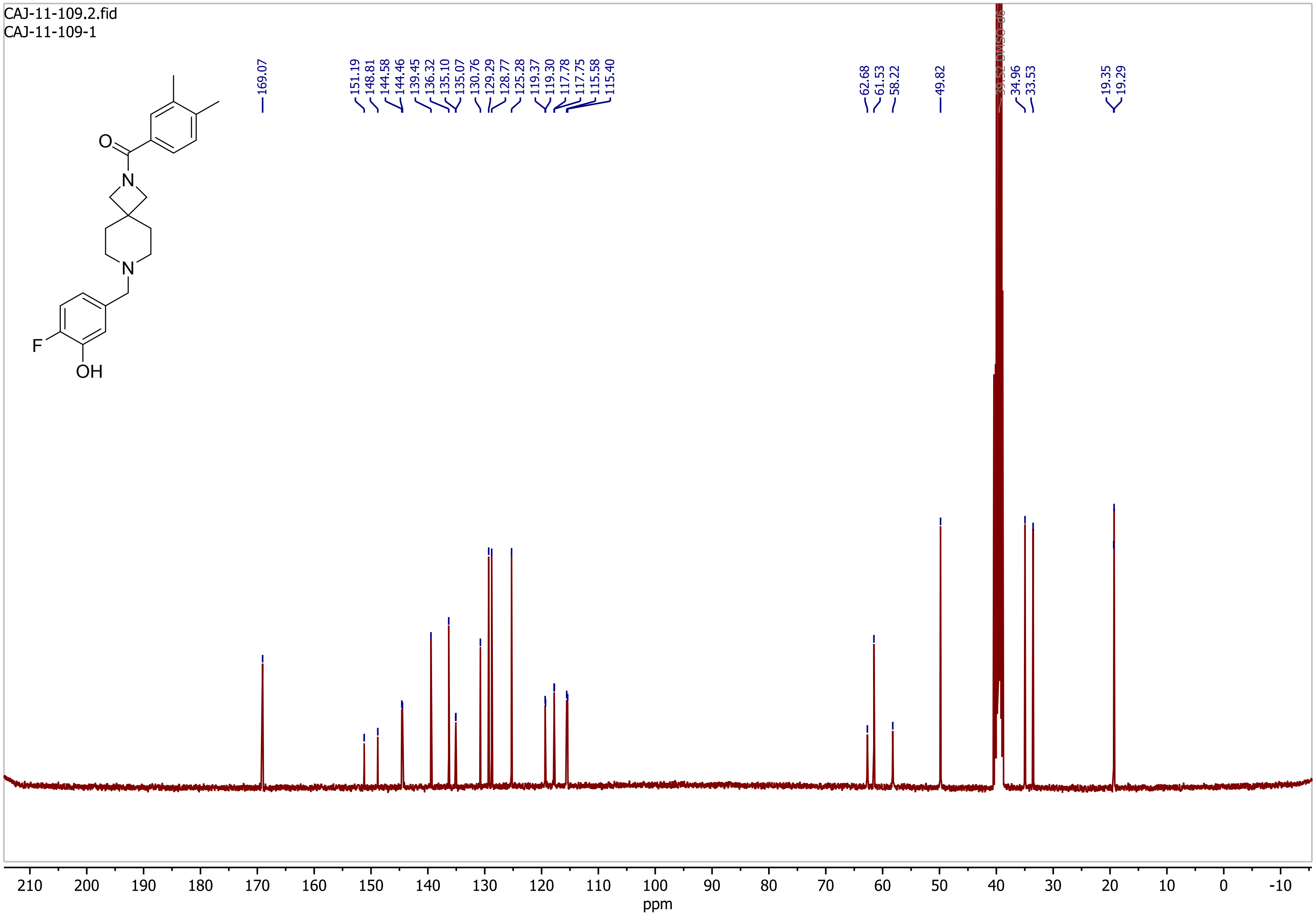

Figure S19: ^{13}C NMR spectra of **8** in DMSO-d_6.

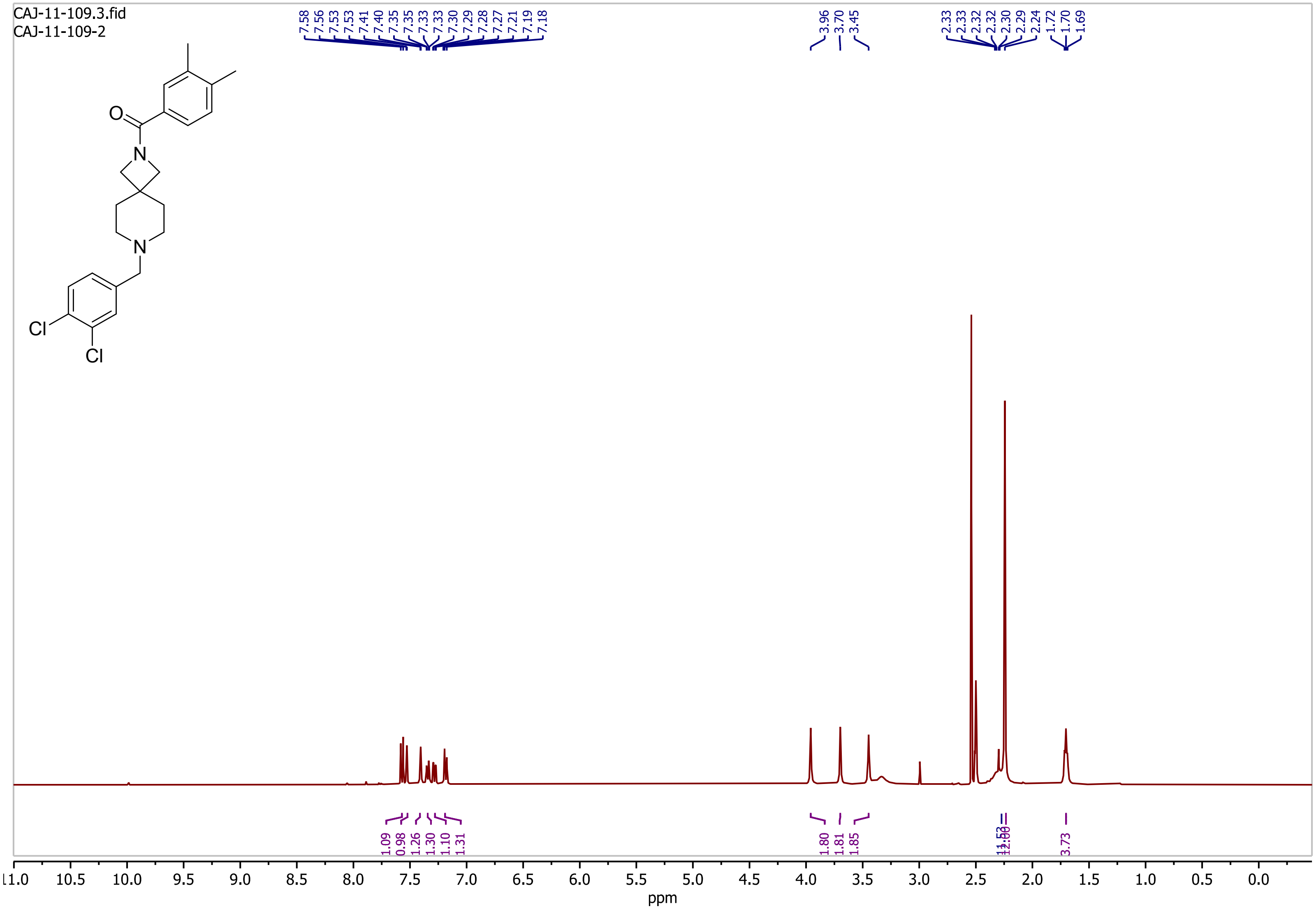

Figure S20: ^{1}H NMR spectra of **9** in DMSO-d_6.

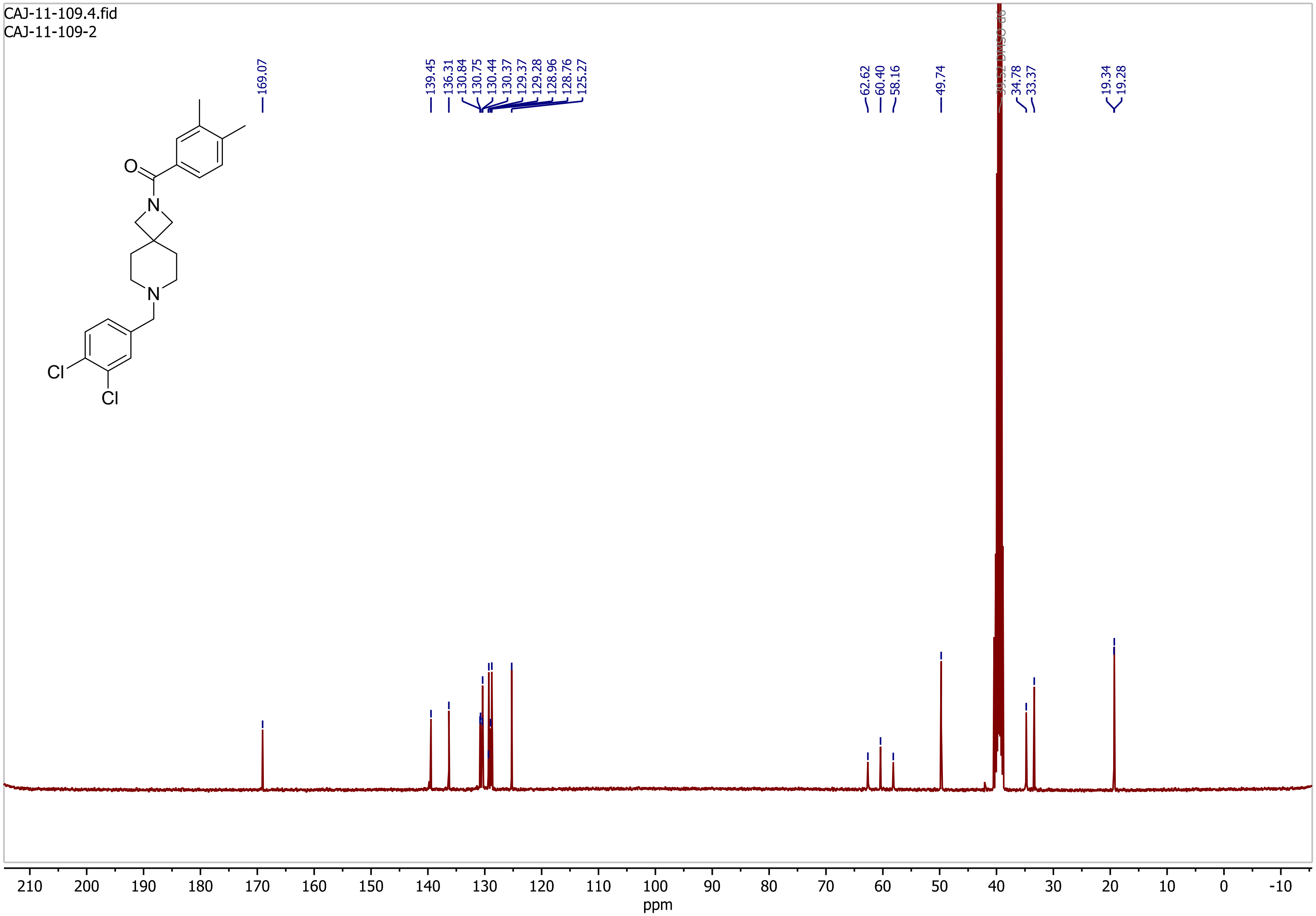

Figure S21: ^{13}C NMR spectra of **9** in DMSO-d_6.

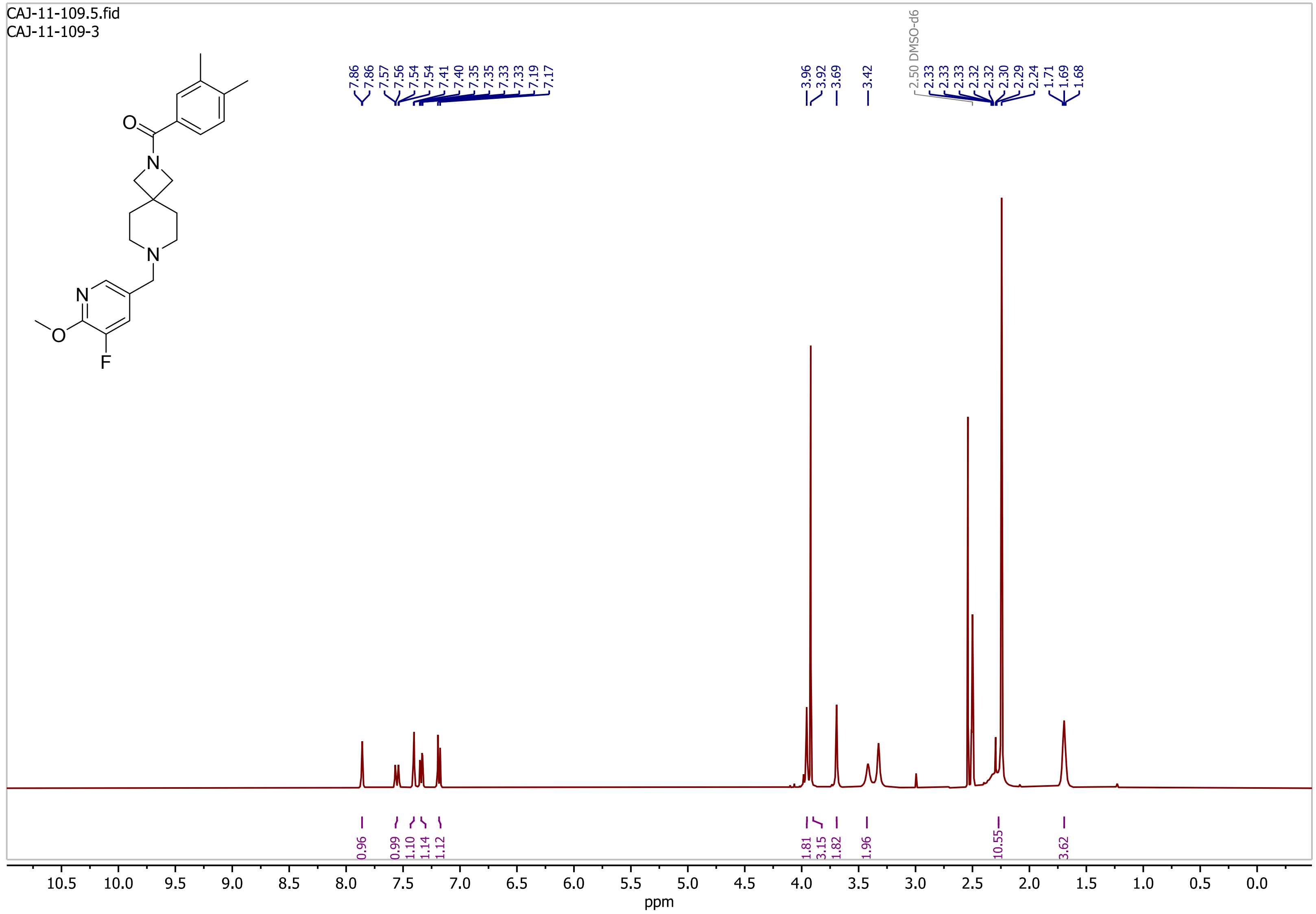

Figure S22: ^{1}H NMR spectra of **10** in DMSO-d_6.

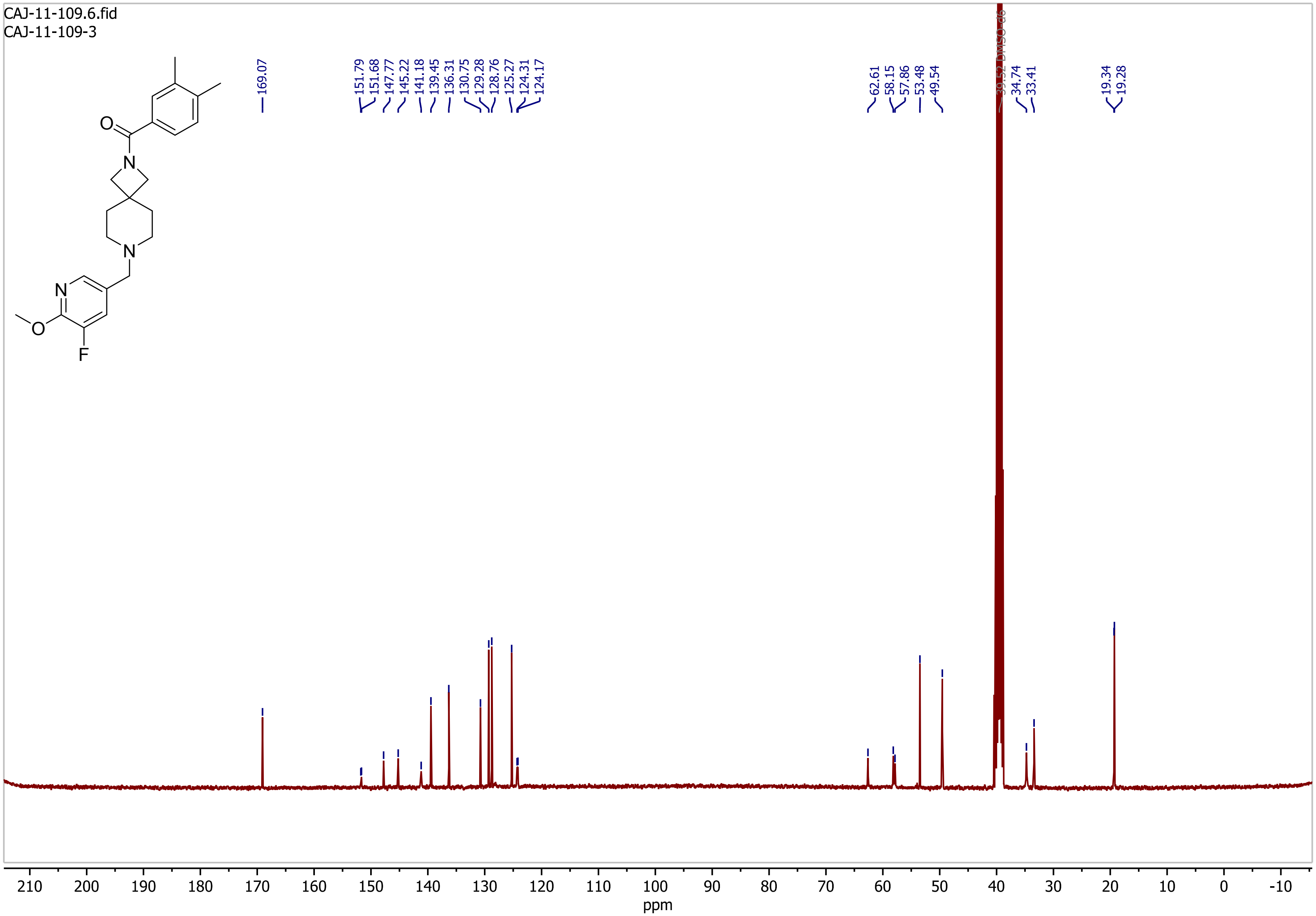

Figure S23: ^{13}C NMR spectra of **10** in DMSO-d_6.

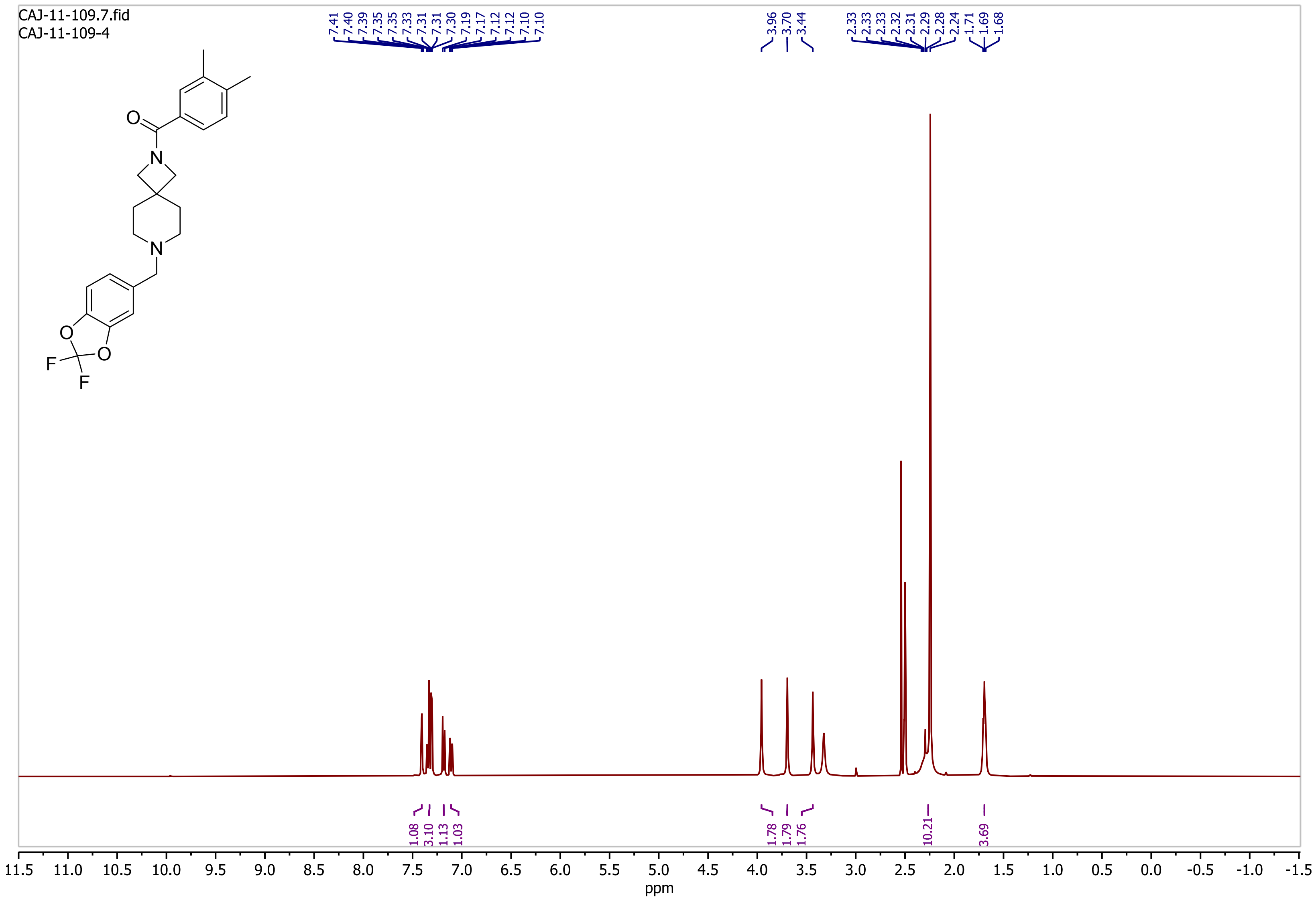

Figure S24: ^{1}H NMR spectra of **11** in DMSO-d_6.

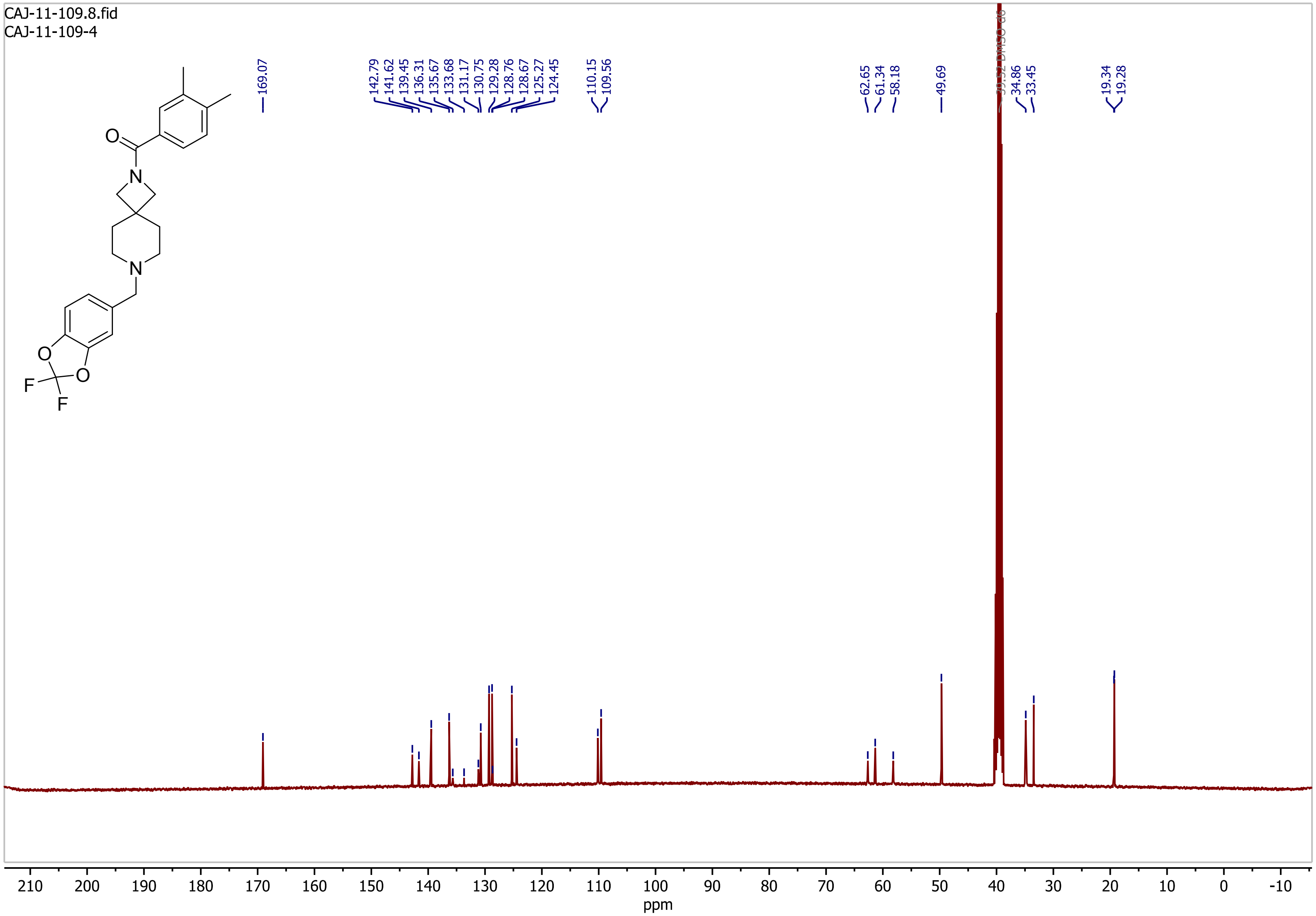

Figure S25: ^{13}C NMR spectra of **11** in DMSO-d_6.

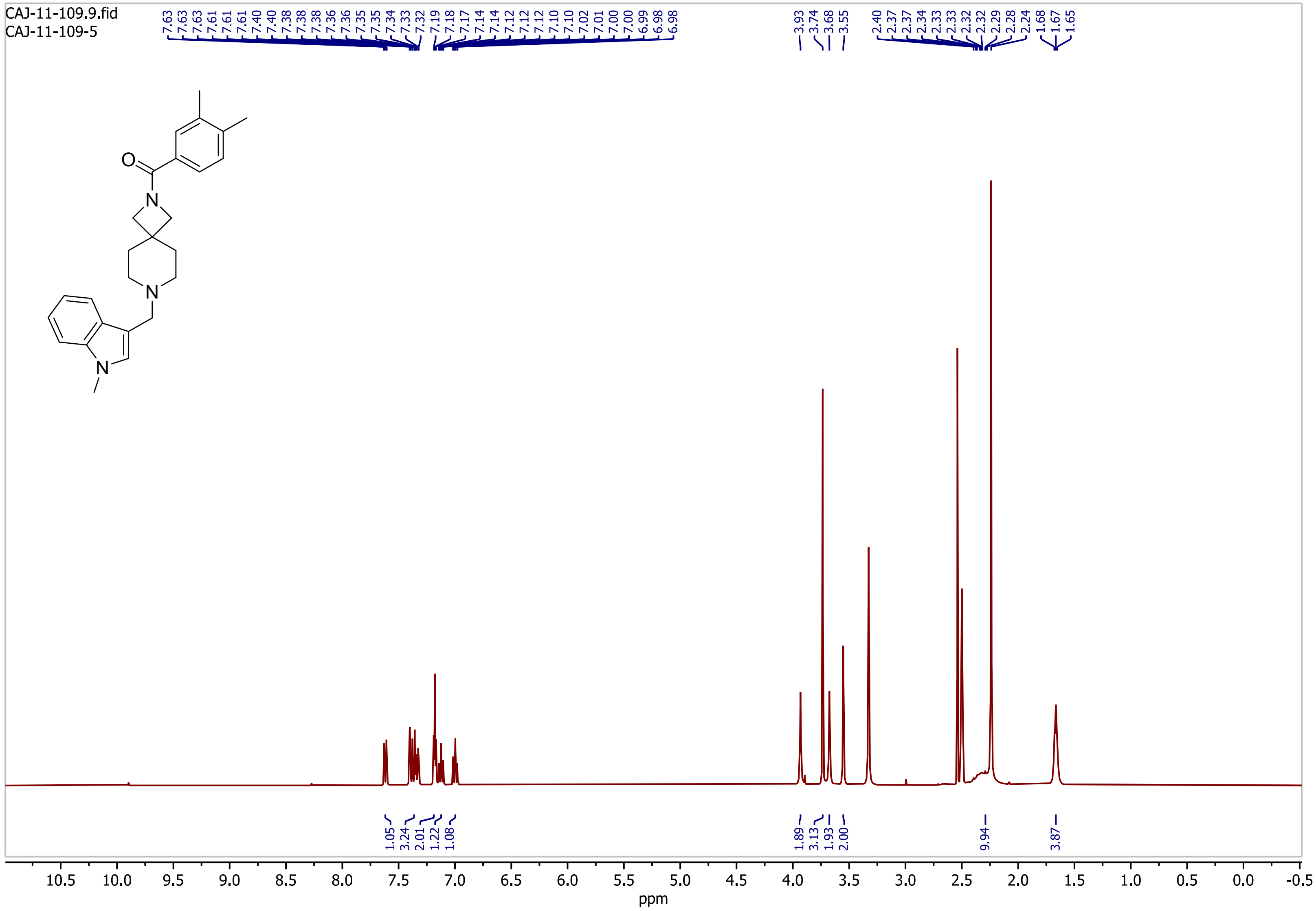

Figure S26: ^{1}H NMR spectra of **12** in DMSO-d_6.

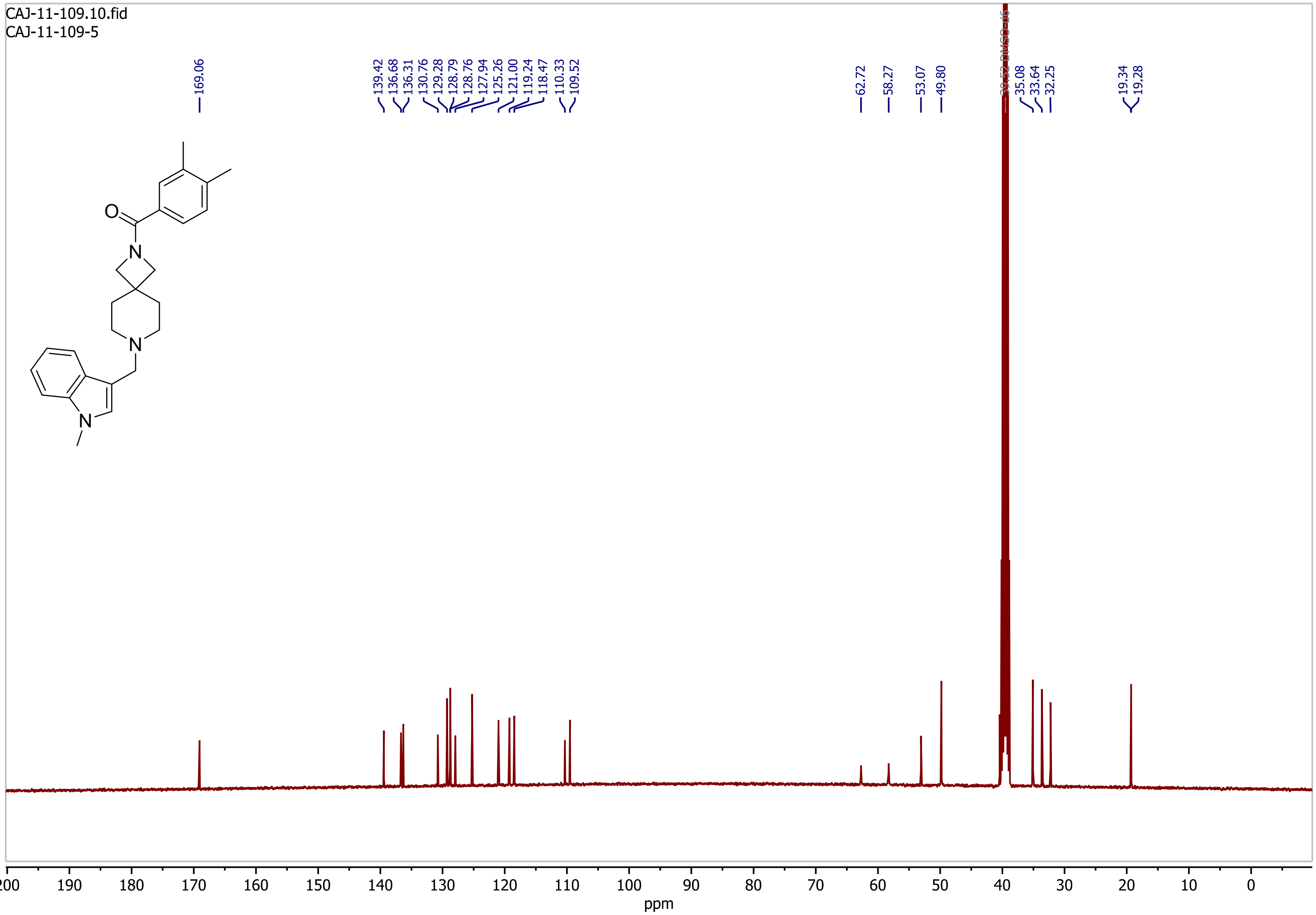

Figure S27: ^{13}C NMR spectra of **12** in DMSO-d_6.

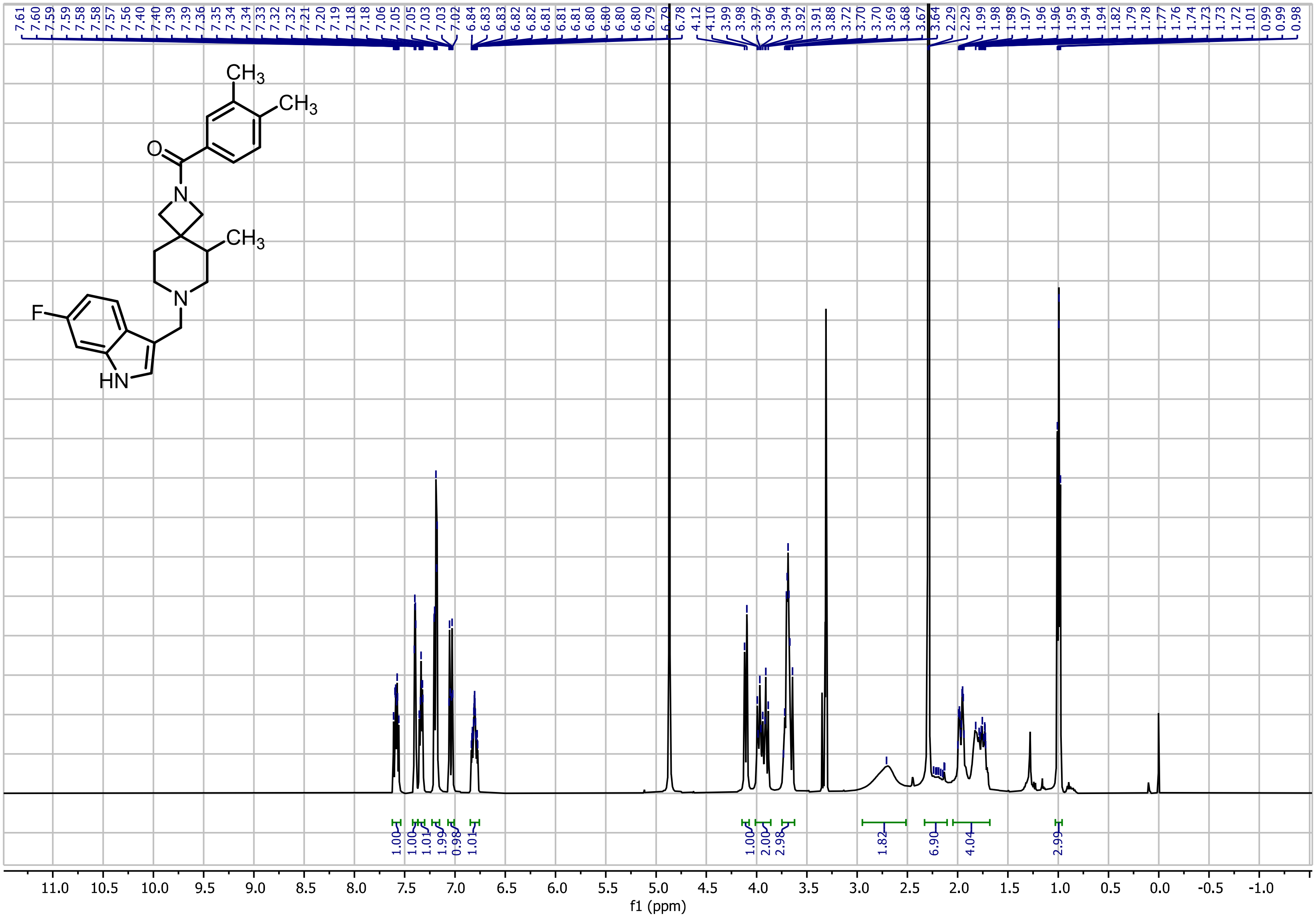

Figure S28: ^{1}H spectrum of **13** in MeOD-d_4.

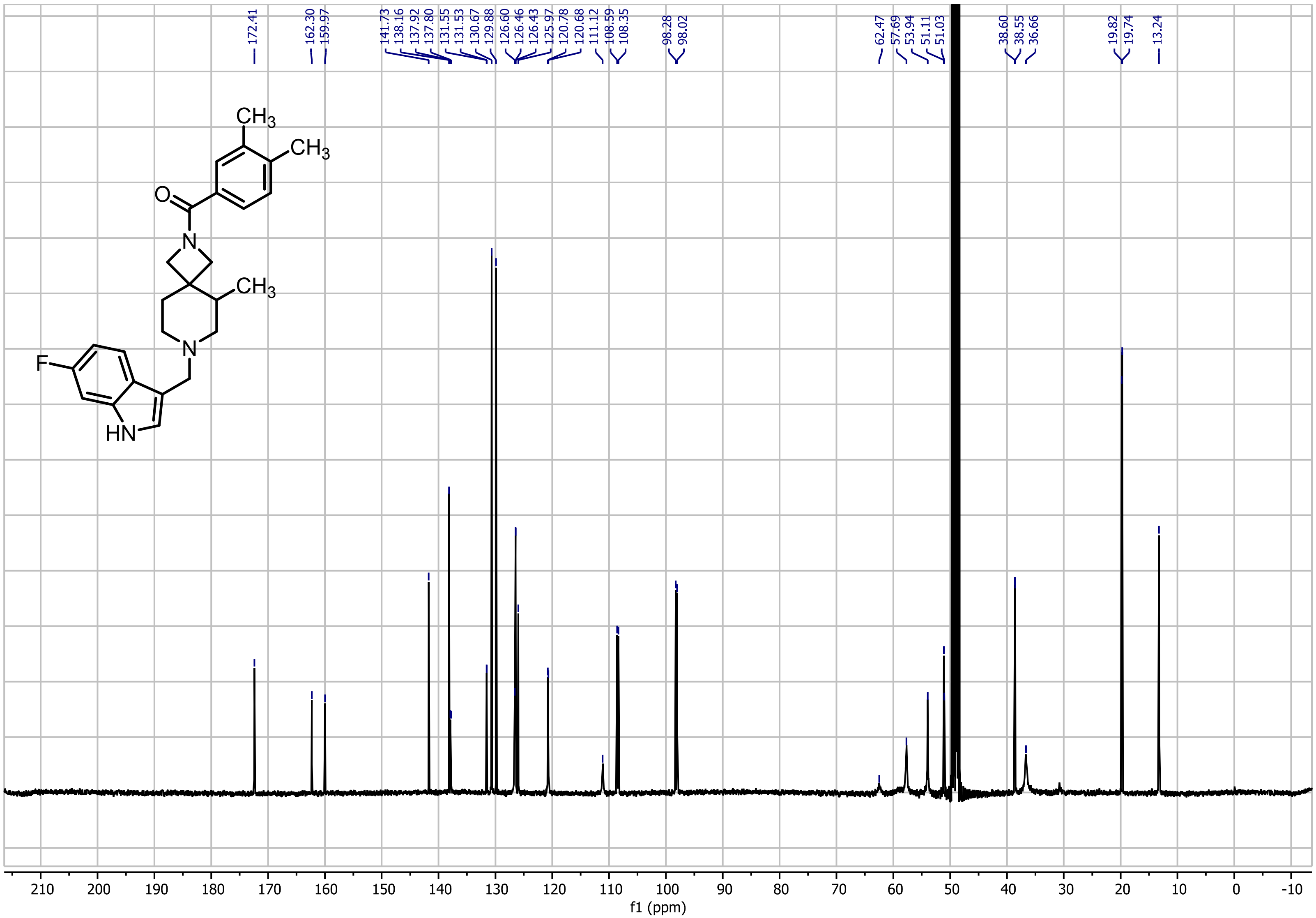

Figure S29: ^{13}C spectrum of **13** in MeOD-d_4.

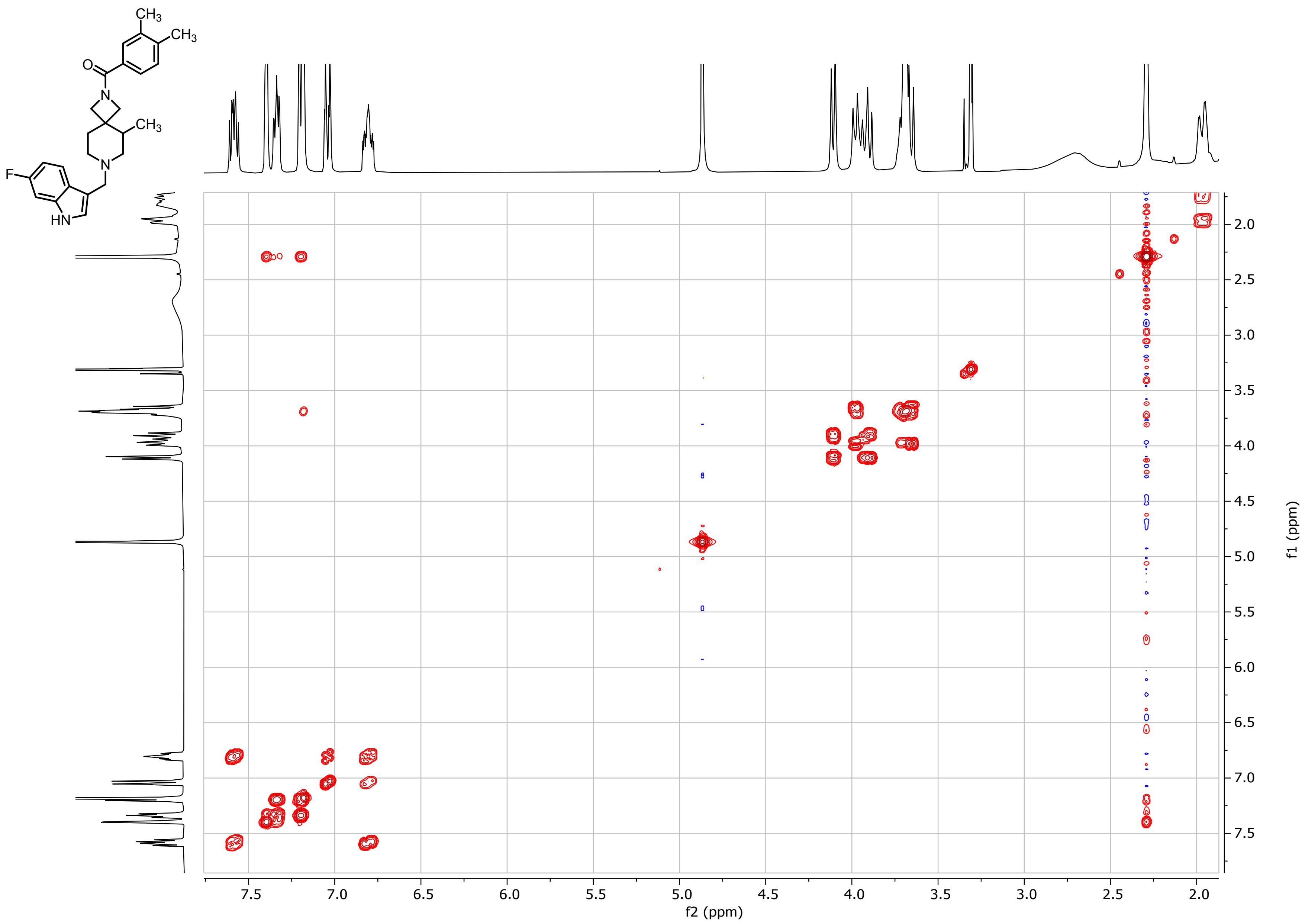

Figure S30: COSY spectrum of **13** in MeOD-d_4.

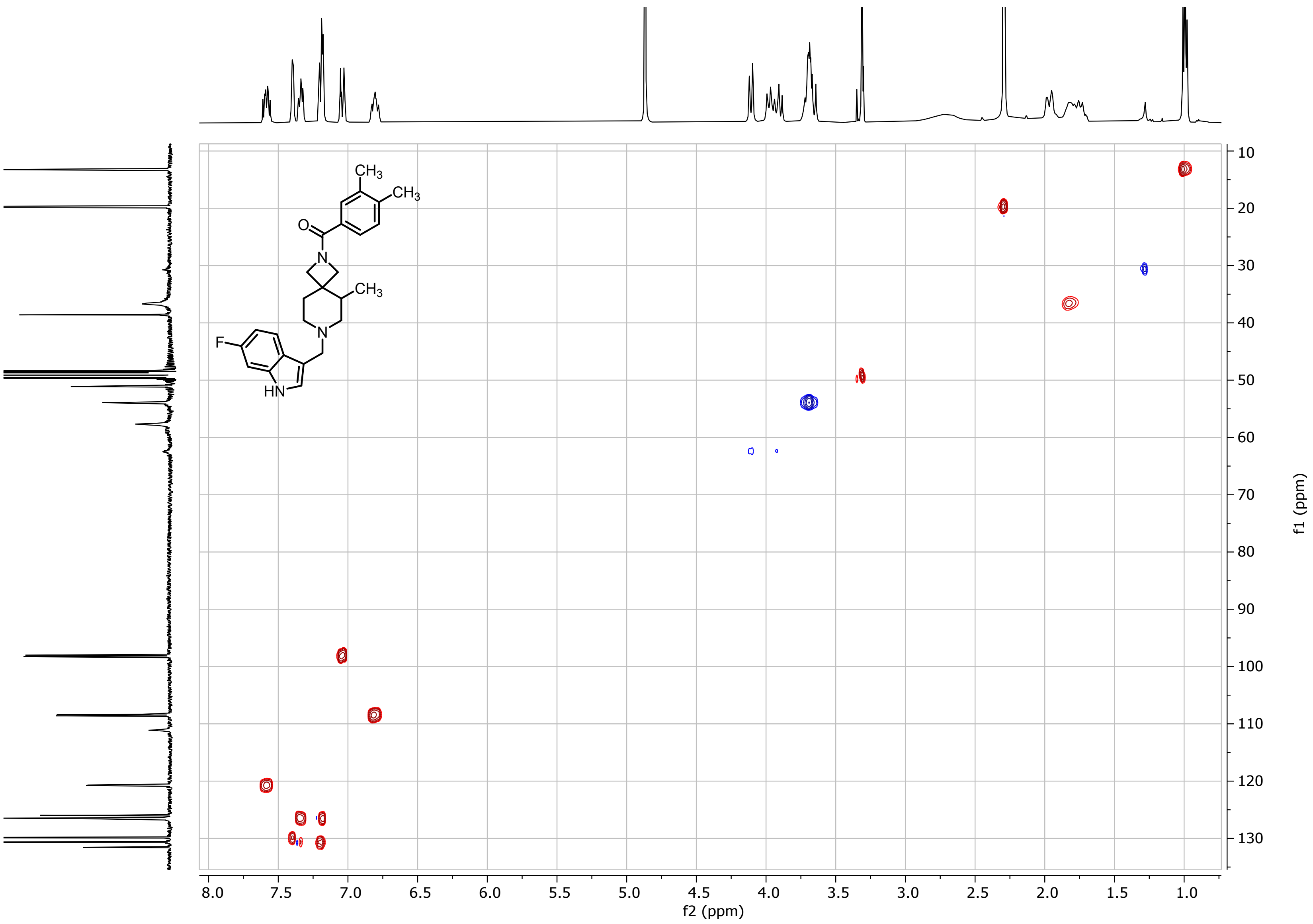

Figure S31: HSQC spectrum of **13** in MeOD-d_4.

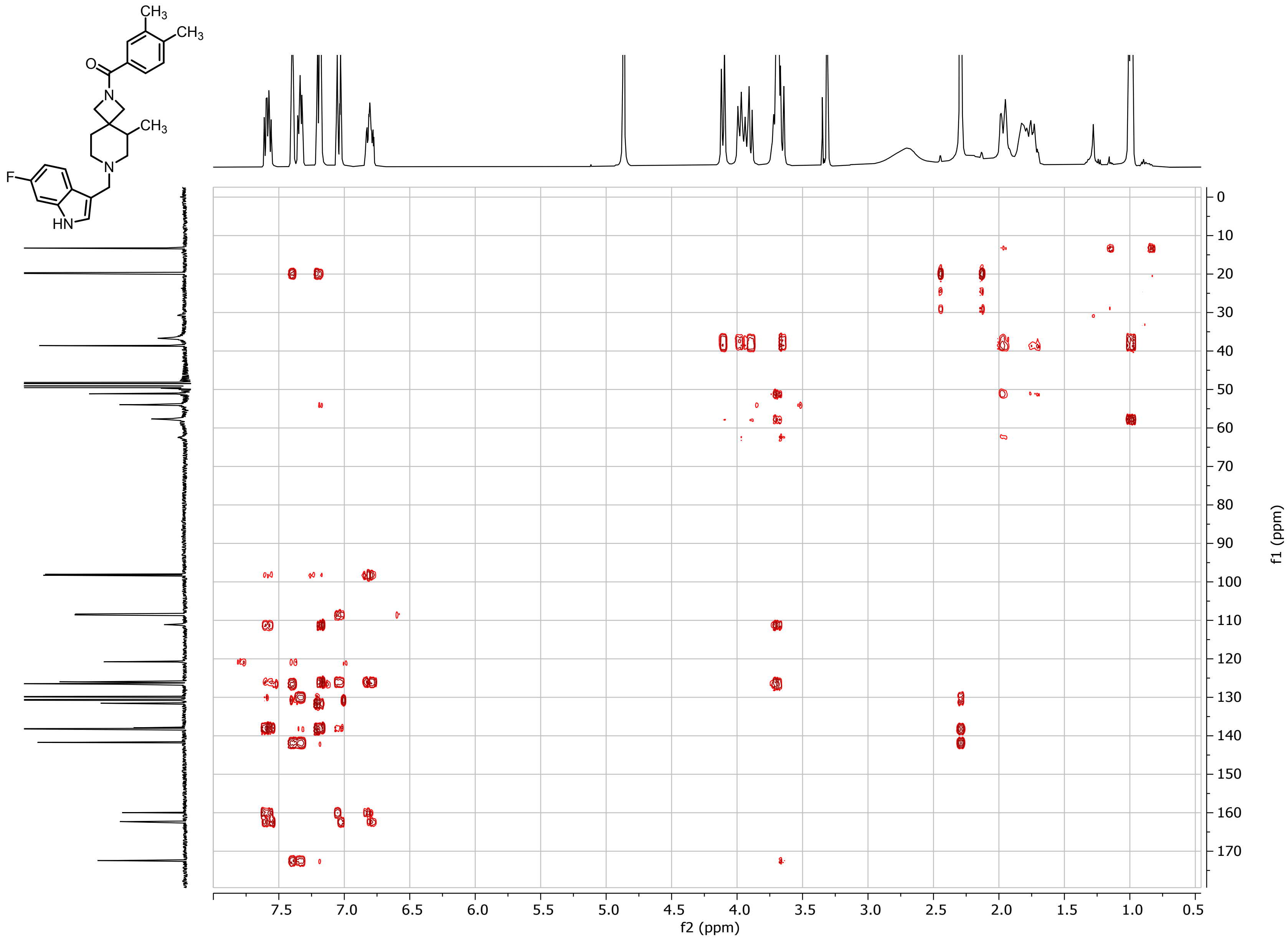

Figure S32: HMBC spectrum of **13** in MeOD-d_4.

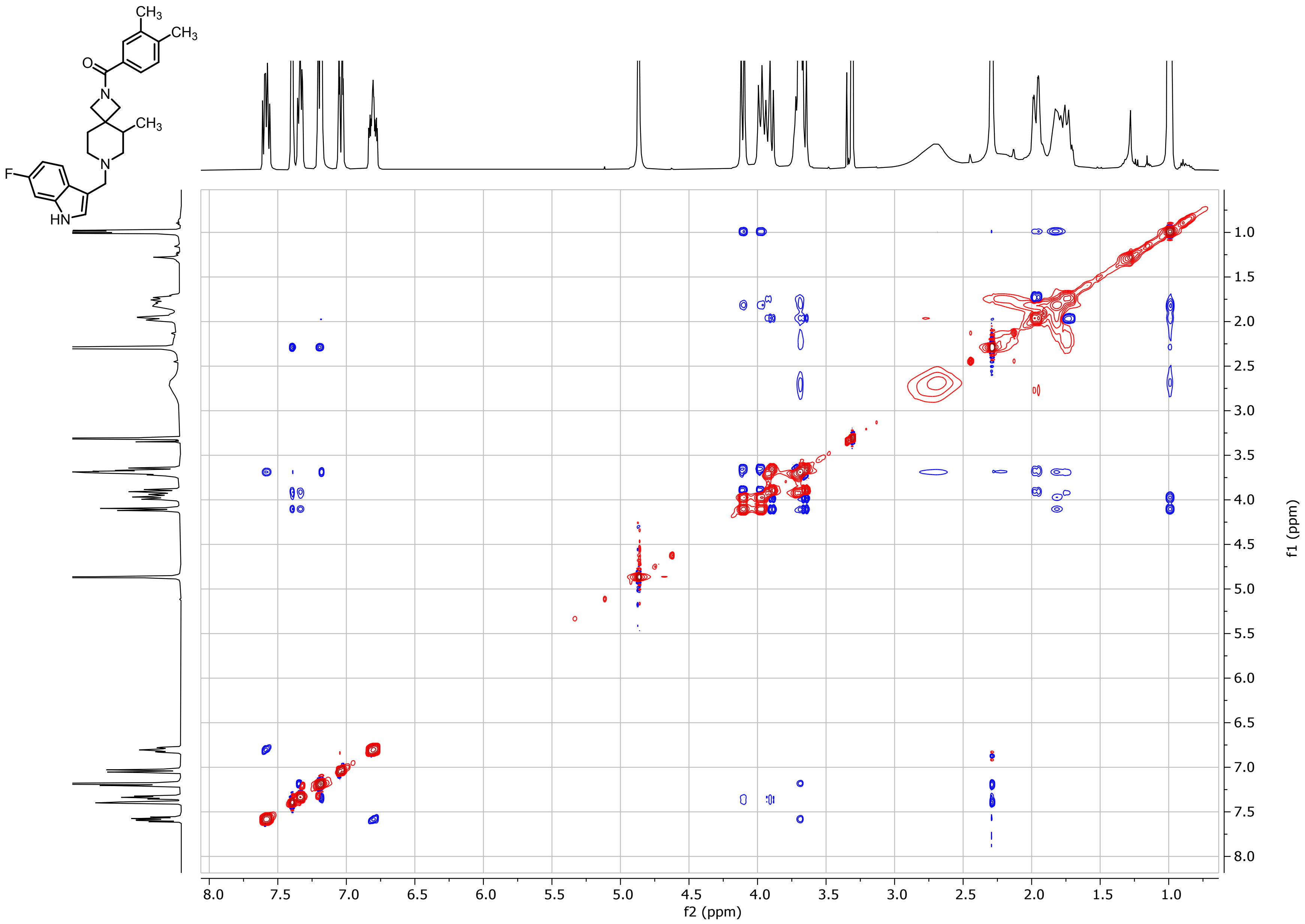

Figure S33: 2D NOESY spectrum of **13** in MeOD-d_4.

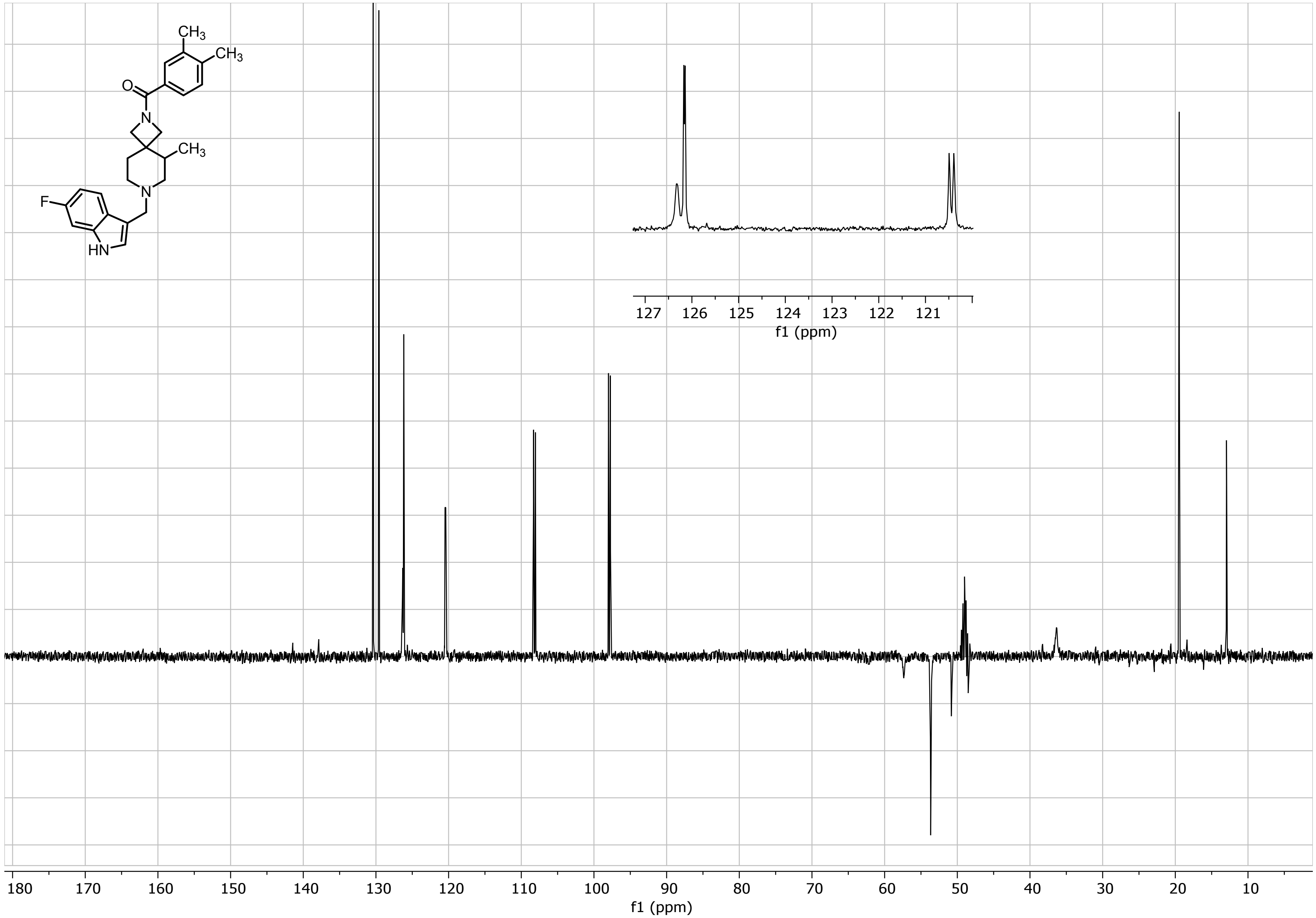

Figure S34: DEPT-135 spectrum of **13** in MeOD-d_4.

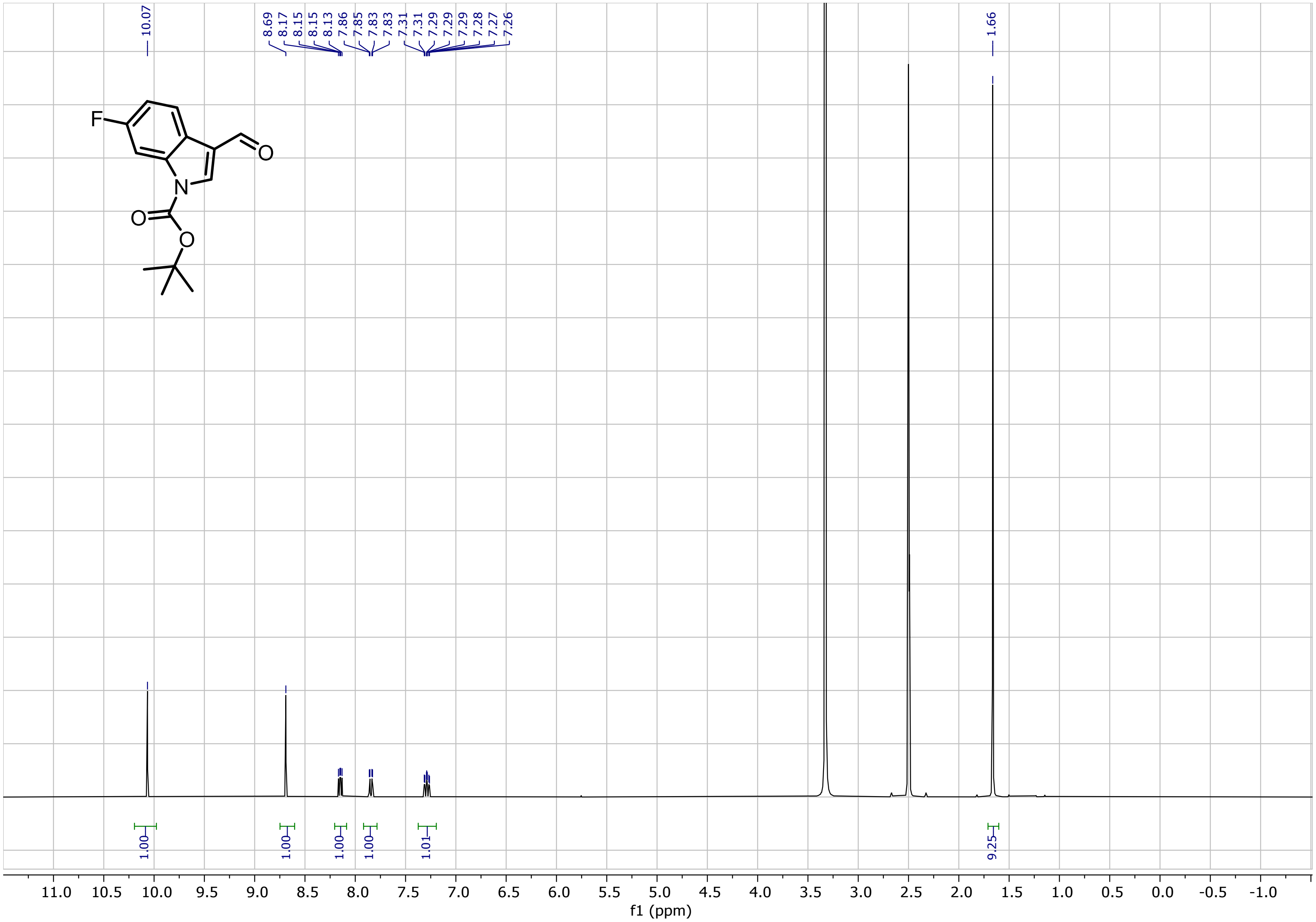

Figure S35: ^{1}H NMR spectrum of **35** in DMSO-d_6.

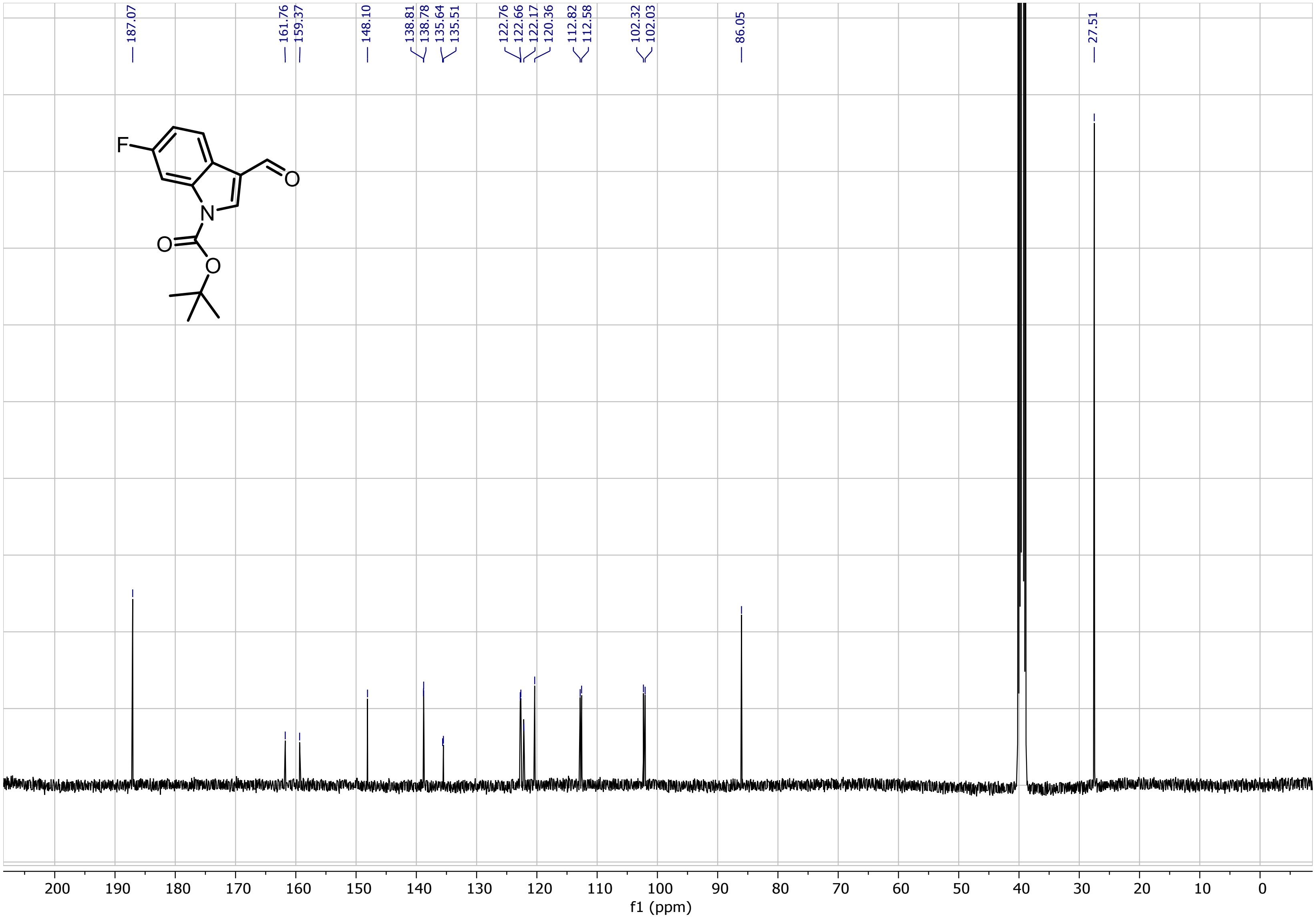

Figure S36: ^{13}C NMR spectrum of **35** in DMSO-d_6.

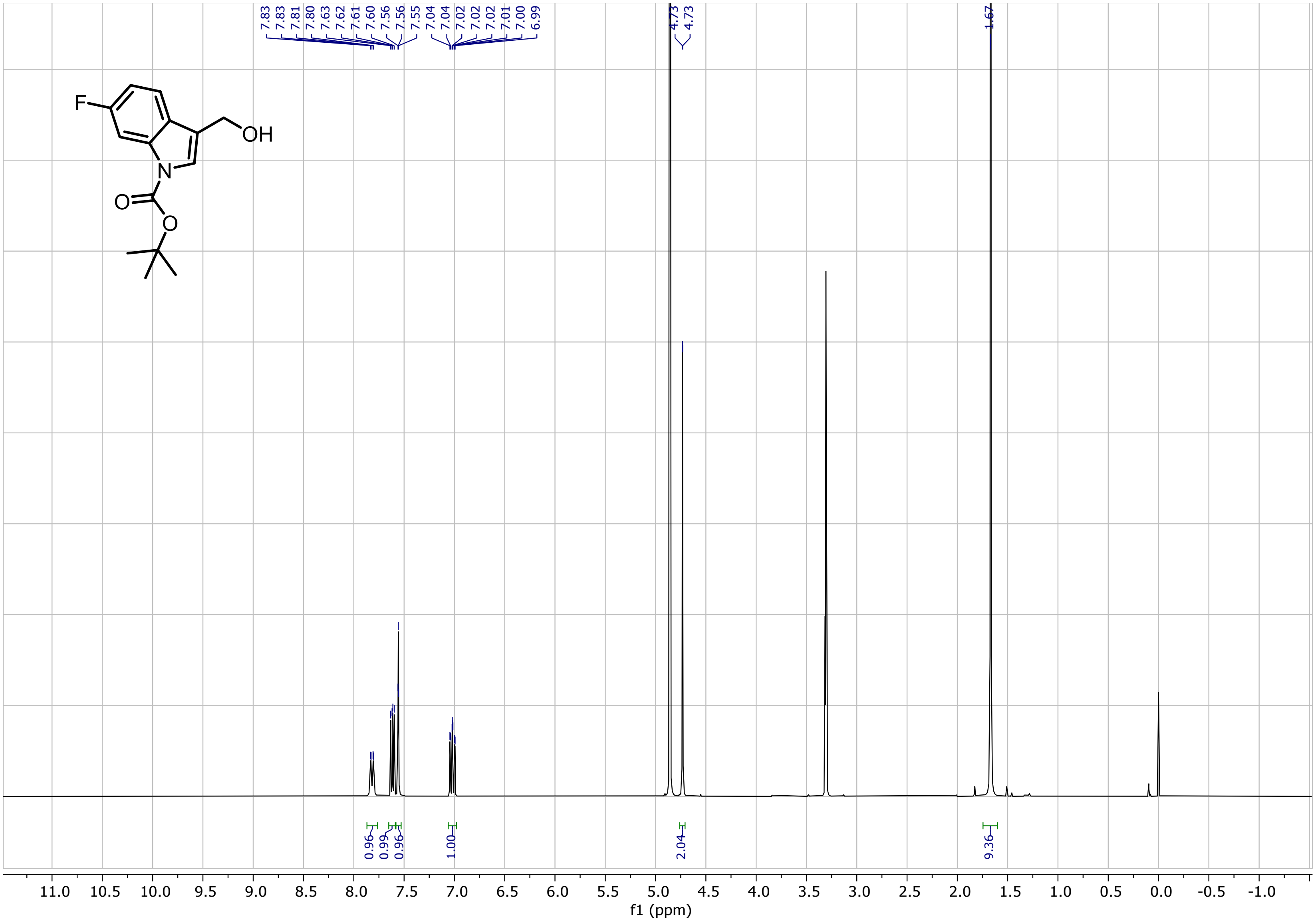

Figure S37: ^{1}H NMR spectrum of **36** in MeOD-d_4.

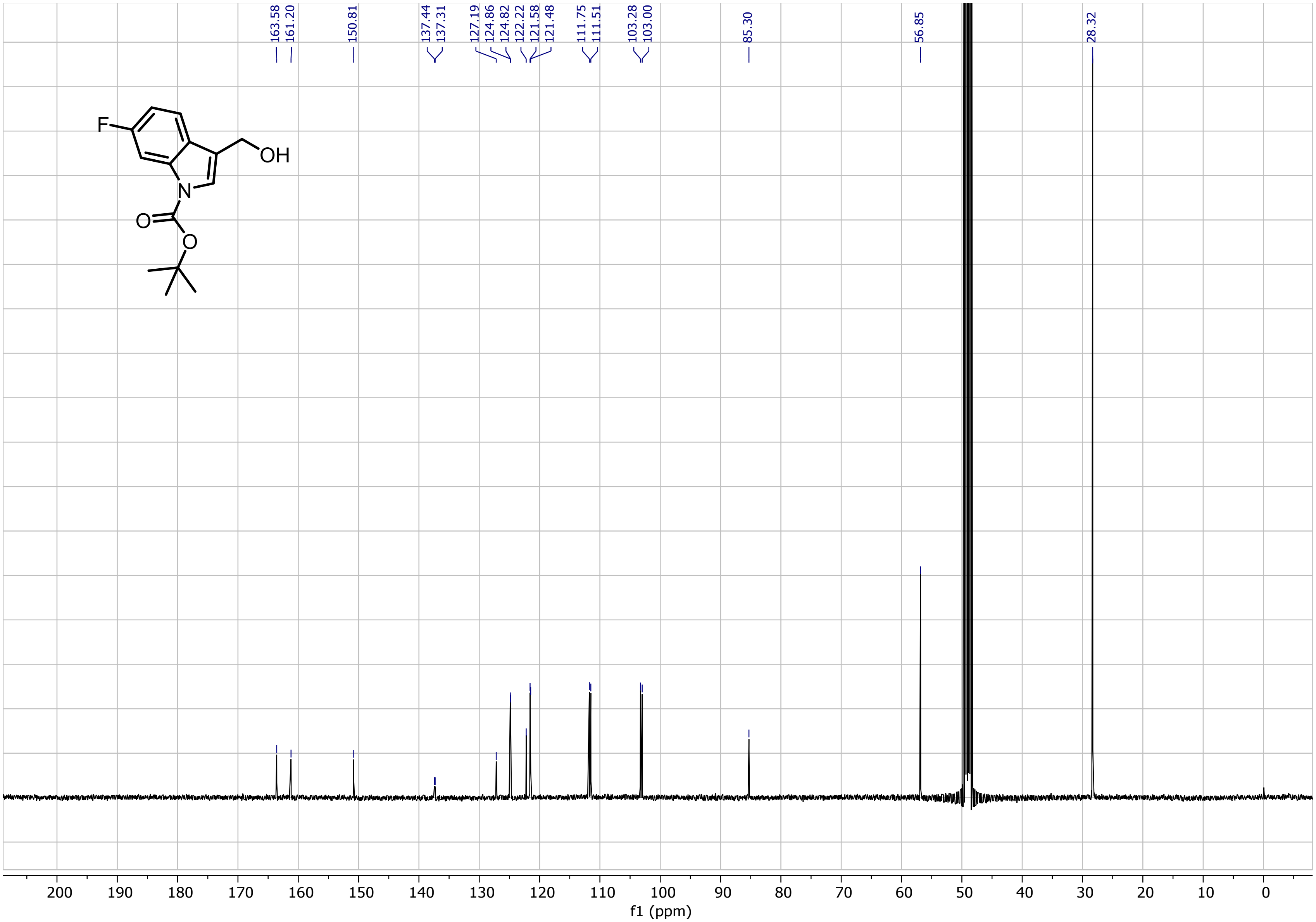

Figure S38: ^{13}C NMR spectrum of **36** in MeOD-d_4.

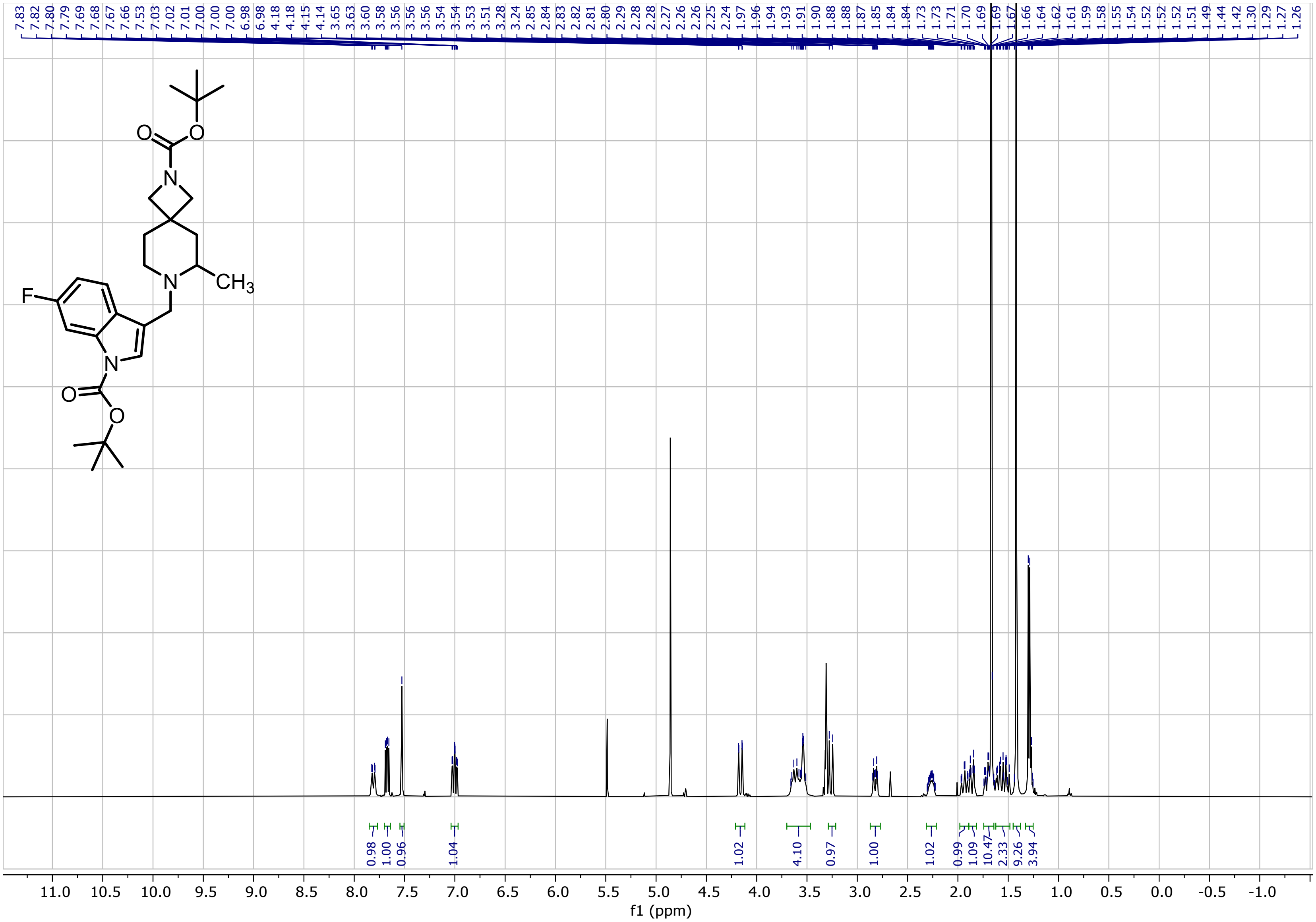

Figure S39: ^{1}H NMR spectrum of **38** in MeOD-d_4.

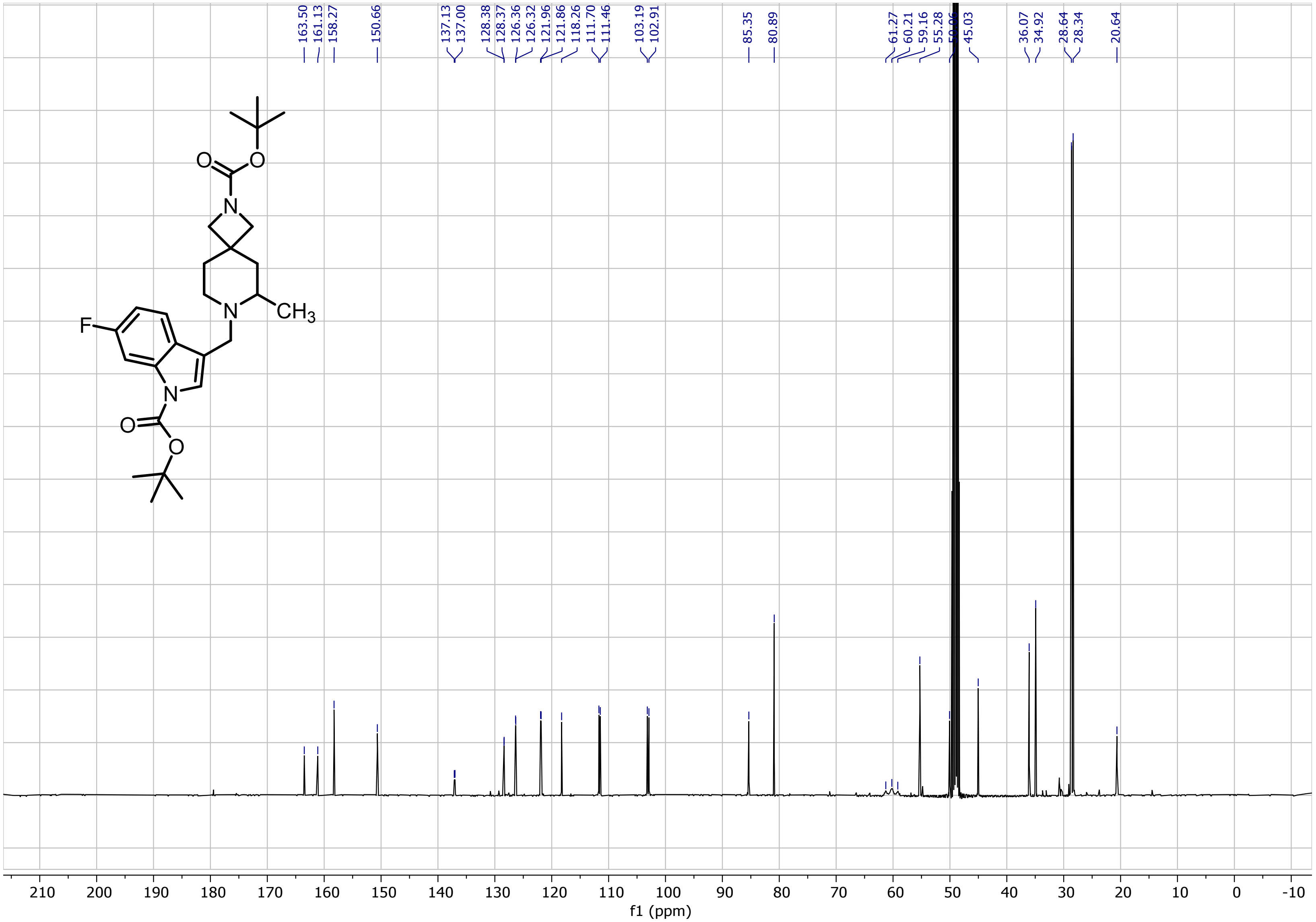

Figure S40: ^{13}C NMR spectrum of **38** in MeOD-d_4.

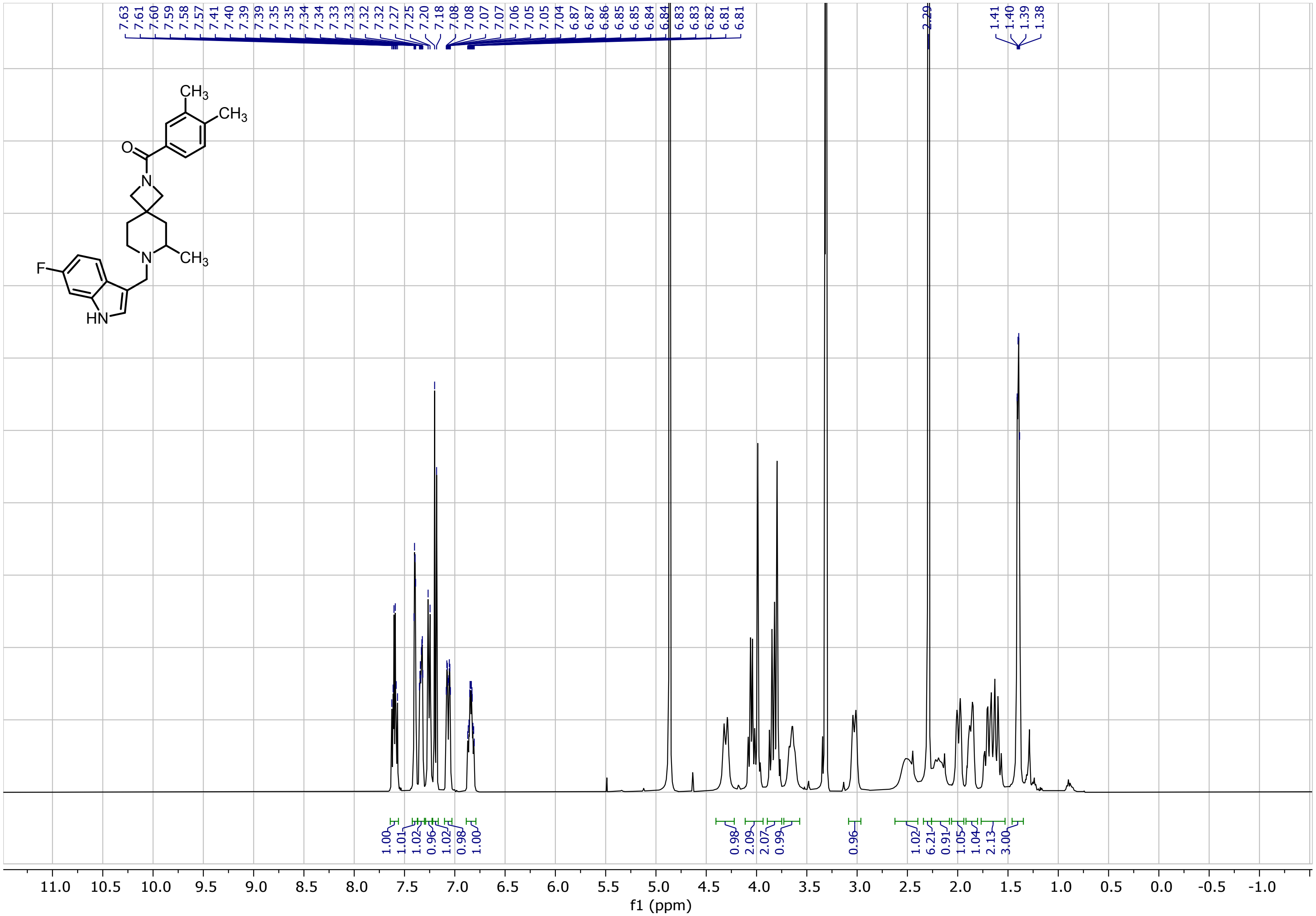

Figure S41: ^{1}H NMR spectrum of **14** in MeOD-d_4.

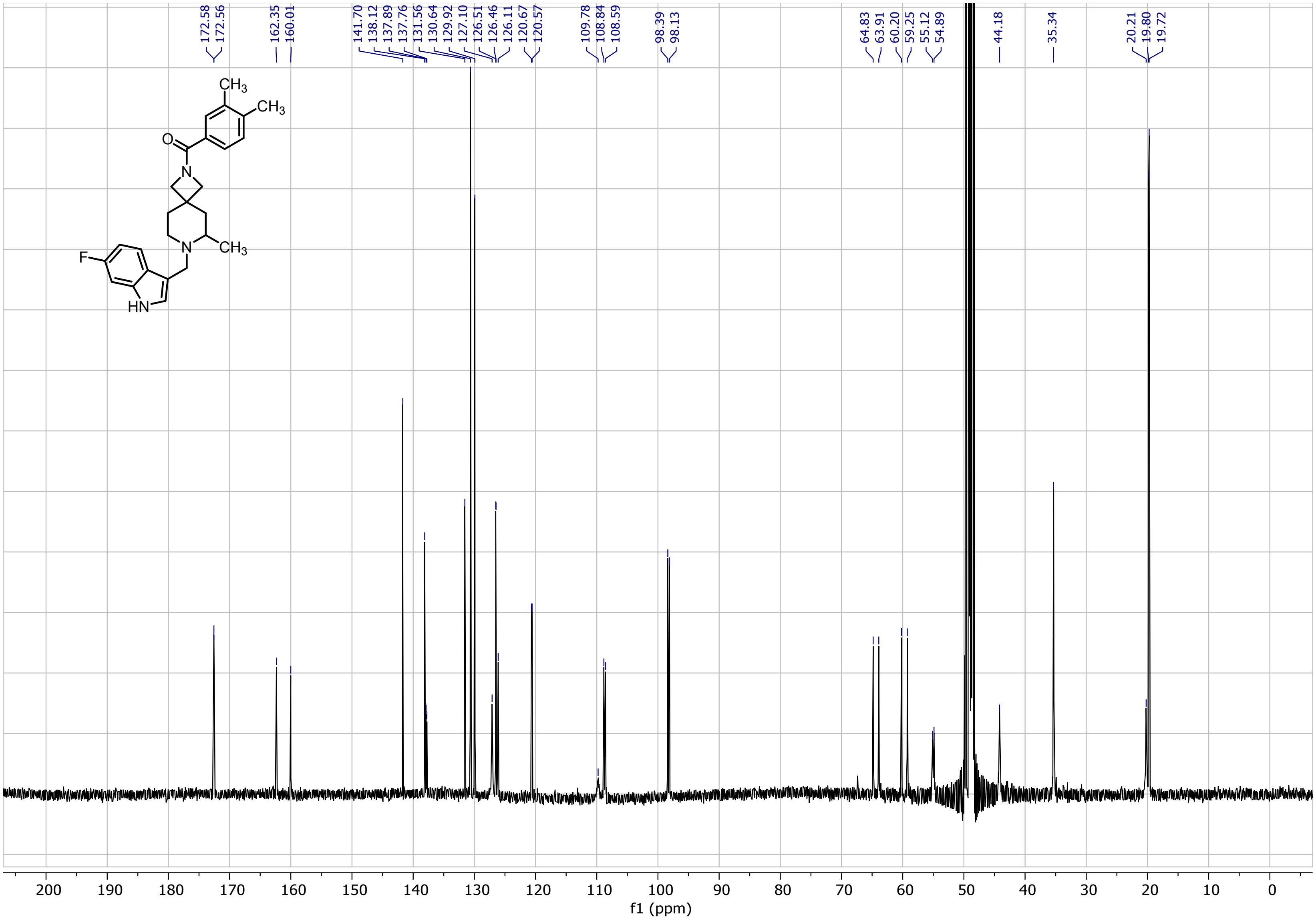

Figure S42: ^{13}C NMR spectrum of **14** in MeOD-d_4.

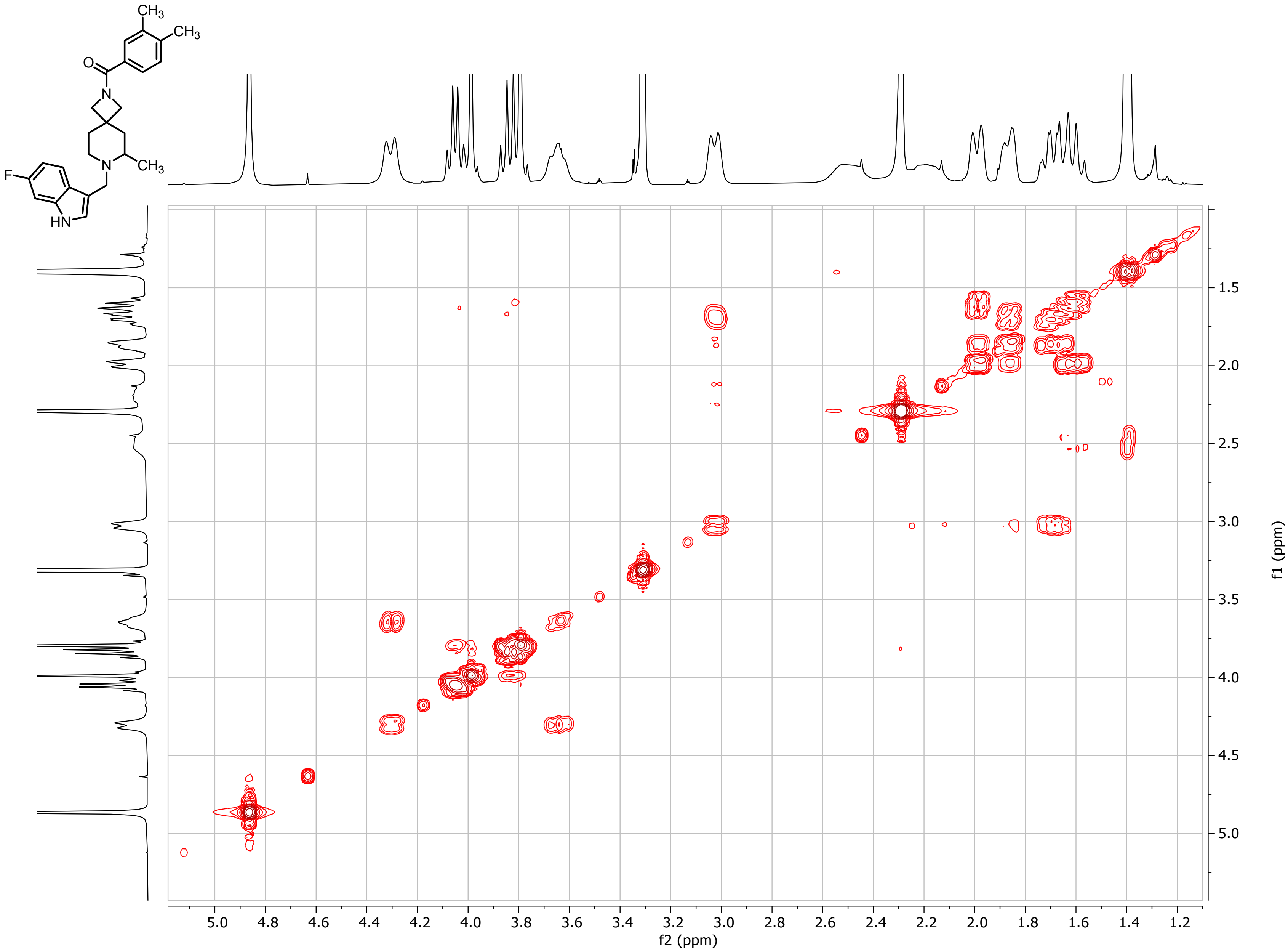

Figure S43: COSY spectrum of **14** in MeOD-d_4.

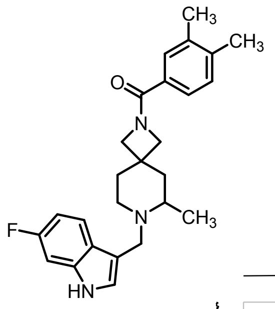

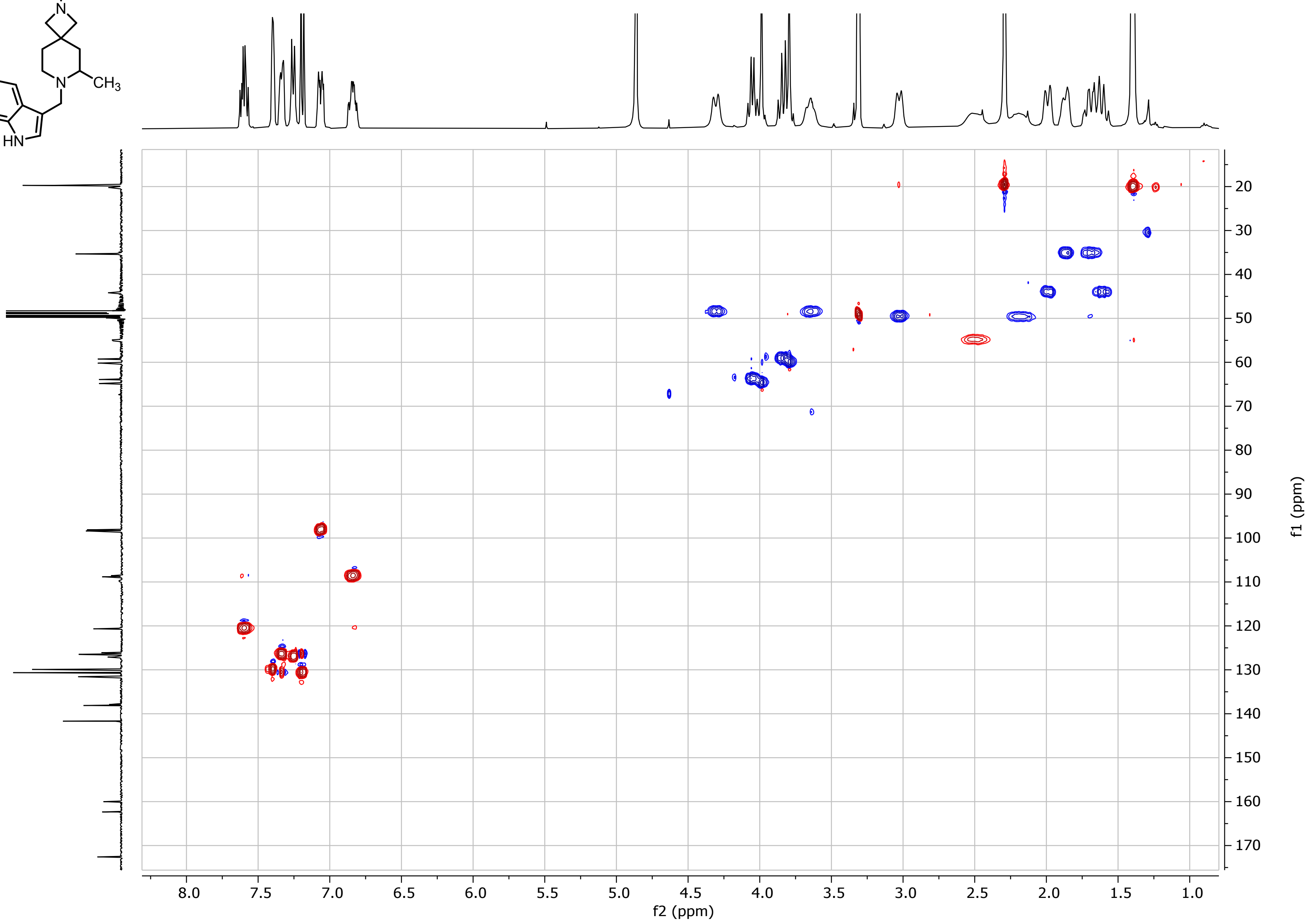

Figure S44: HSQC spectrum of **14** in MeOD-d_4.

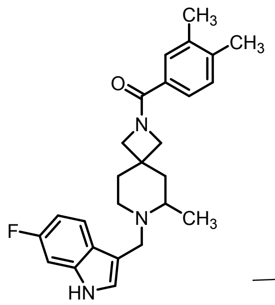

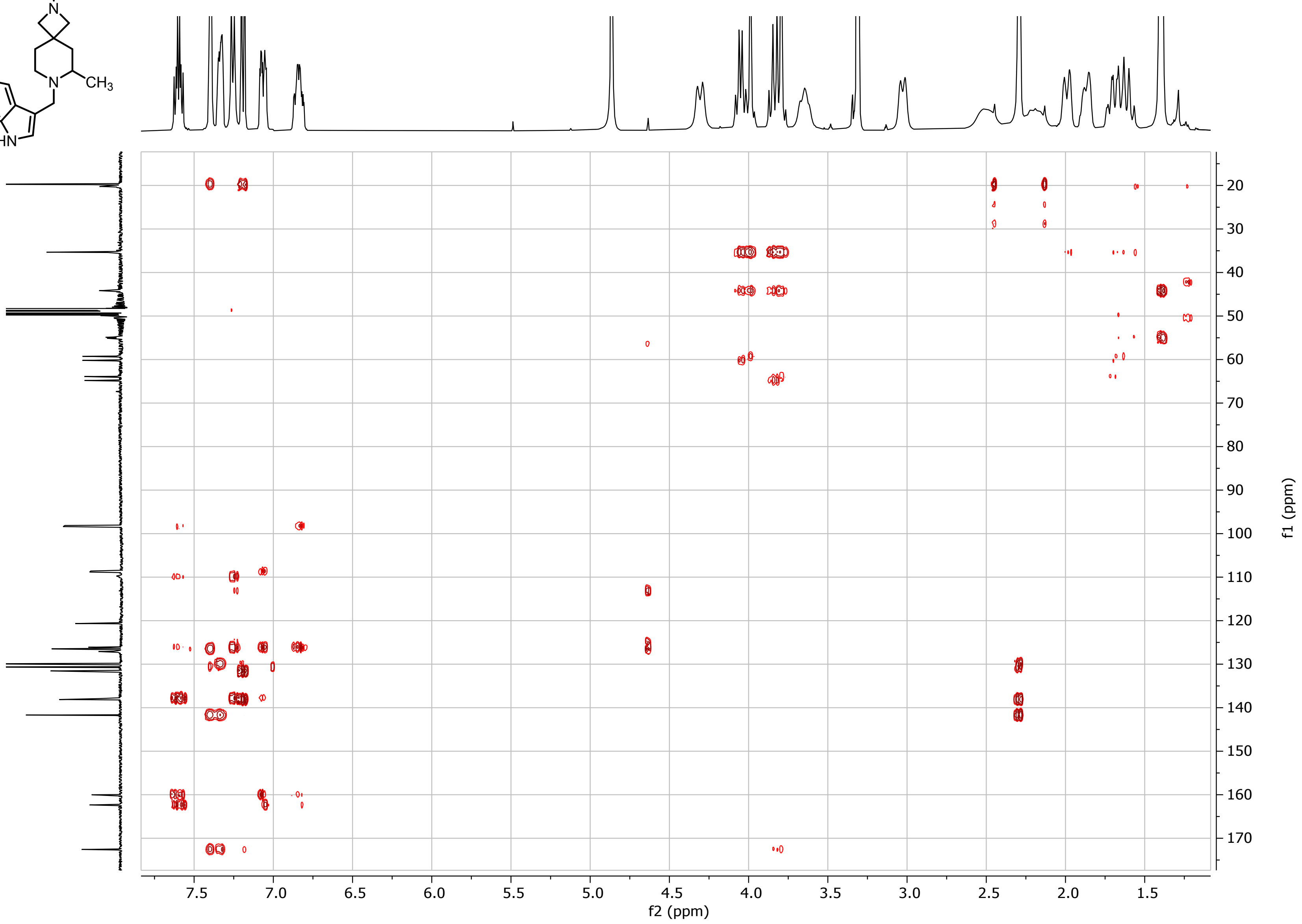

Figure S45: HMBC spectrum of **14** in MeOD-d_4.

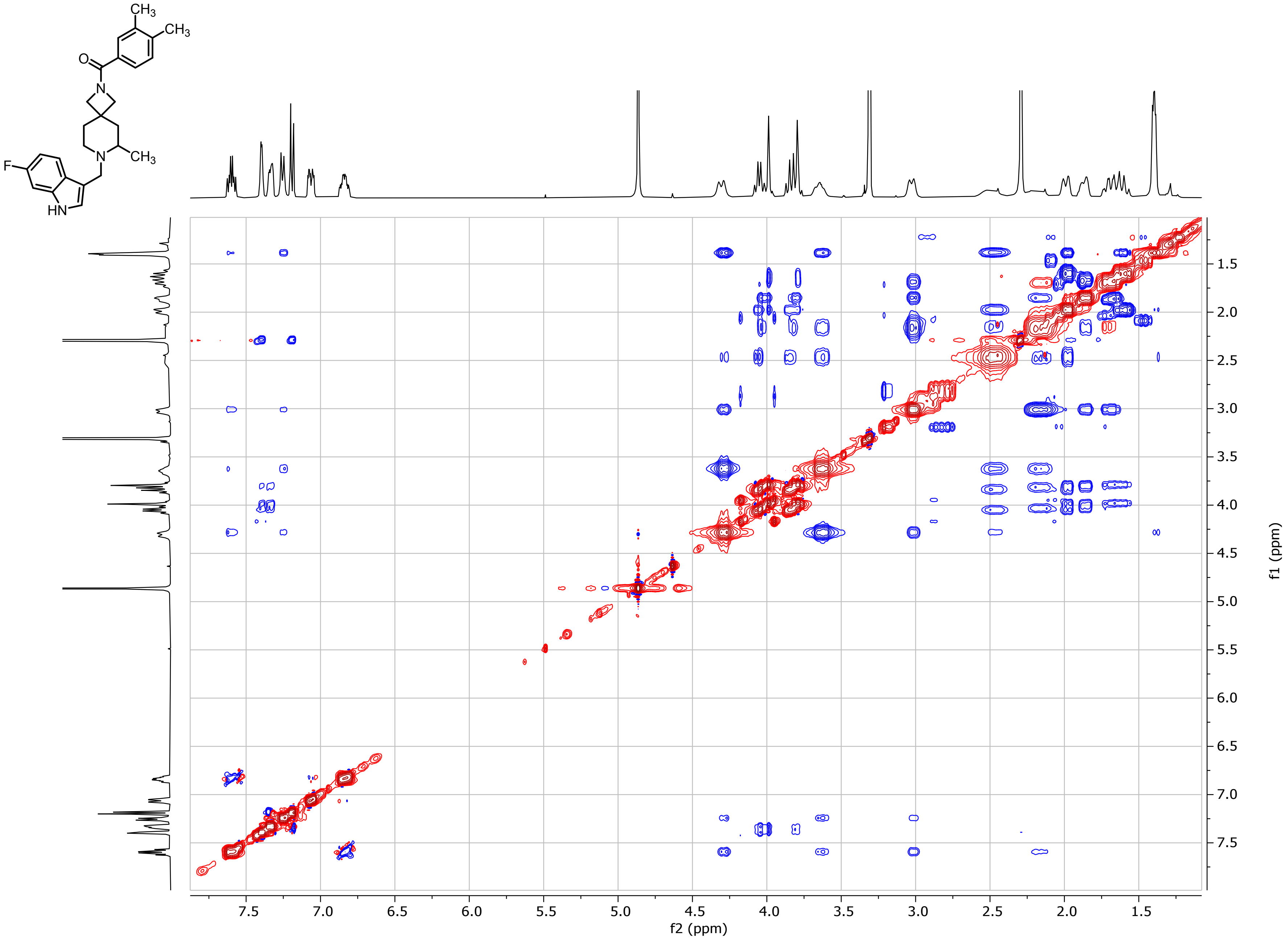

Figure S46: 2D-NOESY spectrum of **14** in MeOD-d_4.

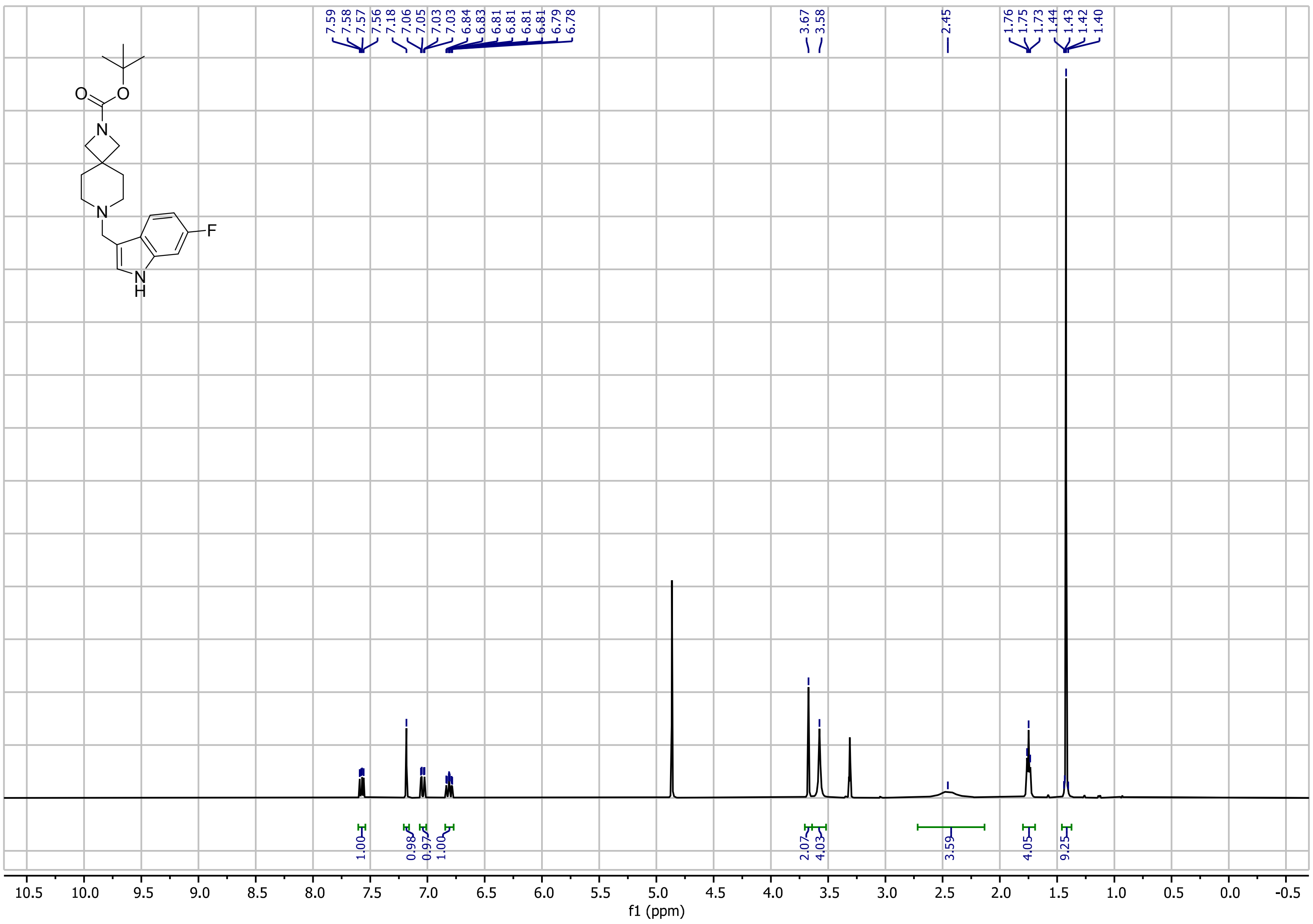

Figure S47: ^{1}H NMR spectra of **15** in MeOD-d_4.

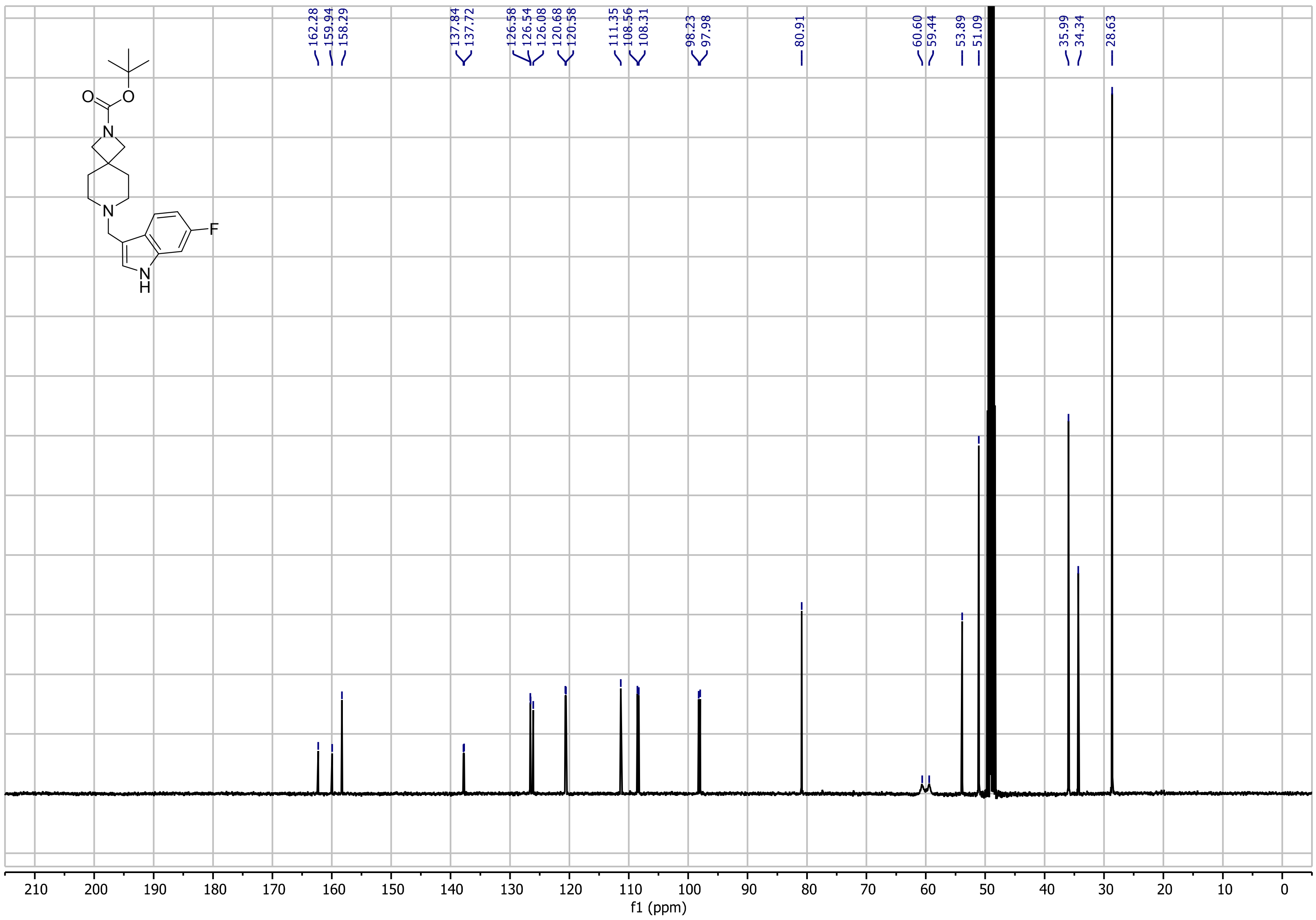

Figure S48: ^{13}C NMR spectra of **15** in MeOD-d_4.

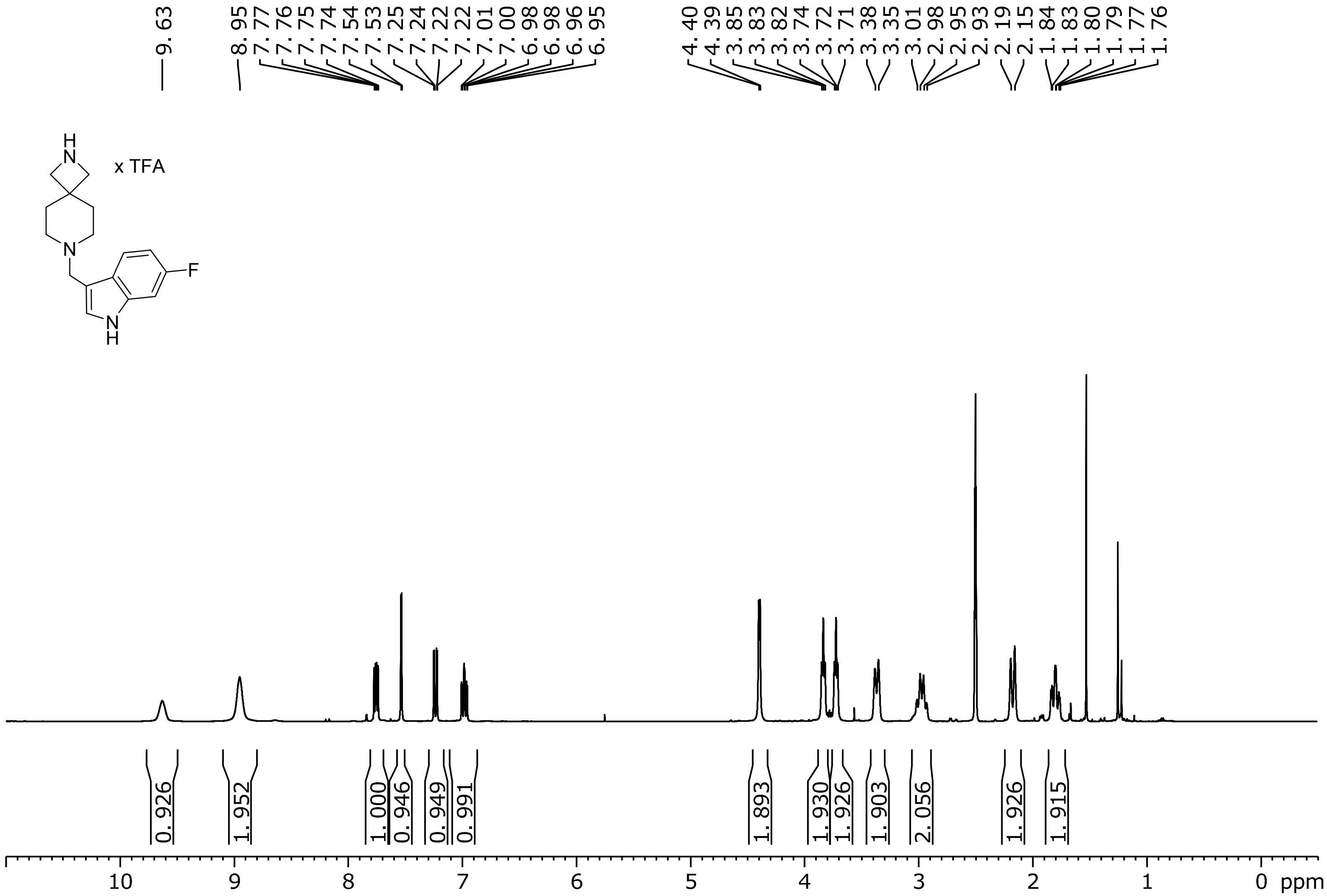

Figure S49: ^{1}H NMR spectra of **16** in MeOD-d_4.

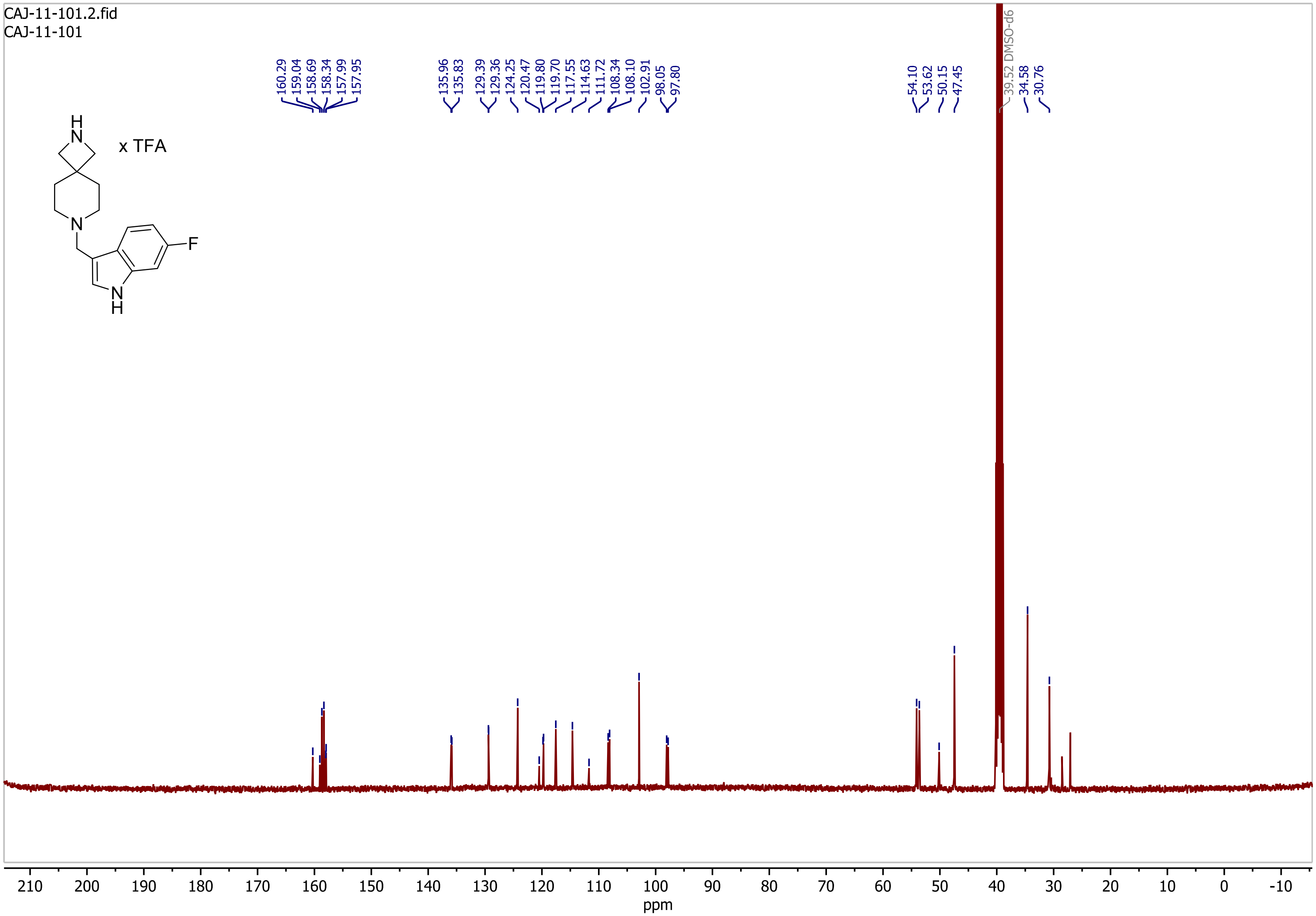

Figure S50: ^{13}C NMR spectra of **16** in MeOD-d_4.

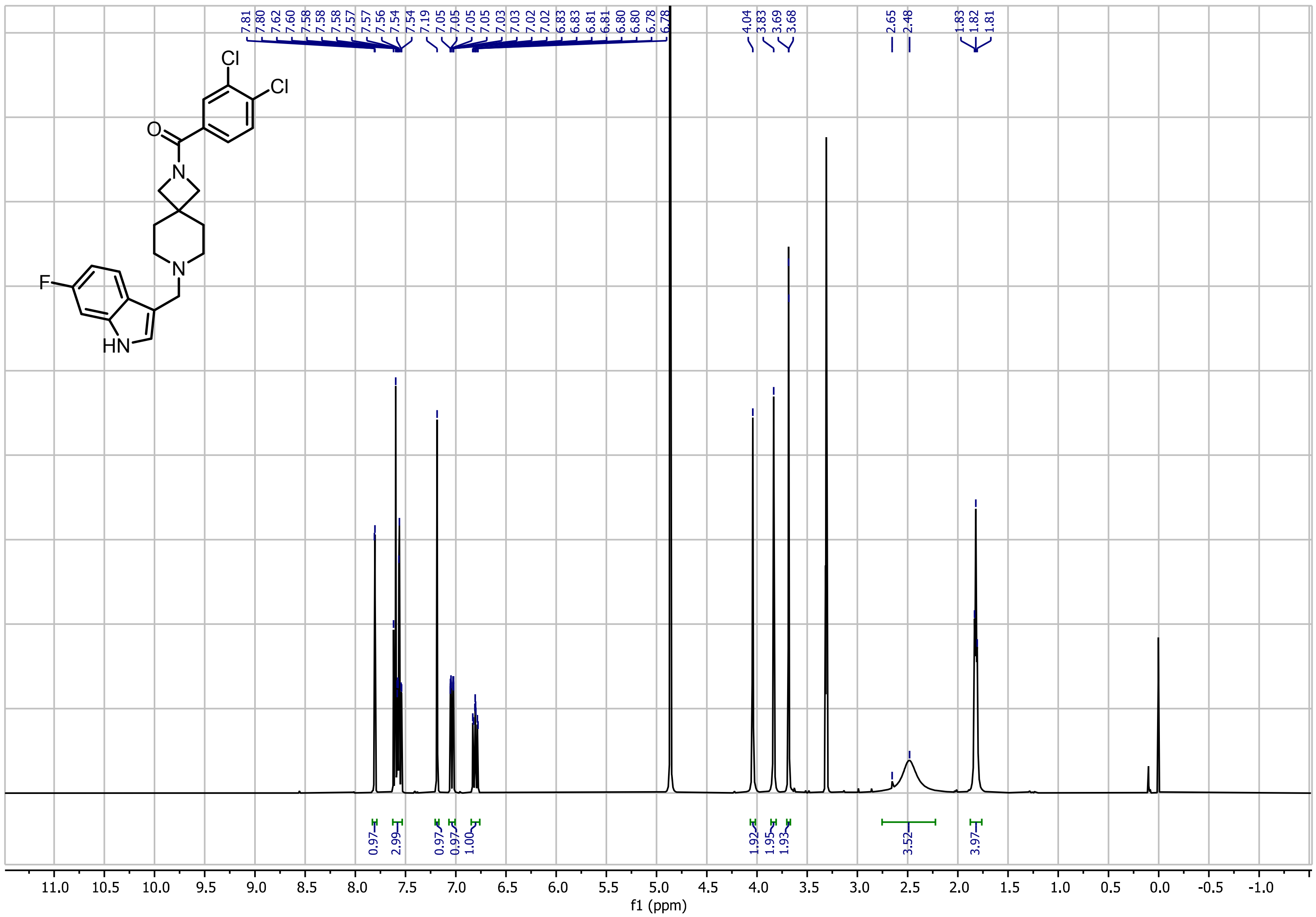

Figure S51: ^{1}H NMR spectrum of **17** in MeOD-d_4.

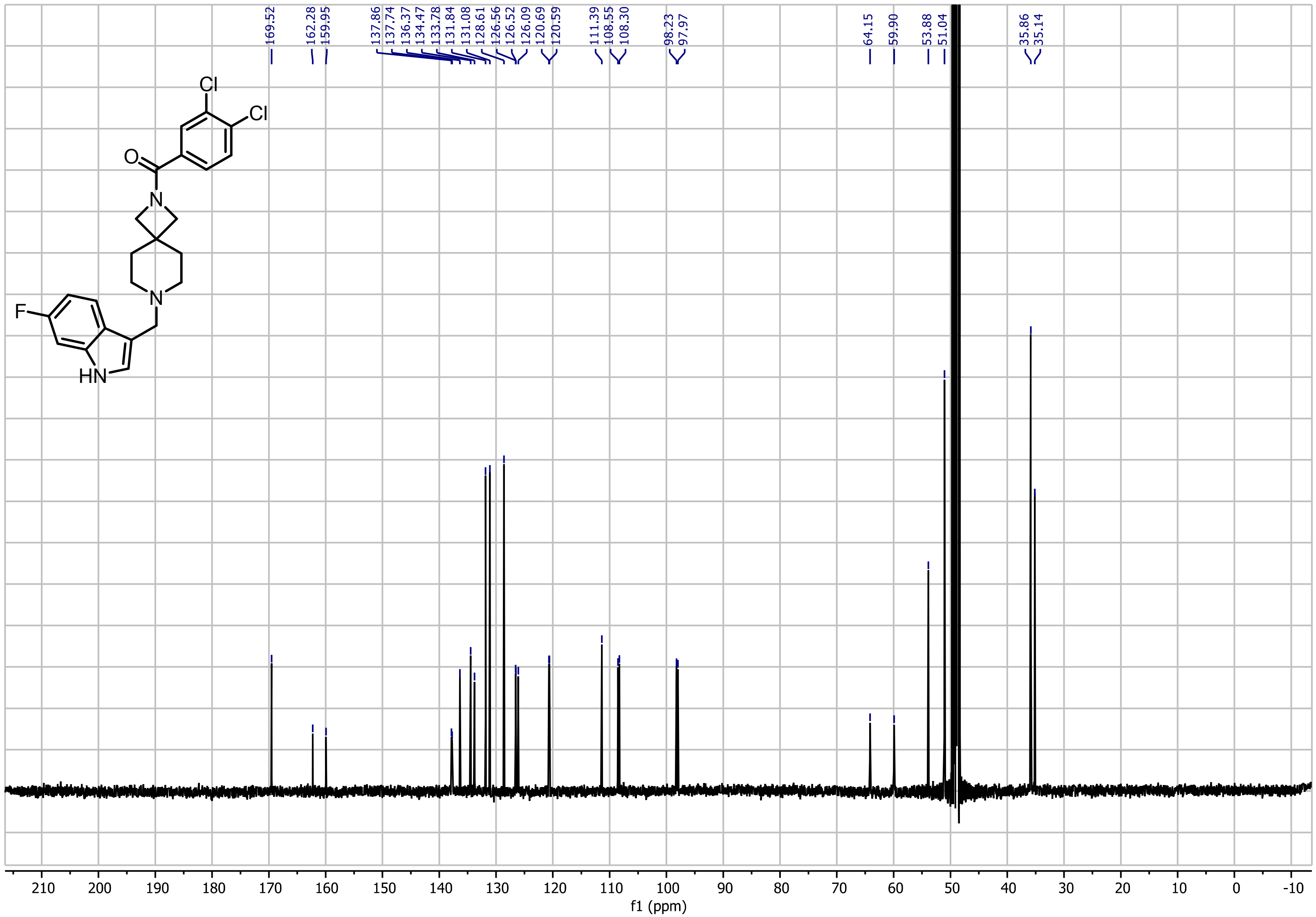

Figure S52: ^{13}C NMR spectrum of **17** in MeOD-d_4.

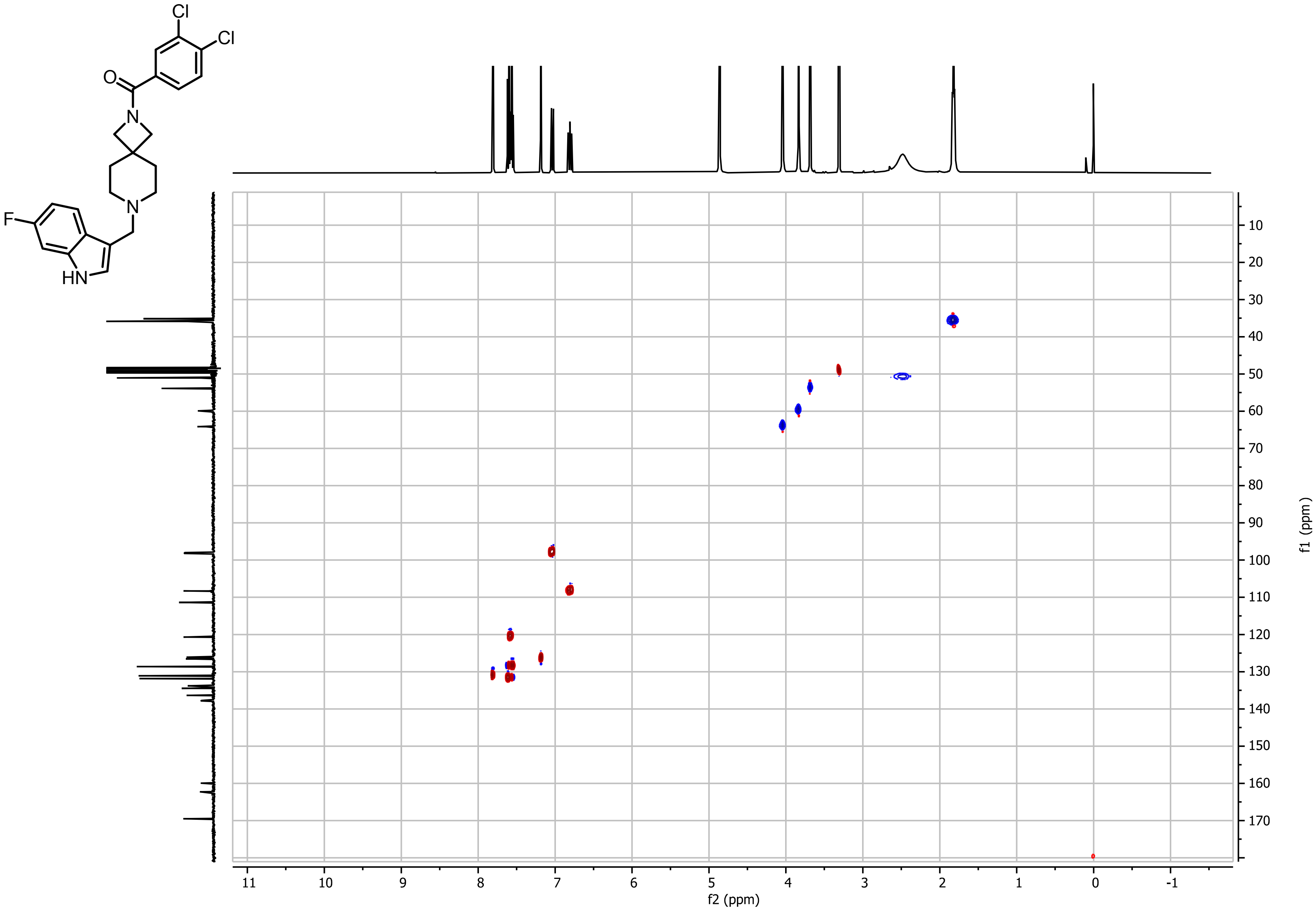

Figure S53: HSQC spectrum of **17** in MeOD-d_4.

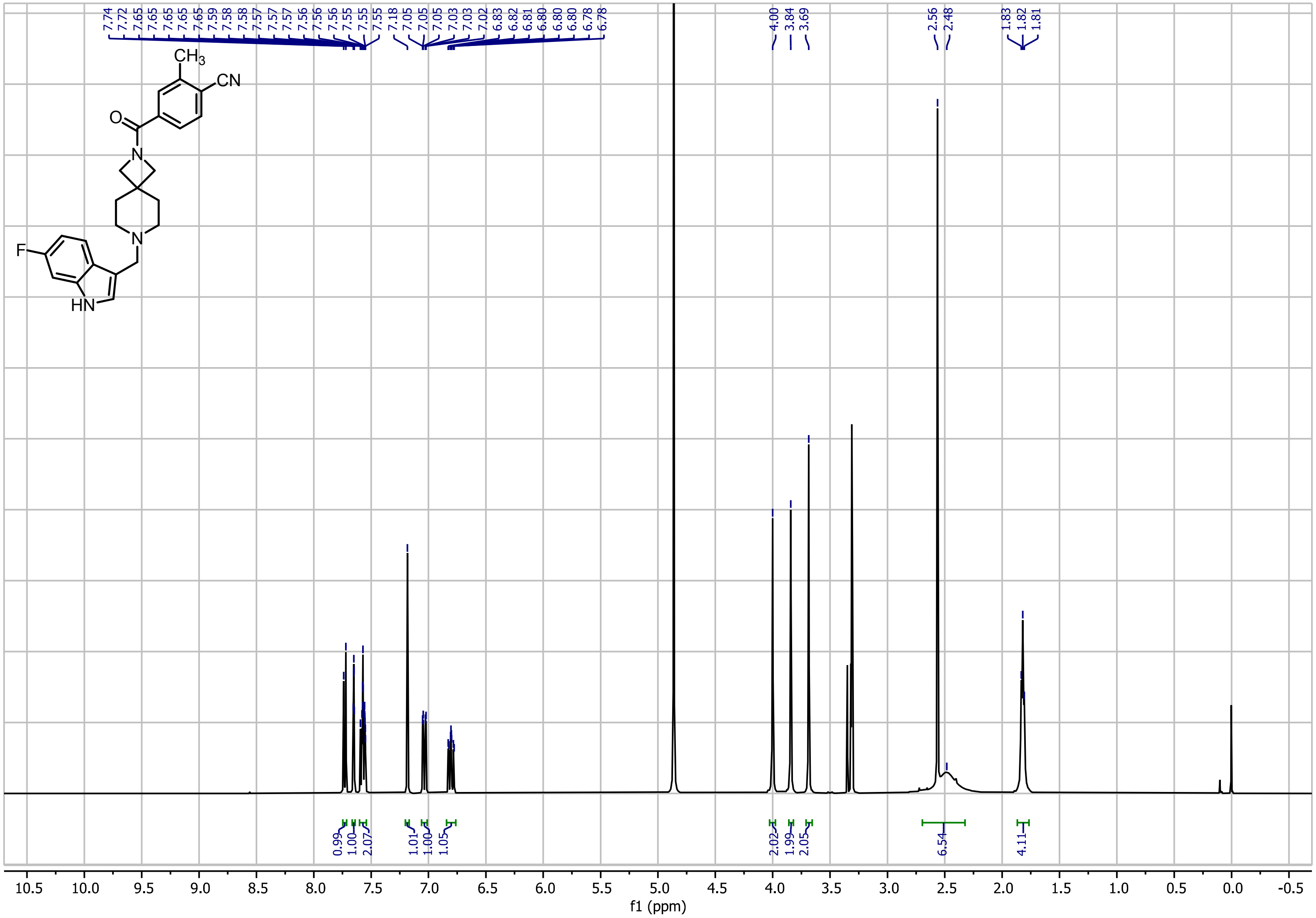

Figure S54: ^{1}H NMR spectrum of **18** in MeOD-d_4.

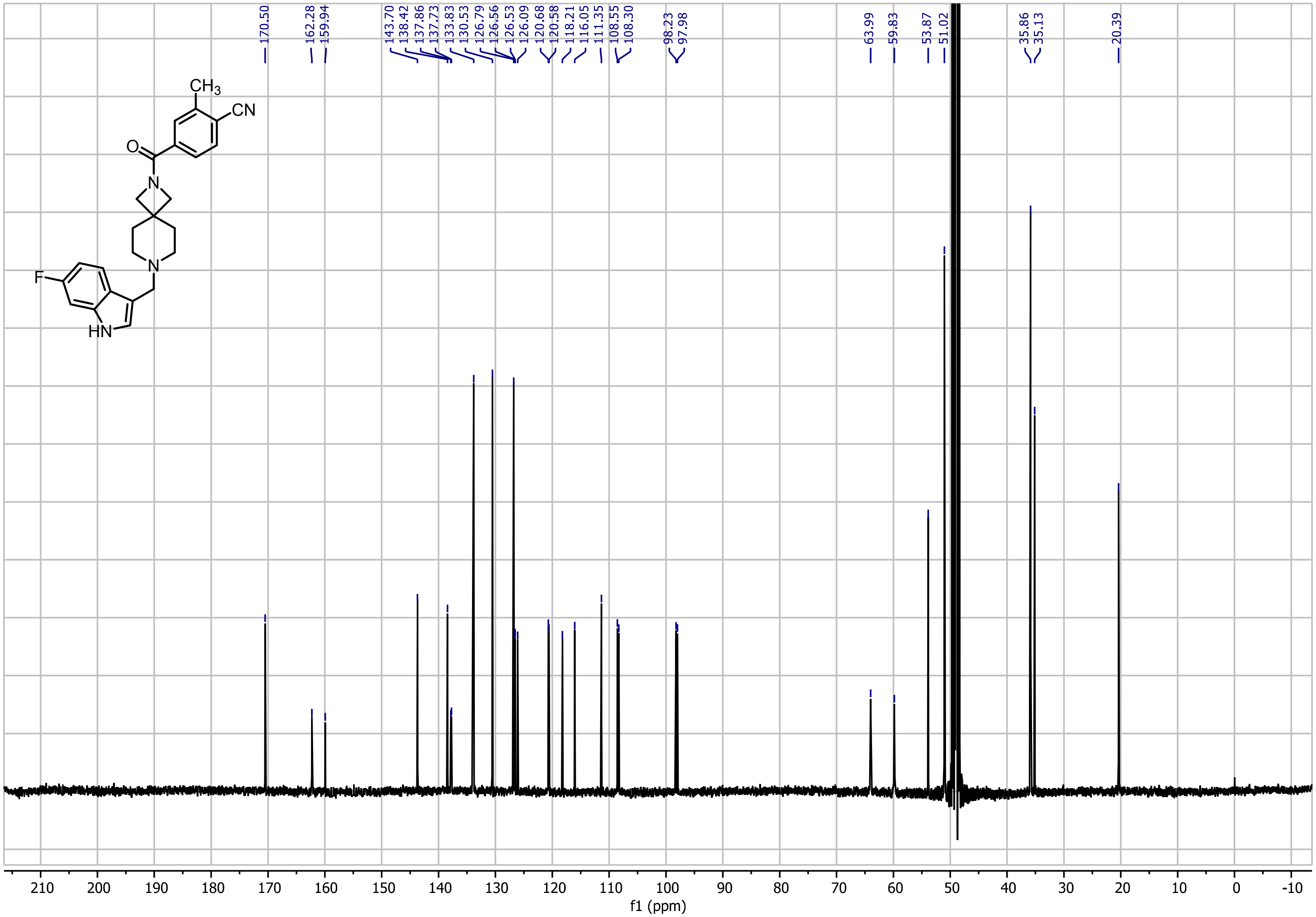

Figure S55: ^{13}C NMR spectrum of **18** in MeOD-d_4.

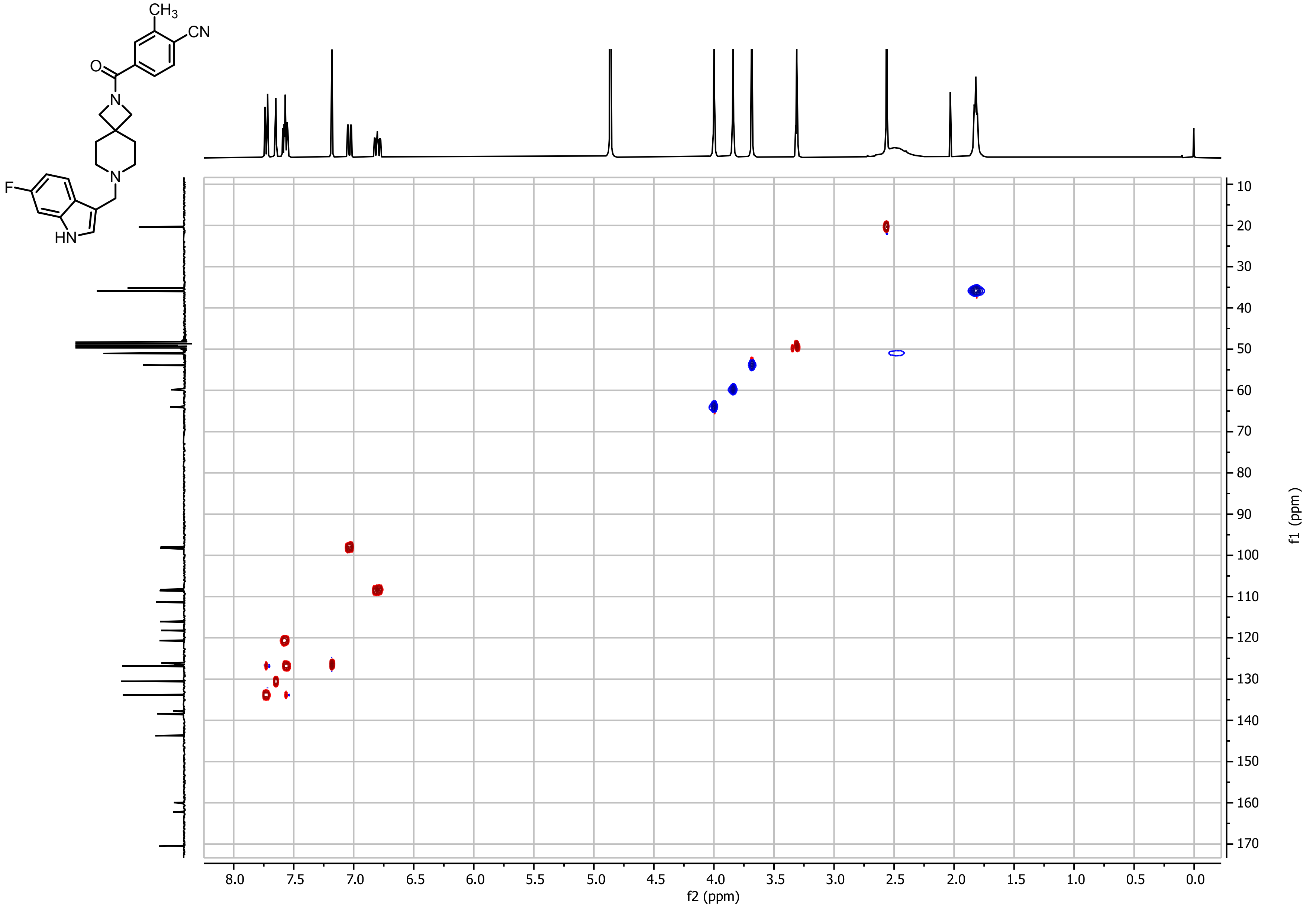

Figure S56: HSQC spectrum of **18** in MeOD-d_4.

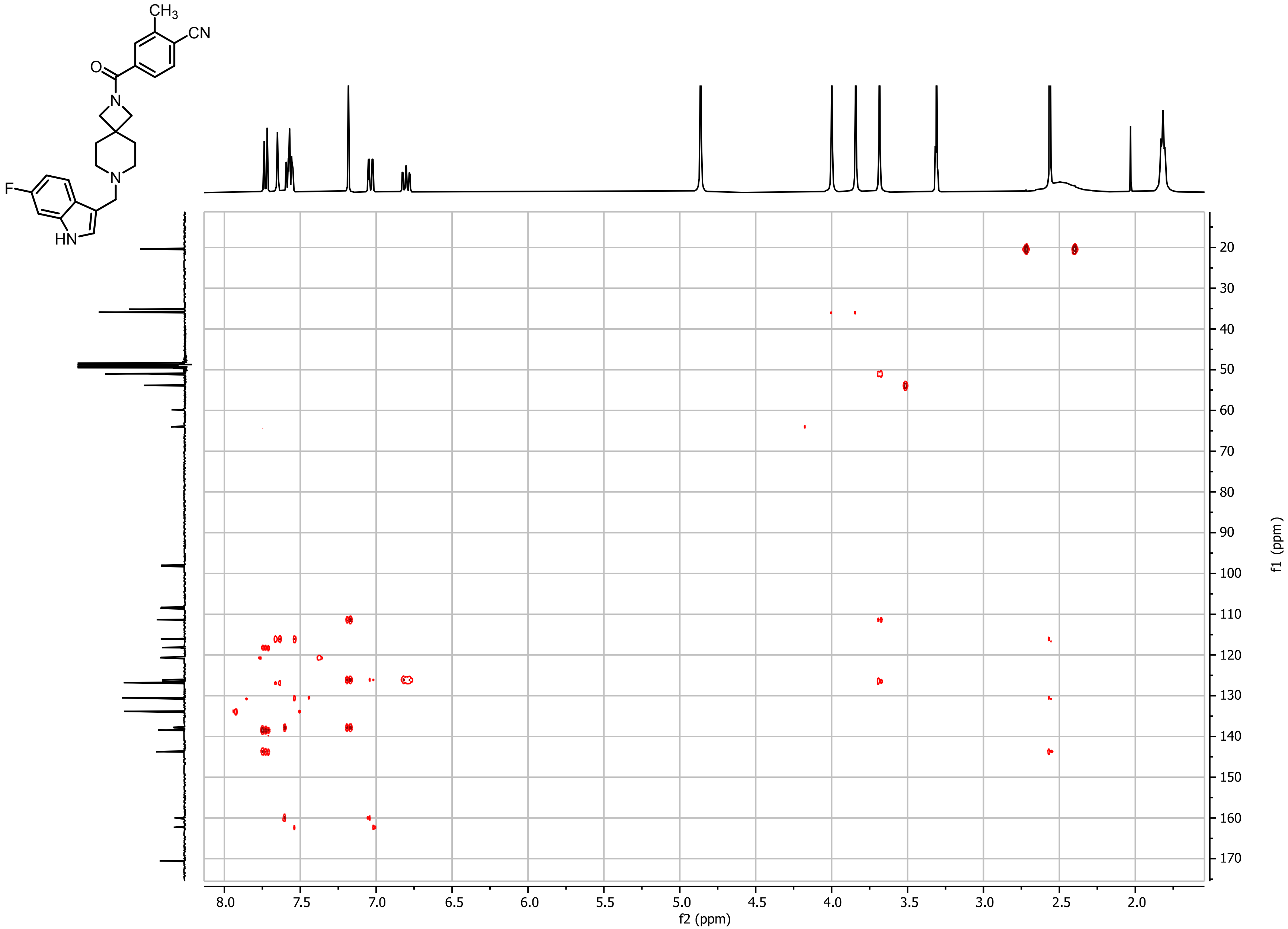

Figure S57: HMBC spectrum of **18** in MeOD-d_4.

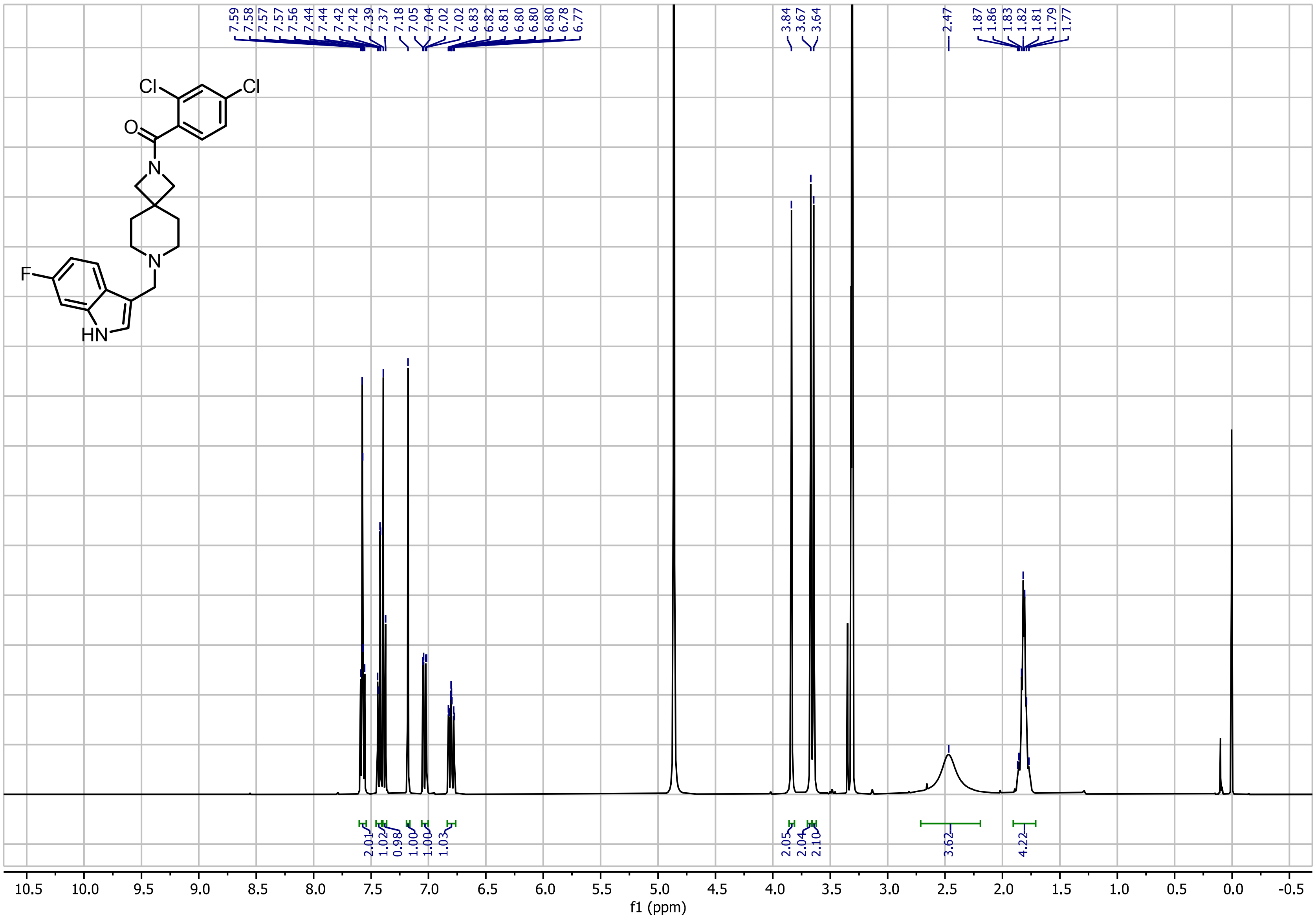

Figure S58: ^{1}H NMR spectrum of **19** in MeOD-d_4.

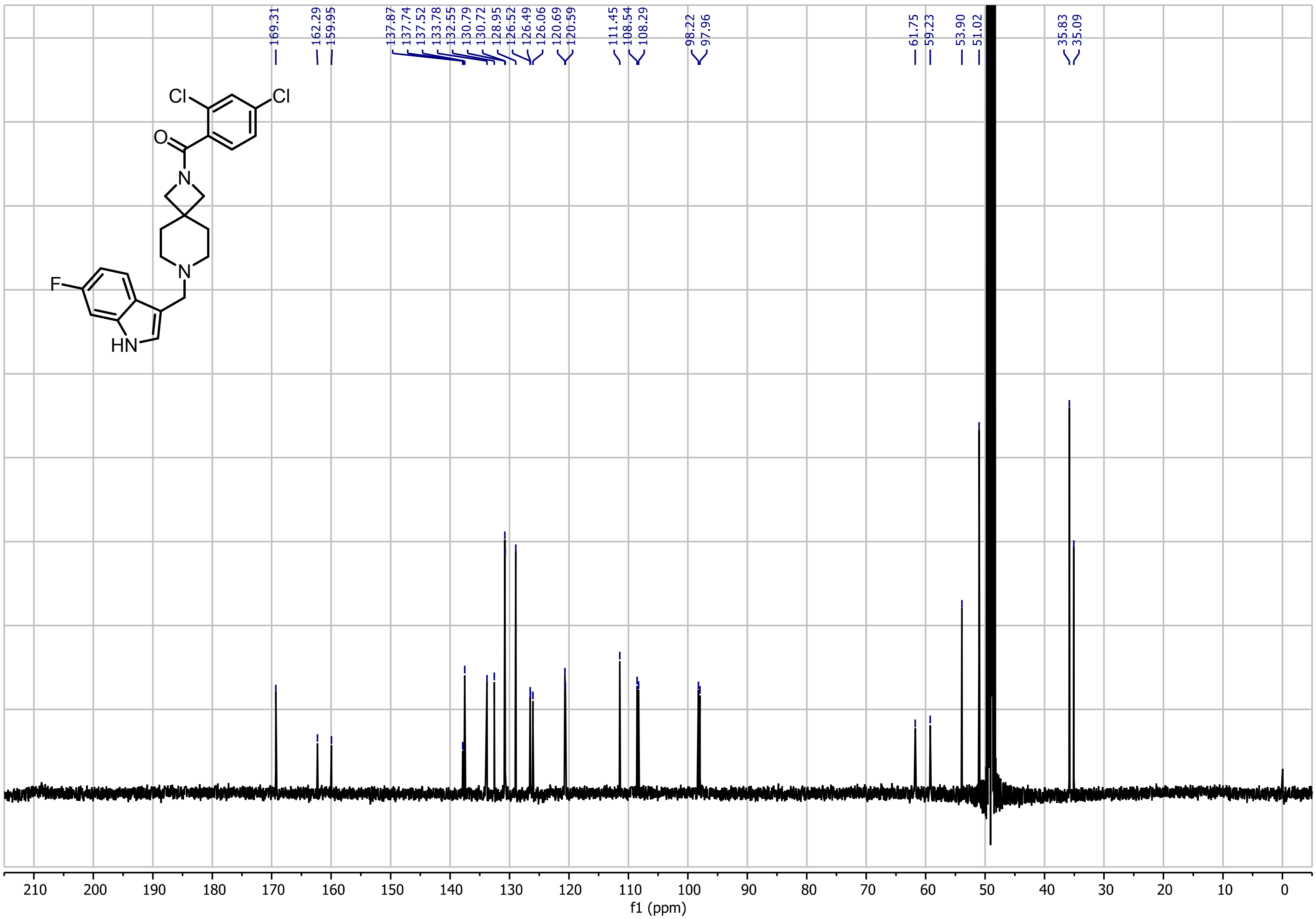

Figure S59: ^{13}C NMR spectrum of **19** in MeOD-d_4.

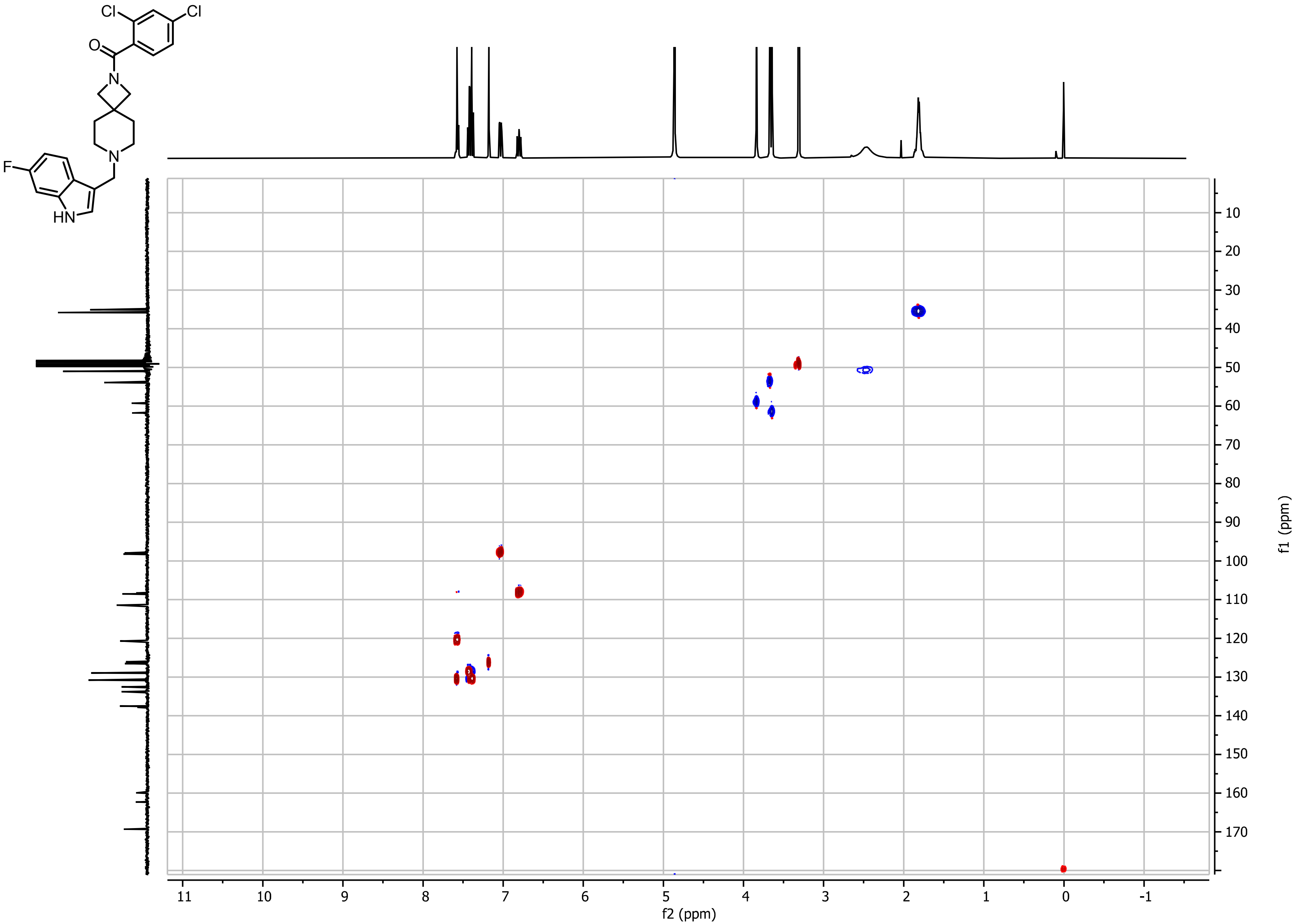

Figure S60: HSQC spectrum of **19** in MeOD-d_4.

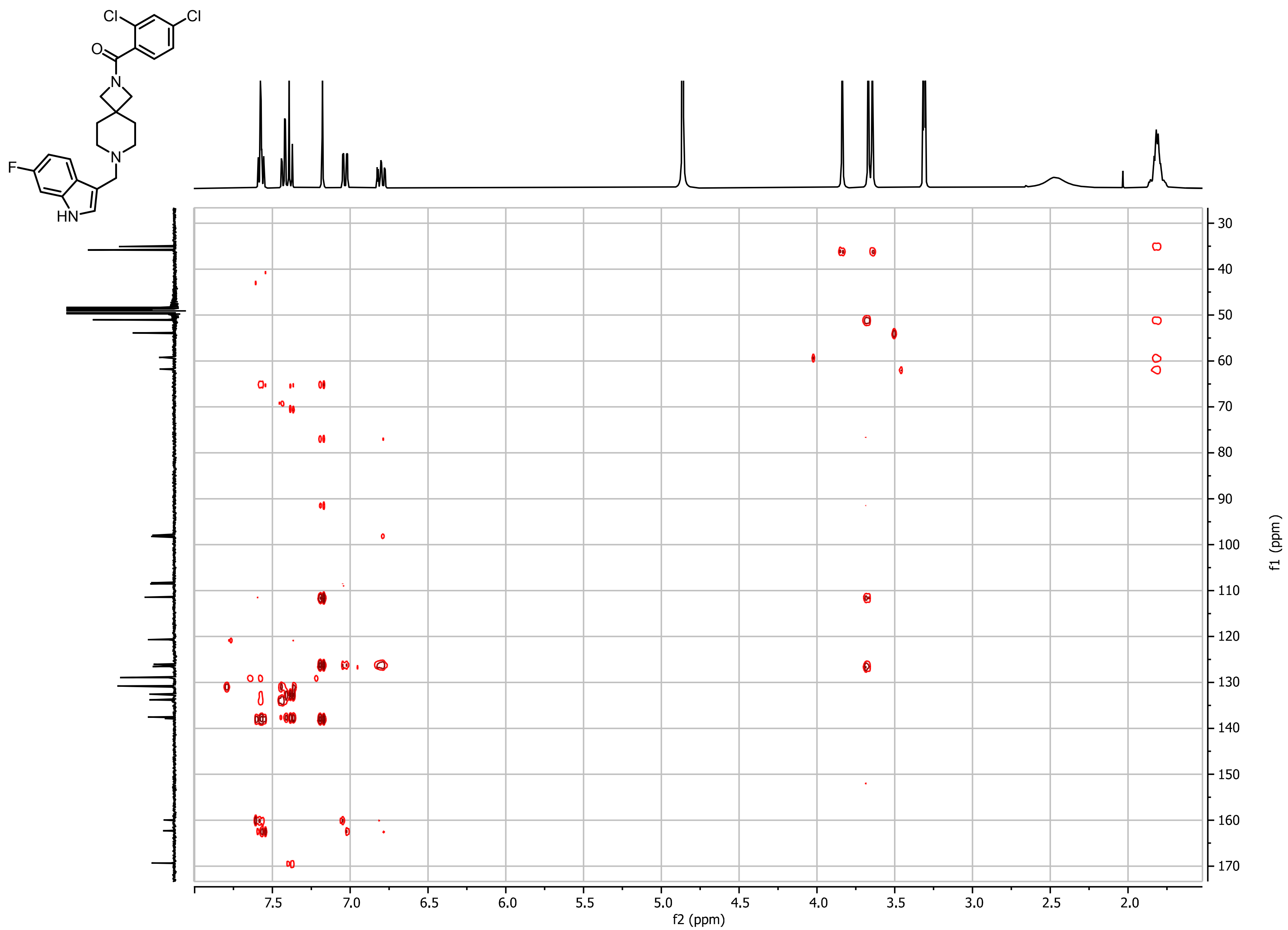

Figure S61: HMBC spectrum of **19** in MeOD-d_4.

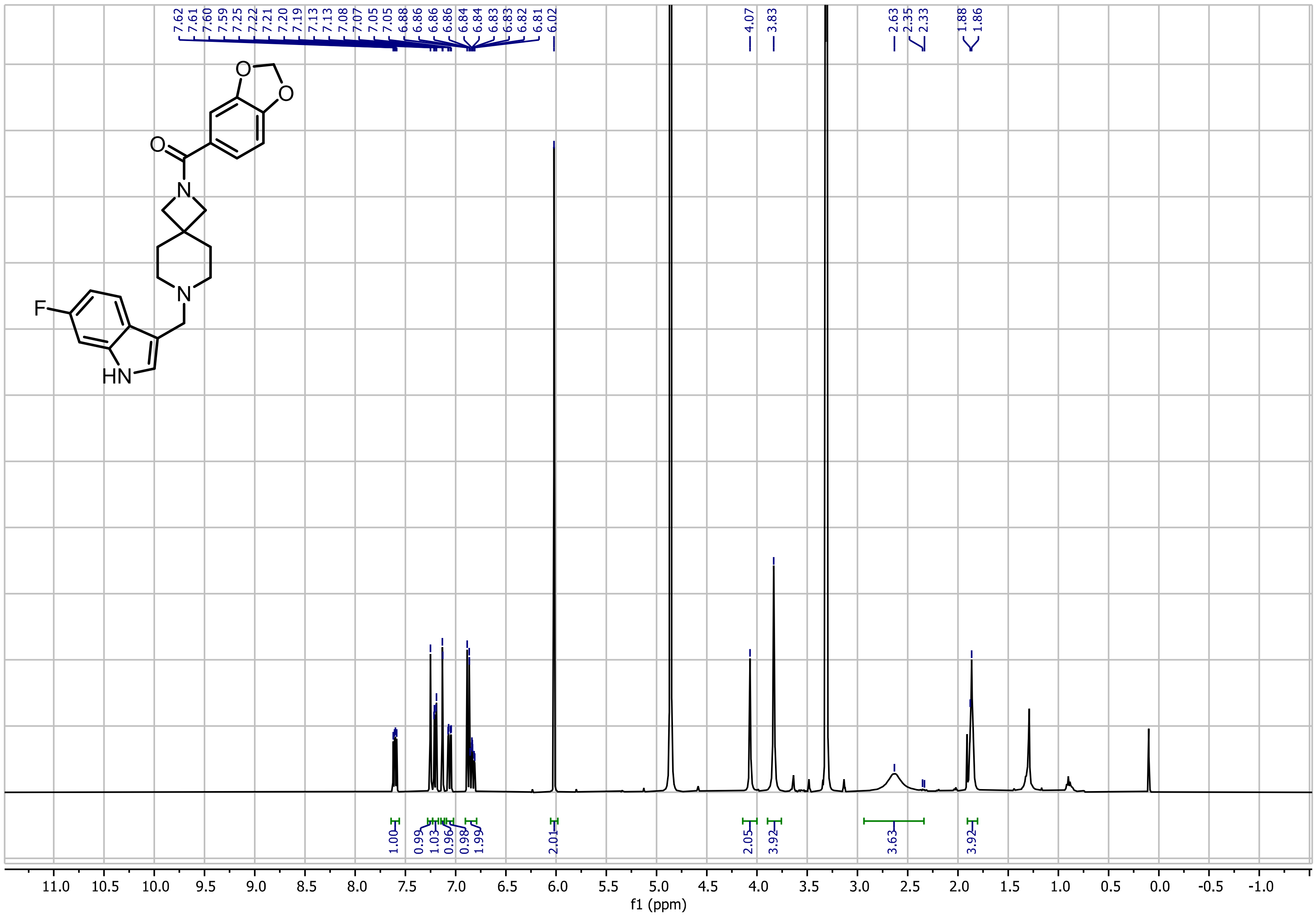

Figure S62: ^{1}H NMR spectrum of **20** in MeOD-d_4.

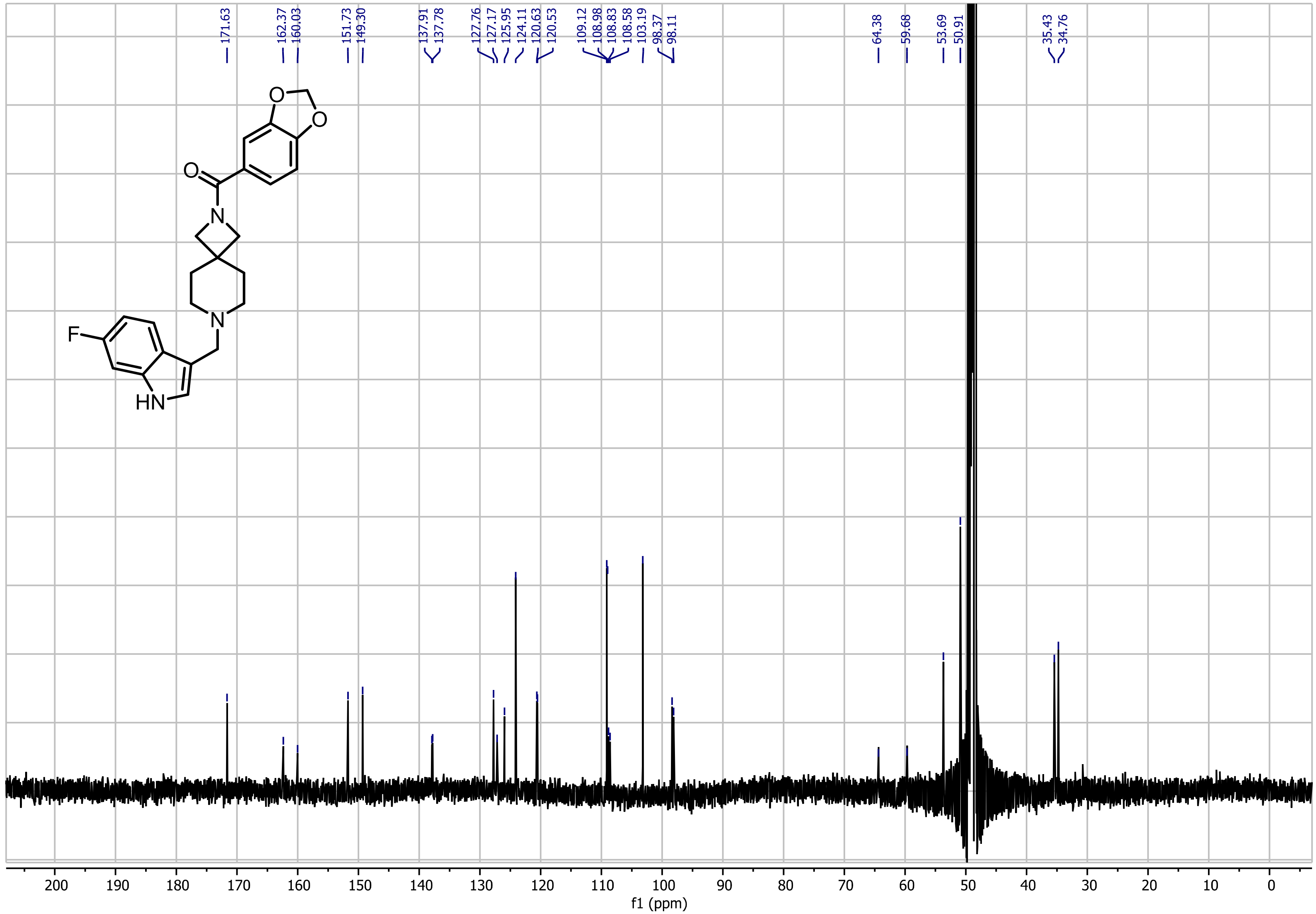

Figure S63: ^{13}C NMR spectrum of **20** in MeOD-d_4.

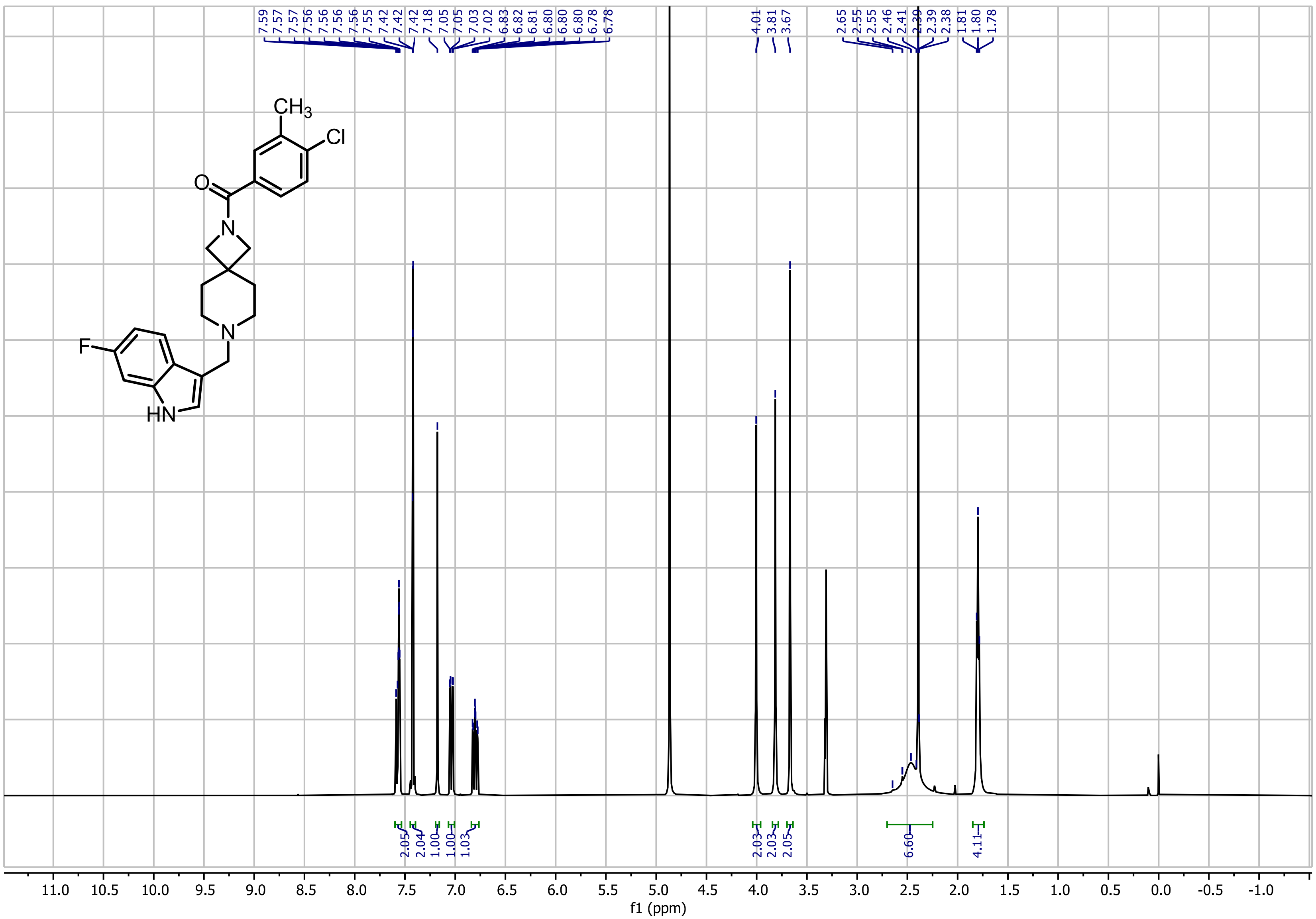

Figure S64: ^{1}H NMR spectrum of **21** in MeOD-d_4.

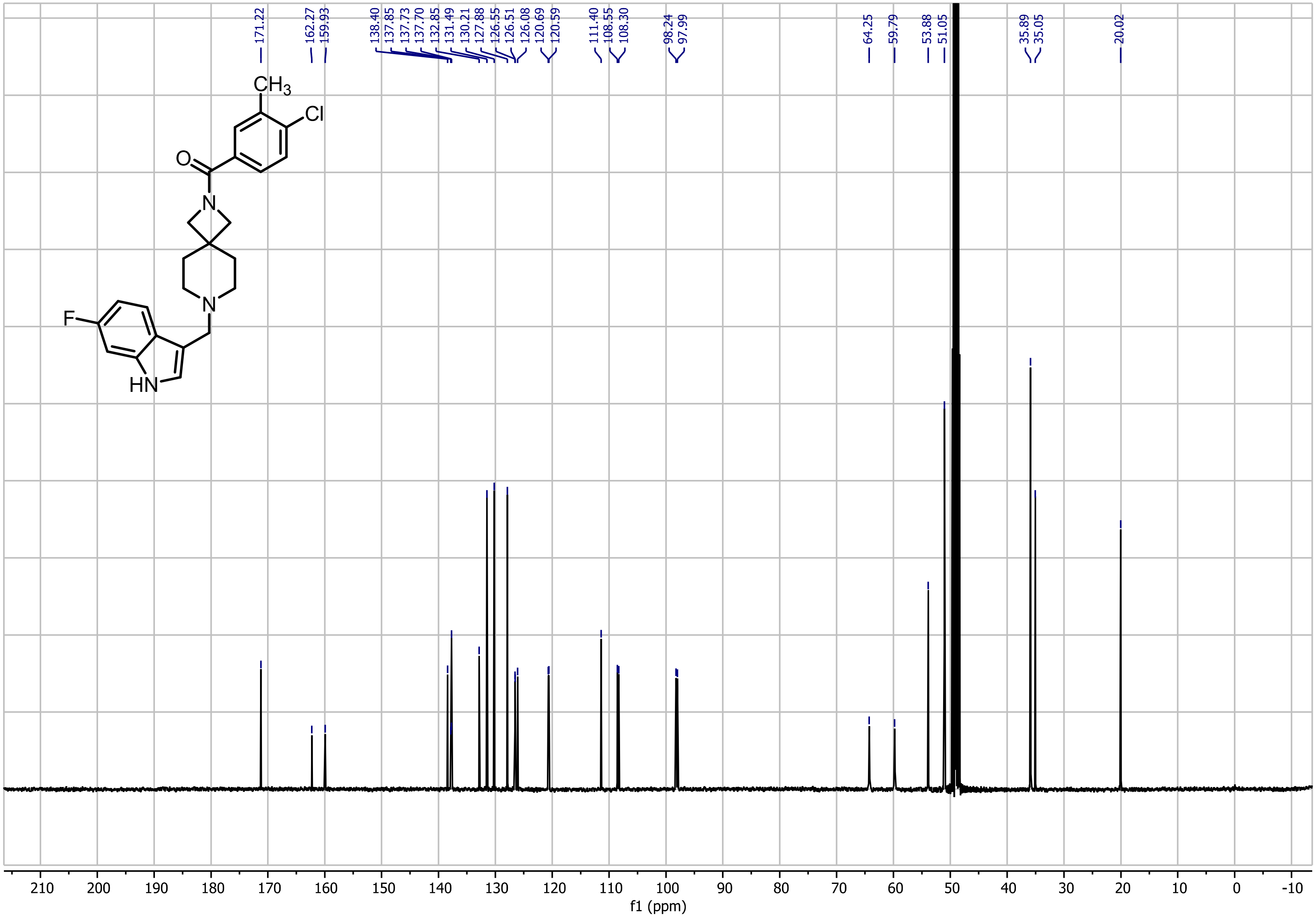

Figure S65: ^{13}C NMR spectrum of **21** in MeOD-d_4.

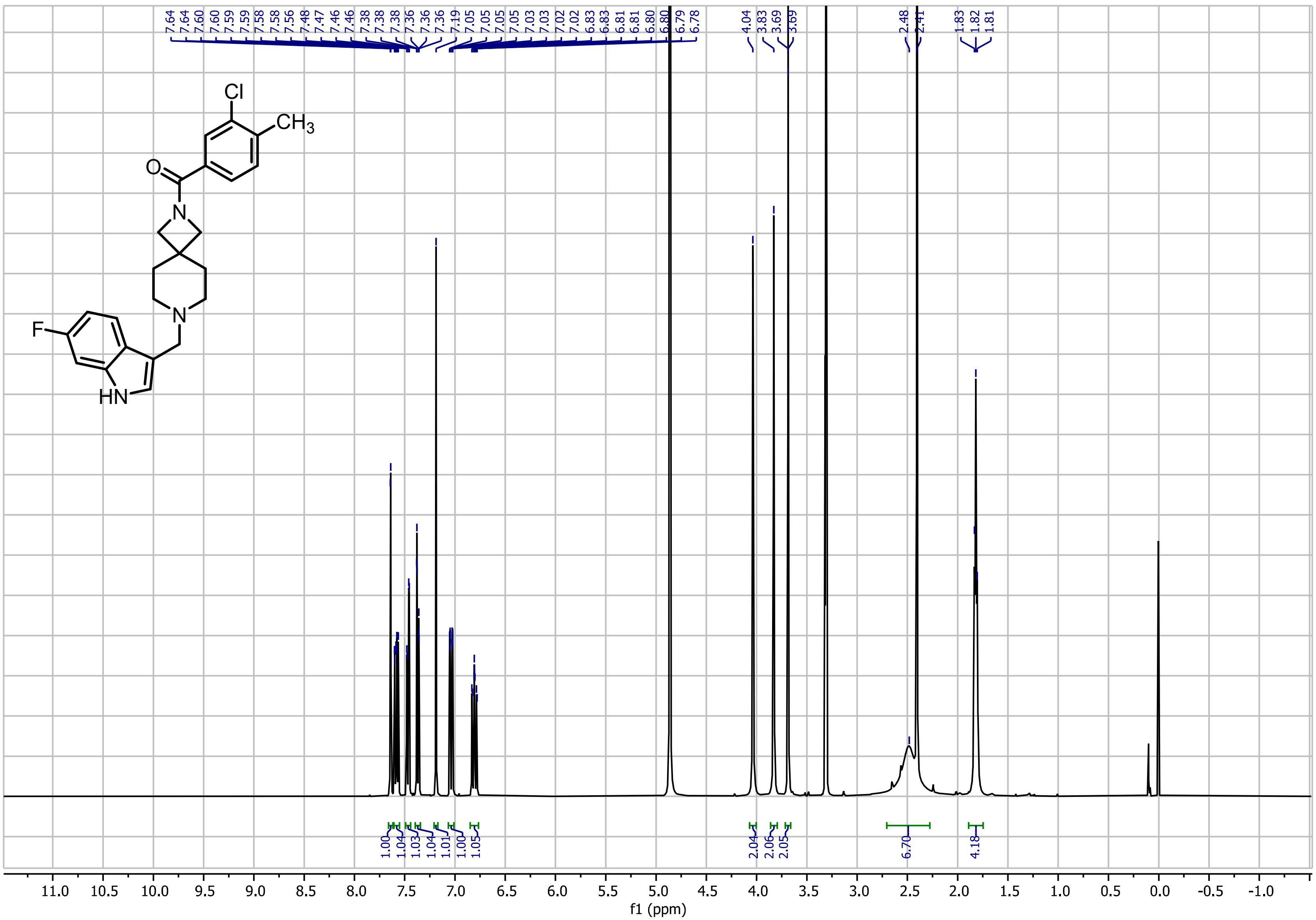

Figure S66: ^{1}H NMR spectrum of **22** in MeOD-d_4.

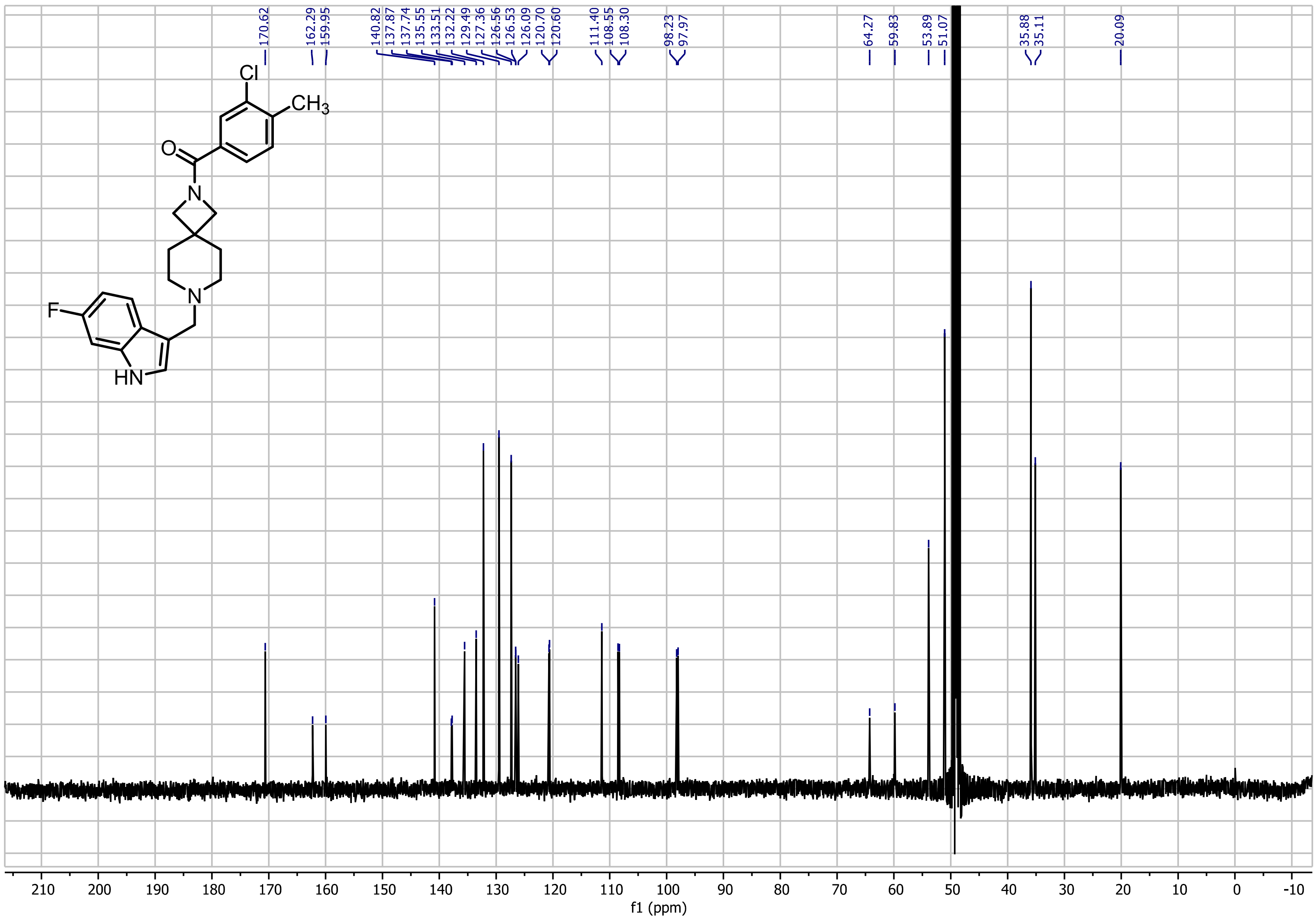

Figure S67: ^{13}C NMR spectrum of **22** in MeOD-d_4.

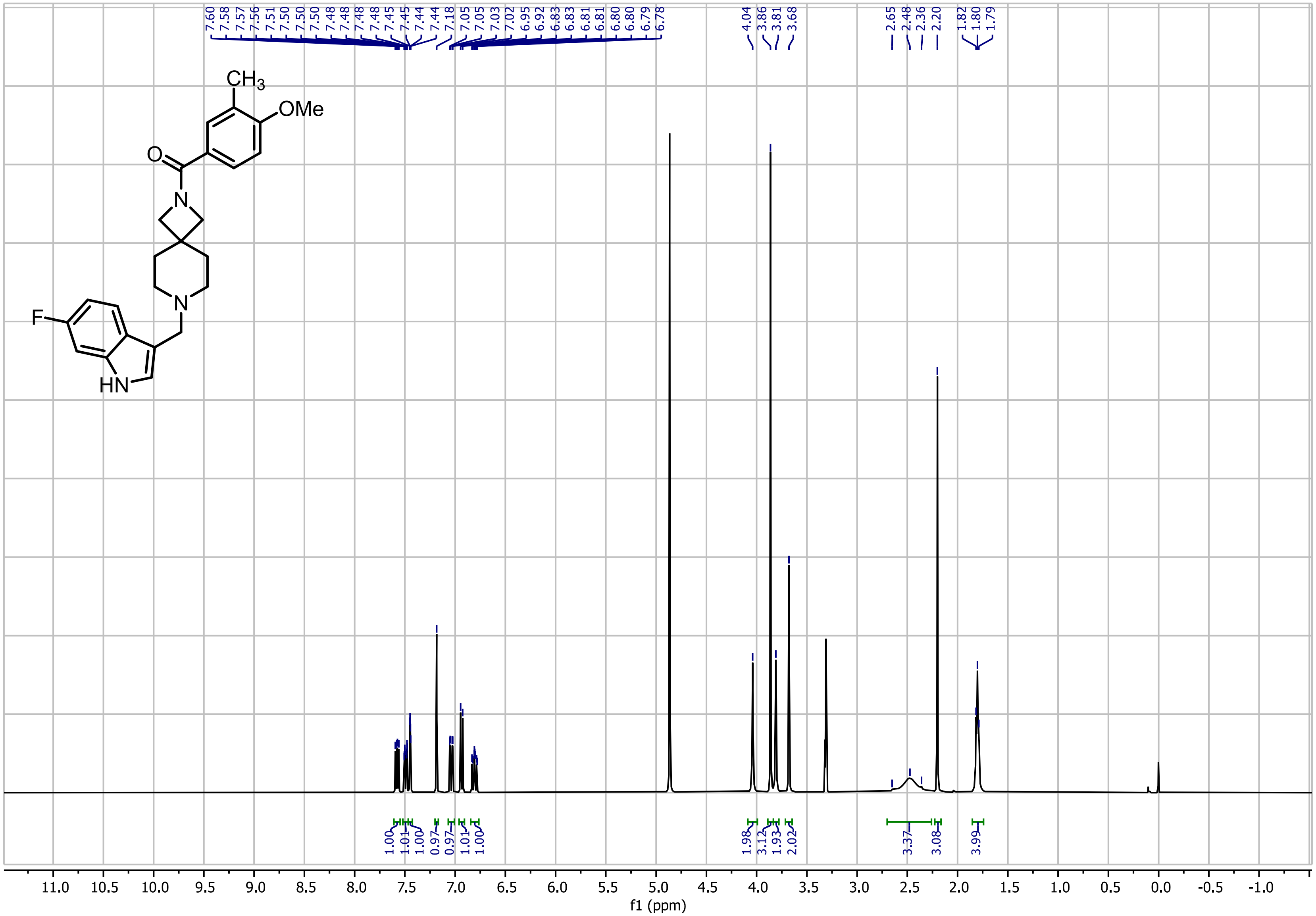

Figure S68: ^{1}H NMR spectrum of **23** in MeOD-d_4.

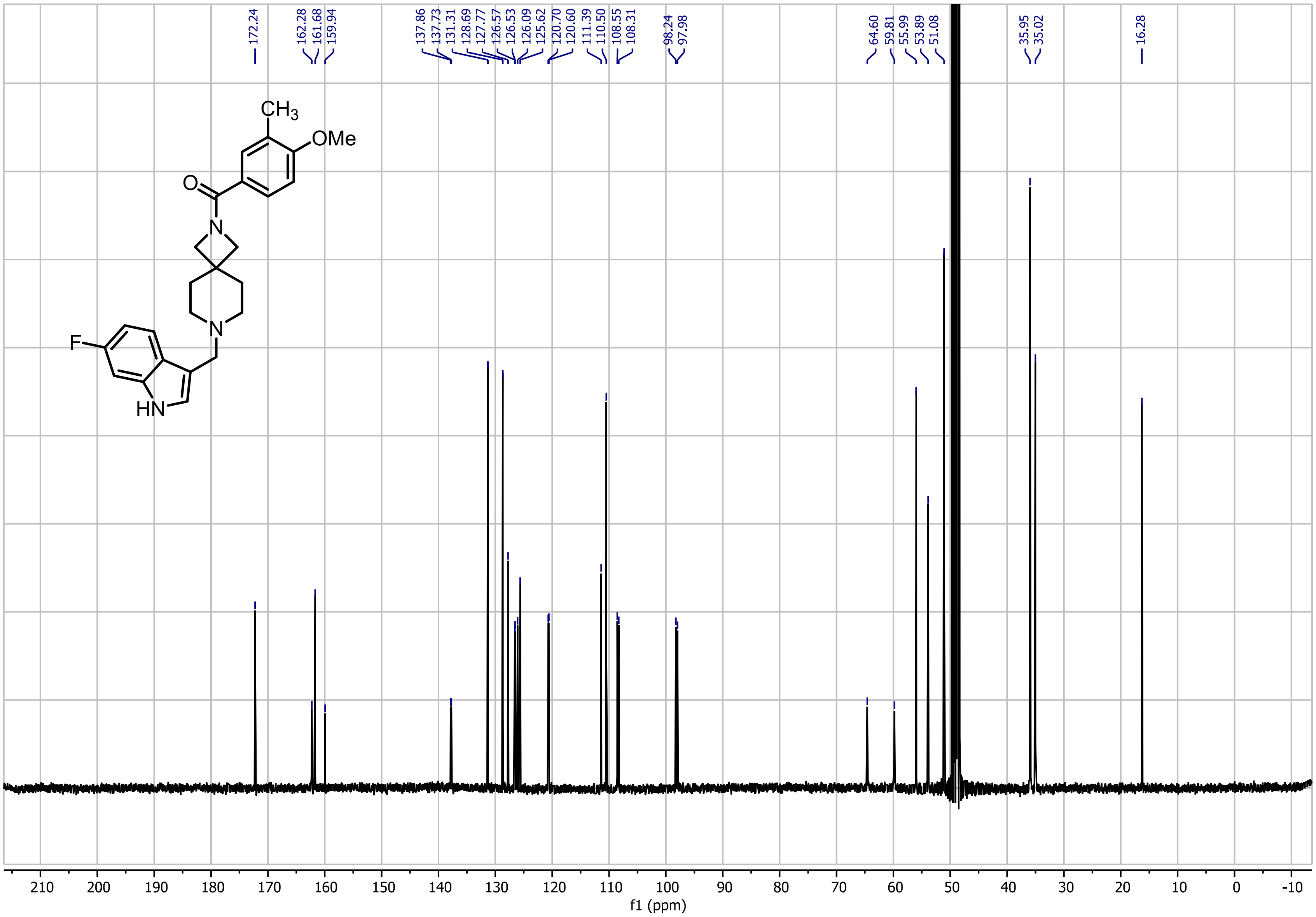

Figure S69: ^{13}C NMR spectrum of **23** in MeOD-d_4.

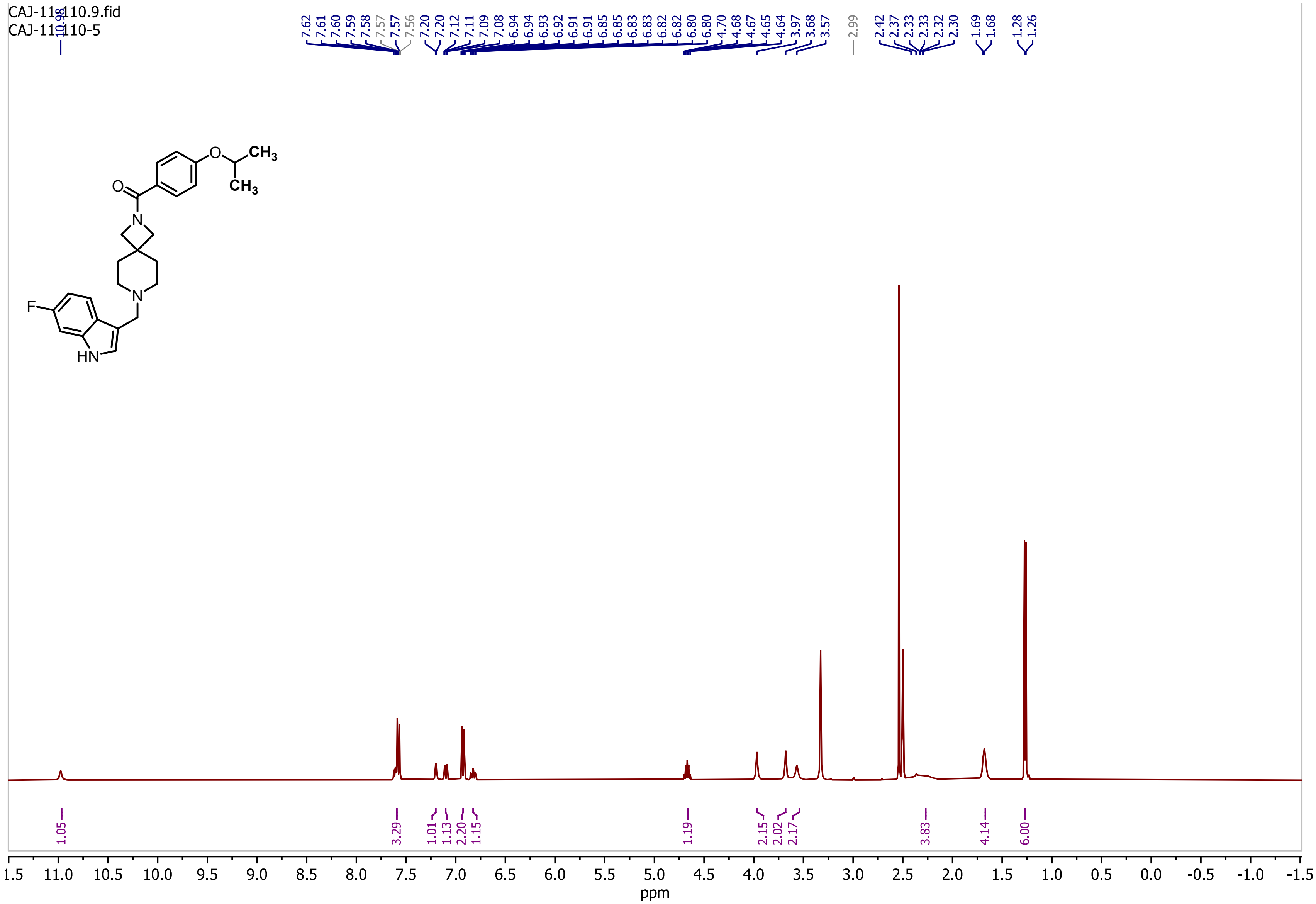

Figure S70: ^{1}H NMR spectrum of **24** in MeOD-d_4.

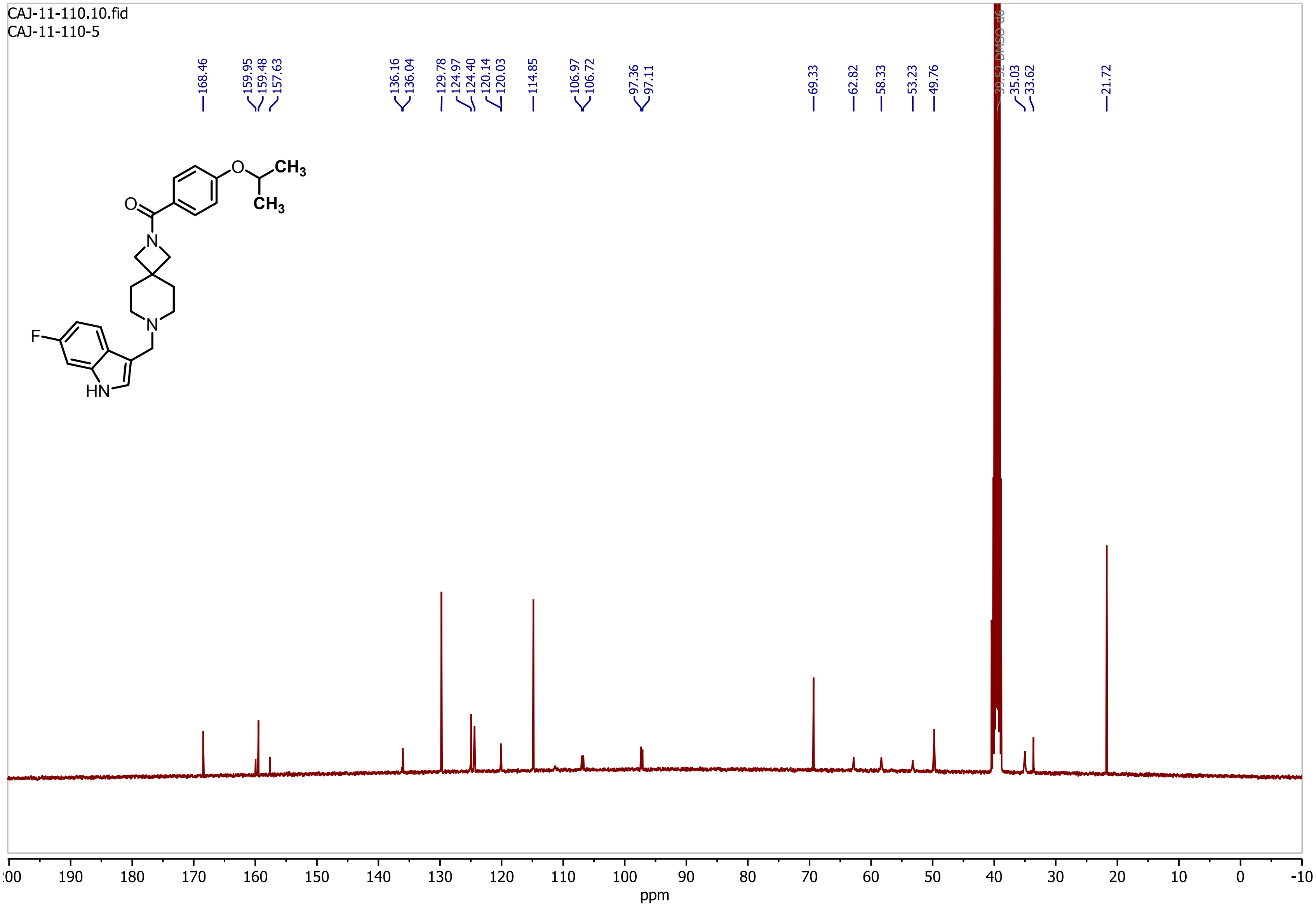

Figure S71: ^{13}C NMR spectrum of **24** in MeOD-d_4

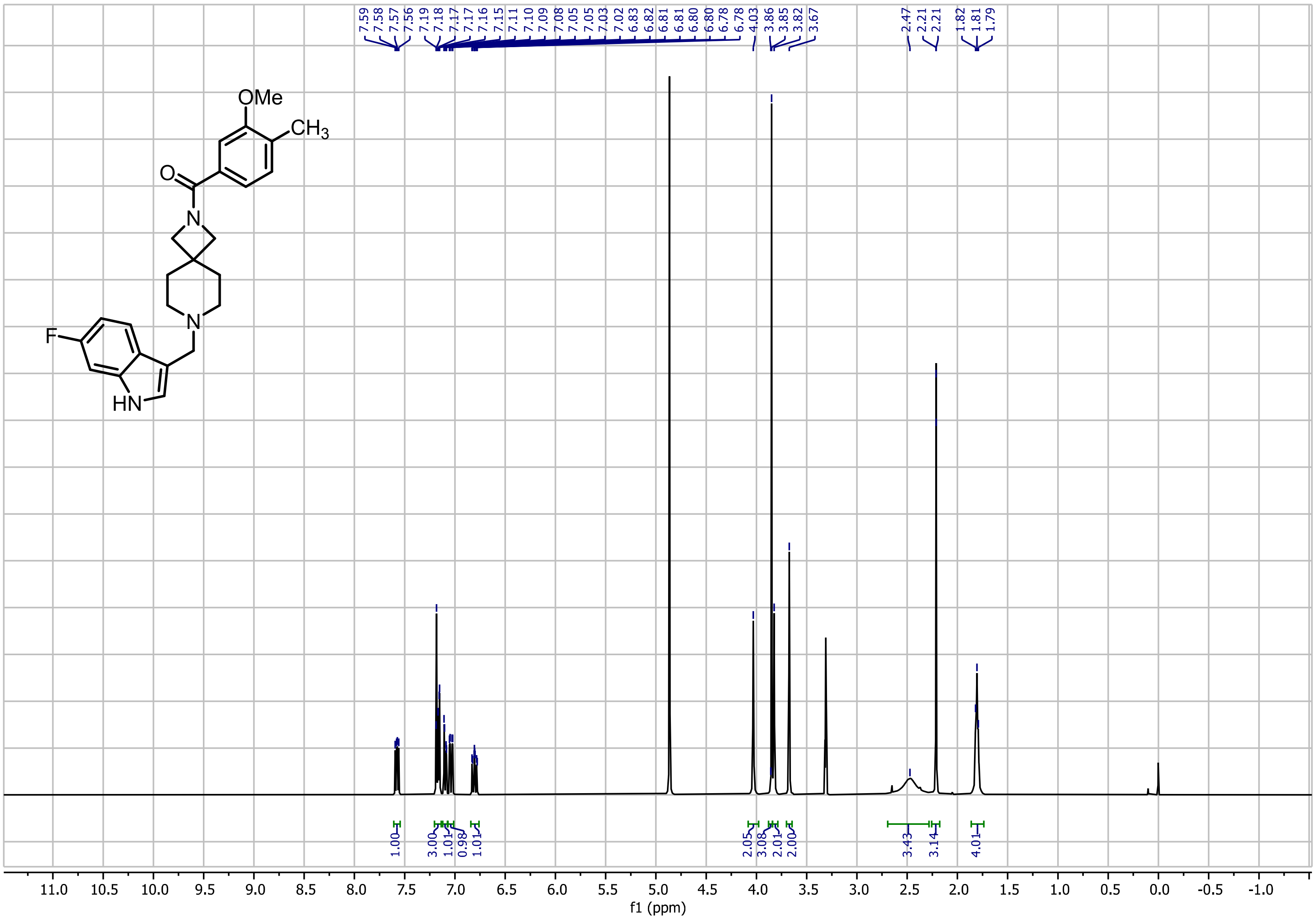

Figure S72: ^{1}H NMR spectrum of **25** in MeOD-d_4.

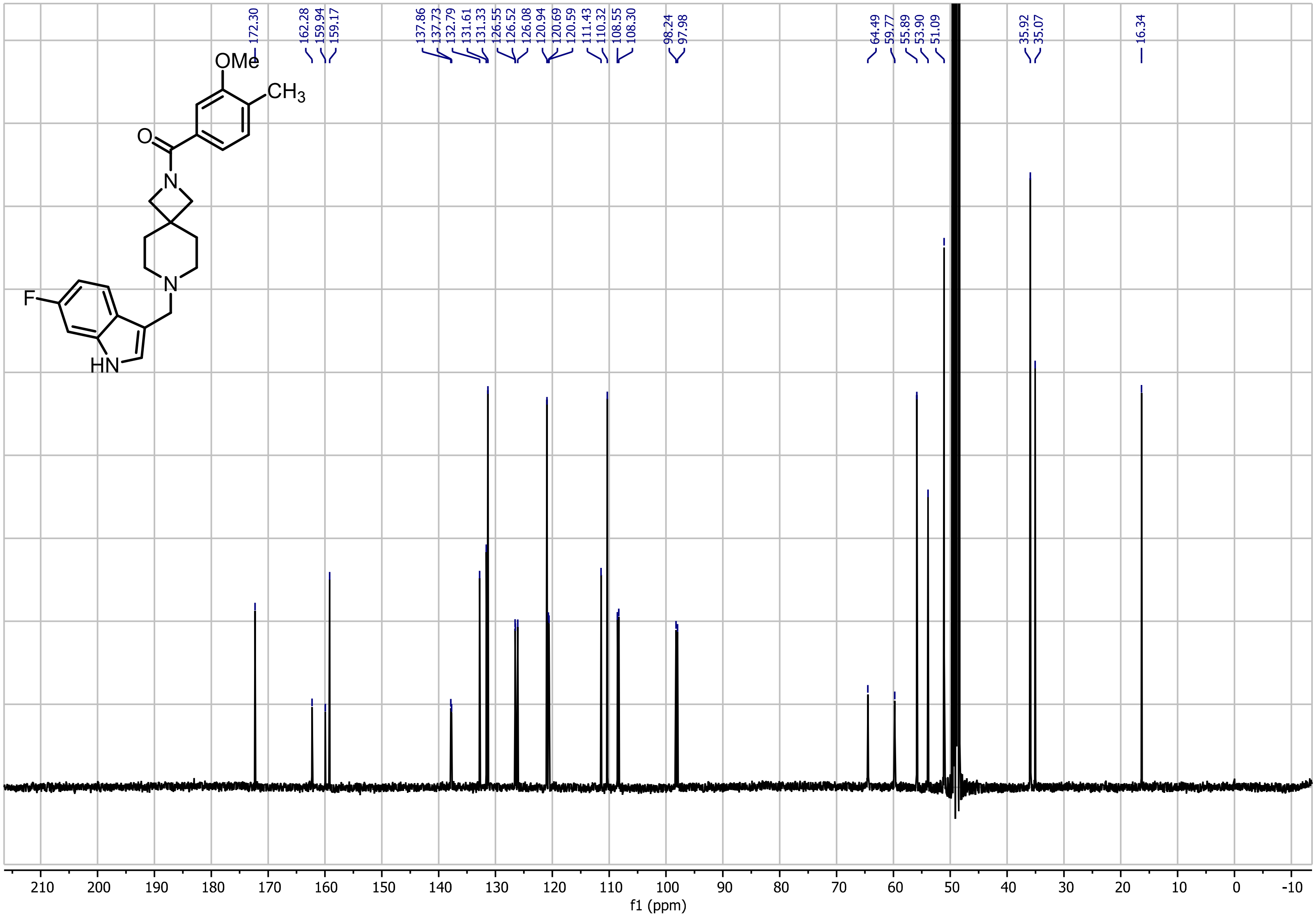

Figure S73: ^{13}C NMR spectrum of **25** in MeOD-d_4.

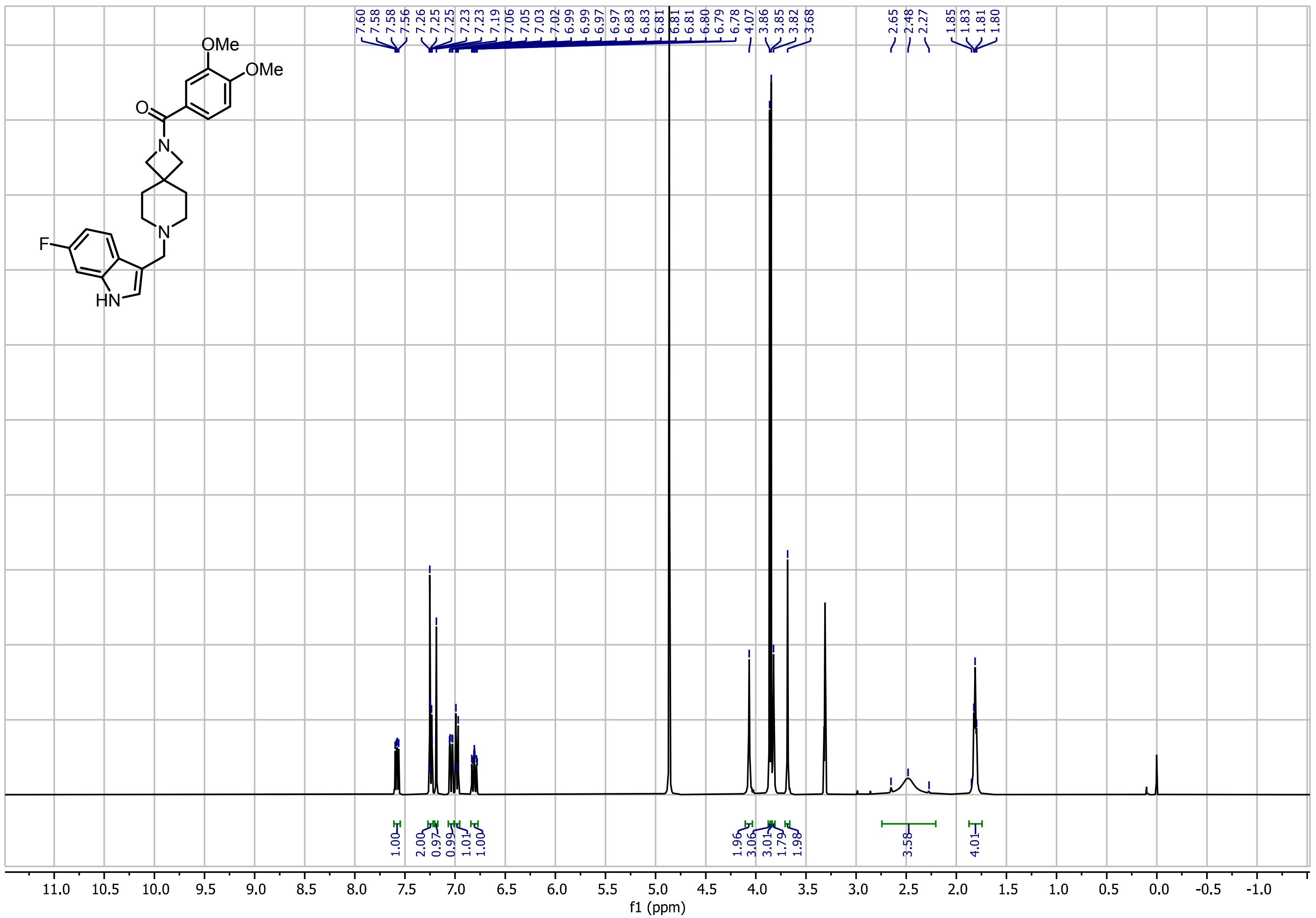

Figure S74: ¹H NMR spectrum of **26** in MeOD-d_4.

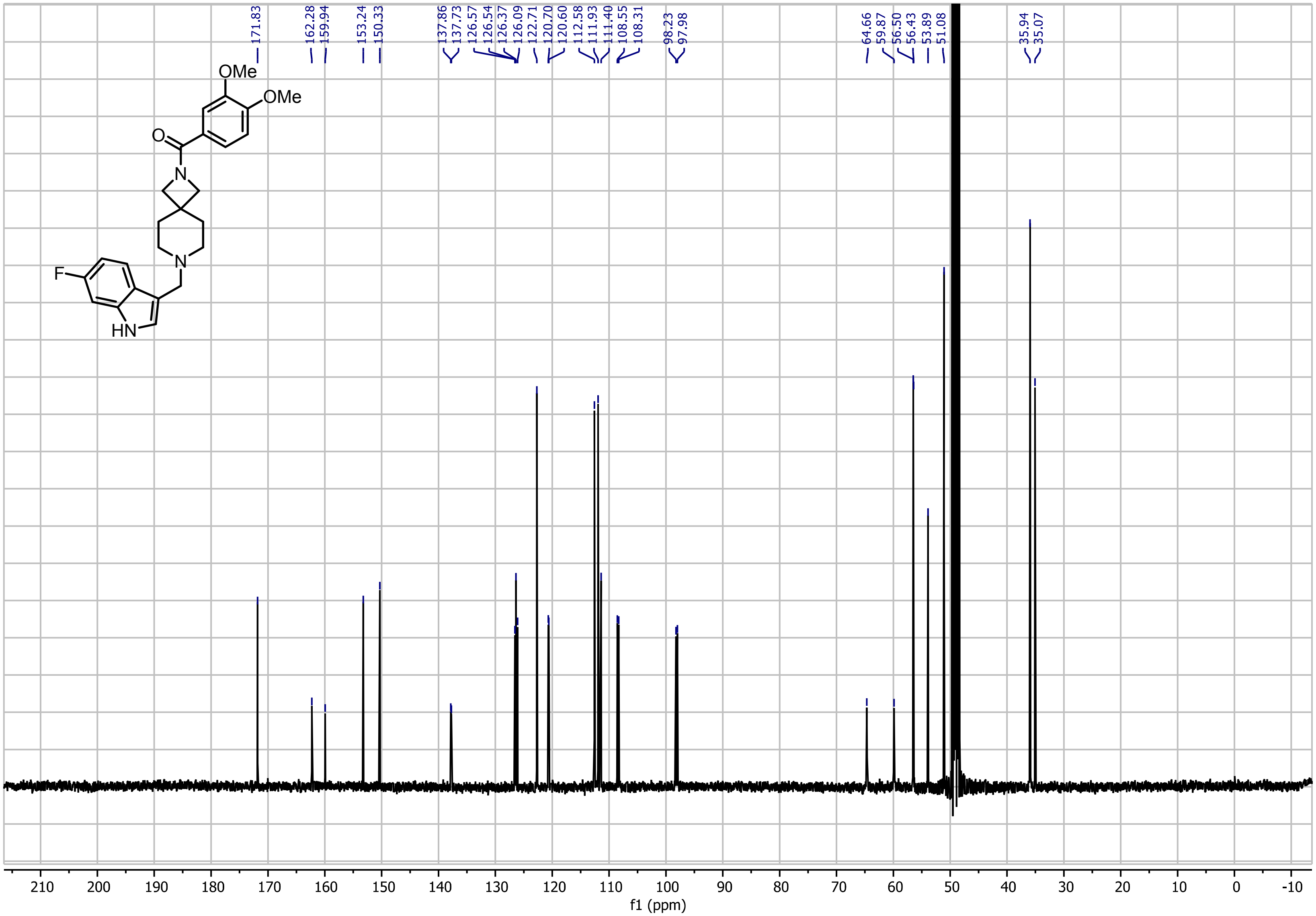

Figure S75: ^{13}C NMR spectrum of **26** in MeOD-d_4.

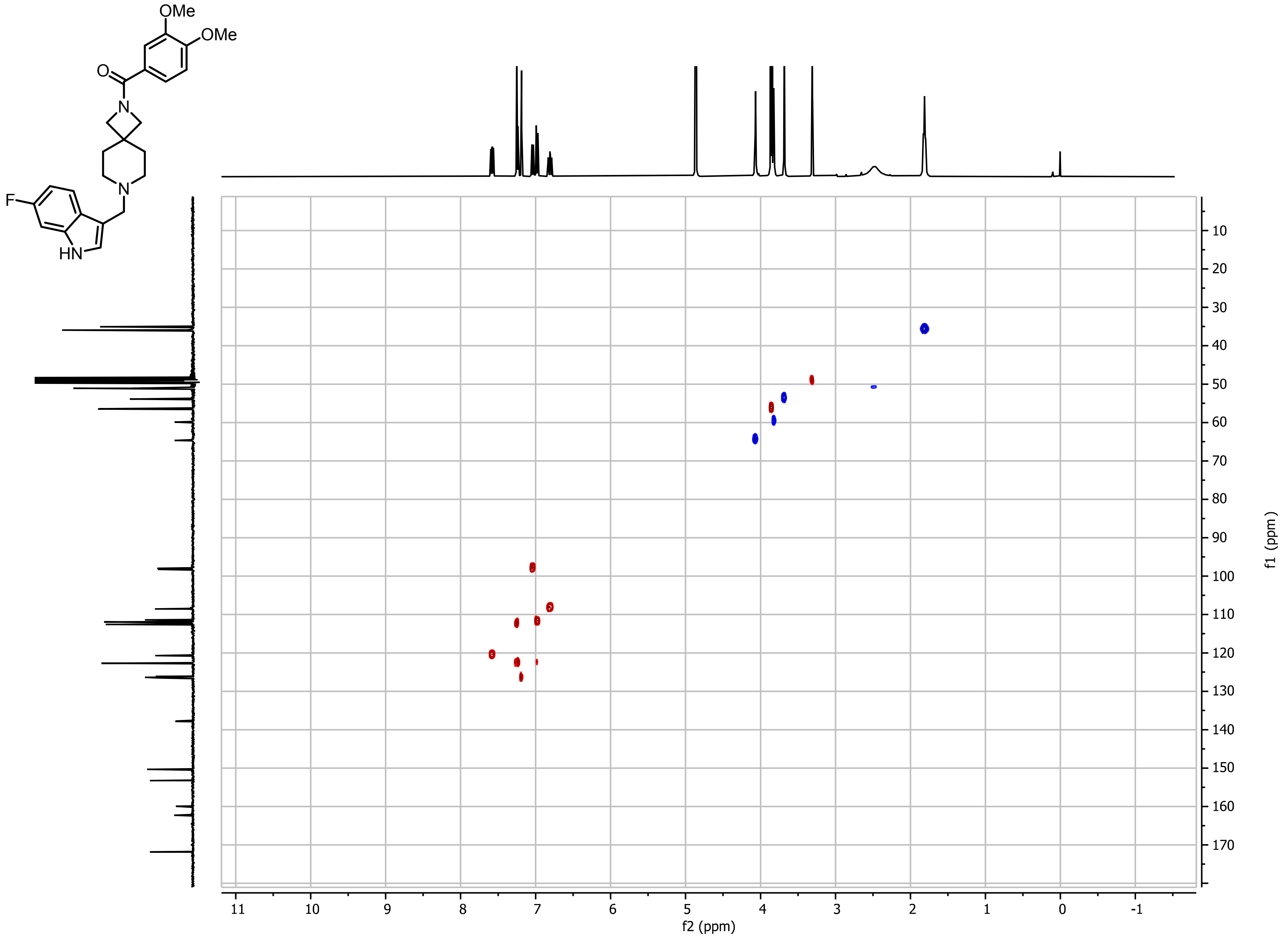

Figure S76: HSQC spectrum of **26** in MeOD-d_4.

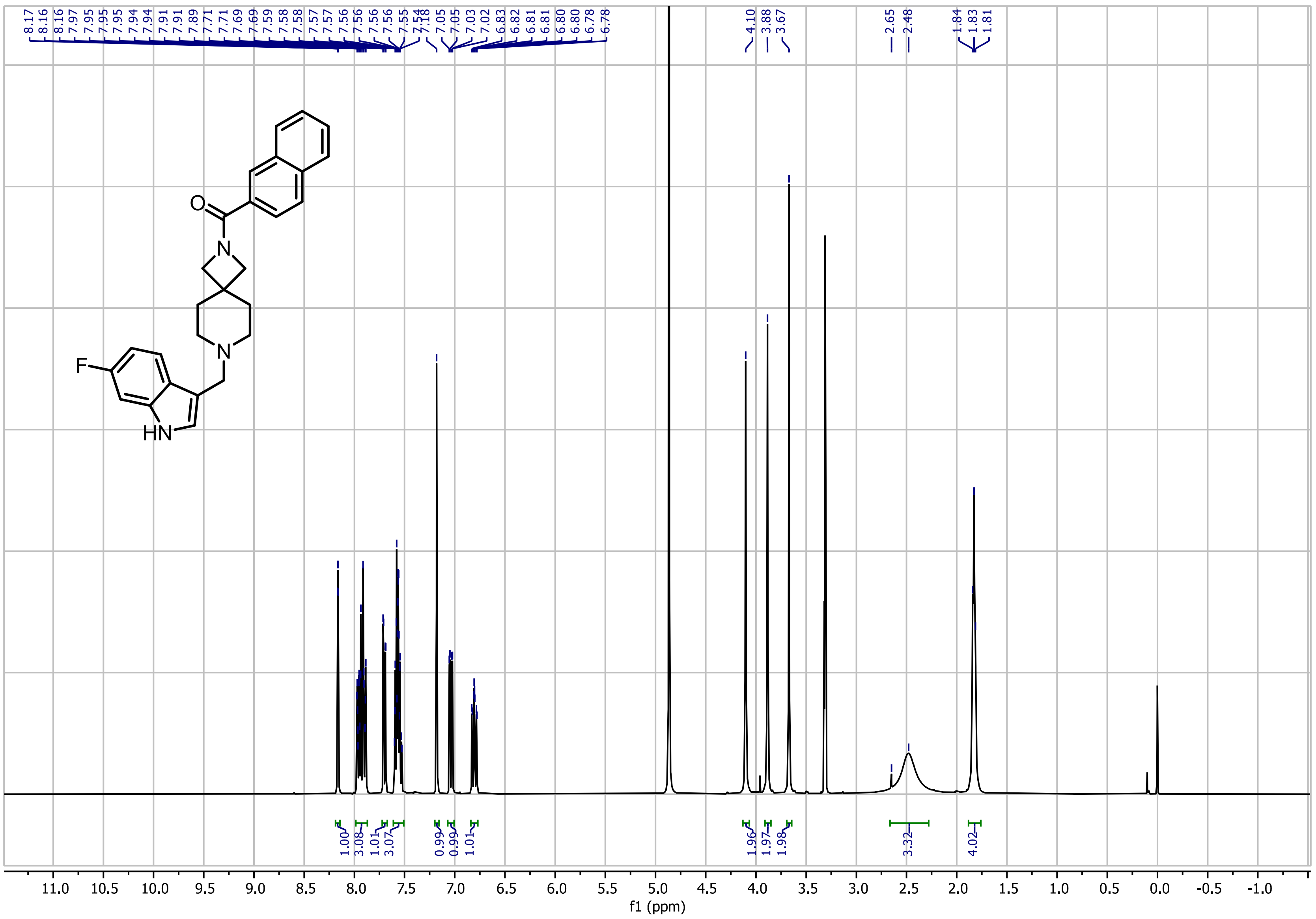

Figure S77: ¹H NMR spectrum of **27**in MeOD-d_4.

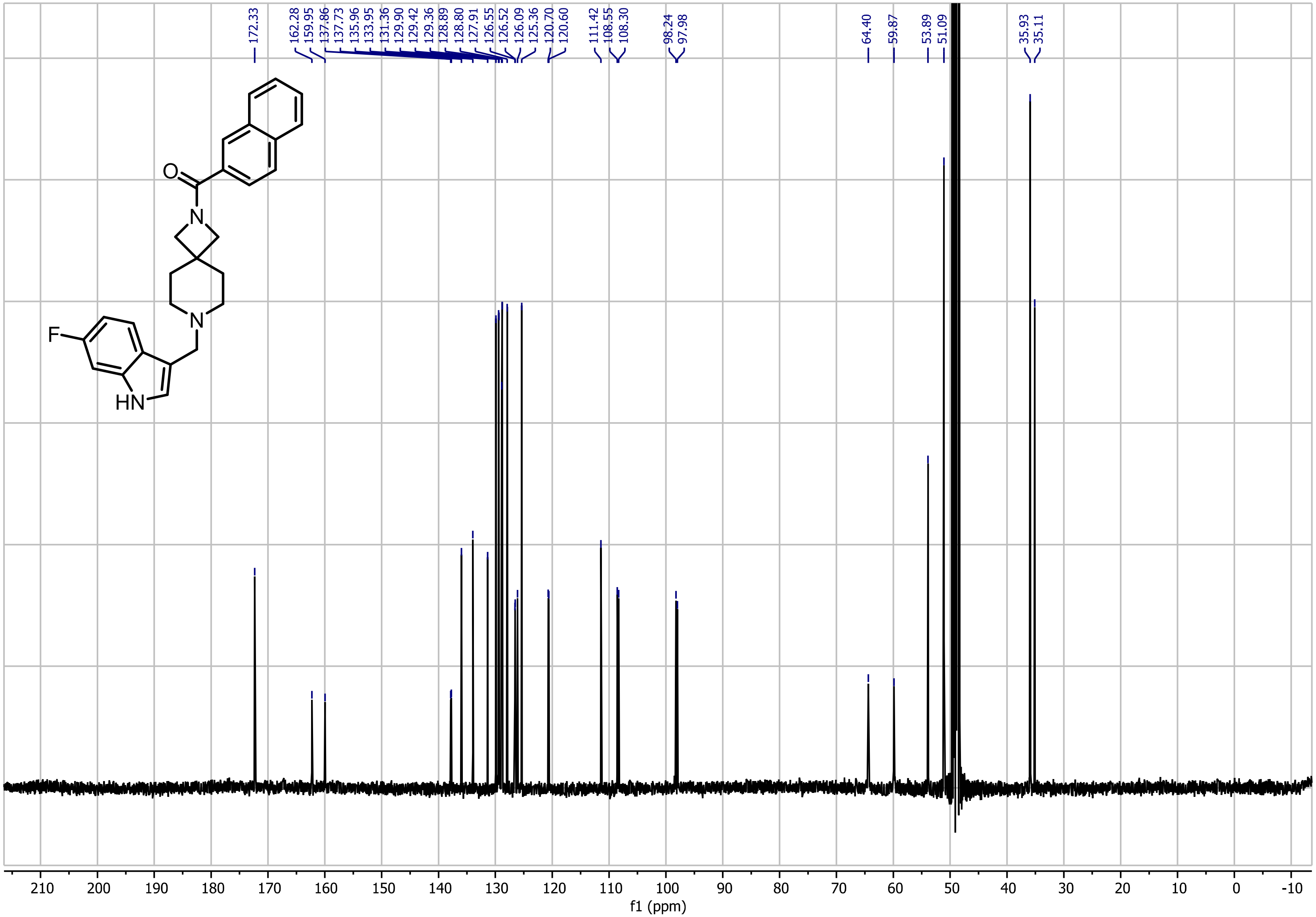

Figure S78: ^{13}C NMR spectrum of **27** in MeOD-d_4.

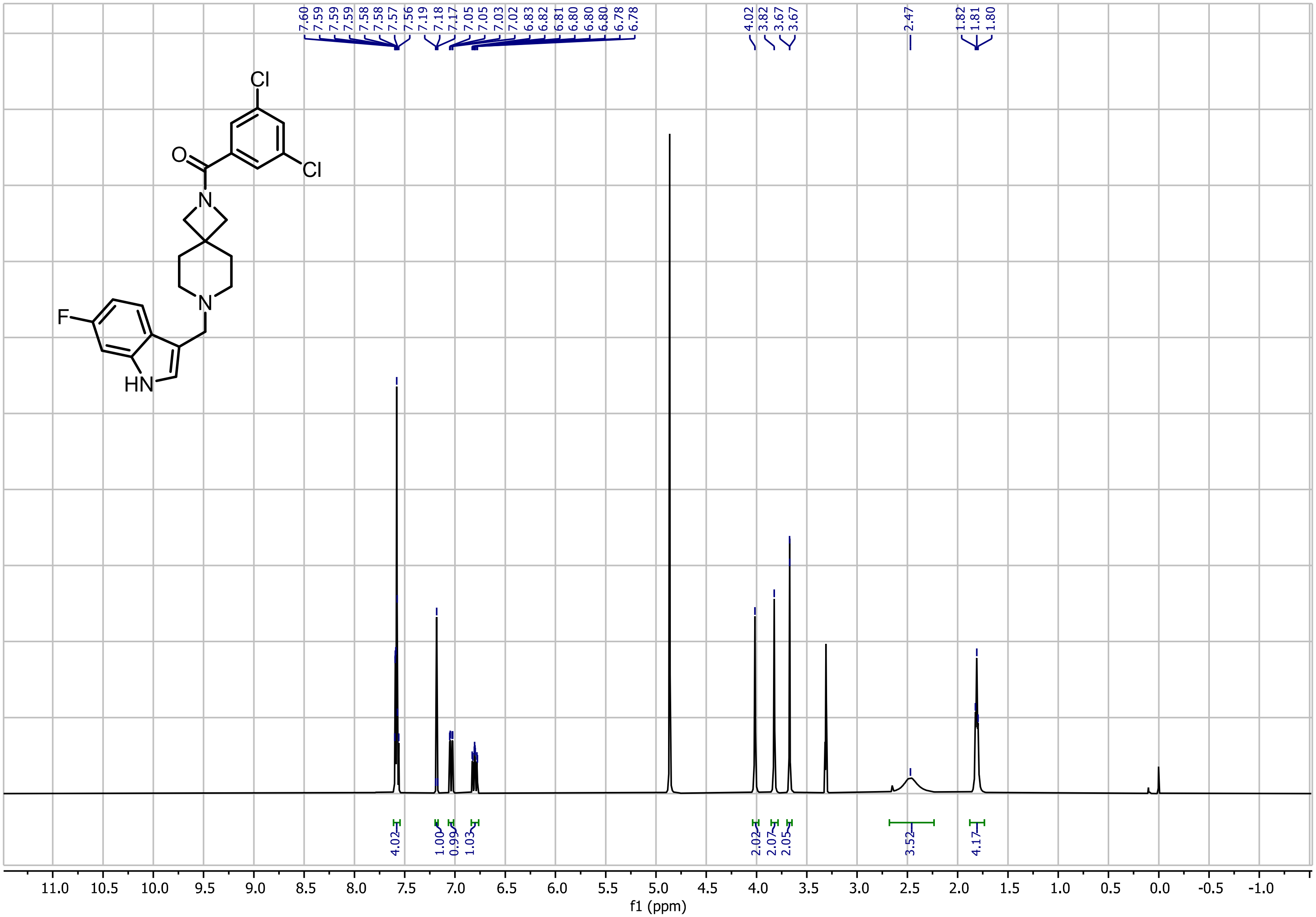

Figure S79: ^{1}H NMR spectrum of **28** in MeOD-d_4.

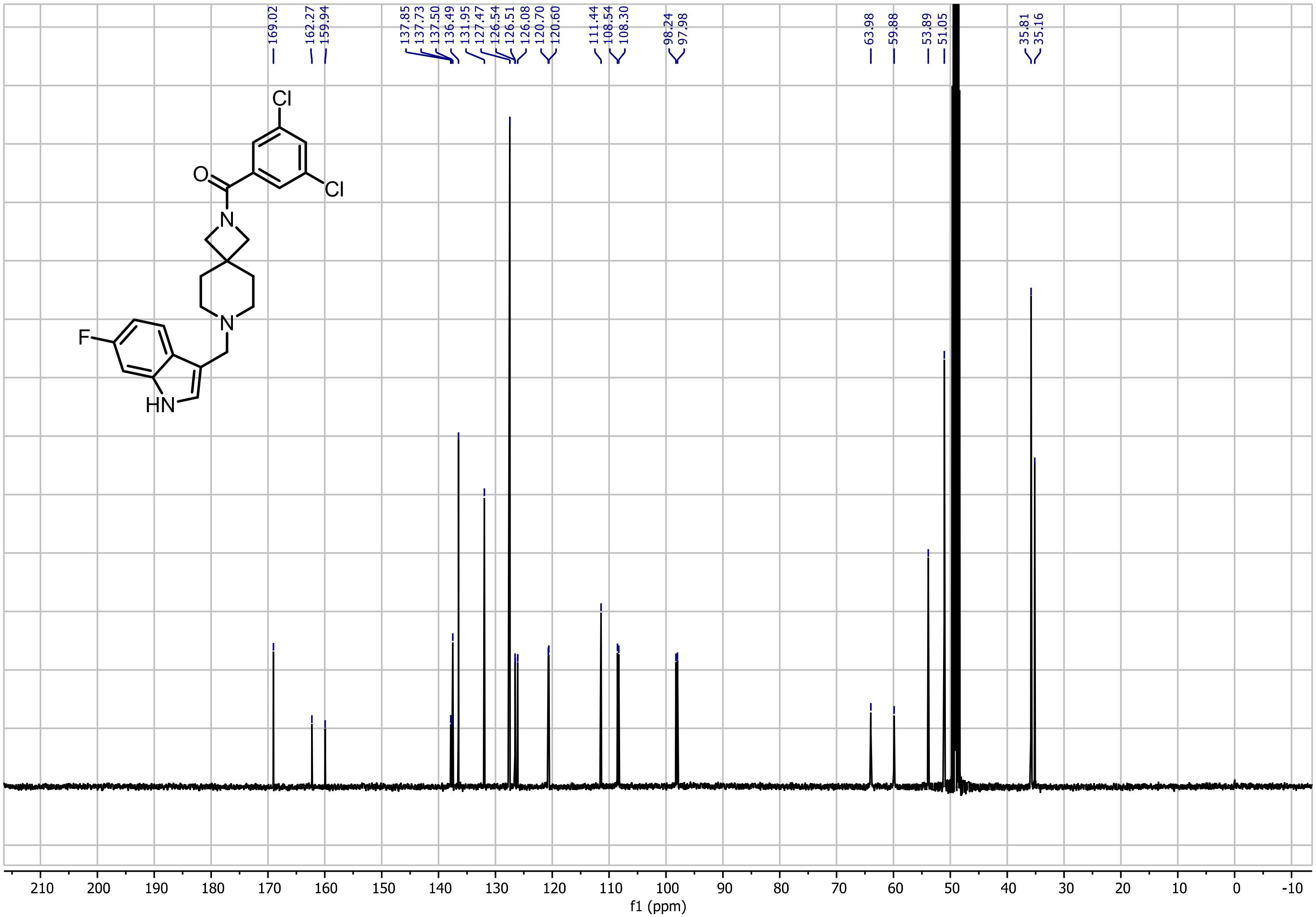

Figure S80: ^{13}C NMR spectrum of **28** in MeOD-d_4.

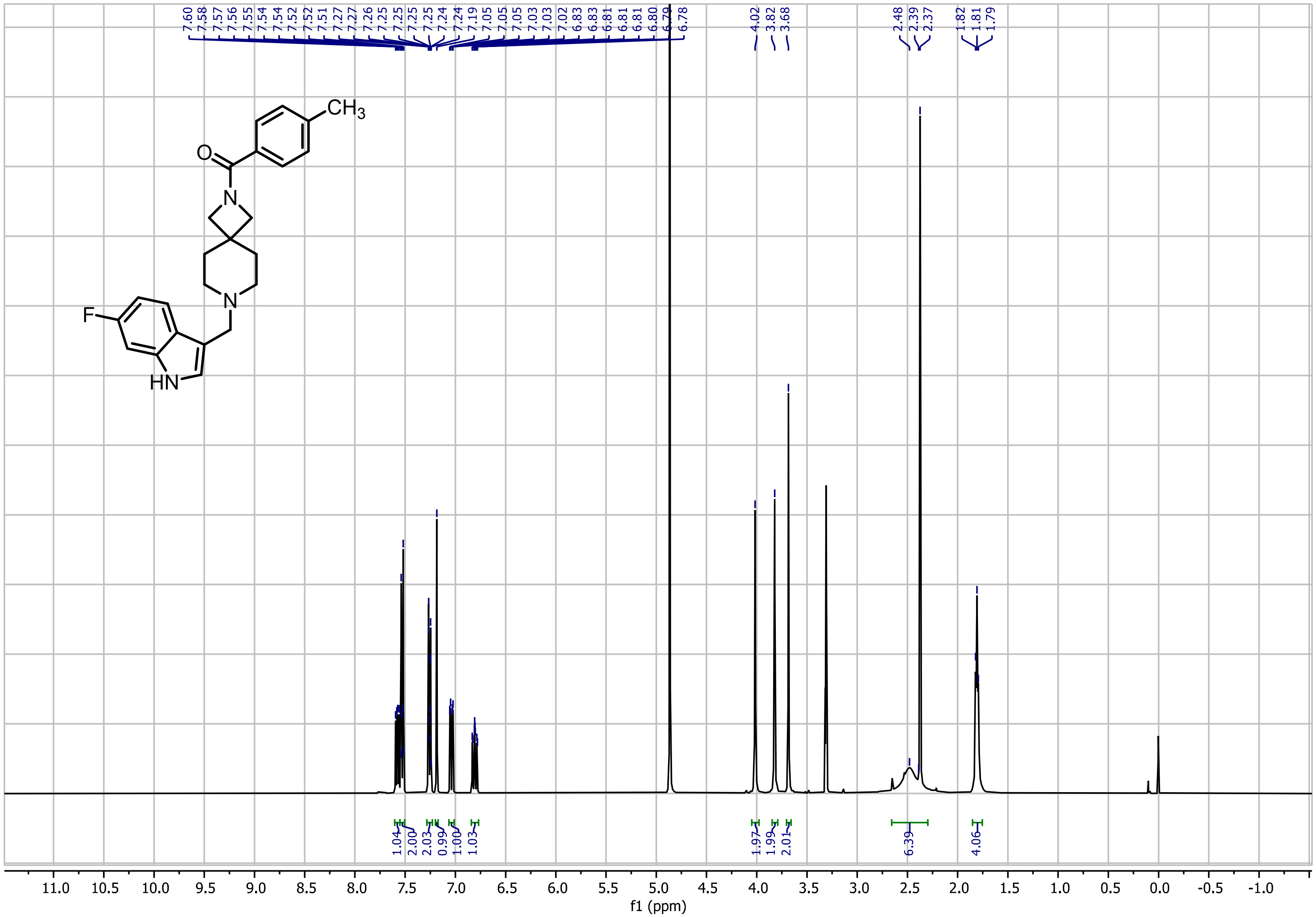

Figure S81: ^{1}H NMR spectrum of **29** in MeOD-d_4.

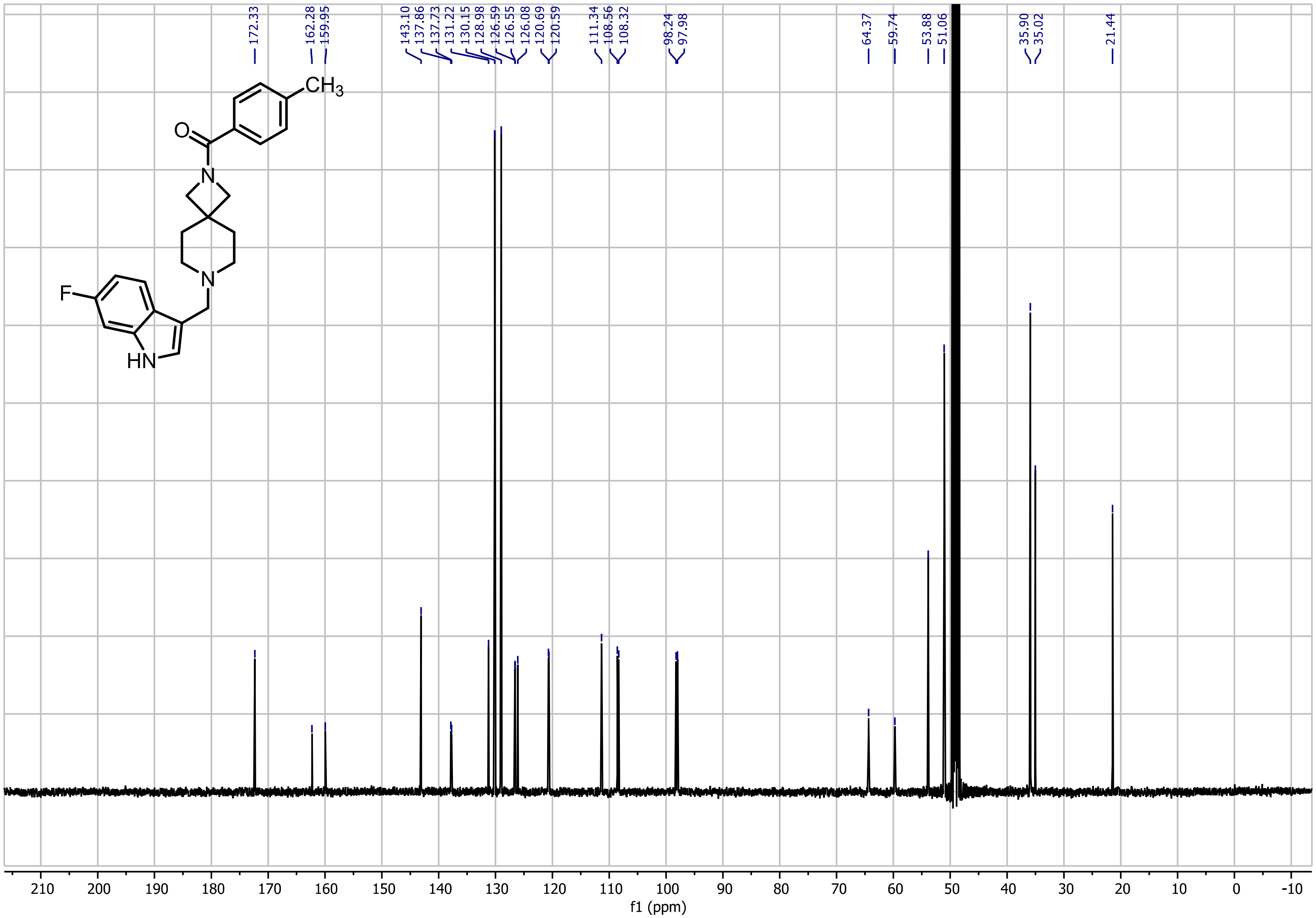

Figure S82: ^{13}C NMR spectrum of **29** in MeOD-d_4.

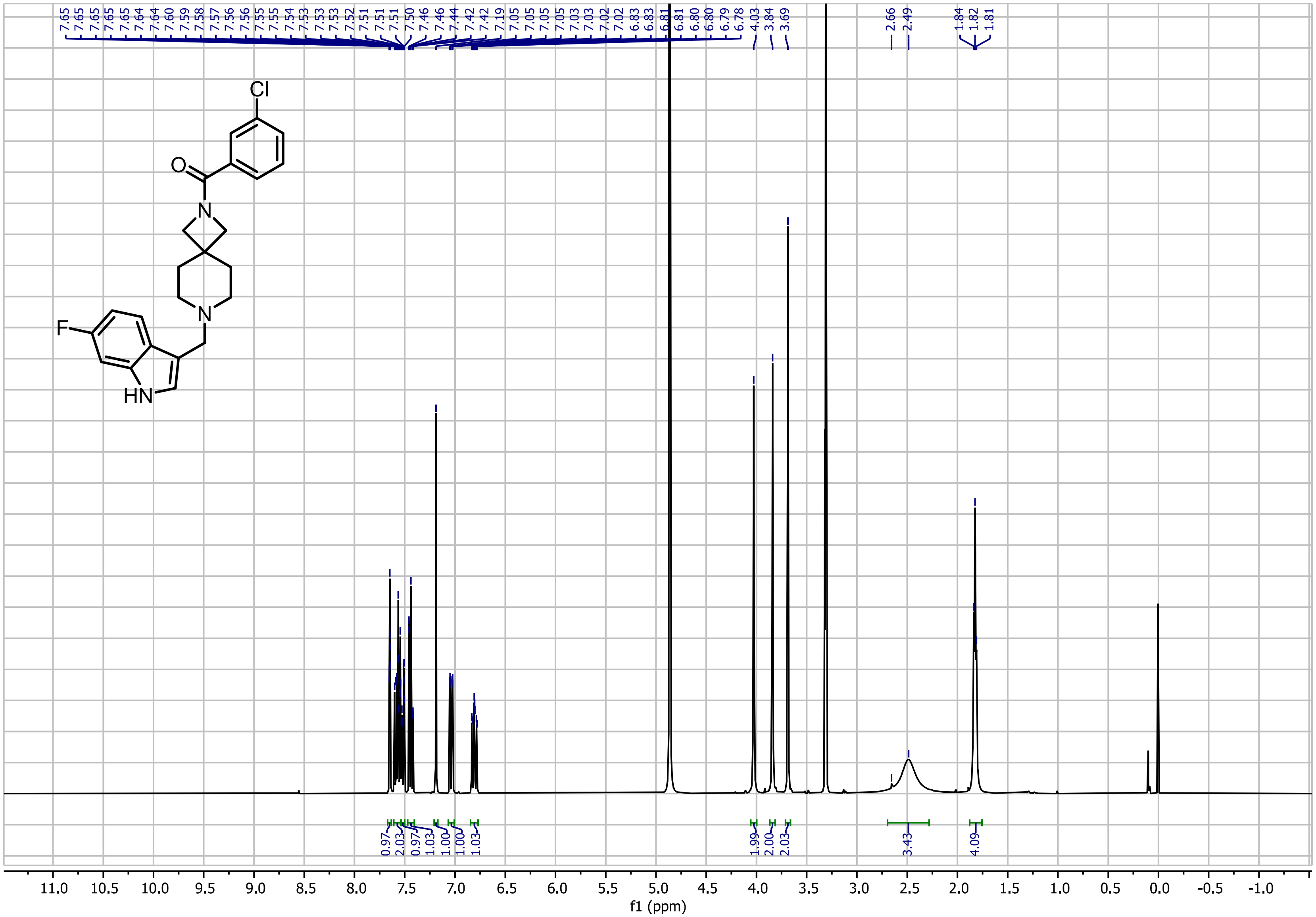

Figure S83: ^{1}H NMR spectrum of **30** in MeOD-d_4.

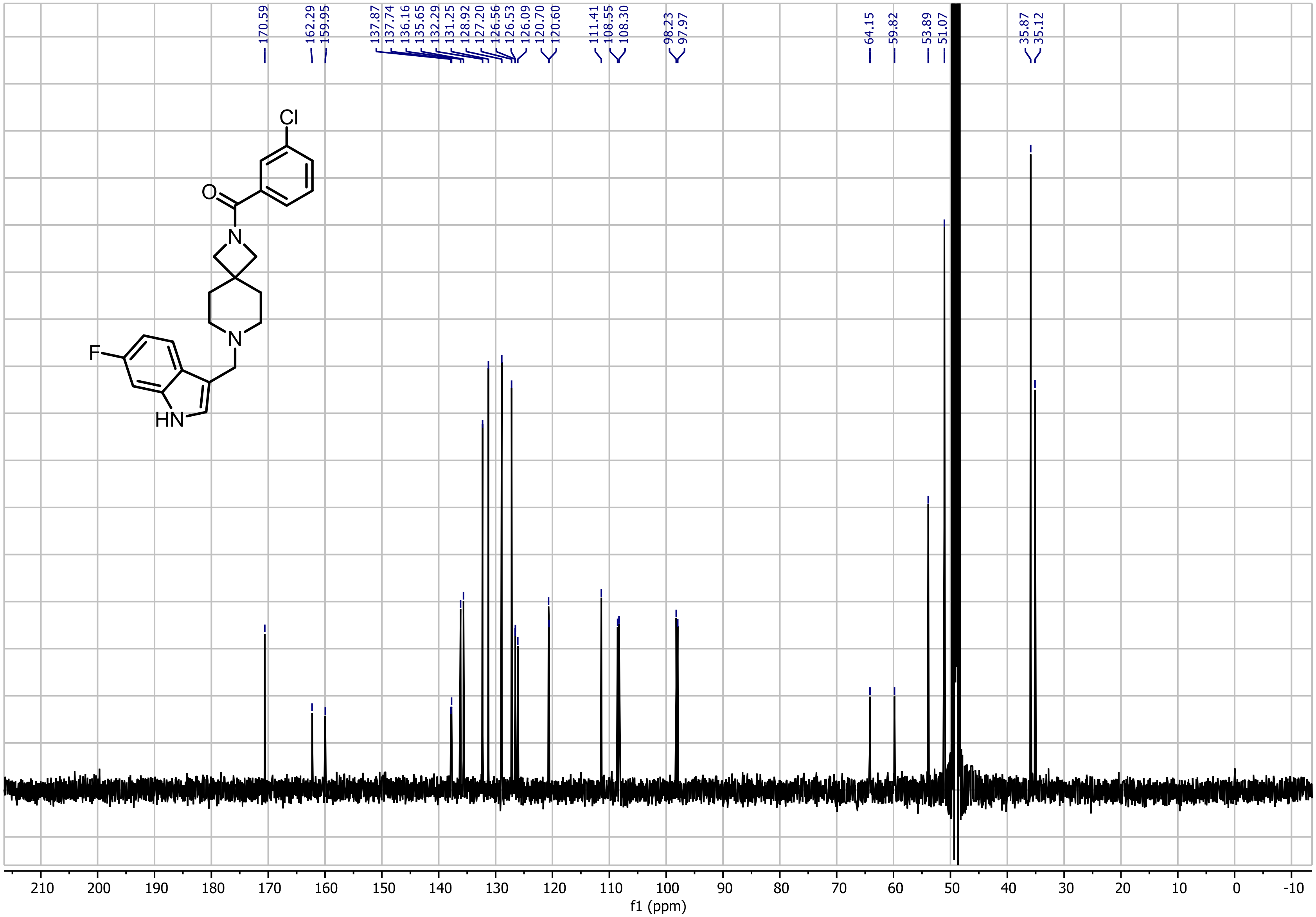

Figure S84: ^{13}C NMR spectrum of **30** in MeOD-d_4.

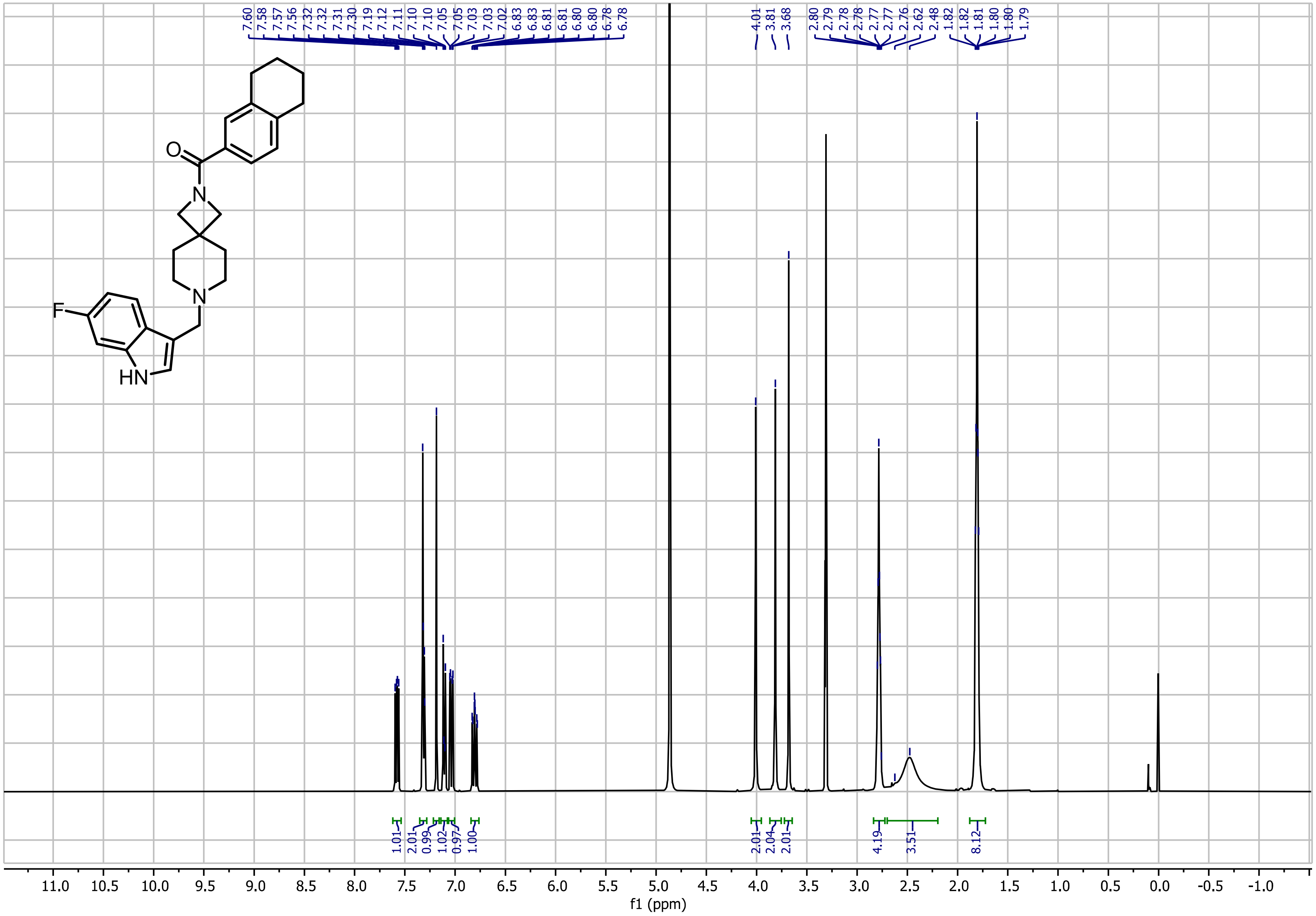

Figure S85: ^{1}H NMR spectrum of **31** in MeOD-d_4.

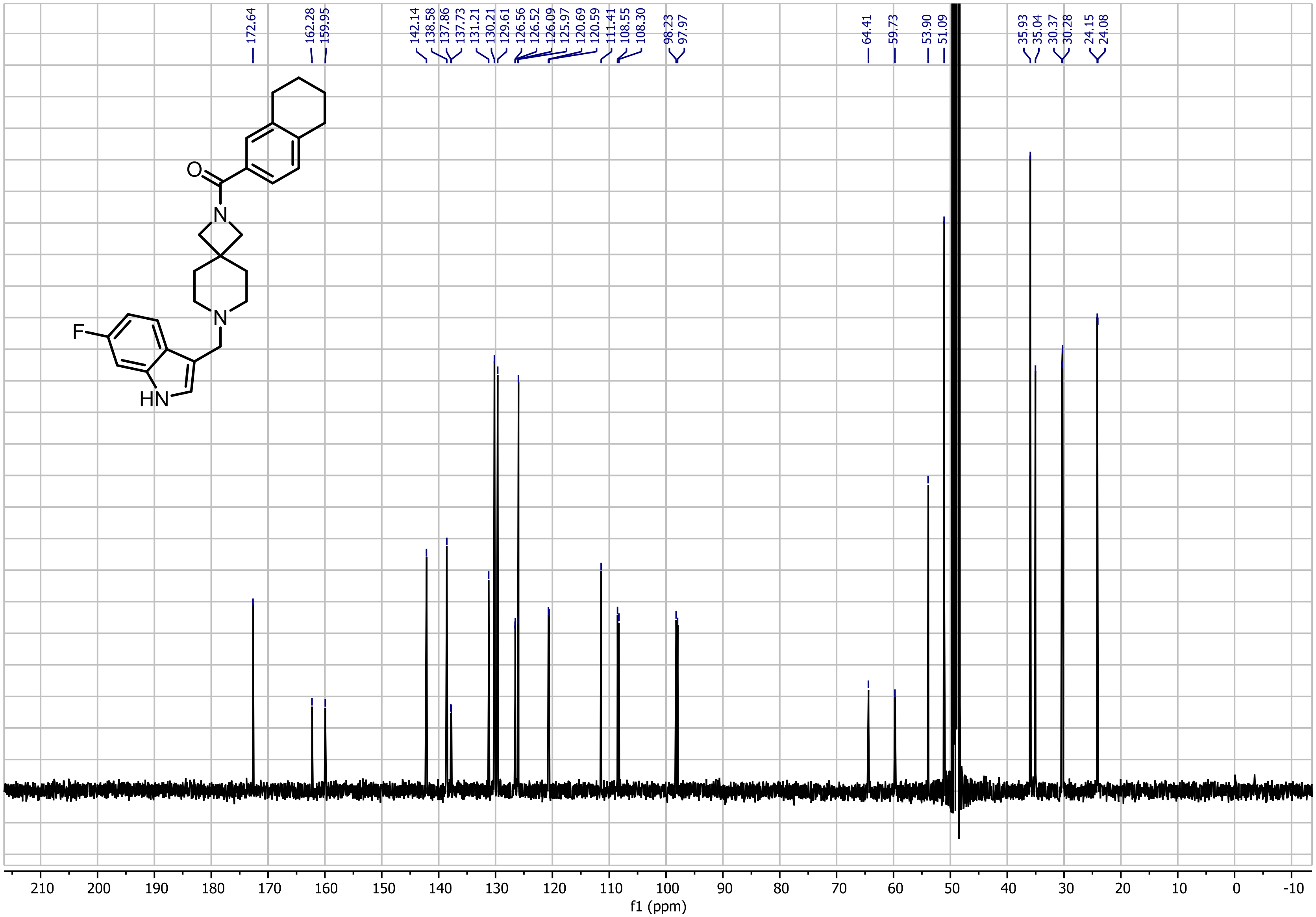

Figure S86: ^{13}C NMR spectrum of **31** in MeOD-d_4.

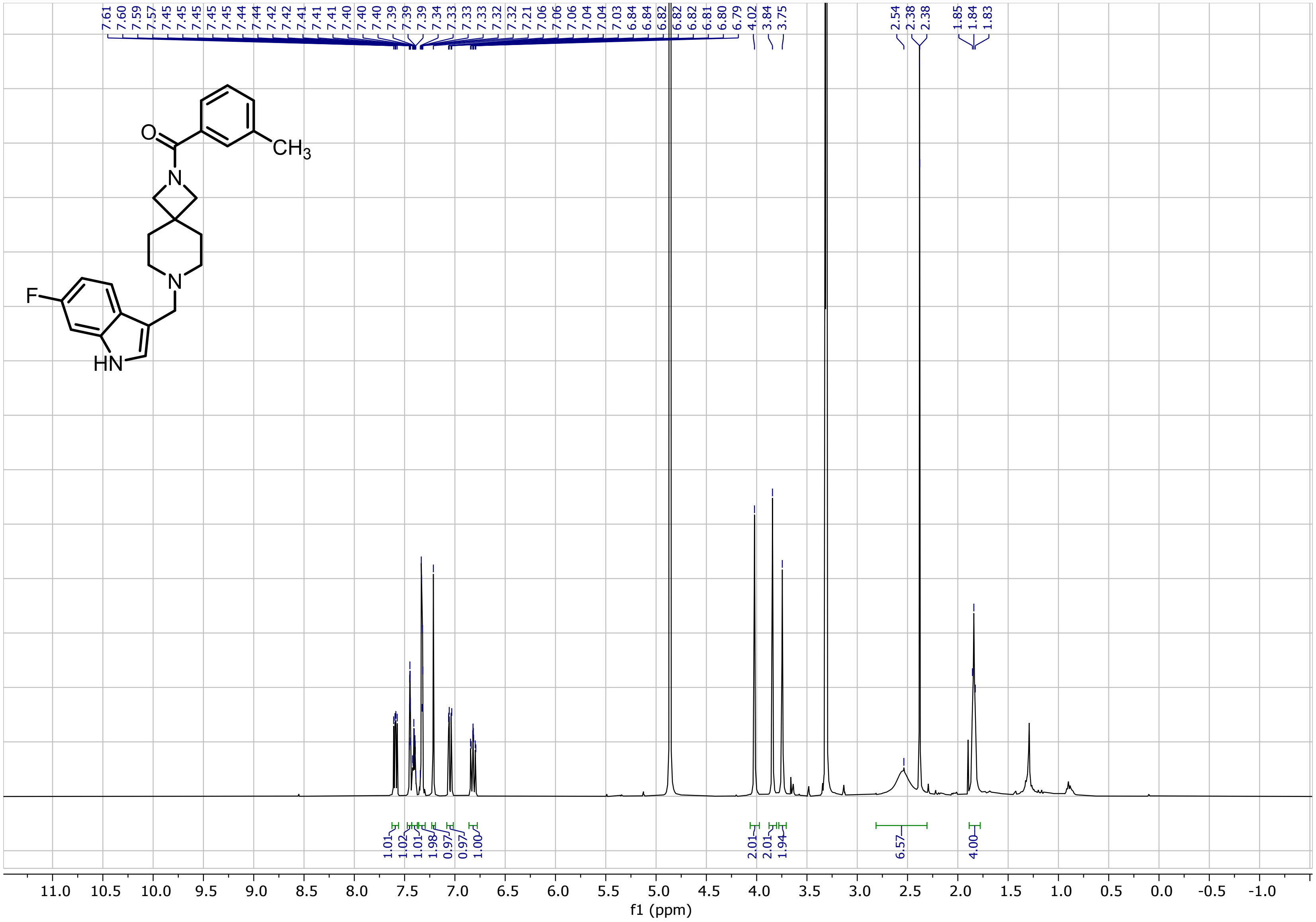

Figure S87: ^{1}H NMR spectrum of **32** in MeOD-d_4.

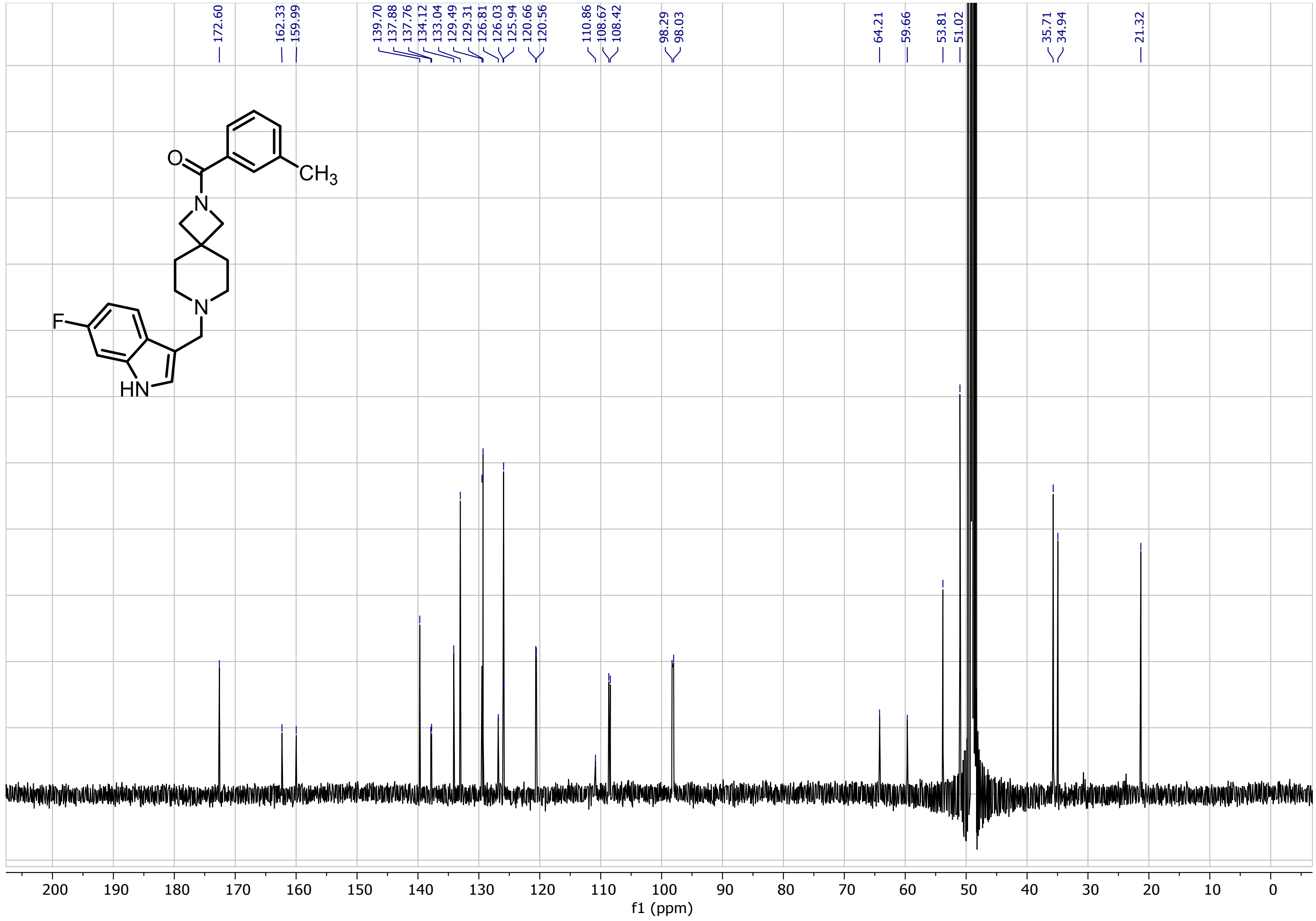

Figure S88: ^{13}C NMR spectrum of **32** in MeOD-d_4.

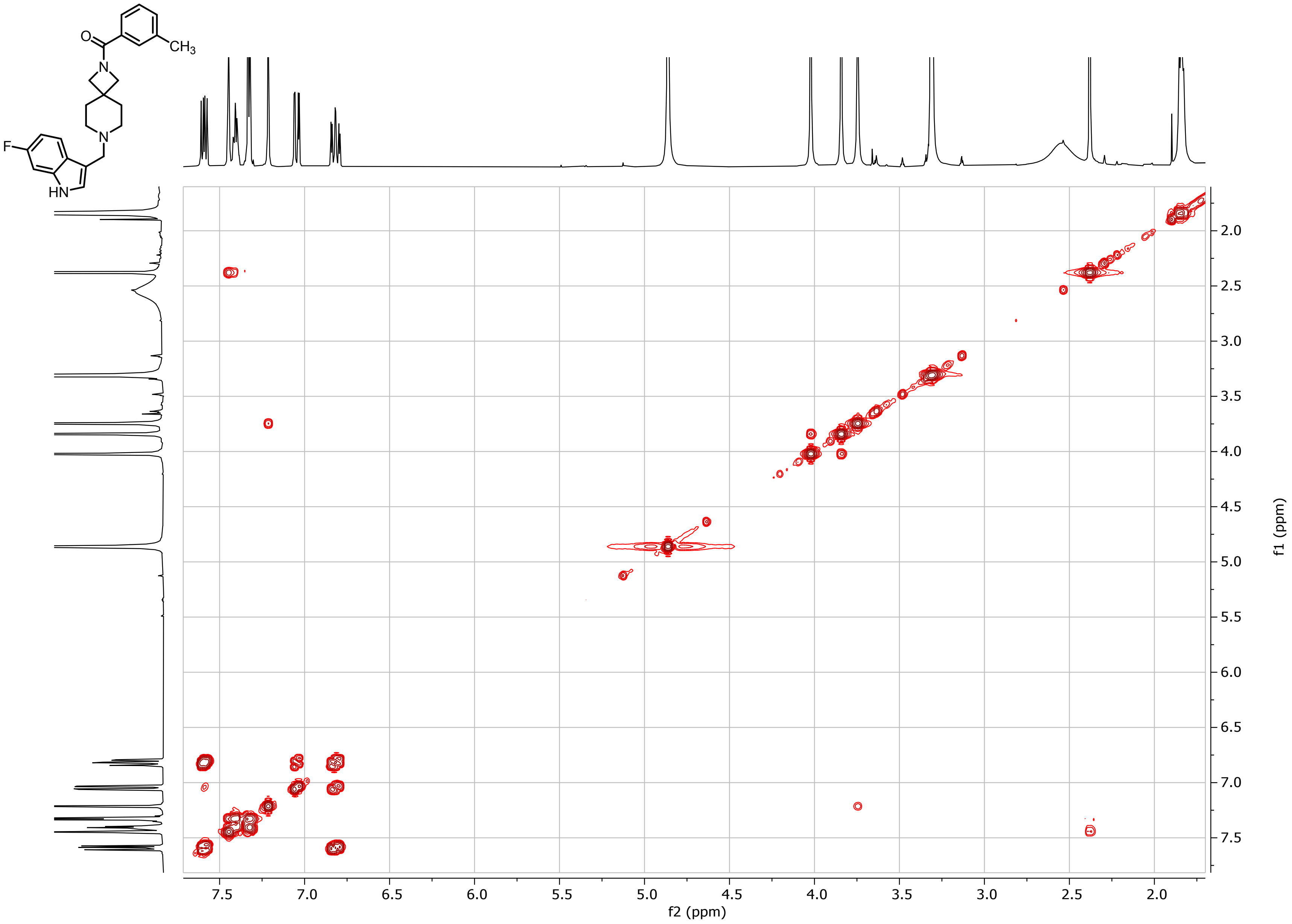

Figure S89: COSY spectrum of **32** in MeOD-d_4.

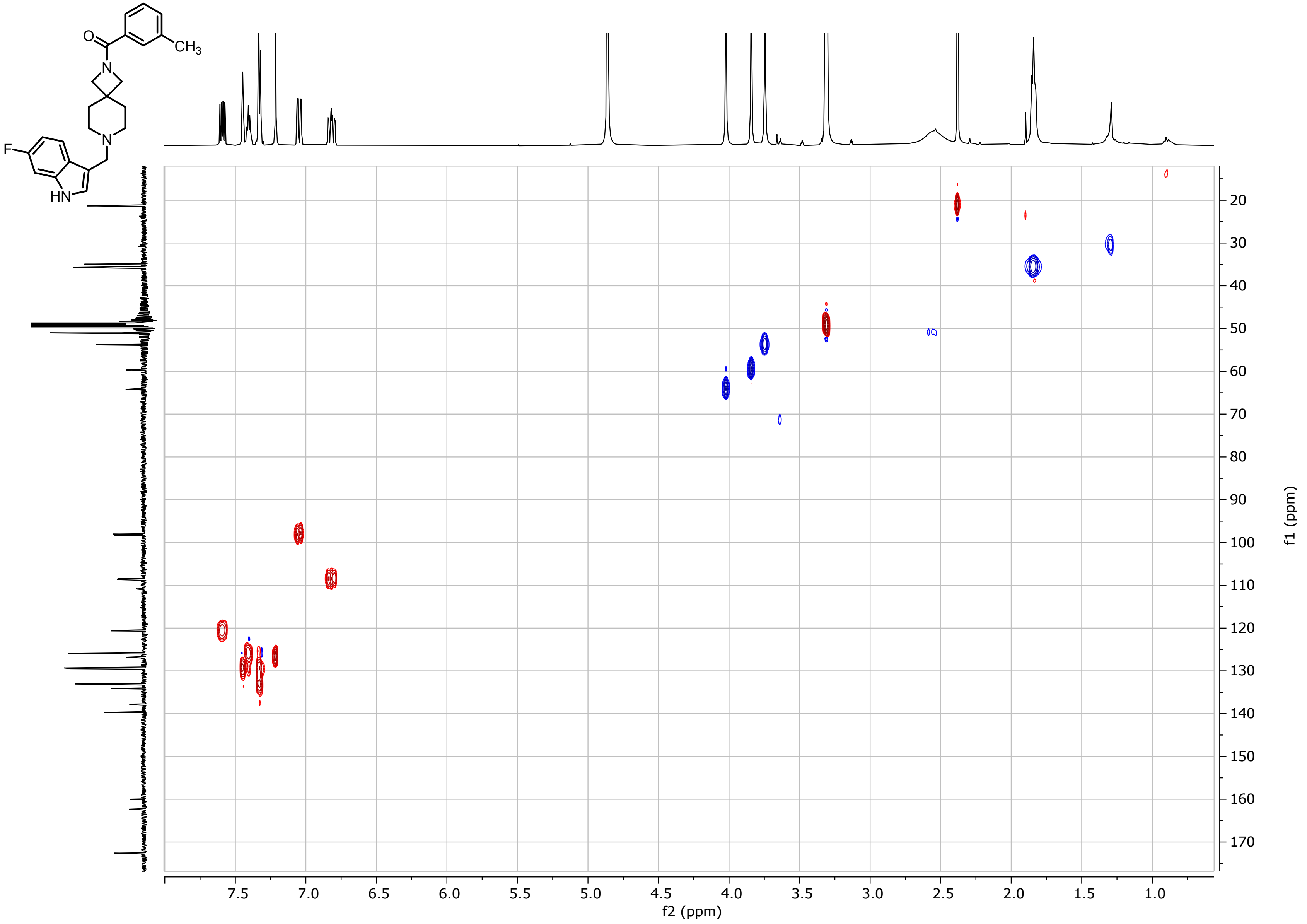

Figure S90: HSQC spectrum of **32** in MeOD-d_4.

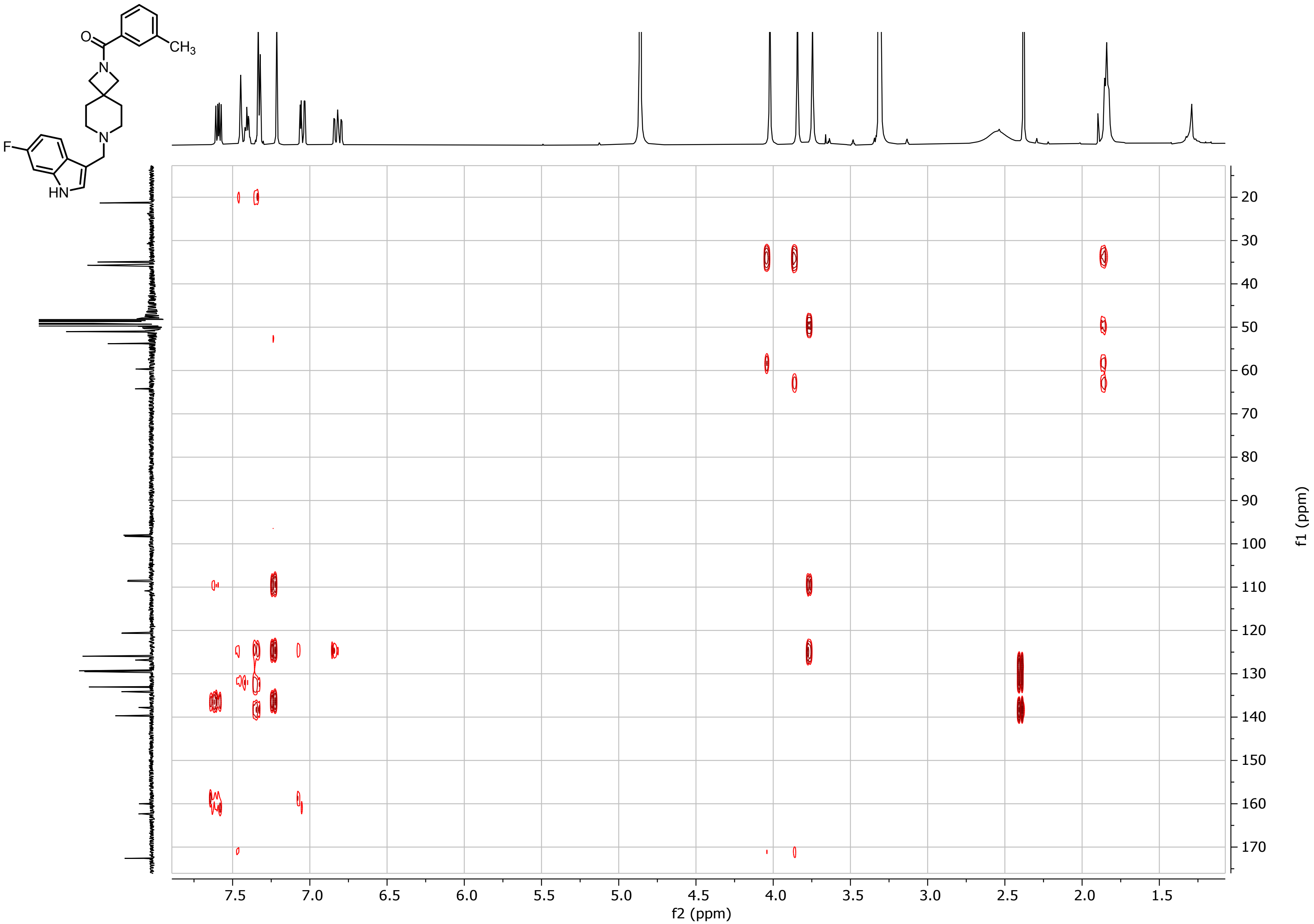

Figure S91: HMBC spectrum of **32** in MeOD-d_4.

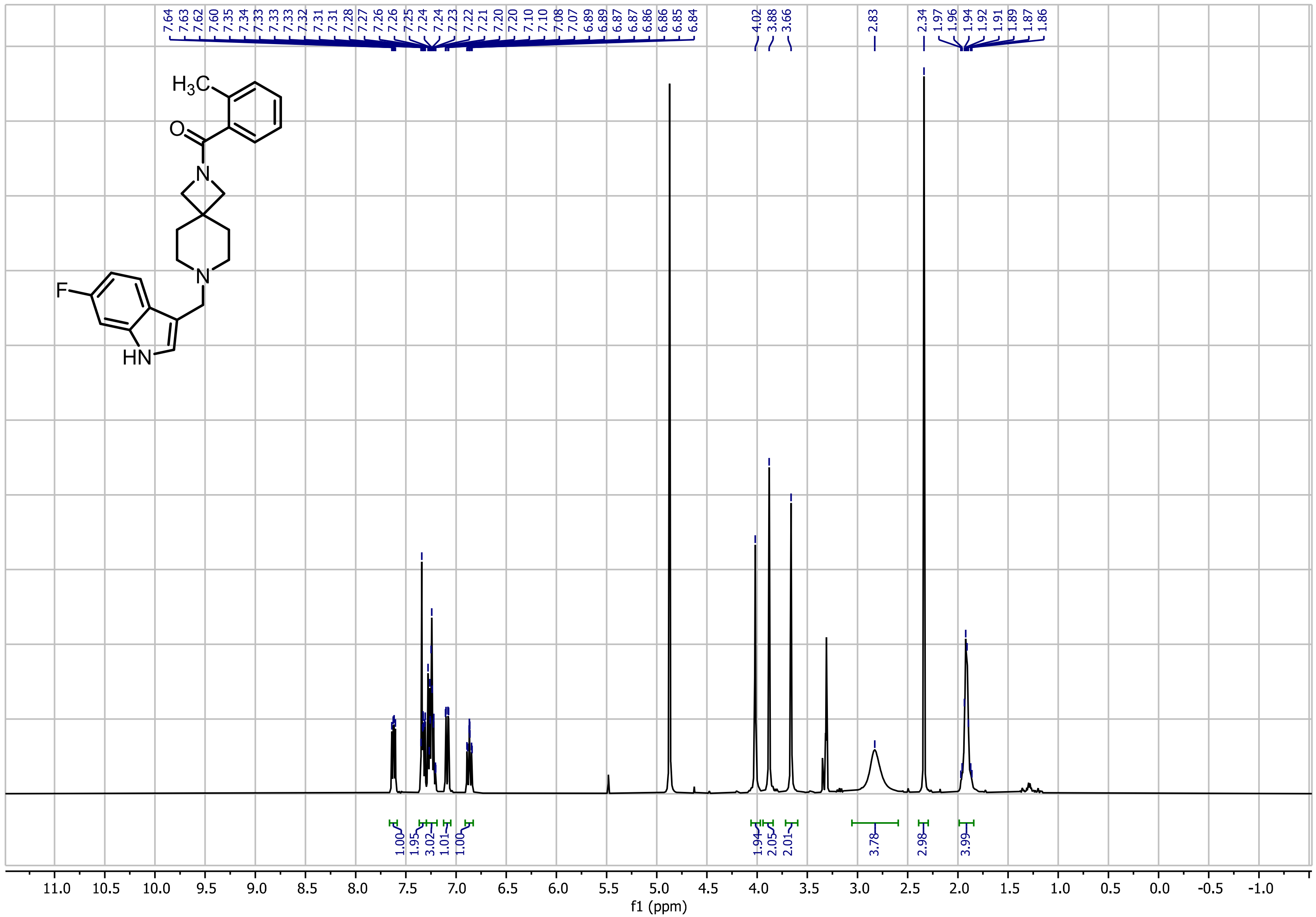

Figure S92: ^{1}H NMR spectrum of **33** in MeOD-d_4.

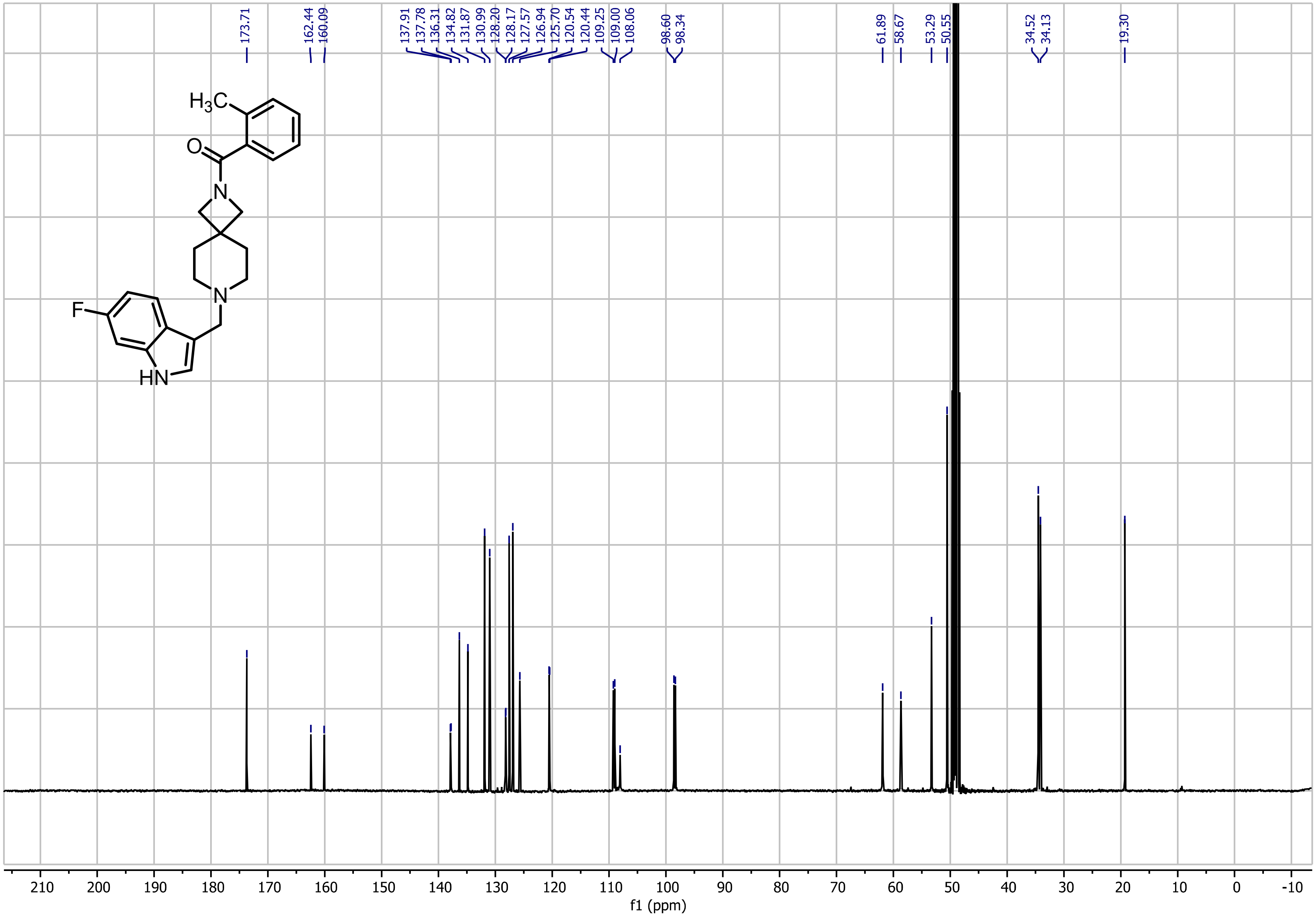

Figure S93: ^{13}C NMR spectrum of **33** in MeOD-d_4.

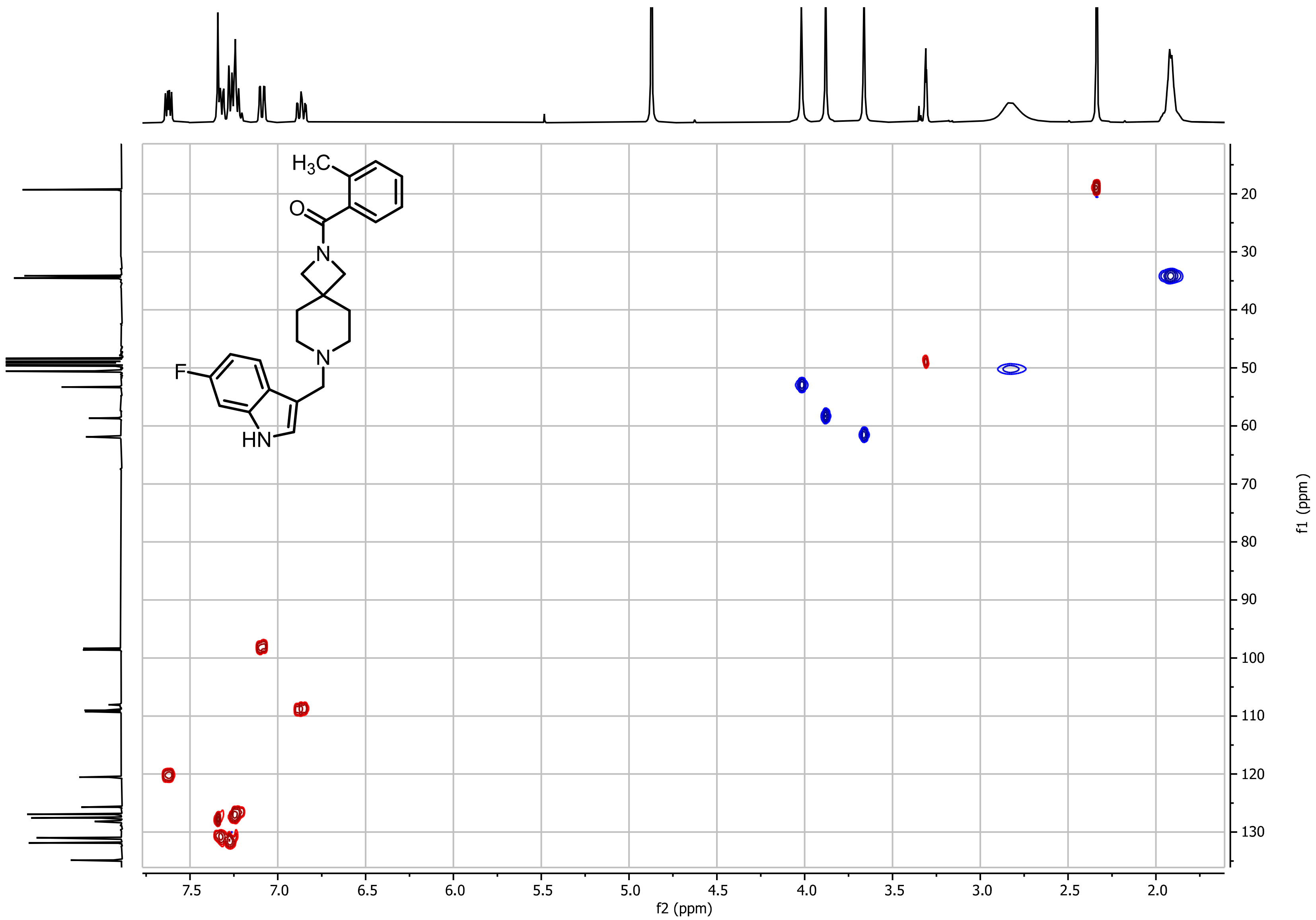

Figure S94: HSQC spectrum of **33** in MeOD-d_4.

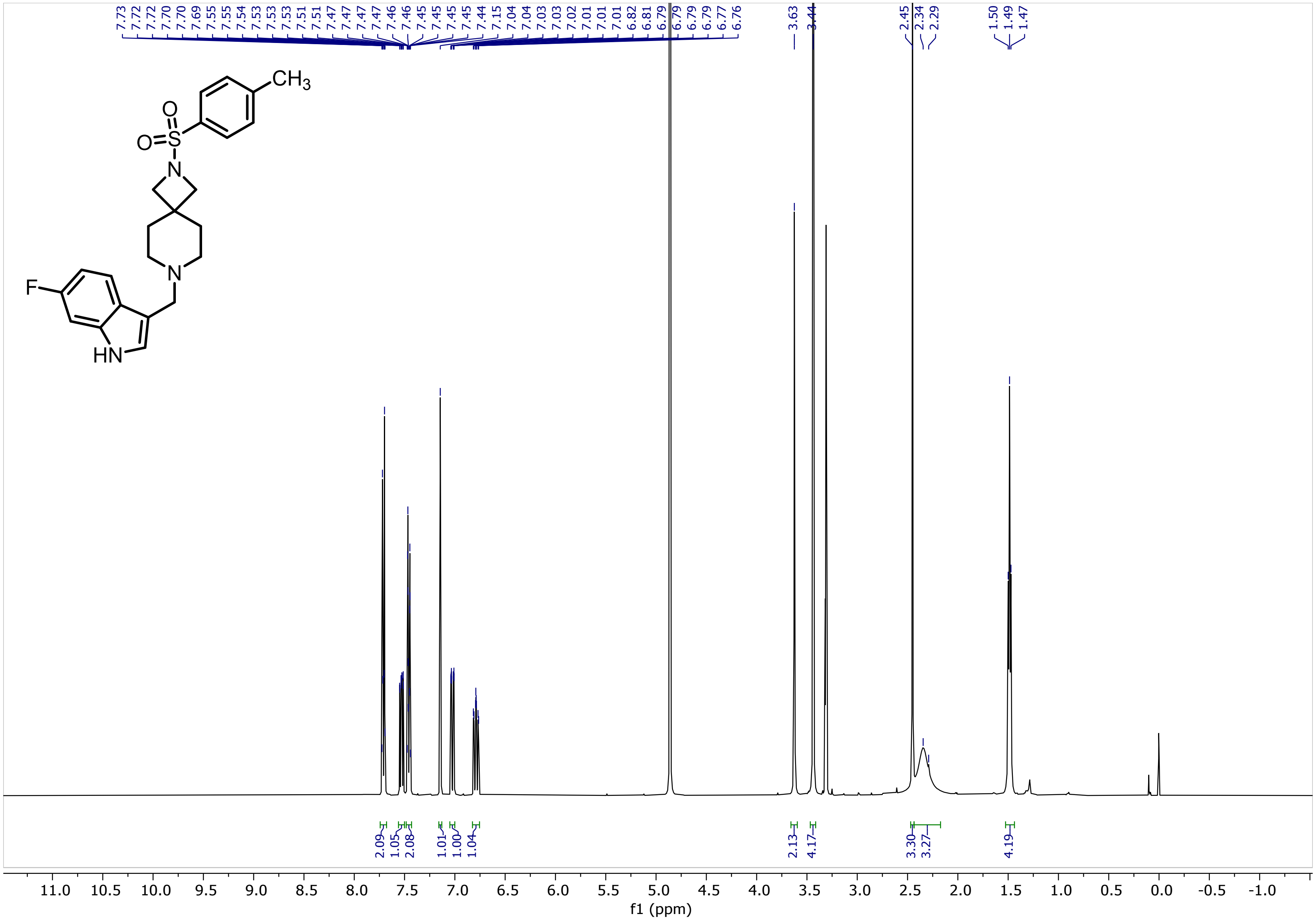

Figure S95: ^{1}H NMR spectrum of **34** in MeOD-d_4.

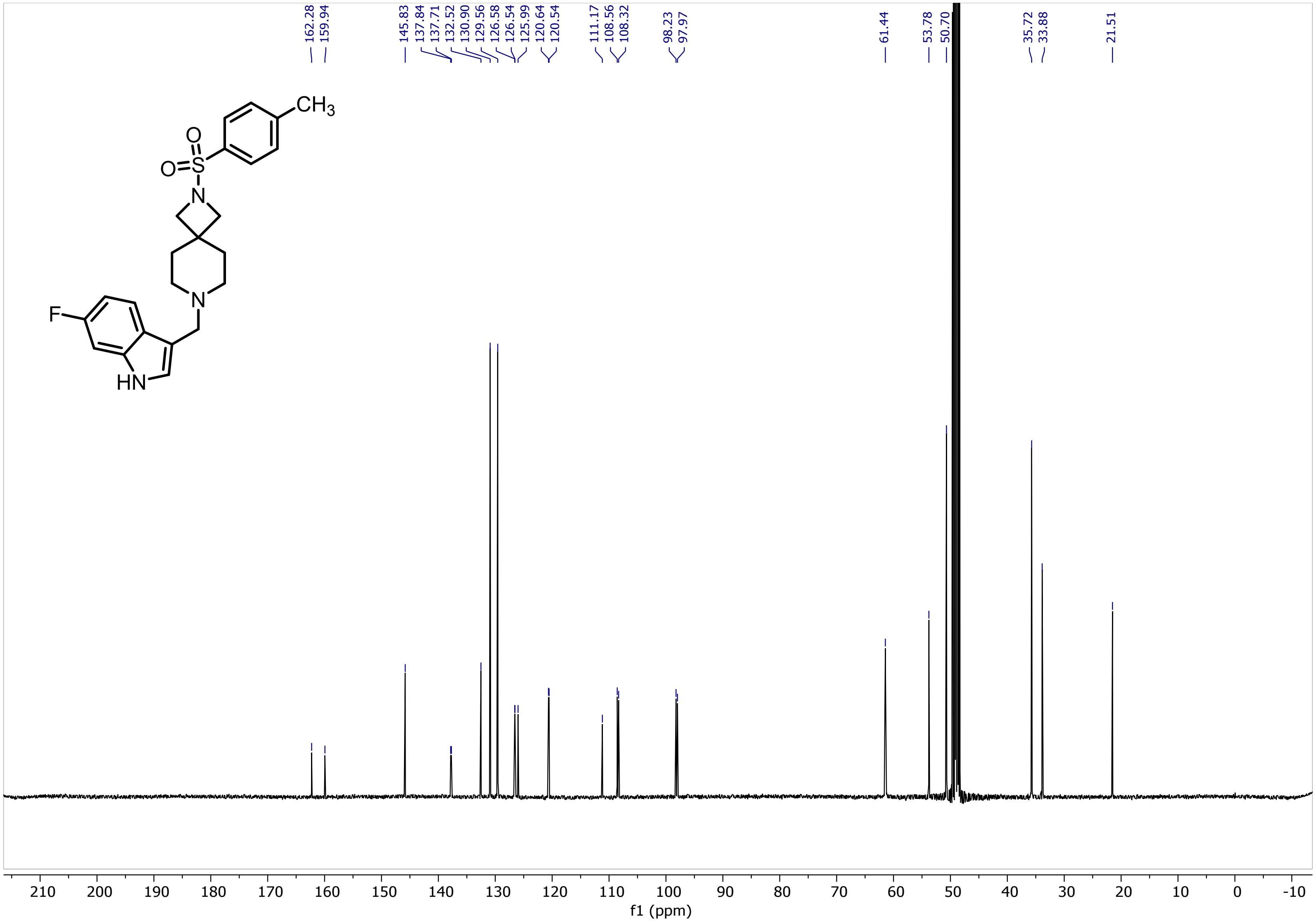

Figure S96: ^{13}C NMR spectrum of **34** in MeOD-d_4.

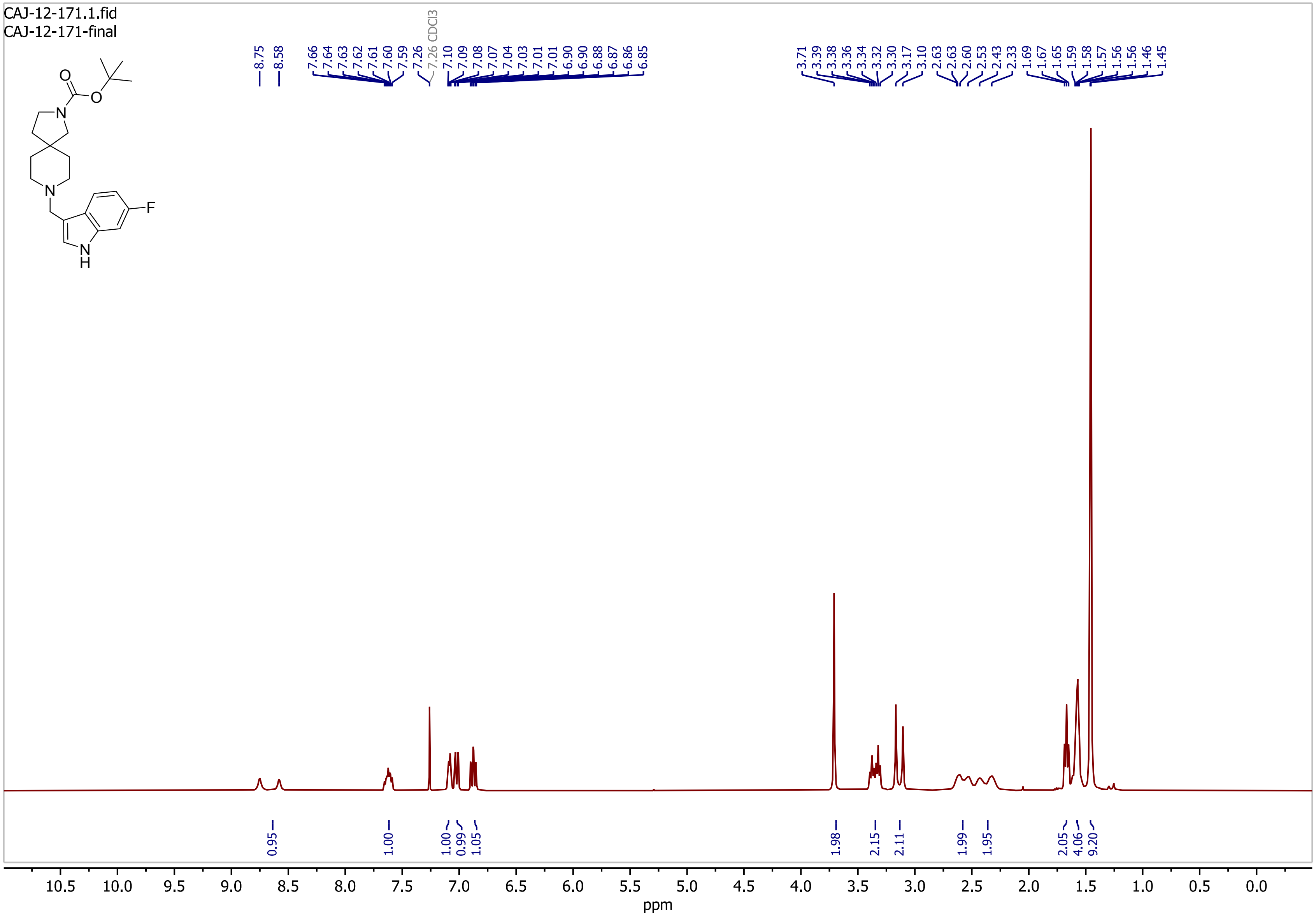

Figure S97: ^{1}H NMR spectrum of **40** in $CDCl_3$.

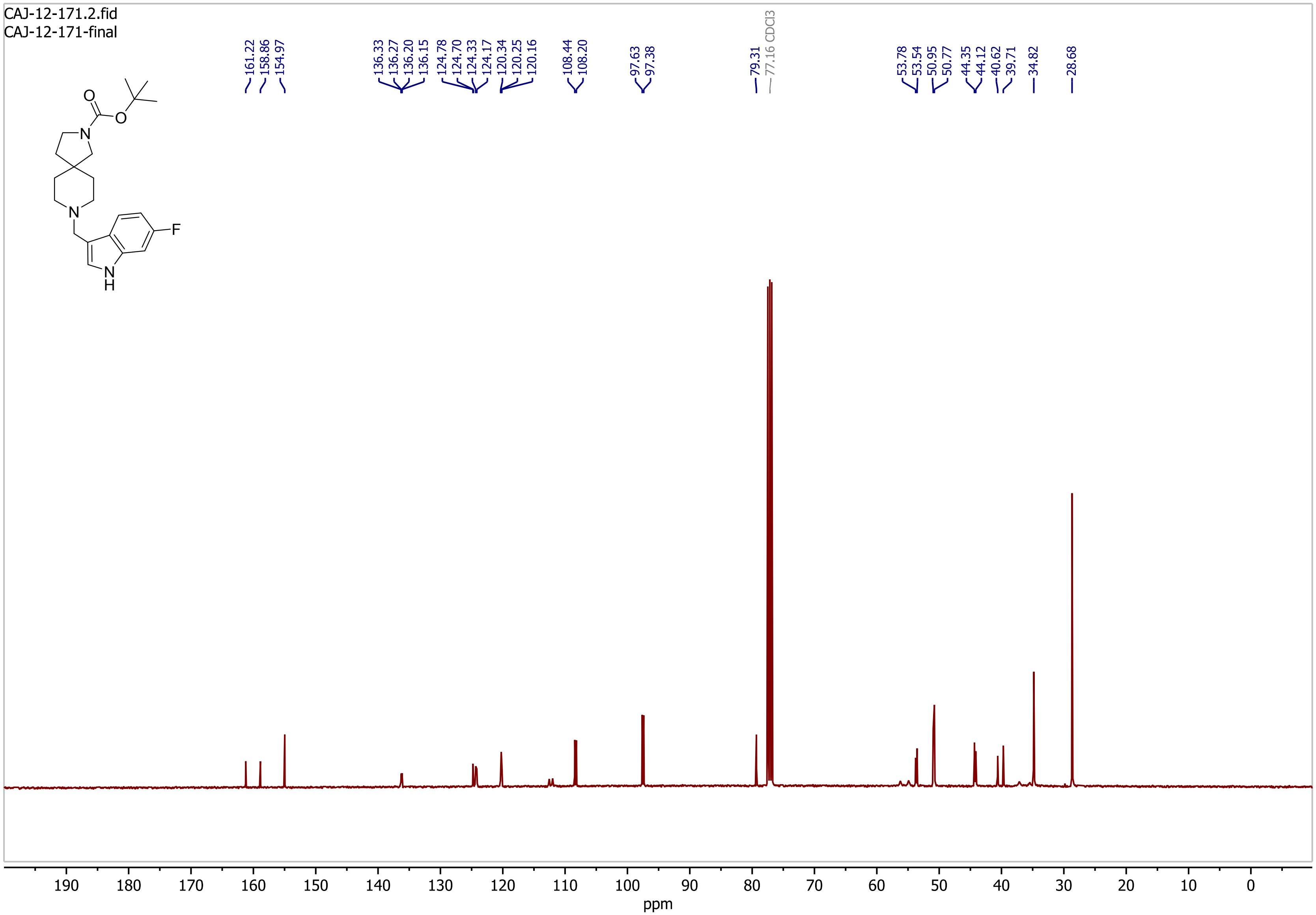

Figure S98: ^{13}C NMR spectrum of **40** in $CDCl_3$.

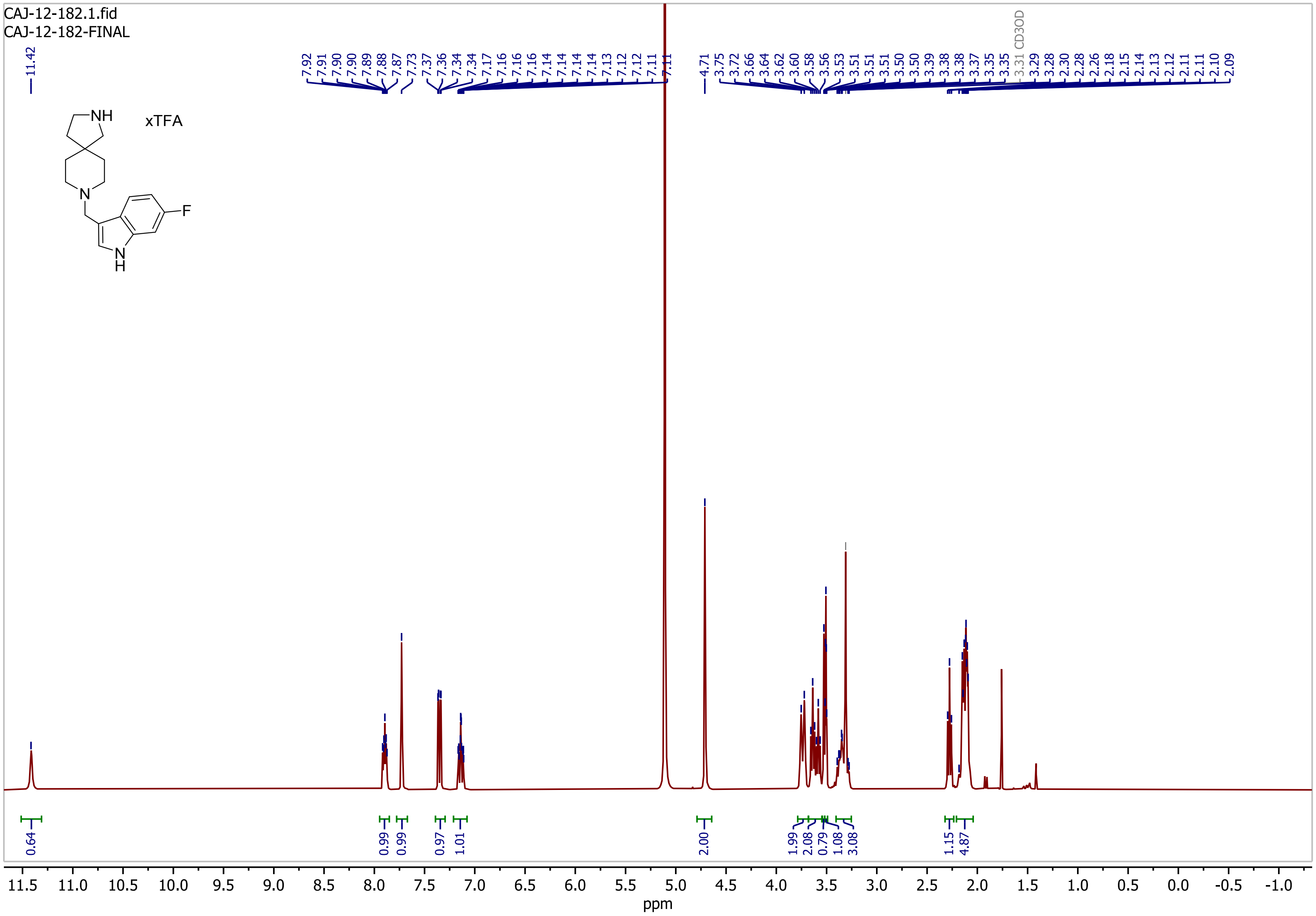

Figure S99: ^{1}H NMR spectrum of **41** in MeOD-d_4.

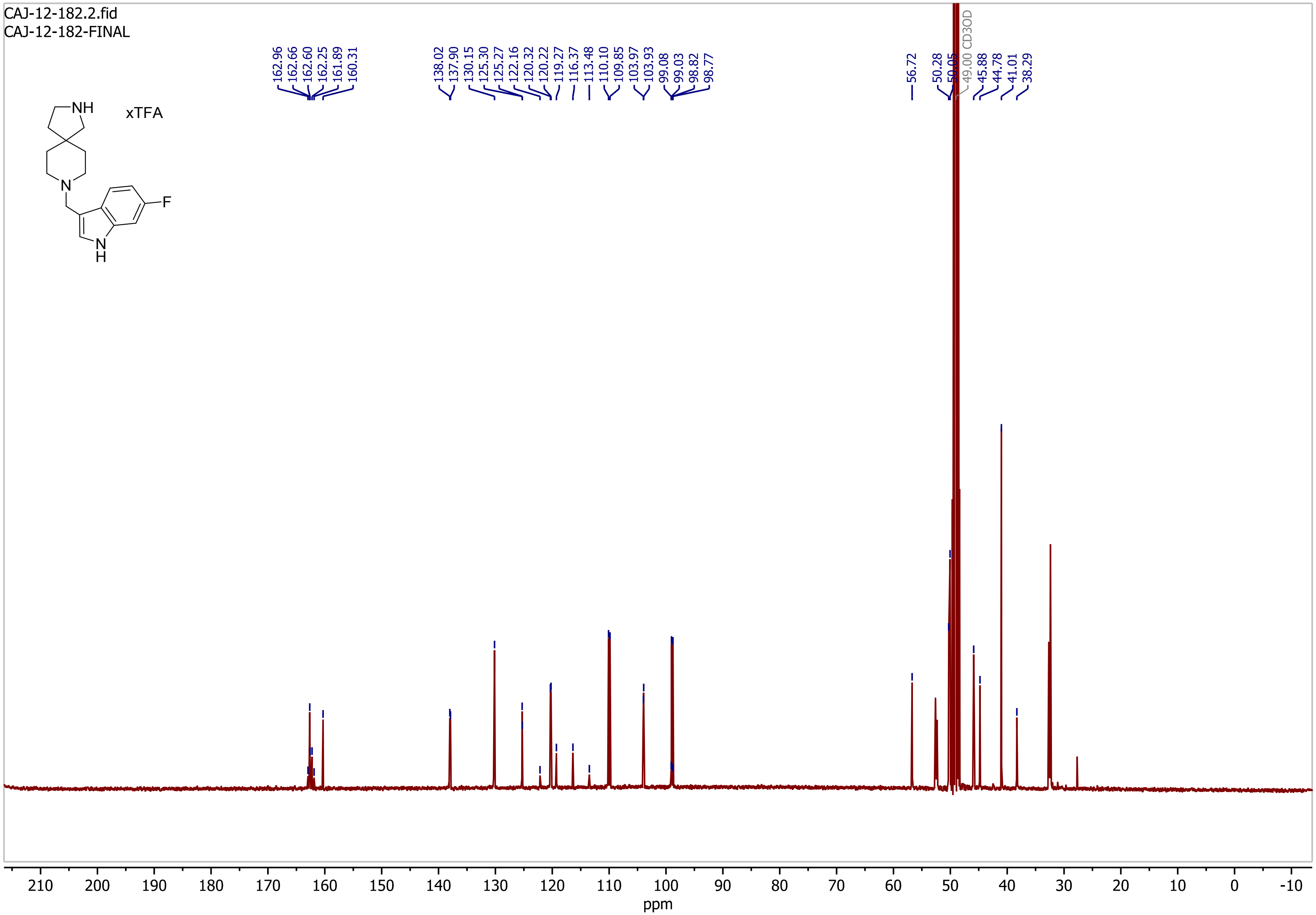

Figure S100: ^{13}C NMR spectrum of **41** in MeOD-d_4.

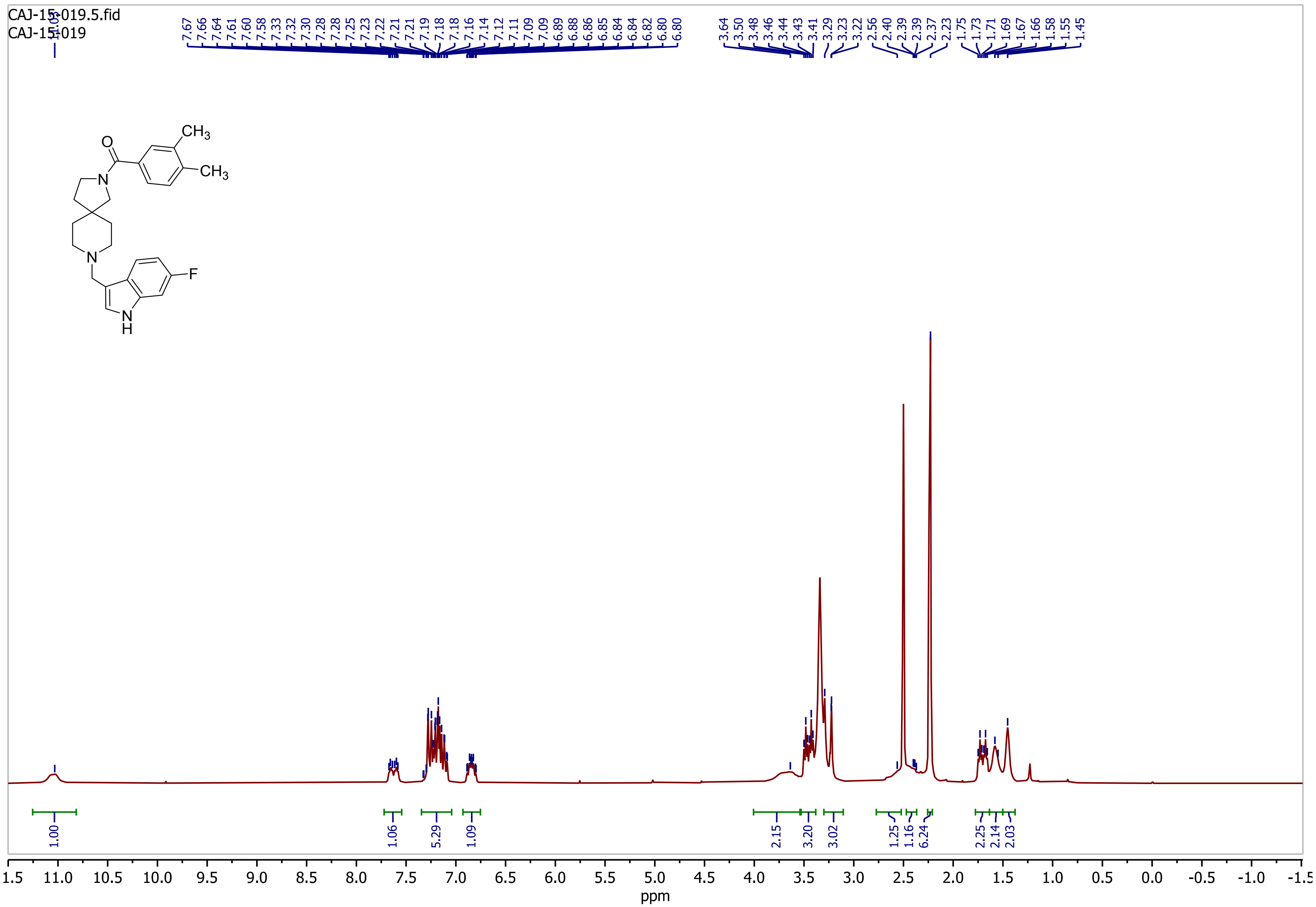

Figure S101: ^{1}H NMR spectrum of **41** in DMSO-d_6.

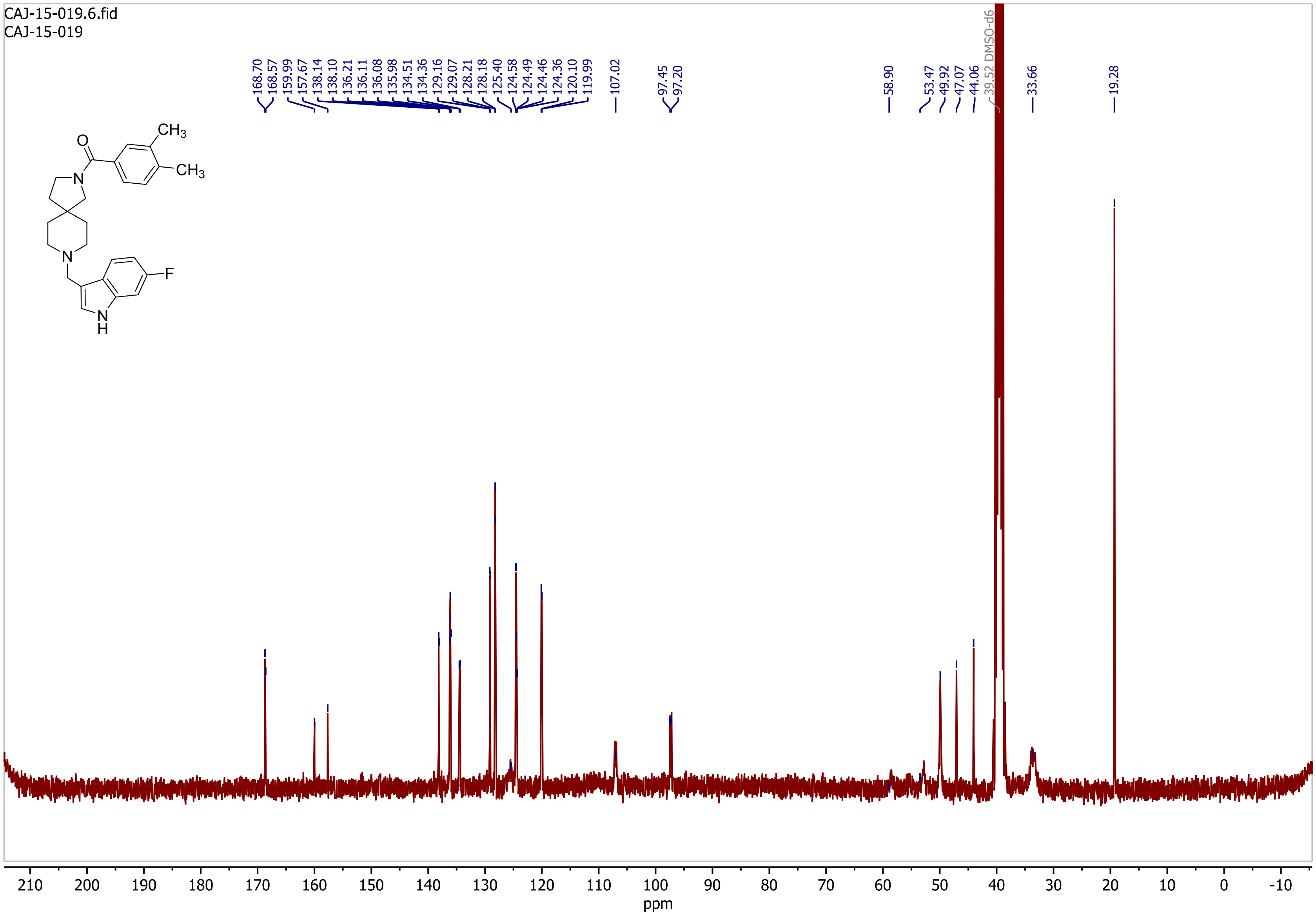

Figure S102: ^{13}C NMR spectrum of **41** in DMSO-d_6.

IV. DMPK Methods

i. Human Dopamine GPCR Binding Antagonist Assay

Percent inhibition was determined through radioligand binding assays at EuroFins (ww.Eurofins.com). Below are the reported methods used by Eurofins obtained through their site; further information about the assays can be obtained through the hyperlinked text below.

$D_{4.4}R$

CHO-K1 cells stably transfected with a plasmid encoding the human dopamine D4.4 receptor are used to prepare membranes in modified Tris-HCl buffer pH 7.4. A 100 μg‡ aliquot of membrane is incubated with 1.2 nM [^{3}H]Spiperone for 120 minutes at 25 °C. Non-specific binding is estimated in the presence of 10 μM haloperidol. Membranes are filtered and washed, and the filters are then counted to determine [^{3}H]Spiperone specifically bound. Compounds are screened at 10 μM.
Note: ‡Membrane protein may change from lot to lot, the concentration used will be adjusted if necessary.
D4.4 Human Dopamine GPCR Binding Antagonist Radioligand LeadHunter Assay - TW (eurofinsdiscovery.com)

D_1R

This assay measures binding of [^{3}H]SCH-23390 to human dopamine D1 receptors. CHO cells stably transfected with a plasmid encoding the human dopamine D1 receptor are used to prepare membranes in modified Tris-HCl pH 7.4 buffer using standard techniques. A 20 μg‡ aliquot of membrane is incubated with 1.4 nM [^{3}H]SCH-23390 for 120 minutes at 37 °C. Non-specific binding is estimated in the presence of 10 μM (+)-butaclamol. Membranes are filtered and washed 3 times and the filters are counted to determine [^{3}H]SCH-23390 specifically bound. Compounds are screened at 10 μM.
Note: ‡Membrane protein may change from lot to lot, the concentration used will be adjusted if necessary.
D1 Human Dopamine GPCR Binding Antagonist Radioligand LeadHunter Assay - TW (eurofinsdiscovery.com)

$D_{2L}R$

Human recombinant dopamine D2L receptor expressed in CHO cells are used in modified Tris-HCl buffer pH 7.4. A 20 μg‡ aliquot is incubated with 0.16 nM [^{3}H]Spiperone for 120 minutes at 25 °C. Non-specific binding is estimated in the presence of 10 μM haloperidol. Receptor proteins are filtered and washed, and the filters are then counted to determine [^{3}H]Spiperone specifically bound. Compounds are screened at 10 μM.

Note: ‡Membrane protein may change from lot to lot, the concentration used will be adjusted if necessary.
d2l-human-dopamine-gpcr-binding-antagonist-radioligand-leadhunter-assay-tw (eurofinsdiscovery.com)

$D_{2S}R$

This assay measures binding of [^{3}H]Spiperone to human dopamine D2S (D2B) receptors. CHO cells stably transfected with a plasmid encoding the human dopamine D2S receptor are used to prepare membranes in modified Tris-HCl pH 7.4 buffer using standard techniques. A 15 µg‡ aliquot of membrane is incubated with 0.16 nM [^{3}H]Spiperone for 120 minutes at 25 °C. Non-specific binding is estimated in the presence of 10 µM haloperidol. Membranes are filtered and washed 3 times and the filters are counted to determine [^{3}H]Spiperone specifically bound. Compounds are screened at 10 µM.
Note: ‡Membrane protein may change from lot to lot, the concentration used will be adjusted if necessary.
D2S Human Dopamine GPCR Binding Antagonist Radioligand LeadHunter Assay - TW (eurofinsdiscovery.com)

D_3R

This assay measures binding of [^{3}H]Spiperone to human dopamine D3 receptors. CHO cells stably transfected with a plasmid encoding the human dopamine D3 receptor are used to prepare membranes in modified Tris-HCl pH 7.4 buffer using standard techniques. A 10 µg‡ aliquot of membrane is incubated with 0.7 nM [^{3}H]Spiperone for 120 minutes at 37 °C. Non-specific binding is estimated in the presence of 25 µM S(-)-sulpiride. Membranes are filtered and washed 3 times, and the filters are counted to determine [^{3}H]Spiperone specifically bound. Compounds are screened at 10 µM.
Note: ‡Membrane protein may change from lot to lot, the concentration used will be adjusted if necessary.
D3 Human Dopamine GPCR Binding Antagonist Radioligand LeadHunter Assay - TW (eurofinsdiscovery.com)

ii. Materials

Potassium phosphate, ammonium formate, formic acid, magnesium chloride, carbamazepine, were purchased from Sigma-Aldrich (St. Louis, MO). Human liver microsomes (HLM) and rat liver microsomes (RLM) were obtained from BD Bioseceinces (Billerica, MA), with pooled gender at a concentration of 20 mg/mL protein. Microsomes were stored in an -80 °C freezer. All solvents used for bioanalysis were purchased from Sigma-Aldrich or Fisher Scientific (Waltham, MA) and were of high-performance liquid chromatography (HPLC) grade.

iii. Microsomal Stability

The metabolic stability of each compound was investigated in rat and human hepatic microsomes using substrate depletion methodology (% parent compound remaining). Prior to use, microsomes were removed from the freezer and allowed to thaw in a 37 °C water bath before placement on wet ice.

In separate 96-well plates for each time point, a mixture of 0.1M potassium phosphate-buffered (pH 7.4), 3 mM $MgCl_2$, and 2.5 mg/mL microsomes were pre-warmed at 37 °C for five minutes. Following the pre-incubation, 1 µM test compound and 1 mM NADPH (for 3, 7, 15, 25, or 45min time points) or buffer (for 0 min time point) were added to initiate the reaction. At the respective times, each plate's reaction was precipitated by the addition of 2 volumes of ice-cold acetonitrile containing an internal standard (carbamazepine, 50 nM). The plates were centrifuged at 4000 rcf (4 °C) for 5 min. The resulting supernatants were transferred and diluted 1:1 (supernatant: water) into new 96-well plates in preparation for LC/MS/MS analysis. Each compound was assayed in triplicate within the same 96-well plate. The in vitro half-life (t1/2, min, Eq. 1), intrinsic clearance (CLint, mL/min/kg, Eq. 2) and subsequent predicted hepatic clearance (CLhep, mL/min/kg, Eq. 3) was determined employing the following equations:

1) $t_{1/2}$ = ln(2) / k ; where k represents the slope from linear regression analysis (% test compound remaining)
2) CL_{int} = (0.693 / $t_{1/2}$) (reaction volume / mg microsomes) (45 mg microsomes / gram of liver) (20[a] gm of liver / kg body weight); [a] scale-up factors of 20 (human) and 45 (rat)
3) $CL\ hep = \dfrac{Q\ CLint}{Q + CLint}$

iv. Plasma Protein Binding

The protein binding of each compound was determined in rat or human plasma via equilibrium dialysis employing Single-Use RED Plates with inserts (ThermoFisher Scientific, Rochester, NY). Plasma (220 µL) was added to the 96 well plate containing test compound (5 µL) and mixed thoroughly. Subsequently, 200 µL of the plasma-compound mixture was transferred to the cis chamber (red) of the RED plate, with an accompanying 350 µL of phosphate buffer (25 mM, pH 7.4) in the trans chamber. The RED plate was sealed and incubated for 4 hours at 37°C with shaking. At completion, 50 µL aliquots from each chamber were diluted 1:1 (50 µL) with either plasma (cis) or buffer (trans) and transferred to a new 96 well plate, at which time ice-cold acetonitrile (2 volumes) was added to extract the matrices. The plate was centrifuged (3000 rpm, 10 min) and supernatants transferred and diluted 1:1 (supernatant: water) into a new 96 well plate, which was then sealed in preparation for LC/MS/MS analysis. Each compound was assayed in triplicate within the same 96-well plate.

v. Animal Care and Housing

All in vivo studies were carried out using adult male Sprague–Dawley rats (~300 g; 9-11 weeks old; Harlan, Indianapolis, IN), or age-matched adult male wild-type C57BL/6 mice (~40 g; 7-8 weeks old; Taconic Farms, Hudson, NY). Animals were group-housed under a 12/12 h light-dark cycle (lights on at 6 AM) with food and water available ad libitum unless stated elsewhere. All animal experiments were approved by the Vanderbilt University Animal Care and Use Committee, and experimental procedures conformed to guidelines established by the National Research Council Guide for the Care and Use of Laboratory Animals. All efforts were made to minimize animal suffering and the number of animals used.

vi. *In vivo* DMPK experimental

Compounds were formulated in a cassette format (5 compounds per formulation) in 10% ethanol, 39% PEG400, and 51% DMSO in sterile water at the concentration of (0.5 mL/kg) and administered intravenously to male Sprague- Dawley rats weighing 225 to 250 g (Harlan, Inc., Indianapolis, IN) at the dose of 0.2 mg/kg per compound. The rat blood was collected at 0.0333, 0.117, 0.25, 0.5, 1, 2, 4, 7, 24 hr. Trunk blood was collected in EDTA Vacutainer tubes, and plasma was separated by centrifugation and stored at -80 °C until analysis. Plasma JPET # 257204 4 was separated by centrifugation (4000 rcf, 4 °C) and stored at 80°C until analysis. The sample extraction of plasma (20 μL) was performed by a method based on protein precipitation using three volumes of ice-cold acetonitrile containing an internal standard (50 ng/mL carbamazepine). The samples were centrifuged (3000 rcf, 5 min) and supernatants transferred and diluted 1:1 (supernatant: water) into a new 96 well plate, which was then sealed in preparation for LC/MS/MS analysis.

vii. Liquid Chromatography-Mass Spectrometry Analysis

The analysis of *in vitro* and *in vivo* samples from microsomal stability, plasma protein binding, or *in vivo* pharmacokinetic experiments was determined employing LC-MS/MS with an electrospray ionization enabled 5500 Sciex instrument (Sciex, Foster City, CA) that was coupled to Agilent HPLC pumps and autosampler (Agilent Technologies, Santa Clara, CA). Analytes were separated by gradient elution using a Kinetex C18 column (2.1 × 50 mm, 5 μm; Phenomenex, Torrance, CA) warmed to 35 °C. Mobile phase A was 0.5% formic acid in water and mobile phase B was 0.5% formic acid in acetonitrile. The gradient started at 5% B after a 0.2-minute hold and was linearly increased to 95% B over 1.5 minute, held at 95% B for 0.2 minute, and returned to 5% B in 0.1 minute, followed by a re-equilibration (0.3 minute). The total run time was 2.8 minutes, and the HPLC flow rate was 0.5 ml/min. Mass spectral analyses were performed using multiple reaction monitoring, with transitions and voltages specific for each analyte using a Turbo Ion Spray source (source temp 500 °C) in positive ionization mode (5.0 kV spray voltage). Data were analyzed using OS Sciex Analyst 1.5.1 (SCIEX, Framingham, MA, USA). Non-compartmental analysis for in vivo pharmacokinetics was done using Phoenix 64 WinNonlin (Certara, L.P., St. Louis, MO, USA) Further analysis employed Graphpad Prism 10.1.0 (GraphPad Software, Boston, Massachusetts USA), Microsoft Excel version 2310 (Microsoft, Redmond, WA, USA).

viii. Kinetic Solubility

Method: Adapted standard shake flask method to run in 1 mL 96-deep-well plates at a concentration of 100 μM in McIlvaine Buffer at pH values of 2.2 and 6.8 from 10 mM DMSO stock solutions. Compounds are prepared in triplicate and incubated in buffer at room temperature for 18 hours while shaking at 700 RPM. After incubation the 96-deep-well plate is centrifuged at 5000 *g* for 10 minutes, half of the volume is transferred to another deep well plate and centrifuged again at 5000 *g* for 10 minutes. 200 μL are transferred from each well to a Greiner Bio-one 200 μL 96-well V-bottom plate and sealed. A six-point calibration curve is prepared for each compound ranging from 100 μM down to 0.5 μM. All samples are analyzed via UV-UHPLC on an Agilent 1290 Infinity (binary pump, auto-sampler, column compartment at 55 ˚C, and PDA) with a Phenomenex Kinetex EVO C18, 50 x 1 mm, 1.7 μm, 100 Å column, at 0.5 mL. Injections of 3 μL are analyzed with gradient elution using Milli-Q water with 0.05% trifluoroacetic acid (A1) and acetonitrile with 0.05% trifluoroacetic acid (B1) from 95:5 A1/B1 to 5:95 A1/B1 over 1.3 minutes with a 0.2 minute hold at 5:95 A1/B1. Wavelengths at 215 nm and 254 nm are monitored, peaks are integrated, and the peak area and peak height are used with linear regression analysis from the calibration curves to determine the solubility values.[1-3]

Solubility Data for Selected Compounds:

VUID	Compound No.	Solubility (μM)	
		pH = 2.2	pH = 6.8
**VU6053634**	**4**	95.3	88.9
**VU6063659**	**5**	83.4	87.0
**VU6063524**	**6**	89.4	83.1
**VU6063528**	**9**	84.2	31.6
**VU6063525**	**12**	88.3	87.4
**VU6063946**	**13**	90.5	88.2
**VU6063242**	**20**	86.4	82.7
**VU6063244**	**21**	84.6	81.5
**VU6063243**	**22**	87.1	83.0
**VU6063265**	**27**	85.0	79.7
**VU6063267**	**29**	91.9	85.3
**VU6063269**	**31**	82.7	79.6
**VU6063270**	**32**	91.2	87.0
**VU6063271**	**33**	92.9	87.7

ix. References

(1) Lin, B.; Pease, J. H., A high throughput solubility assay for drug discovery using microscale shake-flask and rapid UHPLC–UV–CLND quantification. *Journal of Pharmaceutical and Biomedical Analysis* **2016,** *122,* 126-140.

(2) Yamashita, T.; Dohta, Y.; Nakamura, T.; Fukami, T., High-speed solubility screening assay using ultra-performance liquid chromatography/mass spectrometry in drug discovery. *Journal of Chromatography A* **2008,** *1182* (1), 72-76.

(3) McIlvaine, T. C., A buffer solution for colorimetric comparison. *Journal of Biological Chemistry* **1921,** *49* (1), 183-186.

V. Computational Methods

i. Virtual Screening

Dataset Preparation:

We trained our ligand-based quantitative structure-activity relationship (QSAR) models using publicly available datasets in PubChem for orthosteric antagonists of the D_2, D_3, D_4, and D_5 dopamine receptors. For the purposes of this study, we only included in our training datasets only molecules for which there are confirmatory screens (i.e., K_i, K_d, or IC_{50} values) performed. In cases where multiple confirmatory measurements were available for a molecule, the values were averaged. Molecules with repeat measurements were excluded if their activity values exhibited standard deviations exceeding 10x the average.

Molecules were standardized via the following steps: (i) molecules were obtained in SDF format, (ii) explicit hydrogen atoms were added, (iii) all molecules were neutralized, (iv) any salts or other non-defining molecular entities in the SDF were removed; (v) a single 3D conformer was generated for all molecules using the CORINA conformer generator[1]; (vi) molecules were excluded if they lacked valid Gasteiger atom types.[2] Subsequently, features were generated for each molecule using a collection of 2D and 3D signed property autocorrelations and other metrics as previously described in Mendenhall & Meiler 2016[3] as the 'Minimal' feature set.

Neural Network Model Architecture and Training:

Our QSAR models consisted of a multitask artificial neural network (ANN) for each target (D_4R) and off-target (D_2R, D_3R, and D_5R) protein. Each ANN contained a single hidden layer with 32 neurons. The input and hidden layers employed 5% and 25% dropout, respectively. The models were trained to predict the likelihood of a molecule being active at or below 5 specific thresholds (1 nM, 10 nM, 100 nM, 1000 nM, and 10,000 nM). Each molecule therefore contained a classification label (i.e., 1.0 for active or 0.0 for inactive) for each result. A parity of 0.5 was utilized, such that model outputs for any result label above 0.5 indicated true (i.e., active at that result label), and mean absolute error was utilized for the loss. Sigmoid activation functions were used throughout. Calibration curves were generated to associate predicted probabilities with model output values, which we refer to here and elsewhere as 'localPPV'.[2,3]

Screening of Compound Libraries:
Utilizing our QSAR models, we virtually screened the 2019q3-4 Enamine REAL database and LifeChemicals discovery set. All test molecules were prepared to be consistent with the training dataset.

ii. Property-based Small Molecule Flexible Alignment

We used BCL::MolAlign to perform property-based small molecule flexible alignment of select virtual hits and experimentally validated screening molecules to the selective D_4R antagonist L-745,870 in its experimentally determined binding pose.[4,5] We used default properties, property weights, Monte Carlo – Metropolis (MCM) iterations, refinement iterations, and independent cycles for BCL::MolAlign. We modified the default conformer generation settings in BCL::MolAlign to be consistent with the update to the BCL conformer generator.[6] Specifically, conformers were generated utilizing the conformation comparer SymmetryRMSD with a tolerance of 0.25 Å. For each molecule, 4000 iterations were performed during the conformer generation process. Up to 5 unique poses from the best scoring alignments were saved for further analysis.

iii. Protein-ligand Docking and Interaction Energy Analysis

Molecular Docking:
For our docking studies, we made use of a crystallographic structure of mouse D_4R bound to L745,870 (PDB ID 6IQL)[5] as template for human D_2R and D_4R. We prepared the receptor as a monomer and performed comparative modeling in Rosetta[7] and refined with backbone-constrained Rosetta Relax (iterative sidechain repacking and minimization) to generate the human receptors. We reasoned that because we were looking specifically for selective D_4R antagonists, we should dock our small molecule ligands into receptor pockets pre-organized to accept a known selective antagonist. Prior to docking, protonation states were assigned to each molecule using the Molecular Operating Environment (MOE) protomers pKa tool.[8] The molecular docking phase utilized RosettaLigand[9] employing the Talaris2014 Rosetta energy function.[10] For each molecule, BCL::Conf was used to generate up to 250 unique conformers. BCL::Conf was run with 8000 iterations. Conformations were compared using the SymmetryRMSD metric at a tolerance of 0.25 Å.[6] Subsequently, up to 10,000 candidate poses were generated during docking – docking poses were initialized from each of the possible 5 best alignments from BCL::MolAlign, and 2000 poses were generated from each alignment. The docked poses selected as most likely were those with the best Talaris2014 interaction energies.

Interaction Energy Analysis:

Following docking, select compounds were further evaluation with quantum mechanical (QM) interaction energy analysis. All QM calculations were performed using GAMESS 2023 R1[11] and the density functional tight-binding method DFTB3-D3[12,13] and aqueous SMD solvent. A region of the protein-ligand complex was selected for QM calculations (i.e., the QM model system) by identifying residues within 4.0 Å of any ligand atom unless otherwise specified in the main text. Breakpoints in the backbone were capped with hydrogen atoms. We performed geometry optimization with DFTB3-D3 using the QM model system. Atoms previously involved in covalent connections to residues not included in the QM model system were frozen in space during geometry optimization. Following geometry optimization, two calculations were performed on the QM model system to estimate the interaction energies: one for the protein-ligand complex and another where the ligand was separated from the protein by 100 Å.

iv. Molecular Dynamics Simulations

Molecular dynamics simulations were conducted using the AMBER 21 software package.[14] The ff19SB protein force field[15], OPC water model[16], Lipid21 lipids force field[17], and GAFF2 force field for ligands were employed. Protein systems were embedding in explicit POPC membranes and solvated with explicit OPC waters using the packmol-memgen program[18] in AmberTools. The system was neutralized and brought to a concentration of 150 mM NaCl.[19] We utilized periodic boundary conditions and the Particle Mesh Ewald (PME) method for long-range electrostatic interactions with a 12.0 Angstrom electrostatics cutoff. Covalent bonds involving hydrogen atoms were constrained with the SHAKE algorithm.[20]

We sequentially performed minimization of the solvent, solute, and then full system with 1,000 steps of steepest gradient descent followed by 4,000 steps of conjugate gradient descent for each stage. Afterward, the system was heated to 100K in the canonical (NVT) ensemble and allowed to equilibrate for 1.0 ns. The system was then heated in the isothermal-isobaric (NPT) ensemble at 1 bar with a Monte Carlo barostat and semi-isotropic scaling to 310 K over another 1.0 ns. Hydrogen mass repartitioning was performed on non-solvent atoms to enable a 4.0 fs production timestep. A Langevin thermostat with a collision frequency of 1.0 ps^{-1} was used throughout all heating and production stages.

v. Surface Electrostatic Potential and Molecular Orbital Analysis

QM calculations were again performed using the GAMESS software (version 2023 R1).[11] Geometry optimizations were performed as described above with the tight-binding density functional method DFTB3-D3. Energy calculations from which surface electrostatic potential and molecular orbital analyses were performed using DFT at the wB97X-D/6-31G(d) level of theory[21] on select atom subsets as shown in the corresponding figures. For the visualization of surfaces and orbitals, the wxMacMolPlt tool was employed.[22]

vi. References

(1) Gasteiger, J.; Rudolph, C.; Sadowski, J. Automatic generation of 3D atomic coordinates for organic molecules. *Tetrahedron Computer Methodology* **1990**, *3* (6c), 537-547.

(2) Brown, B. P.; Vu, O.; Geanes, A. R.; Kothiwale, S.; Butkiewicz, M.; Lowe, E. W.; Mueller, R.; Pape, R.; Mendenhall, J.; Meiler, J. Introduction to the BioChemical Library (BCL): An Application-Based Open-Source Toolkit for Integrated Cheminformatics and Machine Learning in Computer-Aided Drug Discovery. *Front. Pharmacol.* **2022**, *13*, Technology and Code. DOI: 10.3389/fphar.2022.833099.

(3) Mendenhall, J.; Meiler, J. Improving quantitative structure-activity relationship models using Artificial Neural Networks trained with dropout. *Journal of computer-aided molecular design* **2016**, *30* (2), 177-189. DOI: 10.1007/s10822-016-9895-2.

(4) Brown, B. P.; Mendenhall, J.; Meiler, J. BCL::MolAlign: Three-Dimensional Small Molecule Alignment for Pharmacophore Mapping. *J. Chem. Inf. Model.* **2019**, *59* (2), 689-701. DOI: 10.1021/acs.jcim.9b00020.

(5) Zhou, Y.; Cao, C.; He, L.; Wang, X.; Zhang, X. C. Crystal structure of dopamine receptor D4 bound to the subtype selective ligand, L745870. *eLife* **2019**, *8*, e48822. DOI: 10.7554/eLife.48822.

(6) Mendenhall, J.; Brown, B. P.; Kothiwale, S.; Meiler, J. BCL::Conf: Improved Open-Source Knowledge-Based Conformation Sampling Using the Crystallography Open Database. *J. Chem. Inf. Model.* **2021**, *61* (1), 189-201. DOI: 10.1021/acs.jcim.0c01140.

(7) Song, Y.; DiMaio, F.; Wang, R. Y.; Kim, D.; Miles, C.; Brunette, T.; Thompson, J.; Baker, D. High-resolution comparative modeling with RosettaCM. *Structure* **2013**, *21* (10), 1735-1742. DOI: 10.1016/j.str.2013.08.005.

(8) *Molecular Operating Environment (MOE)*; Chemical Computing Group (CCG): Montreal, Quebec, Canada H3A 2R7, 2007. http://www.chemcomp.com/research-jrnl.htm.

(9) Meiler, J.; Baker, D. ROSETTALIGAND: protein-small molecule docking with full side-chain flexibility. *Proteins* **2006**, *65* (3), 538-548. DOI: 10.1002/prot.21086.

(10) O'Meara, M. J.; Leaver-Fay, A.; Tyka, M. D.; Stein, A.; Houlihan, K.; DiMaio, F.; Bradley, P.; Kortemme, T.; Baker, D.; Snoeyink, J.; et al. Combined Covalent-Electrostatic Model of Hydrogen Bonding Improves Structure Prediction with Rosetta. *J. Chem. Theory Comput.* **2015**, *11* (2), 609-622. DOI: 10.1021/ct500864r.

(11) Barca, G. M. J.; Bertoni, C.; Carrington, L.; Datta, D.; De Silva, N.; Deustua, J. E.; Fedorov, D. G.; Gour, J. R.; Gunina, A. O.; Guidez, E.; Harville, T.; Irle, S.; Ivanic, J.; Kowalski, K.; Leang, S. S.; Li, H.; Li, W.; Lutz, J. J.; Magoulas, I.; Mato, J.; Mironov, V.; Nakata, H.; Pham, B. Q.; Piecuch, P.; Poole, D.; Pruitt, S. R.; Rendell, A. P.; Roskop, L. B.; Ruedenberg, K.; Sattasathuchana, T.; Schmidt, M. W.; Shen, J.; Slipchenko, L.; Sosonkina, M.; Sundriyal, V.; Tiwari, A.; Galvez Vallejo, J. L.; Westheimer, B.; Włoch, M.; Xu, P.;

Zahariev, F.; Gordon, M. S. Recent Developments in the General Atomic and Molecular Electronic Structure System. *J. Chem. Phys.* **2020**, *152* (15), 154102. DOI: 10.1063/5.0005188.
(12) Brandenburg, J. G.; Grimme, S. Accurate Modeling of Organic Molecular Crystals by Dispersion-Corrected Density Functional Tight Binding (DFTB). *The Journal of Physical Chemistry Letters* **2014**, *5* (11), 1785-1789. DOI: 10.1021/jz500755u.
(13) Gaus, M.; Cui, Q.; Elstner, M. DFTB3: Extension of the Self-Consistent-Charge Density-Functional Tight-Binding Method (SCC-DFTB). *J. Chem. Theory Comput.* **2011**, *7* (4), 931-948. DOI: 10.1021/ct100684s.
(14) D.A. Case, I. Y. B.-S., S.R. Brozell, D.S. Cerutti, T.E. Cheatham, III, V.W.D. Cruzeiro, T.A. Darden, R.E. Duke, D. Ghoreishi, M.K. Gilson, H. Gohlke, A.W. Goetz, D. Greene, R Harris, N. Homeyer, S. Izadi, A. Kovalenko, T. Kurtzman, T.S. Lee, S. LeGrand, P. Li, C. Lin, J. Liu, T. Luchko, R. Luo, D.J. Mermelstein, K.M. Merz, Y. Miao, G. Monard, C. Nguyen, H. Nguyen, I. Omelyan, A. Onufriev, F. Pan, R. Qi, D.R. Roe, A. Roitberg, C. Sagui, S. Schott-Verdugo, J. Shen, C.L. Simmerling, J. Smith, R. Salomon-Ferrer, J. Swails, R.C. Walker, J. Wang, H. Wei, R.M. Wolf, X. Wu, L. Xiao, D.M. York and P.A. Kollman. AMBER 2018. 2018.
(15) Tian, C.; Kasavajhala, K.; Belfon, K. A. A.; Raguette, L.; Huang, H.; Migues, A. N.; Bickel, J.; Wang, Y.; Pincay, J.; Wu, Q.; et al. ff19SB: Amino-Acid-Specific Protein Backbone Parameters Trained against Quantum Mechanics Energy Surfaces in Solution. *J. Chem. Theory Comput.* **2020**, *16* (1), 528-552. DOI: 10.1021/acs.jctc.9b00591.
(16) Izadi, S.; Anandakrishnan, R.; Onufriev, A. V. Building Water Models: A Different Approach. *The Journal of Physical Chemistry Letters* **2014**, *5* (21), 3863-3871. DOI: 10.1021/jz501780a.
(17) Dickson, C. J.; Walker, R. C.; Gould, I. R. Lipid21: Complex Lipid Membrane Simulations with AMBER. *J. Chem. Theory Comput.* **2022**, *18* (3), 1726-1736. DOI: 10.1021/acs.jctc.1c01217.
(18) Schott-Verdugo, S.; Gohlke, H. PACKMOL-Memgen: A Simple-To-Use, Generalized Workflow for Membrane-Protein–Lipid-Bilayer System Building. *J. Chem. Inf. Model.* **2019**, *59* (6), 2522-2528. DOI: 10.1021/acs.jcim.9b00269.
(19) Joung, I. S.; Cheatham, T. E. Determination of Alkali and Halide Monovalent Ion Parameters for Use in Explicitly Solvated Biomolecular Simulations. *J. Phys. Chem. B* **2008**, *112* (30), 9020–9041. https://doi.org/10.1021/jp8001614.
(20) Miyamoto, S.; Kollman, P. A. Settle: An analytical version of the SHAKE and RATTLE algorithm for rigid water models. *J. Comput. Chem.* **1992**, *13* (8), 952-962. DOI: 10.1002/jcc.540130805.
(21) Chai, J.-D.; Head-Gordon, M. Long-Range Corrected Hybrid Density Functionals with Damped Atom–Atom Dispersion Corrections. *Phys. Chem. Chem. Phys.* **2008**, *10* (44), 6615. DOI: 10.1039/b810189b.
(22) Bode, B. M.; Gordon, M. S. Macmolplt: A Graphical User Interface for GAMESS. *J. Mol. Graph. Model.* **1998**, *16* (3), 133–138. DOI: 10.1016/S1093-3263(99)00002-9.

vii. Supplemental Figures

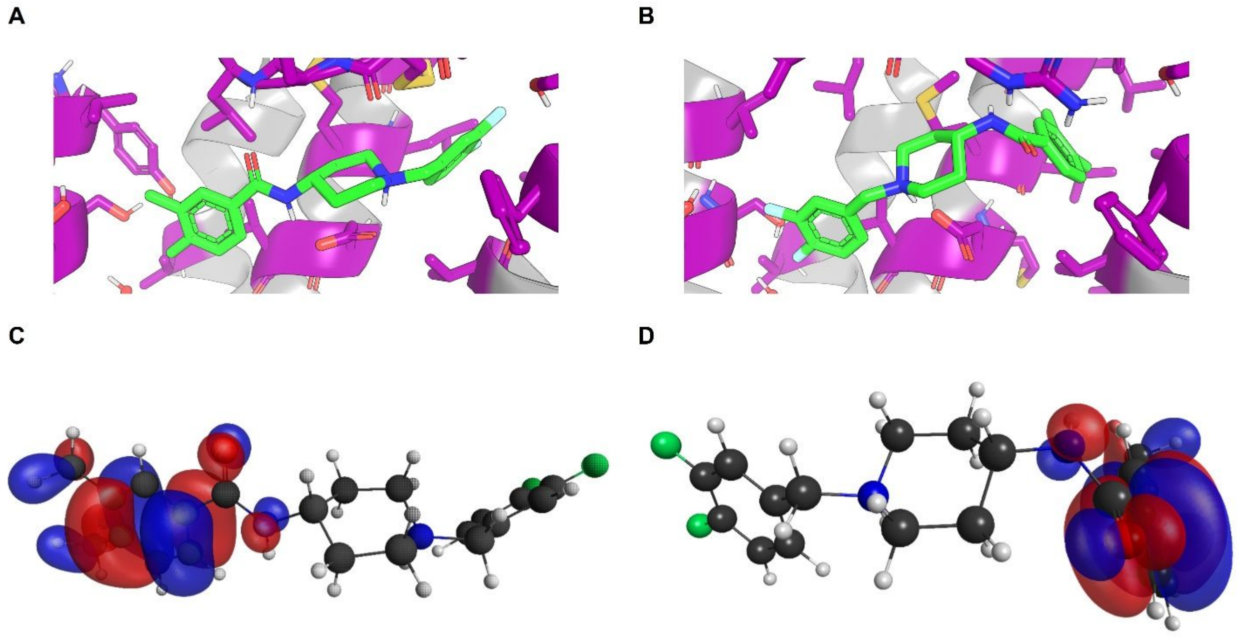

Figure S103. Discriminating two alternative binding modes in Compound *VU6052469*. (A) Docked binding pose 1 of compound *VU6052469*. **(B)** Docked binding pose 2 of compound *VU6052469*. **(C)** Highest occupied molecular orbital (HOMO) of compound *VU6052469* in pose 1 and **(D)** pose 2.

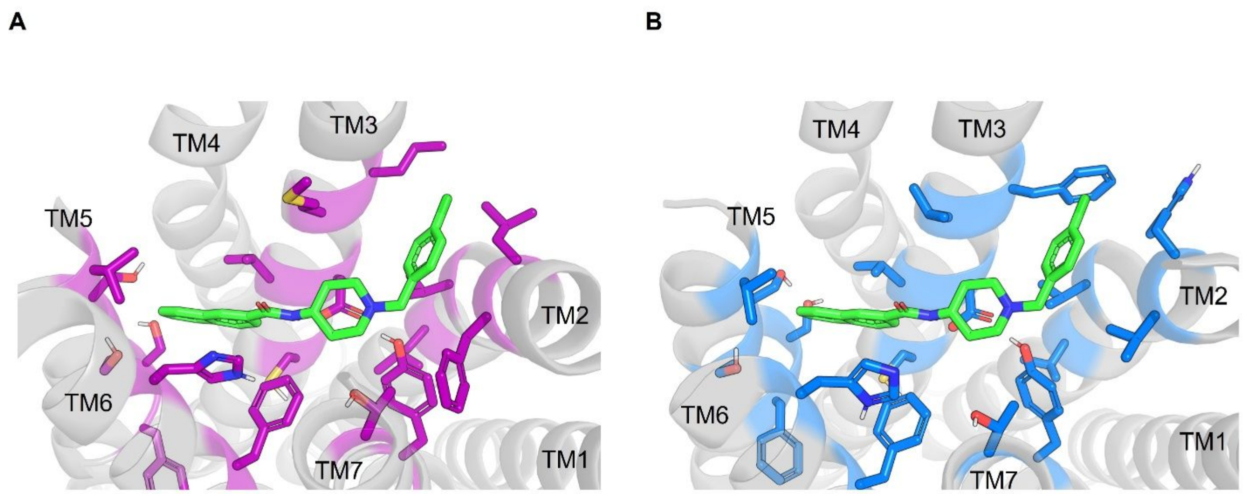

Figure S104. Carato compound docked into D_4R and D_2R. (A) Carato compound docked into D_4R and **(B)** D_2R. Sidechains of pocket residues colored in purple and blue for D_4R and D_2R, respectively.

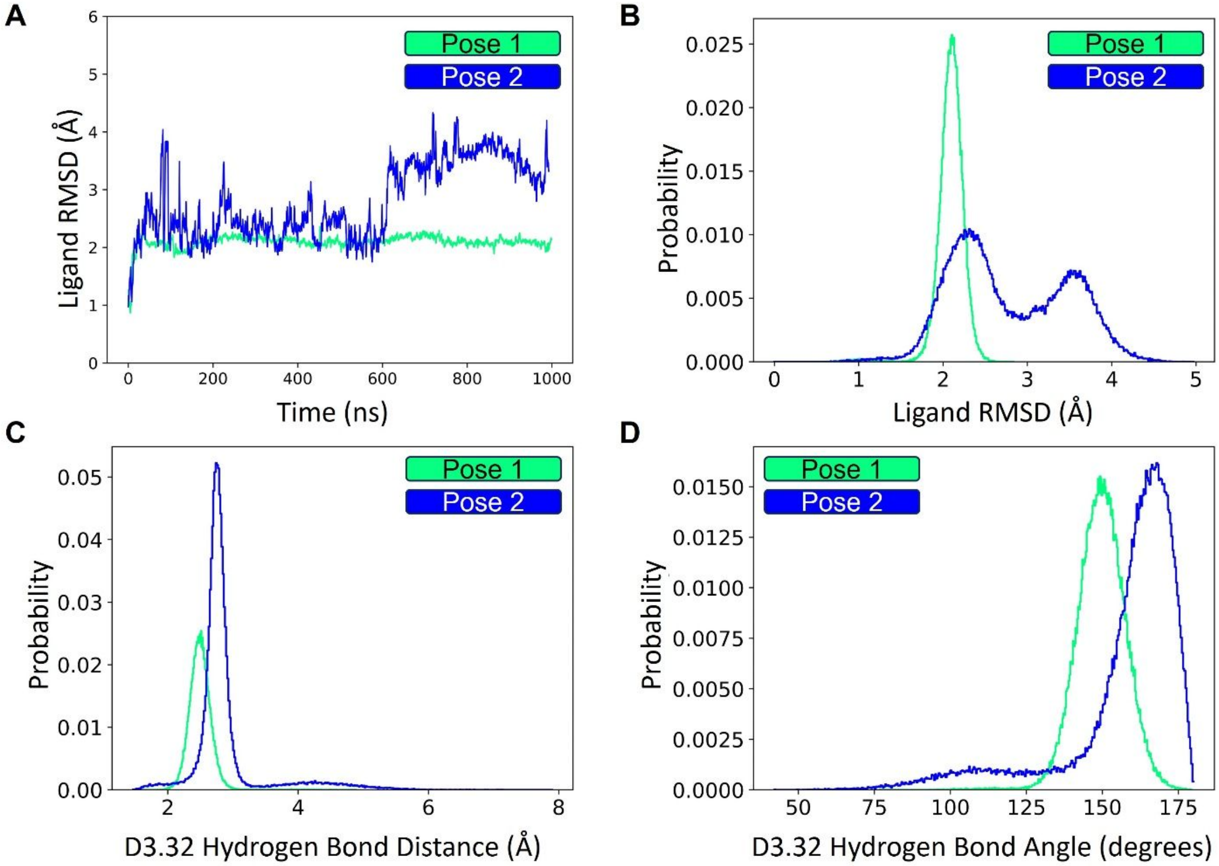

Figure S105. Molecular dynamics simulations of Compound 4 in D_4R. (A) Time-dependent RMSD of compound **4** in complex with D_4R. RMSD values are given as the average over a 100-frame window. **(B)** Probability distribution of compound **4** RMSD values over the course of the simulation. **(C)** Probability distribution for the hydrogen bond distance between the protonated piperidine core of compound **4** and the conserved D3.32 sidechain. Distances are taken between the hydrogen atom of the protonated piperidine core and the center of mass of the sidechain carboxylic acid oxygen atoms. **(D)** Probability distribution of the hydrogen bond angle formed by the protonated piperidine core of compound **4** and the conserved D3.32 sidechain. The angle is defined by the nitrogen and hydrogen atoms of the protonated piperidine core and the center of mass of the sidechain carboxylic acid oxygen atoms.

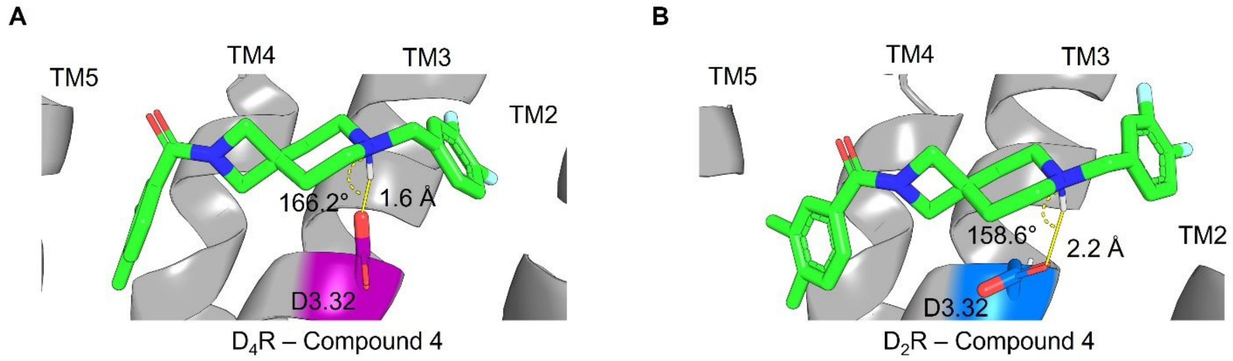

Figure S106. Compound 4 hydrogen bond geometry with conserved D3.32 in D_2R and D_4R. Compound **4** in docked to **(A)** D_4R or **(B)** D_2R.

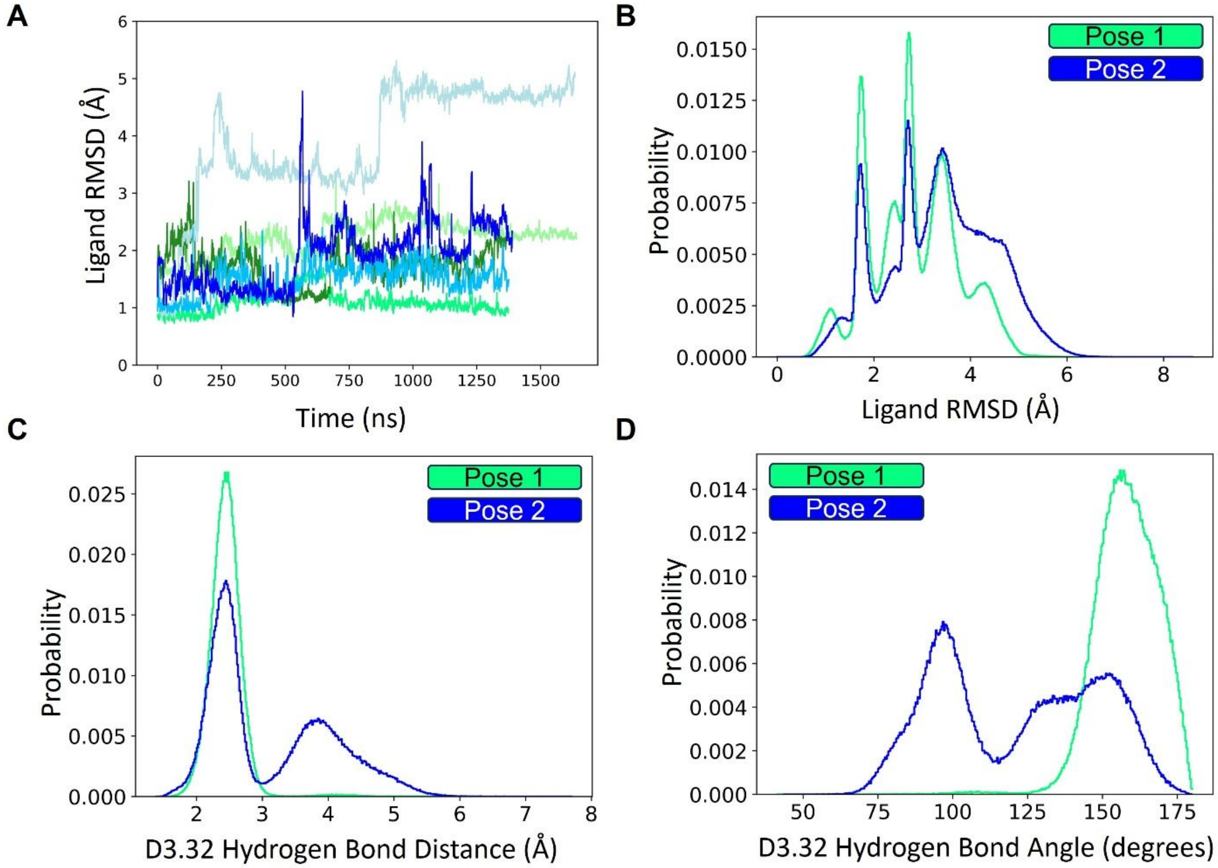

Figure S107. Molecular dynamics simulations of Compound 5 in D_4R. (A) Time-dependent RMSD of compound **5** in complex with D_4R. RMSD values are given as the average over a 100-frame window. Shades of green and blue correspond to pose 1 and 2, respectively. **(B)** Probability distribution of compound **5** RMSD values over the course of the simulation. **(C)** Probability distribution for the hydrogen bond distance between the protonated piperidine core of compound **5** and the conserved D3.32 sidechain. Distances are taken between the hydrogen atom of the protonated piperidine core and the center of mass of the sidechain carboxylic acid oxygen atoms. **(D)** Probability distribution of the hydrogen bond angle formed by the protonated piperidine core of compound **5** and the conserved D3.32 sidechain. The angle is defined by the nitrogen and hydrogen atoms of the protonated piperidine core and the center of mass of the sidechain carboxylic acid oxygen atoms.

viii. Structures of Validated HTS Hits

Below are structures and VU numbers for HTS hit compounds displaying greater than 85% inhibition at 10 μM.

Structure	VUID	Structure	VUID
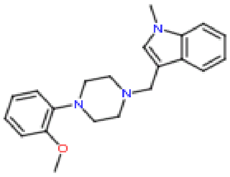	VU6046597	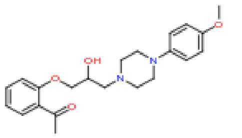	VU6046643
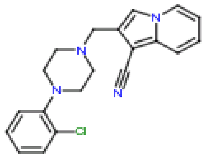	VU6046614	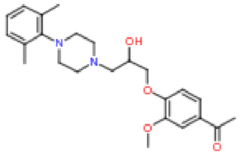	VU6046644
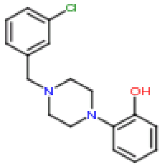	VU6046615	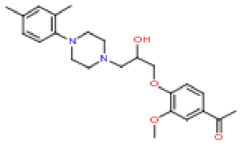	VU6046645
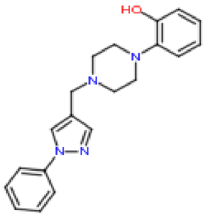	VU6046620	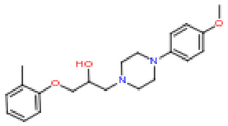	VU6046647
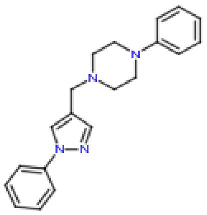	VU6046624	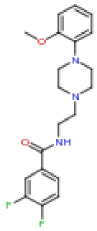	VU6046662
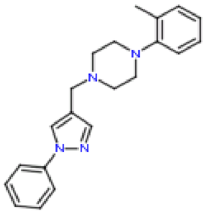	VU6046626	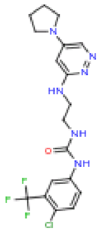	VU6046667

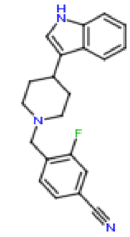	VU6046628	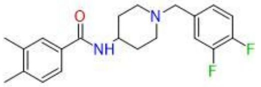	VU6052469
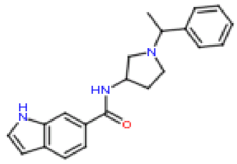	VU6046629	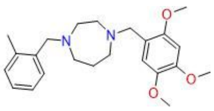	VU6064099
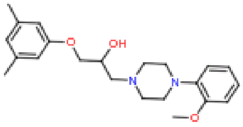	VU6046639		

VI. Metabolite Profiling

Experimental: Hepatocyte Incubations

Substrate concentration: 10 µM

Hepatocyte density: 1 million cells/mL

Hepatocyte information: Pool of 100 mixed-gender human (Lot# 2210212, viability 85.2%) and Male SD rat (Lot# 2310140, viability 82.0%)

Incubation: 37 °C for 4 hours under air with 5% CO_2

Sample Preparation for LC/MS Analysis

- Reaction was halted by addition of 250 µL ice cold acetonitrile (1:1 vol.) to reaction well
- Centrifuged at 4000rcf for 10 minutes
- Aliquoted supernatant to 96 well plate, sealed, and stored in refrigerated compartment of instrument
- Samples analyzed by LC/MS

Experimental: LC/MS Conditions

LC/UV/MS system: Vanquish (pumps, autosampler and PDA) interfaced to Orbitrap Fusion Lumos mass spectrometer
UHPLC column: Accucore C18+ column, 100 x 2.1 mm, 1.5 µm
UHPLC mobile phase gradient:

Time (min)	0.05% TFA in Water	0.05% TFA in ACN	Flow rate (µL/min)
0	95	5	400
1	95	5	400
20	70	30	400
20	5	95	400
24	5	95	400

Mass Spectrometer: Full scan (m/z 100-1000) and data dependent MS/MS analysis

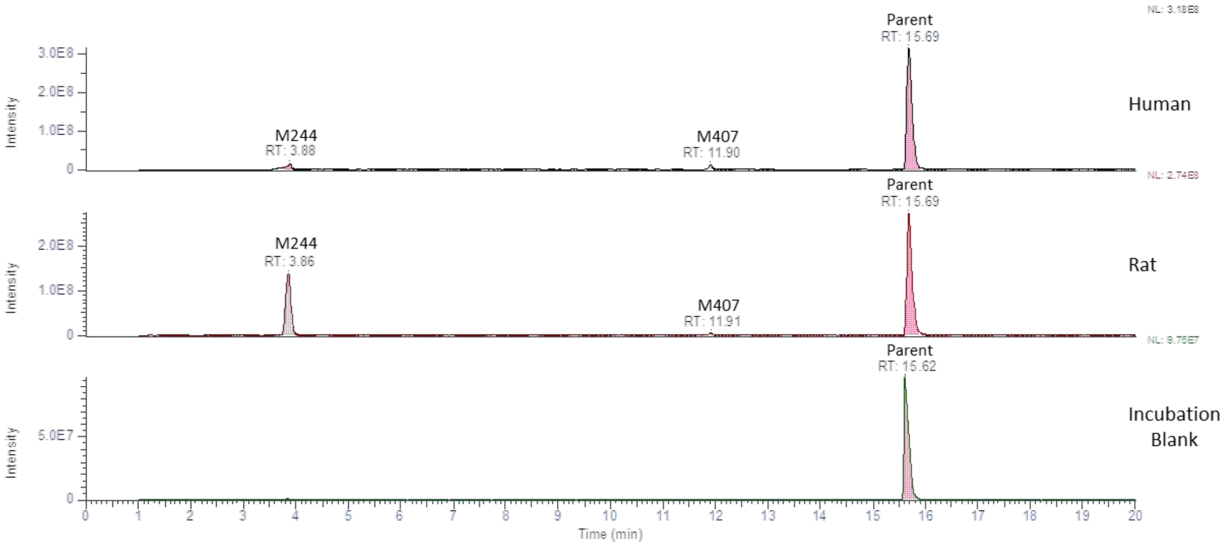

ID	RT	Biotransformation(s)	m/z	Theoretical	Δppm	% Parent Related Material Human	Rat
M244	3.88	De-alkylation	245.1648	245.1648	0.00	6.31	32.41
M407	11.9	Oxidation	408.2082	408.2082	0.00	5.74	1.83
VU6063271	15.65	Parent	392.2133	392.2141	2.04	87.94	65.76

Putative Metabolic Scheme of VU6063271

VU6063271 (Parent, P) [P+H] m/z 392.2133

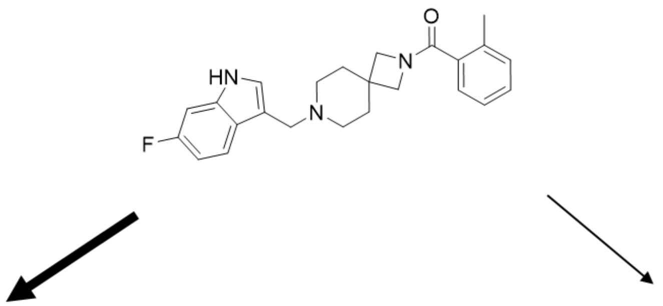

M244 [P-C_9H_6FN] m/z 245.1648

M407 [P+O] m/z 408.2082

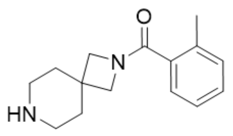

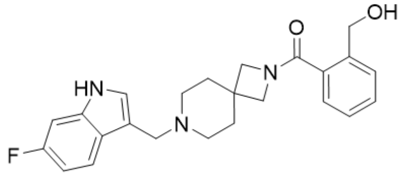

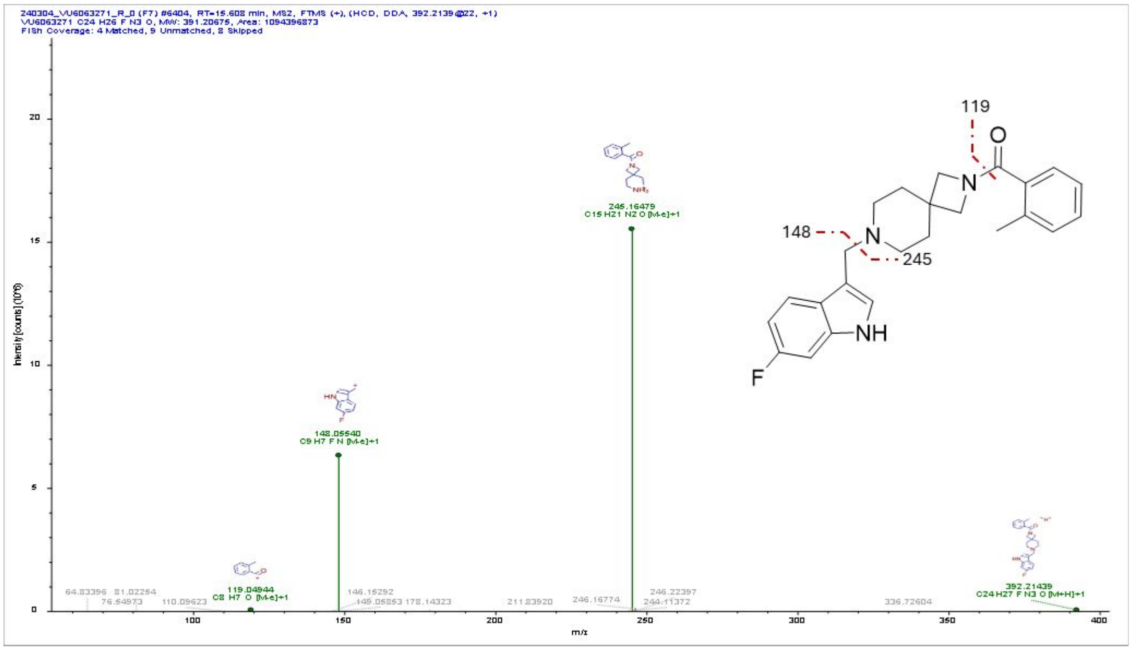
240304_VU6063271_R_0 (F7) #6404, RT=15.608 min, MS2, FTMS (+), (HCD, DDA, 392.2139@22, +1)
VU6063271 C24 H26 F N3 O, MW: 391.20675, Area: 1094396873
FISh Coverage: 4 Matched, 9 Unmatched, 2 Skipped
119
148
245
NH
F
O
N
245.16479
C15 H21 N2 O [M-e]+1
148.05540
C9 H7 F N [M-e]+1
119.04944
C8 H7 O [M-e]+1
392.21439
C24 H27 F N3 O [M+H]+1
m/z

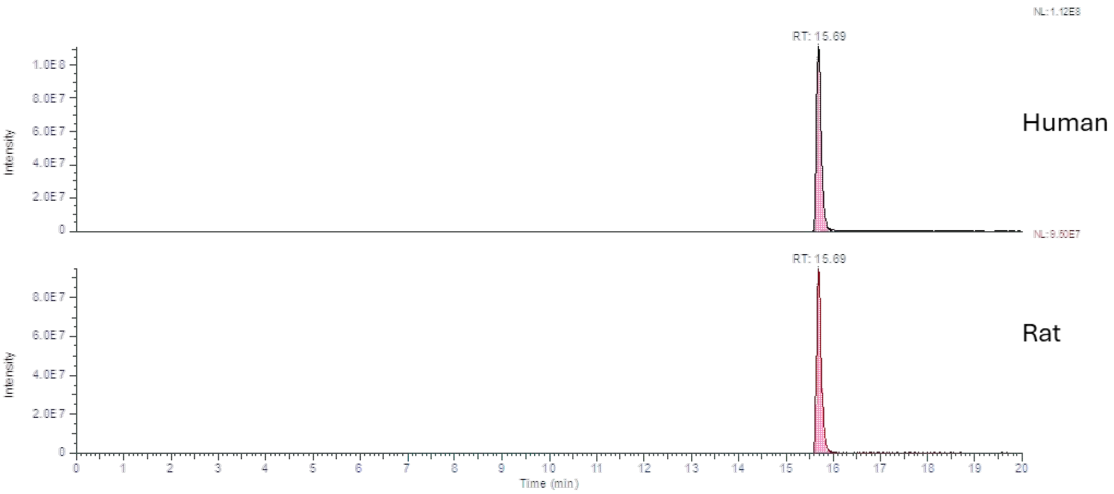
NL: 1.12E8
RT: 15.69
Human
RT: 15.69
Rat
Intensity
Time (min)

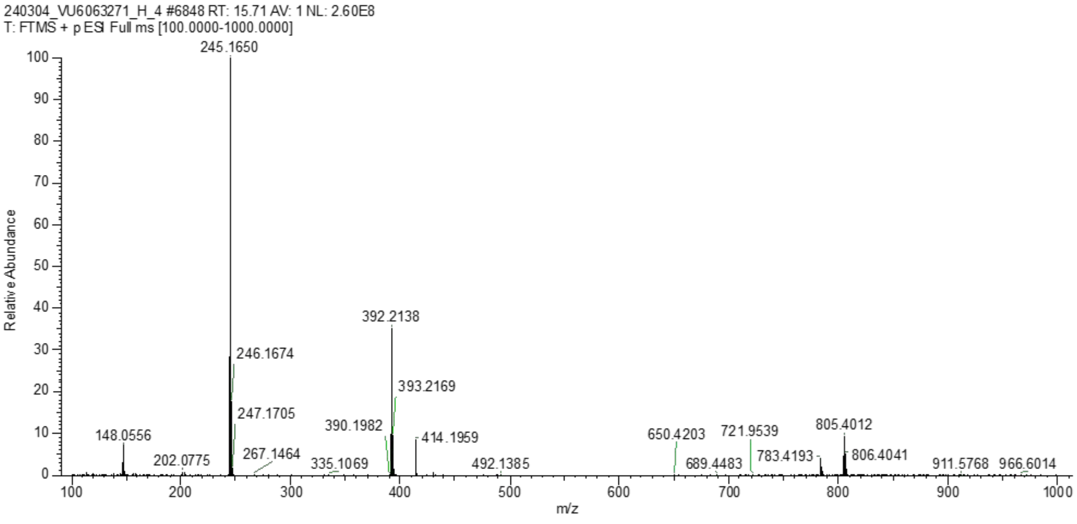
240304_VU6063271_H_4 #6848 RT: 15.71 AV: 1 NL: 2.60E8
T: FTMS + p ESI Full ms [100.0000-1000.0000]
245.1650
392.2138
246.1674
393.2169
247.1705
148.0556
390.1982
414.1959
805.4012
Relative Abundance
m/z

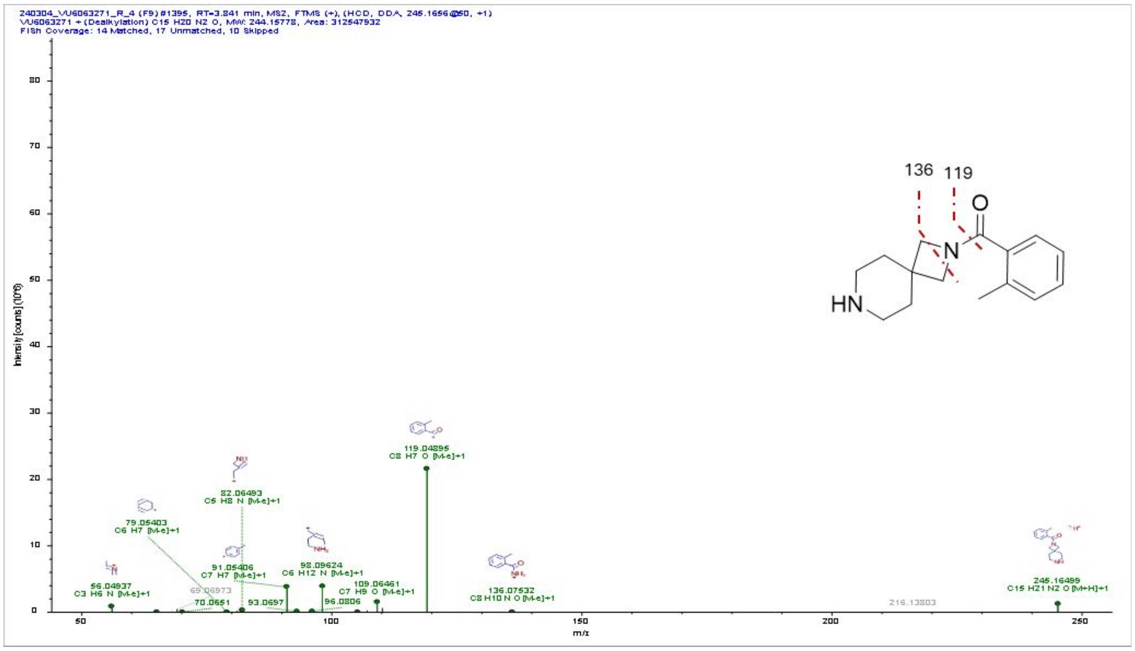
240304_VU6063271_R_4 (F9) #1395, RT=3.841 min, MS2, FTMS (+), (HCD, DDA, 245.1656@50, +1)
VU6063271 + (Dealkylation) C15 H20 N2 O, MW: 244.15778, Area: 312547932
FISh Coverage: 14 Matched, 17 Unmatched, 10 Skipped
Intensity [counts] (10^6)
m/z
136
119
O
N
HN
119.04895
C8 H7 O [M-e]+1
82.06493
C5 H8 N [M-e]+1
79.05403
C6 H7 [M-e]+1
91.05406
C7 H7 [M-e]+1
98.09624
C6 H12 N [M-e]+1
109.06461
C7 H9 O [M-e]+1
56.04937
C3 H6 N [M-e]+1
136.07532
C8 H10 N O [M-e]+1
245.16499
C15 H21 N2 O [M+H]+1
216.13803

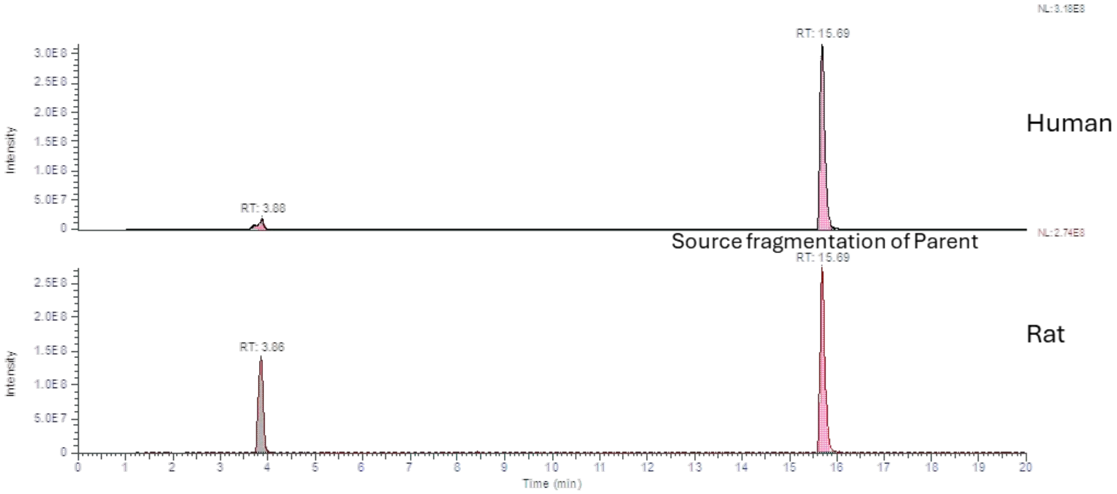
NL: 3.18E8
RT: 15.69
Human
RT: 3.88
Source fragmentation of Parent
NL: 2.74E8
RT: 15.69
Rat
RT: 3.86
Intensity
Time (min)

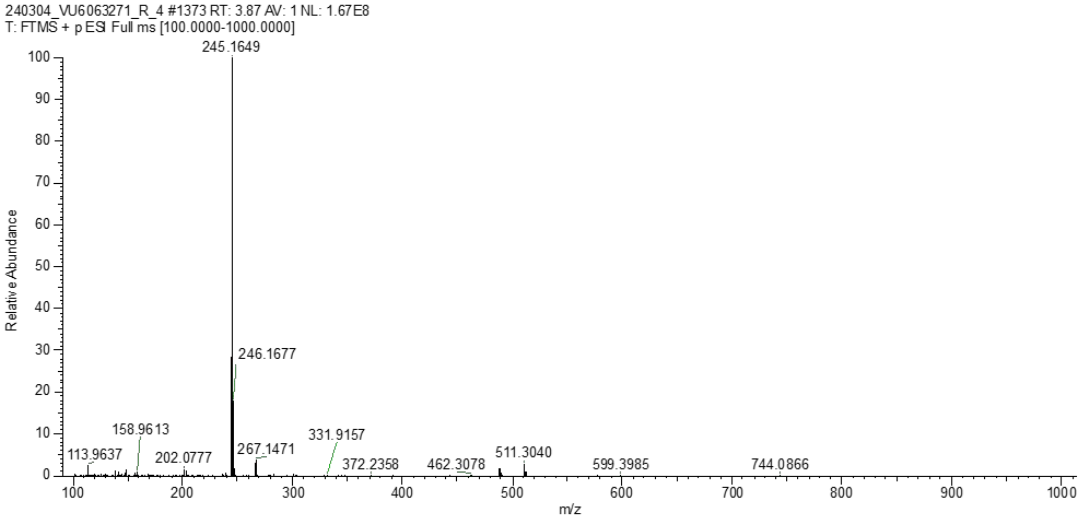
240304_VU6063271_R_4 #1373 RT: 3.87 AV: 1 NL: 1.67E8
T: FTMS + p ESI Full ms [100.0000-1000.0000]
245.1649
246.1677
158.9613
113.9637
202.0777
267.1471
331.9157
372.2358
462.3078
511.3040
599.3985
744.0866
Relative Abundance
m/z

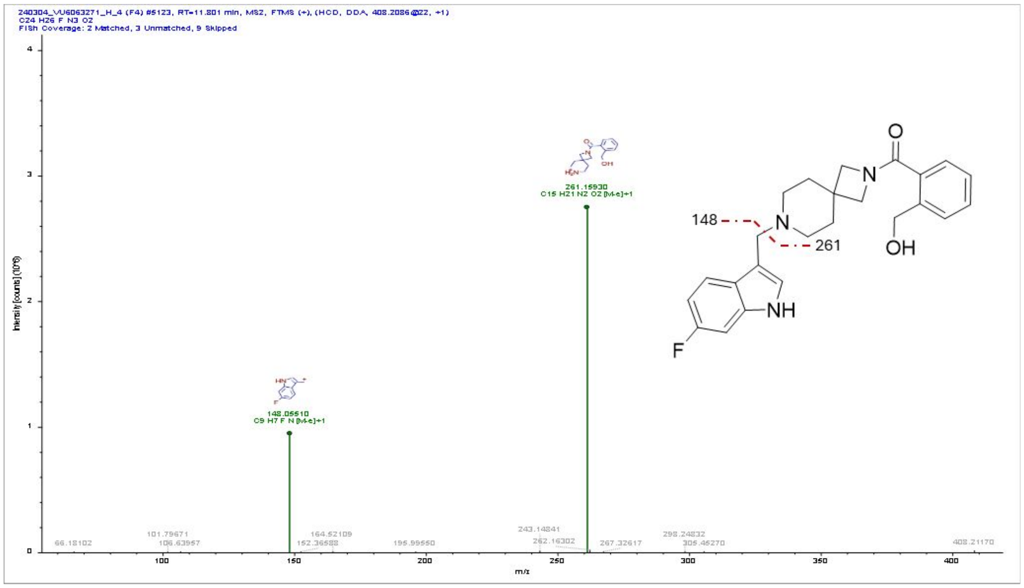
240304_VU6063271_H_4 (F4) #5123, RT=11.801 min, MS2, FTMS (+), (HCD, DDA, 408.2086@22, +1)
C24 H26 F N3 O2
FISh Coverage: 2 Matched, 3 Unmatched, 9 Skipped
Intensity [counts] (10^6)
261.15930
C15 H21 N2 O2 [M-e]+1
148.05510
C9 H7 F N [M-e]+1
148
261
N
O
OH
NH
F
m/z

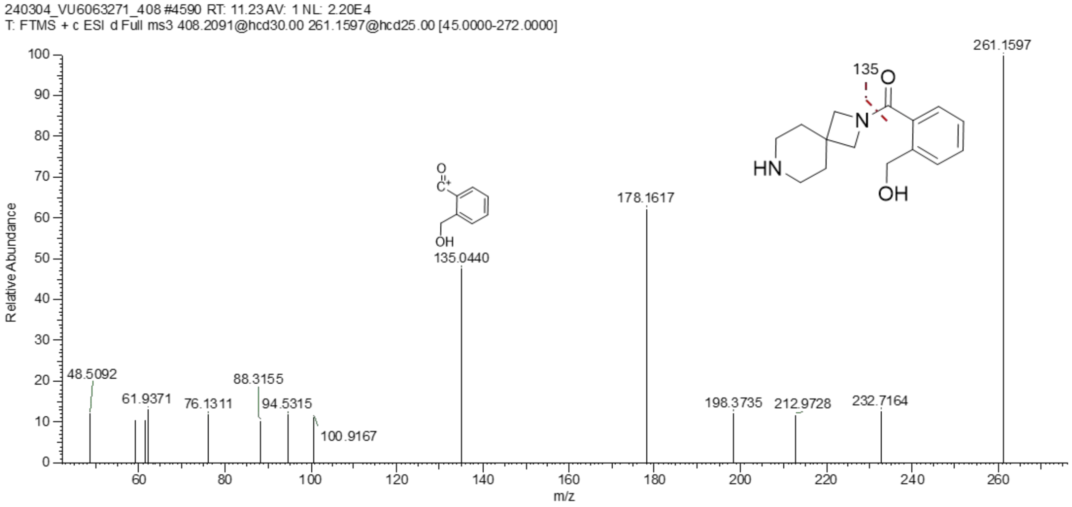
240304_VU6063271_408 #4590 RT: 11.23 AV: 1 NL: 2.20E4
T: FTMS + c ESI d Full ms3 408.2091@hcd30.00 261.1597@hcd25.00 [45.0000-272.0000]
Relative Abundance
261.1597
178.1617
135.0440
48.5092
61.9371
76.1311
88.3155
94.5315
100.9167
198.3735
212.9728
232.7164
135
HN
N
O
OH
m/z

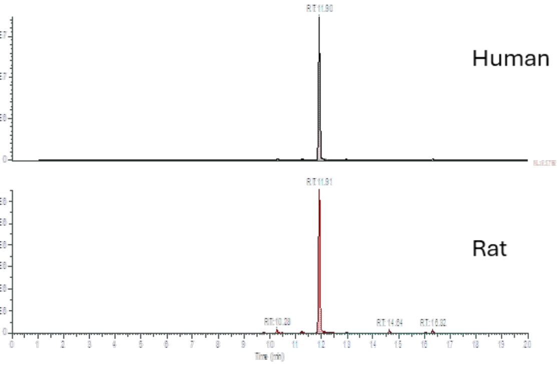
Human
Rat
Intensity
Time (min)

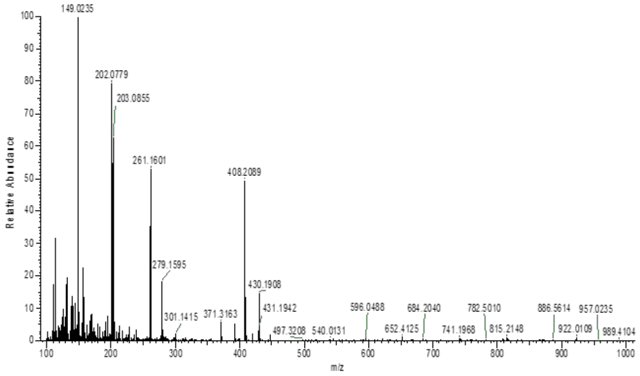
VU6063271_Pooled_H #1600 RT: 11.92 AV: 1 NL: 1.93E6
T: FTMS + p ESI Full ms [100.0000-1000.0000]
Relative Abundance
149.0235
202.0779
203.0855
261.1601
408.2089
279.1595
430.1908
431.1942
301.1415
371.3163
497.3208
540.0131
596.0488
652.4125
684.2040
741.1968
782.5010
815.2148
886.5614
922.0109
957.0235
989.4104
m/z
